# An Aza-Enolate Strategy Enables Iridium-Catalyzed Enantioselective Hydroalkenylations of Minimally Polarized Alkenes en Route to Complex N-Aryl β^2^-Amino Acids

**DOI:** 10.1021/jacs.4c07519

**Published:** 2024-08-06

**Authors:** Fenglin Hong, Craig M. Robertson, John F. Bower

**Affiliations:** Department of Chemistry, University of Liverpool, Crown Street, Liverpool L69 7ZD, United Kingdom

## Abstract

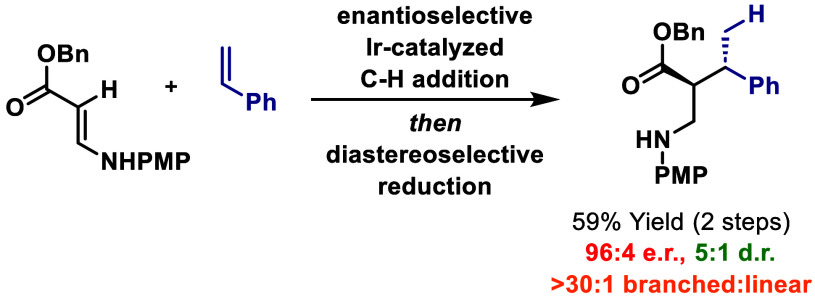

Cationic Ir(I)-complexes modified with homochiral diphosphines promote the hydroalkenylative cross-coupling of β-(arylamino)acrylates with monosubstituted styrenes and α-olefins. The processes are dependent on the presence of an NH unit, and it is postulated that metalation of this generates an iridium aza-enolate that engages the alkene during the C–C bond forming event. The method offers high branched selectivity and enantioselectivity and occurs with complete atom economy. Diastereocontrolled reduction of the products provides β^2^-amino acids that possess contiguous stereocenters.

Reactivity frameworks that allow the regio- and enantioselective intermolecular addition of C–H bonds across minimally activated alkenes (i.e., styrenes and α-olefins)^1^ are of important topical interest because they can underpin new classes of C(sp^2^)–C(sp^3^) and C(sp^3^)–C(sp^3^) cross-coupling that operate within the confines of a “high efficiency” regime (Scheme 1A).^2^ Specifically, this approach (a) avoids substantial prefunctionalization, thereby enhancing step economy,^3^ (b) harnesses readily available alkenes as reaction partners, and (c) occurs with complete atom economy.^4^ Unfortunately, there are a limited range of such processes, with perhaps the most prominent examples being RajanBabu’s Ni-catalyzed asymmetric hydrovinylations of styrenes.^5^ Enantioselective hydroacylations offer another promising avenue, although intermolecular variants involving minimally activated alkenes require specific directing groups to enforce regiocontrol.^6^ We have demonstrated that directed Ir-catalyzed activation of aryl C(sp^2^)–H bonds can underpin enantioselective hydroarylations of styrenes and α-olefins, *where branched regioselectivity is under catalyst control* (Scheme 1B).^7a,7b^ This area was advanced significantly by Li and co-workers, who outlined mechanistically related hydroalkenylation reactions that employ enamides.^7c^

**Scheme 1:**
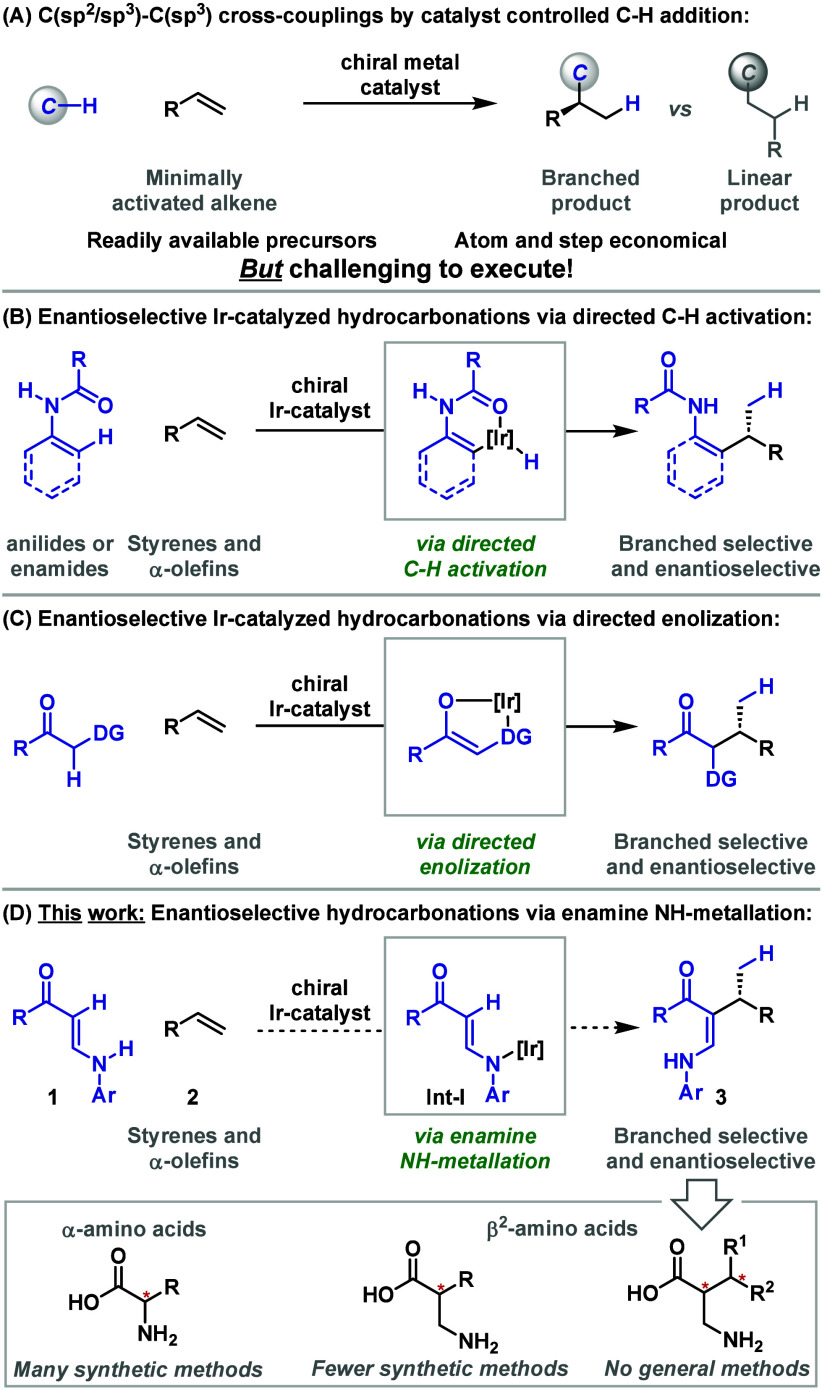
Introduction

The C(sp^2^)–C(sp^3^) cross-couplings in Scheme 1B are notable for the exceptional levels of branched and enantioselectivity that are achieved. To advance this area, we recently discovered Ir-catalyzed directing group controlled enolization reactions that allow the addition of activated C(sp^3^)–H bonds across styrenes and α-olefins, thereby providing a prototype framework for hydroalkylative C(sp^3^)–C(sp^3^) cross-couplings (Scheme 1C).^8^ Compared to Scheme 1B, where a classical carbometalation mechanism is likely operative,^7a,7c^ these processes are distinct because C–C bond formation is proposed to occur via an Ir-enolate. To exploit this unusual mechanistic paradigm further, we have sought other ways of initiating Ir-enolate-based reactivity, and specifically, we have targeted processes that avoid the requirement for additional directing functionality. To this end, we considered whether NH-metalations of persistent enamines **1** might generate Ir-aza-enolates (**Int-I**) and whether these might then engage alkenes **2** in enantioselective hydroalkenylation reactions to provide C(sp^2^)–C(sp^3^) cross-coupling products **3** (Scheme 1D). In principle, stereocontrolled reduction of **3** can then be exploited to provide β^2^-amino acids possessing vicinal stereocenters (Scheme 1D, box).^9^ The synthetic issues associated with accessing β^2^-amino acids have been highlighted by Seebach and co-workers.^9e^ The current study addresses an unmet synthetic challenge in this area as well as offers significant advances from the viewpoint of reactivity.

Based on our earlier work using N-directing groups,^8a,10^ we elected to focus initially on the branched selective C–H alkylation of *N*-phenyl enamine **1a** with styrene **2a** (Table 1). In preliminary experiments, we found Ir(cod)_2_BARF/(*R*)-BINAP (5 mol %) was effective in delivering target **3aa** in 90% yield and 93:7 er at 100 °C in *o*-DCB (entry 1). Complete branched selectivity was observed, and **3aa** was formed as a 12:1 mixture of *Z*:*E* geometric isomers. As corroborated by subsequent studies (vide infra), the geometry of the enamine is likely labile under the reaction conditions and the *Z*-isomer is favored because it is stabilized by a hydrogen bond between the N–H unit and the ester carbonyl. To improve enantioselectivity, a variety of homochiral diphosphine ligands were assayed (e.g., **L2–8**, entries 2–8), and this revealed notable improvements to yield, enantioselectivity, and *Z*/*E* selectivity by using (*R*)-MeO-BIPHEP (96% yield, 96:4 er, >20:1 *Z*/*E*, entry 6). Other solvents offered similar efficiencies (e.g., entries 9 and 10). The nature of the counterion on the Ir-precatalyst is critical, as replacement of BARF with more strongly coordinating variants inhibited the process (entries 11–13). Use of an analogous Rh precatalyst was ineffective (entry 14). The loading of styrene **2a** could be decreased from 300 to 200 mol % (entry 15), although subsequent scope studies were conducted using 300 mol % of the alkene partner. 100 °C was found to be the optimal reaction temperature; decreasing this to 90 °C led to a reduction in yield as a result of incomplete conversion of **1a**, whereas increased temperatures resulted in less clean reaction profiles.

**Table 1:**
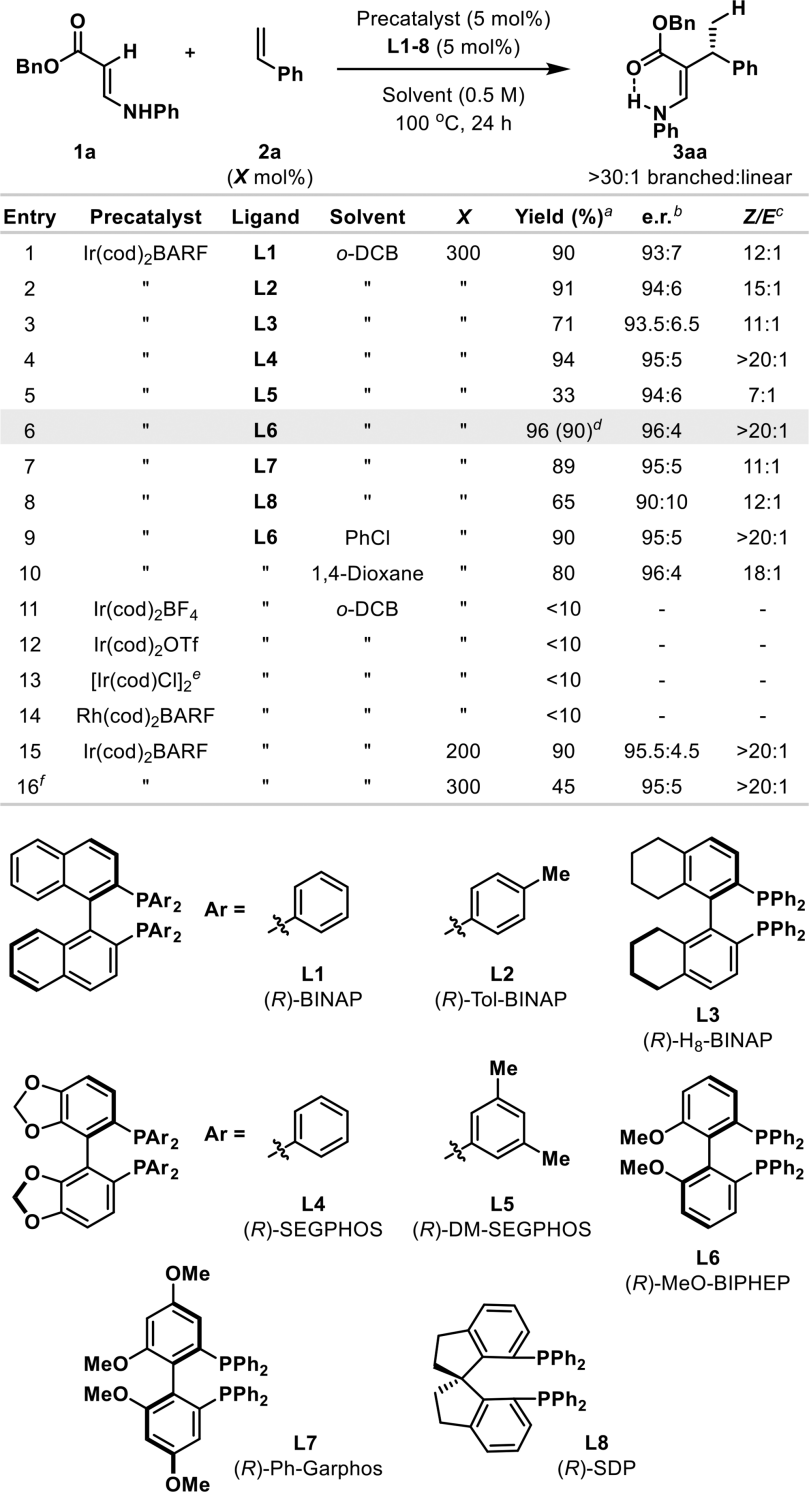
Reaction Optimization^g^

a Determined by ^1^H NMR analysis using 1,3,5-trimethoxybenzene as a standard.

b Determined by chiral SFC analysis.

c Determined by ^1^H NMR analysis of the crude mixture.

d Isolated yield.

e 2.5 mol % of the dimeric precatalyst was used.

f The reaction was performed at 90 °C.

g *o*-DCB = 1,2-dichlorobenzene. Reaction conditions: **1a** (0.1 mmol), precatalyst (5 mol %), **L1–8** (5 mol %), **2a** (0.2 or 0.3 mmol), solvent (0.2 mL), 100 °C, 24 h, in a sealed Schlenk tube.

Table 2 outlines the scope of the enantioselective alkene hydroalkenylation cross-coupling process. Note that reaction times were adjusted for each example based on TLC monitoring of reaction progress. The ester unit of the enamine can be varied, as demonstrated by the efficient formation of **3ba**-**3da** (Table 2A). Interestingly, analogous ketone and amide-based systems were ineffective, and targets **3ea** and **3fa** were not observed.^11^ We also conducted an evaluation of the electronics of the N-aryl unit so that we could establish working parameters for this component (Table 2B). More electron rich 4-methoxy- and 4-hydroxy-phenyl units were efficient, with **3ha** and **3ia** generated in high yield. Electron poor 4-fluoro system **1j** was somewhat less efficient and generated **3ja** in only moderate yield. Interestingly, high efficiencies were observed for the formation of 4-chorophenyl system **3ka**. In all of these cases, the enantioselectivities were similar.

**Table 2:**
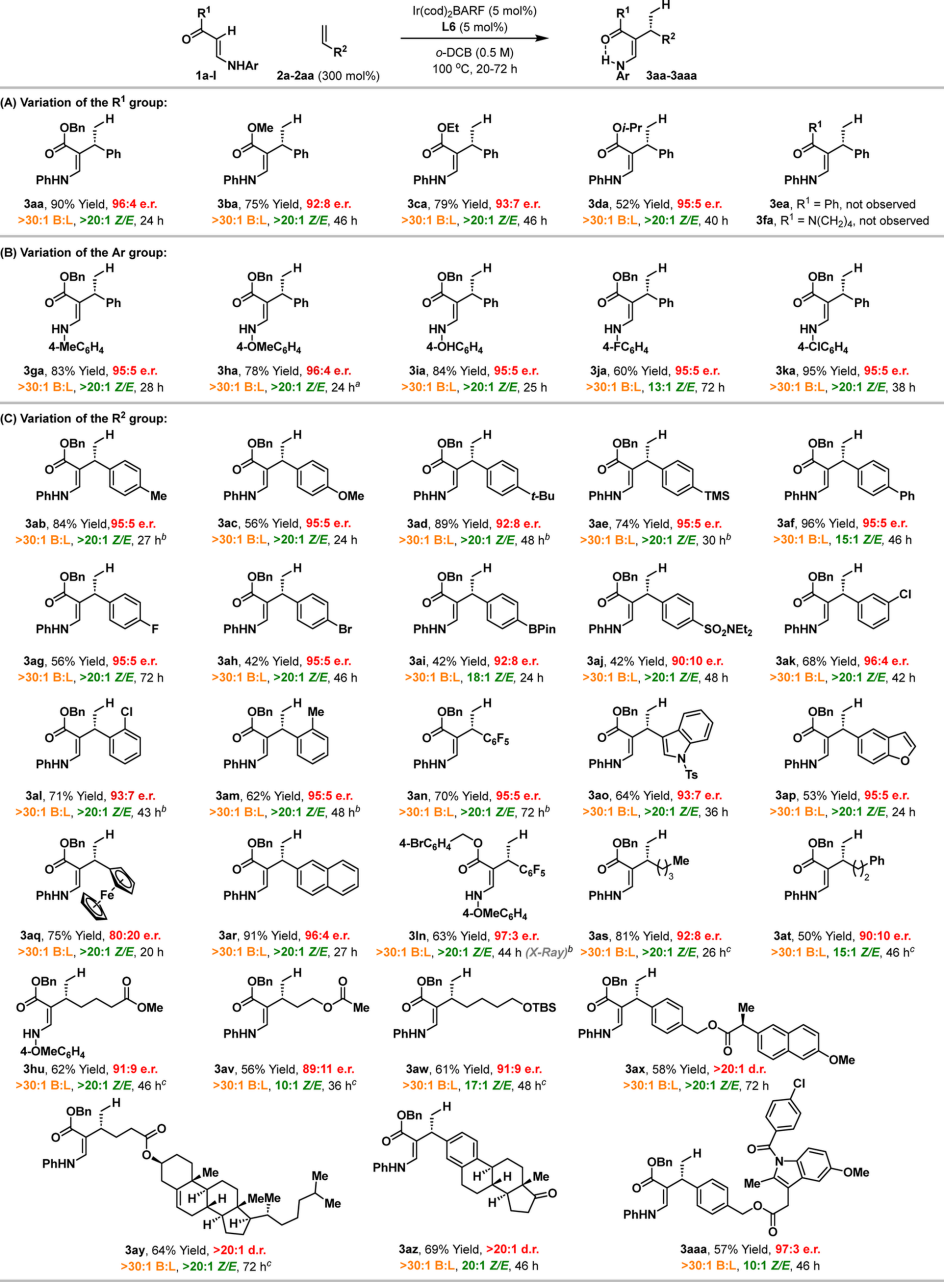
Scope of the Cross-Coupling Process

a The antipode (*ent-***3ha**) was synthesized using (*S*)-MeO-BIPHEP (*ent.-***L6**) (see the Supporting Information).

b Alkene (500 mol %), (*R*)-MeO-BIPHEP (7.5 mol %), and [Ir(cod)_2_]BARF (7.5 mol %) were used.

c (*R*)-SEGPHOS (**L4**) was used.

Clearly the most significant aspect of scope lies in the range of alkene partners that the method can tolerate. As shown in Table 2C, we have established that the protocol is excellent in this regard. Using enamine **1a** as the test substrate, cross-couplings with a variety of electronically and sterically distinct styrenes proceeded with uniformly high enantioselectivities and, in general, with very good levels of chemical efficiency. For example, similar results were obtained for pentafluorophenyl and naphthyl systems **3an** and **3ar**, demonstrating a wide electronic range. *Ortho*-chloro and methylstyrenes efficiently participated to provide **3al** and **3am**, indicating that the method is not overly sensitive to sterically demanding units. Potentially labile or sensitive functionality is tolerated, including C(sp^2^)–Br bonds (**3ah**), a BPin unit (**3ai**), a TMS unit (**3ae**), and an indole (**3ao**). These results highlight the mildness of the process, which operates under essentially neutral conditions and in the absence of additives. Cross-coupling of enamine **1l** with pentafluorostyrene **2n** gave target **3ln**, which was analyzed by single crystal X-ray diffraction; this allowed the determination of absolute stereochemistry and revealed the aforementioned hydrogen bonding interaction between the N–H unit and ester carbonyl.

Beyond styrenic systems, processes involving less activated aliphatic alkenes can also be achieved, with **L4** offering optimal efficiencies in these cases (Table 2C). For example, hydroalkenylation of hex-1-ene **2s** with **1a** provided **3as** in excellent yield and 92:8 er. Other aliphatic alkenes cross-coupled to provide **3at** to **3aw** with similar selectivities. These processes also demonstrate that aliphatic esters (**3hu** and **3av**) and O-TBS groups (**3aw**) are stable to the reaction conditions. The applicability of the method to more functional-group-rich alkene substrates was validated by efficient cross-coupling to deliver **3ax** to **3aaa**. Here, esters (**3ax**, **3ay**, **3aaa**), amides (**3aaa**), aryl chlorides (**3aaa**), ketones (**3az**), spectator alkenes (**3ay**), and epimerizable stereocenters (**3ax**) were all transferred through the cross-coupling without incident. At the current level of development, more highly substituted alkenes do not participate efficiently (Figure S1), and the development of this aspect is ongoing.

As alluded to in the introduction, our primary synthetic motivation for developing the cross-couplings described here was to gain access to challenging β^2^-amino acids. This required the identification of a method for the diastereoselective reduction of the enamine unit of the products (Scheme 2A). For this purpose, we selected **3ha** as a model substrate because the resulting PMP-protected amine should be easy to deprotect under oxidative conditions.^12^ Enamine reduction under substrate control was chemically efficient using a variety of protocols (e.g., PtO_2_/H_2_,^13^ Ir(cod)_2_BARF/H_2_,^14^ TFA/NaBH_3_CN),^15^ but these offered minimal diastereocontrol (up to 2:1 dr; see the Supporting Information for details). Accordingly, potential catalyst controlled methods were evaluated, focusing initially on hydrogenation procedures using homochiral Ir-, Rh-, or Ru-catalysts;^14,16,17^ however, these efforts did not lead to improved outcomes (see the Supporting Information for details). After extensive investigations, we found that the combination of **Cat. A** and Cl_3_SiH provides **4** in 5:1 dr and 75% yield.^18^ The diastereomers of **4** were readily separable such that enrichment could be achieved by chromatographic purification. Conversion of **4** to N-Ts system **5** provided crystals suitable for X-ray diffraction, and this allowed the assignment of the relative configuration.^19^ The reduction conditions were adapted from Zhang and co-workers’ protocol that involves systems related to **3ha**; the process is proposed to proceed via reversible enamine to imine tautomerization, in advance of C=N reduction.^18^ Interestingly, the conditions were also diastereoselective for the reduction of *ent***-3ha**, which delivered *ent***-4** in 6:1 dr, showing that the process is primarily under substrate control. Thus, sequential Ir-catalyzed cross-coupling and reduction steps control the absolute and relative configuration of the stereocenters of the target. This sequence also has applications beyond amino acid synthesis; for example, diastereoselective reduction of **3al** provided **6** in 6:1 dr, and Pd-catalyzed cyclization of this provided tetrahydroquinoline **7** in 46% yield.^20^ The cross-coupling products can also undergo “global” decarboxylative reduction (H_2_, Pd/C) to provide γ-stereogenic amines (**8a**–**c**) Scheme 2B).^21^ These are challenging to access by other means, and their availability here demonstrates another strategic benefit of combining Ir-catalyzed cross-coupling with a reduction step.

**Scheme 2:**
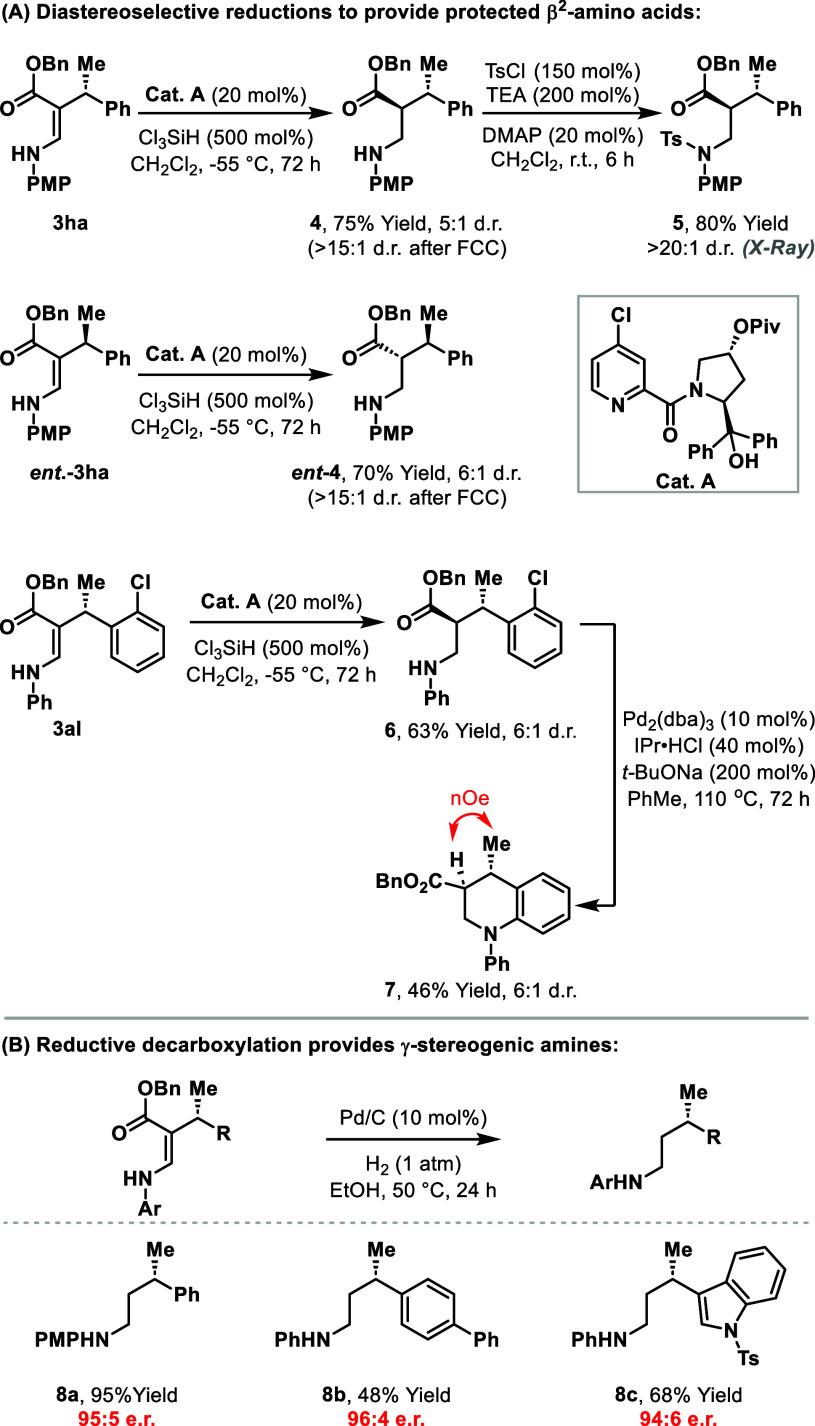
Derivatizations of the Cross-Coupling Products

A series of control experiments have provided insight into the mechanism of Ir-catalyzed hydroalkenylative cross-coupling (Scheme 3A). Most significantly, we have confirmed that efficient cross-coupling requires an NHAr unit. In support of this, O-Bn acrylate **9** and enamine **1m**, which does not have an NH, were both unsuitable reaction partners (i.e., **10** and **3ma** were not observed). BnNH- and AcNH-based systems **1n** and **1o** were also evaluated; the former did participate, providing **3na** in 12% yield, whereas no product (**3oa**) was observed with the latter. Overall, these results highlight the importance of having an appropriately nucleophilic/coordinating NH-unit, with the optimal balance being that of aniline-derived systems. Exposure of **1a** (R = H) or **1m** (R = Me) to optimized conditions, but in the absence of alkene and in the presence of D_2_O, resulted in substantial deuterium exchange at C3–H, which, in both cases, was higher than in the absence of the Ir-catalyst (Scheme 3B). The origins of the Ir-enhancement of the H/D exchange process are unclear,^22^ but these results do indicate that both systems are similarly nucleophilic through C3. Thus, the differing reactivity of **1a** vs **1m** in the cross-coupling protocol appears to derive solely from the presence or absence of an NH unit. ArNH-directing groups underpinned our earlier work on Ir-catalyzed C–H alkylations of glycine derivatives^8a^ and Rh-catalyzed carbonylative heterocyclizations of cyclopropanes.^10^ In both cases, N–H metalation was proposed as the key substrate binding event, and we favor this as the initiating step for the current processes. Thus, a viable pathway involves NH-metalation of **1a** to provide **Int-I**,^23^ which then coordinates the alkene to form **Int-II**. Inner sphere and enantiodetermining carbometalation provides **Int-III**, which undergoes protodemetalation and tautomerization to deliver the targets.^8b^ The branched selectivity arises upon conversion of **Int-II** to **Int-III** and may reflect either a preference for the bulky Ir-center to move to the less hindered end of the alkene, or electronic effects.^24^ An outer sphere carbometalation pathway, involving attack of **Int-I** onto an Ir-π-complex, is disfavored because we observe first order kinetics with respect to the catalyst (see the Supporting Information). An alternative pathway involving N-directed C–H activation from **Int-I** cannot be discounted, although we disfavor this because of the high strain of the aza-iridacyclobutene that would arise^25^ and because the conversion of **Int-II** to **Int-III** is consistent with the Ir-enolate-based reactivity we have previously observed.^8b^

**Scheme 3:**
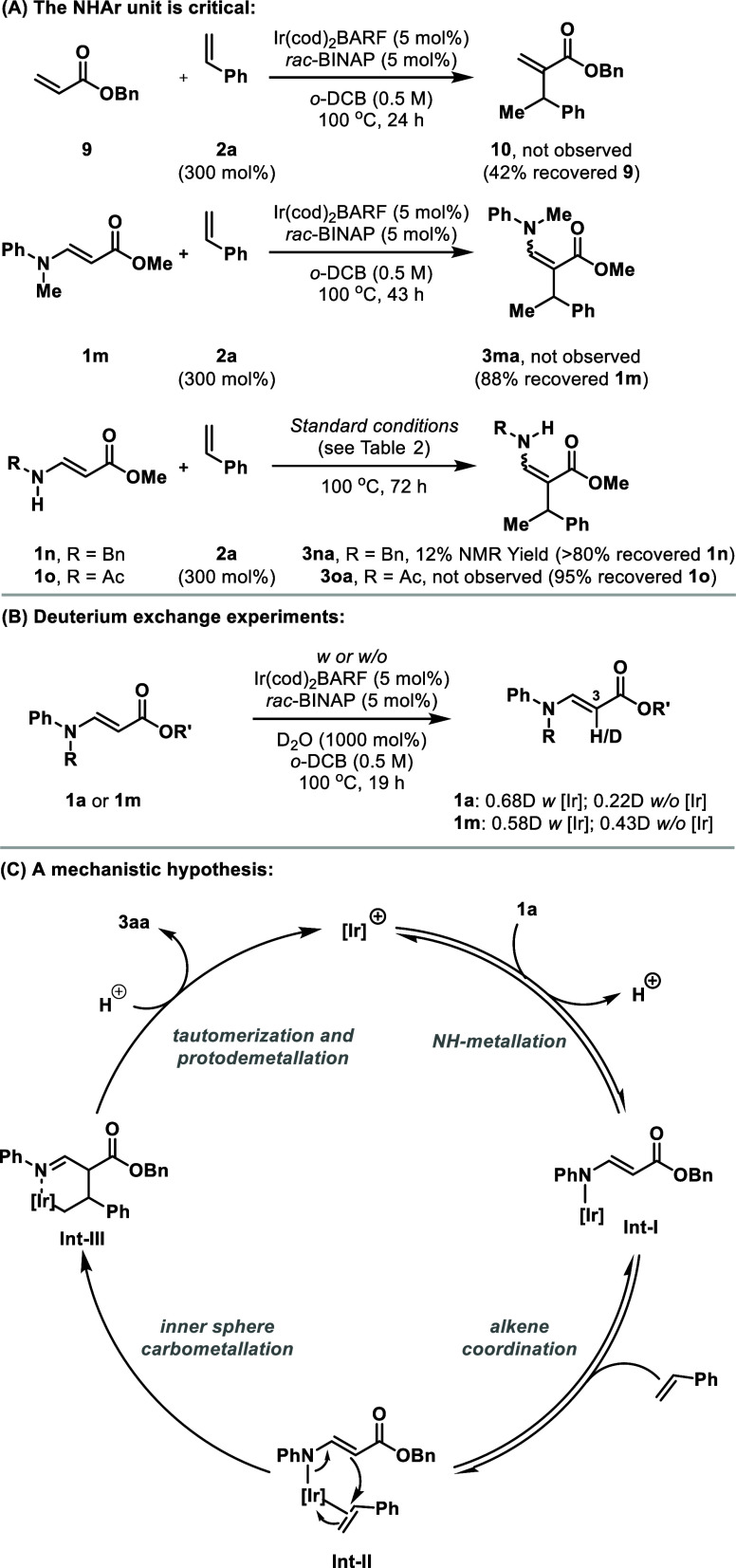
Mechanistic Studies

In summary, we outline a strategy where NH-metalation of persistent enamines generates Ir-aza-enolates that engage styrenes or α-olefins in branched and enantioselective C–H addition reactions. This unique C(sp^2^)–C(sp^3^) cross-coupling adds to an emerging range of processes that harness the untapped potential of Ir-enolate-like species for the design of enantioselective and byproduct free C–H addition reactions.^8^ The processes described here are mechanistically distinct from Li’s C–H activation-driven alkene hydroalkenylation reactions,^7c^ and this, in turn, offers distinct opportunities for reaction design. Indeed, whereas Li’s protocol requires α-olefins and terminal aryl-methyl-ketone-derived enamides, the present method harnesses internal enamines and encompasses both styrenes and α-olefins as the coupling partner. This complementarity is exploited to provide products that enable a reductive entry to challenging β^2^-amino acids. Beyond this, the broad occurrence of NH enamines, and particularly as intermediates in organocatalysis, suggests that the strategy described here might enable the design of tandem catalytic cross-couplings.^26^

## Supporting Information Available

Experimental details, characterization data, and crystallographic data (PDF)
