# Supplementary material for: An Aza-Enolate Strategy Enables Iridium-Catalyzed Enantioselective Hydroalkenylations of Minimally Polarized Alkenes en Route to Complex N-Aryl β2-Amino Acids
Source: J Am Chem Soc. 2024 Aug 6;146(33):22923–9. doi: 10.1021/jacs.4c07519 (PMC11345758; doi:10.1021/jacs.4c07519)
Supplement: Supplementary file 1 — ja4c07519_si_001.pdf [file ja4c07519_si_001.pdf]

An Aza-Enolate Strategy Enables Iridium-Catalyzed Enantioselective Hydroalkenylations of Minimally Polarized Alkenes En Route to Complex N-Aryl β^2-Amino Acids

Fenglin Hong,† Craig M. Robertson,† and John F. Bower†*

† Department of Chemistry, University of Liverpool, Crown Street, Liverpool, L69 7ZD, United Kingdom

Supporting Information

Table of Contents

General experimental details 2
Experimental procedures and data 3
Optimization of reaction conditions 3
Substrate synthesis 4
Reaction scope 11
Figure S1 56
Applications and derivatizations 57
Evaluation of other protecting groups 67
Mechanistic studies 68
Scheme S1: Control experiments 68
Scheme S2: Deuterium exchange and labelling experiments 69
Figure S2: Kinetic experiment 71
References 72
NMR Spectra 74

General experimental details

All reagents requiring purification were purified using standard laboratory techniques according to methods published by Armarego, and Perrin (Pergamon Press, 1966). Catalytic reactions were carried out in Young-type re-sealable tubes. Styrene and other commercially available alkenes (liquid) were quickly distilled using a Hickman distilling head before use. All other commercially available alkenes (solid) were used as received without any further purification. Iridium catalysts were synthesized according to previously reported procedures[1,2]. Anhydrous THF, PhMe, and CH_2Cl_2 (DCM) were obtained by either passed through drying columns supplied by Anhydrous Engineering Ltd or purchased from commercial sources (Acros or Aldrich). Anhydrous 1,2-dichlorobenzene (*o*-DCB), 1,4-dioxane, PhCl, MeOH and EtOH were purchased as anhydrous grade and stored over activated 4Å molecular sieves prior to use. All reactions were performed using dry solvents unless stated otherwise. Triethylamine (TEA) was distilled over CaH_2 and stored over activated 4Å molecular sieves under nitrogen. Flash column chromatography (FCC) was performed using silica gel (Aldrich 40-63 µm, 230-400 mesh). Thin layer chromatography was performed using aluminium backed 60 F_{254} silica plates. Visualisation was achieved by UV fluorescence or a basic $KMnO_4$ solution and heat. Proton nuclear magnetic resonance spectra (NMR) were recorded at 400 MHz or 500 MHz as stated. ^{13}C NMR spectra were recorded at 125 MHz as stated. Chemical shifts (δ) are given in parts per million (ppm). Peaks are described as singlets (s), doublets (d), triplets (t), quartets (q), septets (sept), multiplets (m) and broad (br.). Coupling constants (*J*) are quoted to the nearest 0.5 Hz. When compounds were isolated as a mixture of diastereoisomers, they are referred to as a (major) and b (minor). *In situ* yields were determined by employing 1,3,5-trimethoxybenzene as the internal standard. High resolution mass spectra were determined by the University of Liverpool mass spectrometry service, given to four decimal places. Mass spectra were recorded on Agilent 7200 Accurate Mass QTOF GC/MS (under condition of chemical ionization-CI) and Agilent 6540 UHD Accurate Mass Q-TOF LC/MS (under condition of electrospray ionization-ESI). Infrared spectra were recorded on a Perkin Elmer Spectrum Two FTIR spectrometer as thin films or solids compressed on a diamond plate. Melting points were determined using Reichert melting point apparatus and are uncorrected. Optical rotations were measured using an ADP440^{+} polarimeter at the concentration and temperature stated. Enantiomeric excesses were determined using an Agilent 1290 Infinity chiral SFC as stated for each compound. A diode array detector was used and chromatograms are taken from a wavelength of 254 nm.

Experimental procedures and data

Optimization of reaction conditions

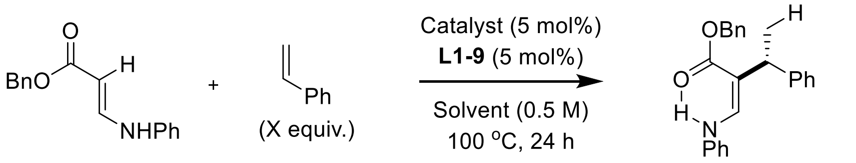

Entry	Precatalyst	Ligand	Solvent	*X*	Yield (%)[a]	E.r.[b]	*E*/*Z*[c]
1	Ir(cod)$_2$BARF	**L1**	*o*-DCB	3	90	93:7	12:1
2	Ir(cod)$_2$BARF	**L2**	*o*-DCB	3	91	94:6	15:1
3	Ir(cod)$_2$BARF	**L3**	*o*-DCB	3	71	93.5:6.5	11:1
4	Ir(cod)$_2$BARF	**L4**	*o*-DCB	3	94	95:5	>20:1
5	Ir(cod)$_2$BARF	**L5**	*o*-DCB	3	33	94:6	7:1
6	Ir(cod)$_2$BARF	**L6**	*o*-DCB	3	<10	-	-
**7**	**Ir(cod)$_2$BARF**	**L7**	***o*-DCB**	**3**	**96 (90)[d]**	**96:4**	**>20:1**
8	Ir(cod)$_2$BARF	**L8**	*o*-DCB	3	89	95:5	11:1
9	Ir(cod)$_2$BARF	**L9**	*o*-DCB	3	65	90:10	12:1
10	Ir(cod)$_2$BARF	**L7**	PhCl	3	90	95:5	>20:1
11	Ir(cod)$_2$BF$_4$	**L7**	1,4-dioxane	3	80	96:4	18:1
12	Ir(cod)$_2$OTf	**L7**	*o*-DCB	3	<10	-	-
13	[Ir(cod)Cl]$_2$[e]	**L7**	*o*-DCB	3	<10	-	-
14	Rh(cod)$_2$BARF	**L7**	*o*-DCB	3	<10	-	-
15	Ir(cod)$_2$BARF	**L7**	*o*-DCB	2	90	95.5:4.5	>20:1
16[f]	Ir(cod)$_2$BARF	**L7**	*o*-DCB	3	45	95:5	>20:1

Reaction conditions: **1a** (0.1 mmol), catalyst (5.00 μmol), **L** (5.00 μmol), styrene (0.2 or 0.3 mmol), solvent (0.2 mL), 100 °C, 24 h, in Schlenk tubes. [a]Measured by ^{1}H NMR using 1,3,5-trimethoxybenzene as the internal standard. [b]Determined by chiral SFC analysis. [c]Determined by ^{1}H NMR analysis of the reaction mixture. [d]Isolated yield. [e]Catalyst (0.0025 mmol). [f]The reaction was performed at 90 °C. *o*-DCB = 1,2-dichlorobenzene.

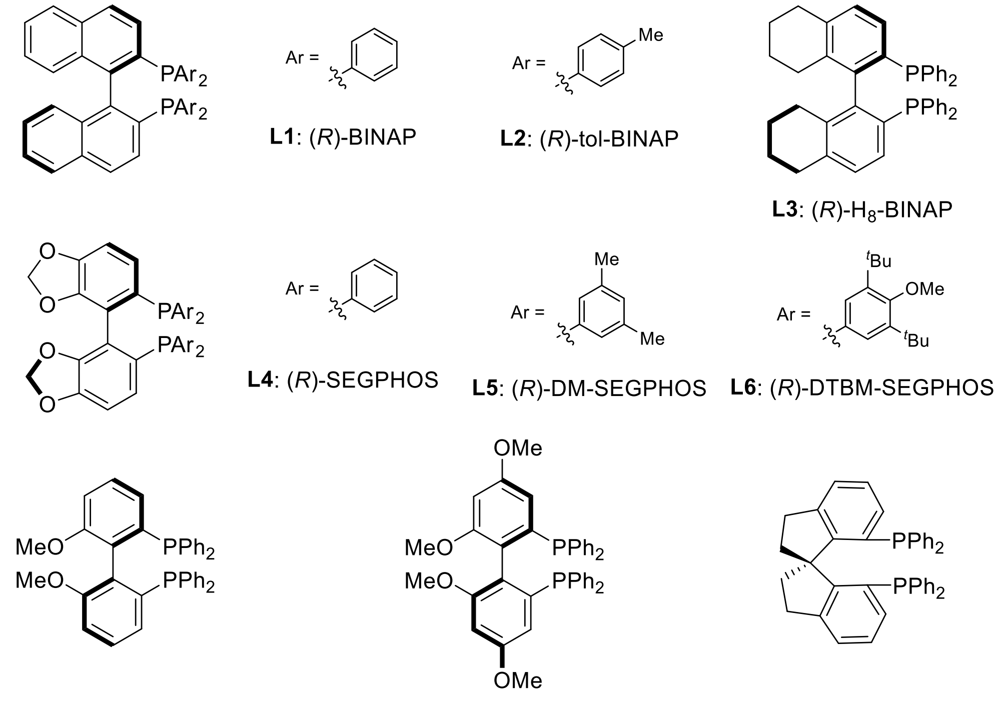

L1: (*R*)-BINAP **L2**: (*R*)-tol-BINAP **L3**: (*R*)-H$_8$-BINAP

L4: (*R*)-SEGPHOS **L5**: (*R*)-DM-SEGPHOS **L6**: (*R*)-DTBM-SEGPHOS

L7: (*R*)-MeO-BIPHEP **L8**: (*R*)-Ph-Garphos **L9**: (*R*)-SDP

Substrate synthesis

Acrylates **1a-l** were synthesized according to the modified procedures.[3]

General procedure A for the synthesis of acrylates **1a-l:**

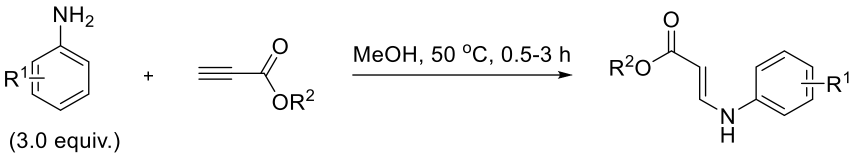

An oven-dried round bottom flask was charged with aniline (3.00 mmol, 300 mol%), and MeOH (5.0 mL). Propiolate (1.00 mmol, 100 mol%) was then added dropwise over 1 minute at room temperature (r.t.). After the addition was complete, the reaction mixture was stirred for 0.5-3 hour(s) at 50 °C and the progress of the reaction was monitored by TLC. Upon completion, the reaction mixture was filtered and concentrated *in vacuo*. The residue was purified by FCC under the conditions noted.

Benzyl (*E*)-3-(phenylamino)acrylate (1a):

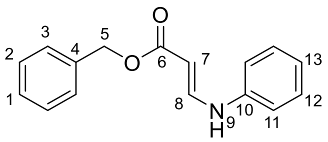

General procedure A: Purification by column chromatography (hexane/EtOAc, 95:5) afforded the title compound (121 mg, 48%) as a colorless solid. **m.p.** = 66 – 68 °C (hexane/EtOAc); **IR (thin film)** ν_{max}/cm^{-1}: 3293 (br), 1665 (s), 1623 (s), 1190 (s), 741 (s), 696 (s); **^{1}H NMR** (500 MHz, $CDCl_3$) δ 9.92 (d, *J* = 13.0 Hz, 1H, H^9), 7.44 – 7.28 (m, 8H, H^1 + H^2 + H^3 + H^8 + H^{12}), 7.04 (t, *J* = 7.5 Hz, 1H, H^{13}), 7.00 (d, *J* = 8.0 Hz, 2H, H^{11}), 5.22 (s, 2H, H^5), 4.95 (d, *J* = 8.5 Hz, 1H, H^7); **^{13}C NMR** (126 MHz, $CDCl_3$) δ 170.0 (C=O), 143.3 (C^8), 140.6 (C^{10}), 136.7 (C^4), 129.6 (C^{12}), 128.5 (C^2), 128.0 (C^1), 127.9 (C^3), 122.7 (C^{13}), 115.4 (C^{11}), 87.0 (C^7), 65.1 (C^5); **HRMS** (ESl): calculated for $C_{16}H_{16}NO_2$ $[M+H]^+$ requires *m/z* 254.1176, found *m/z* 254.1180.

Methyl (*E*)-3-(phenylamino)acrylate (1b):

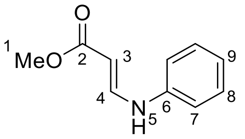

General procedure A: Purification by column chromatography (hexane/EtOAc, 95:5) afforded the title compound (97.4 mg, 55%) as a pale-yellow oil. **^{1}H NMR** (500 MHz, $CDCl_3$)

δ 9.91 (d, J = 12.0 Hz, 1H, H^5), 7.34 – 7.26 (m, 3H, H^4 + H^8), 7.03 (t, J = 7.5 Hz, 1H, H^9), 6.99 (d, J = 7.5 Hz, 2H, H^7), 4.88 (d, J = 8.5 Hz, 1H, H^3), 3.75 (s, 3H, H^1).

The spectroscopic properties were consistent with the data available in the literature.[4]

Ethyl (*E*)-3-(phenylamino)acrylate (1c):

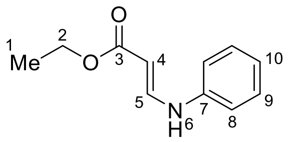

General procedure A: Purification by column chromatography (hexane/EtOAc, 95:5) afforded the title compound (111 mg, 58%) as a pale-yellow oil. **^{1}H NMR** (500 MHz, $CDCl_3$) δ 9.93 (d, J = 13.0 Hz, 1H, H^6), 7.34 – 7.25 (m, 3H, H^5 + H^9), 7.02 (t, J = 7.5 Hz, 1H, H^{10}), 6.99 (d, J = 8.0 Hz, 2H, H^8), 4.87 (d, J = 8.5 Hz, 1H, H^4), 4.21 (q, J = 7.0 Hz, 2H, H^2), 1.33 (t, J = 7.0 Hz, 3H, H^1).

The spectroscopic properties were consistent with the data available in the literature.[5]

Isopropyl (*E*)-3-(phenylamino)acrylate (1d):

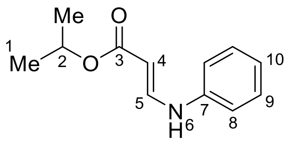

General procedure A: Purification by column chromatography (hexane/EtOAc, 95:5) afforded the title compound (90.2 mg, 44%) as a pale-yellow oil. **IR (thin film)** ν_{max}/cm^{-1}: 3310 (br), 2979 (s), 1601 (s), 1597 (s), 1104 (s), 751 (s); **^{1}H NMR** (500 MHz, $CDCl_3$) δ 9.95 (d, J = 13.0 Hz, 1H, H^6), 7.33 – 7.24 (m, 3H, H^5 + H^9), 7.01 (t, J = 7.5 Hz, 1H, H^{10}), 6.98 (d, J = 7.5 Hz, 2H, H^8), 5.12 – 5.07 (m, 1H, H^2), 4.84 (d, J = 8.5 Hz, 1H, H^4), 1.31 (d, J = 6.5 Hz, 6H, H^1); **^{13}C NMR** (126 MHz, $CDCl_3$) δ 170.0 (C=O), 142.8 (C^5), 140.8 (C^7), 129.7 (C^9), 122.5 (C^{10}), 115.3 (C^8), 88.0 (C^4), 66.4 (C^2), 22.1 (C^1); **HRMS**: a stable ion was not found in ESI and CI.

(*E*)-1-Phenyl-3-(phenylamino)prop-2-en-1-one (1e):

Acrylate **1e** was synthesized according to the reported procedures.[6]

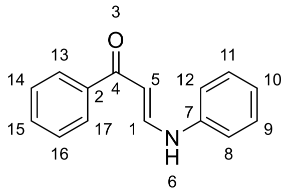

^{1}H NMR (500 MHz, $CDCl_3$) δ 12.18 (d, J = 12.5 Hz, 1H, H^{6}), 7.79 (d, J = 7.0 Hz, 2H, H^{13} + H^{17}), 7.57 – 7.47 (m, 4H, ArH), 7.39 – 7.36 (m, 2H, ArH), 7.15 – 7.10 (m, 3H, ArH), 6.06 (d, J = 8.0 Hz, 1H, H^{5}).

The spectroscopic properties were consistent with the data available in the literature. [6]

(*E*)-3-(Phenylamino)-1-(pyrrolidin-1-yl)prop-2-en-1-one (1f):

Acrylate **1f** was synthesized according to the modified procedures. [4]

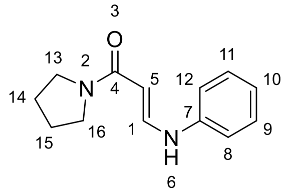

An oven-dried round bottom flask was charged with 1-(pyrrolidin-1-yl)prop-2-yn-1-one (246 mg, 2.00 mmol, 100 mol%) and CuI (19.1 mg, 0.10 mmol, 5 mol%). The flask was evacuated and refilled with N_2 (three cycles), then aniline (0.18 mL, 2.00 mmol, 100 mol%) and dry toluene (1.0 mL) were added. After the addition was complete, the reaction mixture was stirred for 9 hours at 70 °C and the progress of the reaction was monitored by TLC. Upon completion, the reaction mixture was concentrated *in vacuo*. The residue was purified by column chromatography (hexane/EtOAc, 80:20) afforded the title compound (207 mg, 48%) as a pale-yellow solid. **m.p.** = 106 – 108 °C (hexane/EtOAc); **IR (thin film)** ν_{max}/cm^{-1}: 3207 (br), 2867 (s), 1637 (s), 1455 (s), 1384 (s), 1280 (s), 755 (s), 689 (s); **^{1}H NMR** (500 MHz, $CDCl_3$) δ 10.93 (d, J = 12.0 Hz, 1H, H^{6}), 7.30 – 7.27 (m, 2H, H^{9} + H^{11}), 7.19 (dd, J = 12.0, 8.5 Hz, 1H, H^{1}), 6.97 – 6.94 (m, 3H, H^{8} + H^{10} + H^{12}), 4.90 (d, J = 8.5 Hz, 1H, H^{5}), 3.55 (t, J = 7.0 Hz, 2H, H^{13}), 3.46 (t, J = 7.0 Hz, 2H, H^{16}), 2.02 – 1.97 (m, 2H, H^{14}), 1.93 – 1.88 (m, 2H, H^{15}); **^{13}C NMR** (126 MHz, $CDCl_3$) δ 168.9 (C=O), 141.4 (C^{1}), 140.3 (C^{7}), 129.5 (C^{9} + C^{11}), 121.6 (C^{10}), 114.9 (C^{8} + C^{12}), 88.3 (C^{5}), 46.4 (C^{13}), 45.1 (C^{16}), 26.1 (C^{14}), 24.6 (C^{15}); **HRMS** (ESl): calculated for $C_{13}H_{17}N_2O$ $[M+H]^+$ requires *m/z* 217.1335, found *m/z* 217.1341.

Benzyl (*E*)-3-(*p*-tolylamino)acrylate (1g):

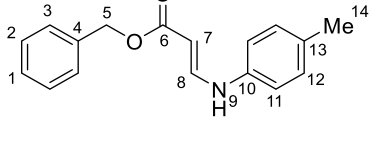

General procedure A: Purification by column chromatography (hexane/EtOAc, 95:5) afforded the title compound (139 mg, 52%) as a colorless solid. **m.p.** = 74 – 76 °C (hexane/EtOAc); **IR (thin film)** ν_{max}/cm^{-1}: 3329 (br), 1668 (s), 1617 (s), 1178 (s), 812 (s); **^{1}H**

NMR (500 MHz, $CDCl_3$) δ 9.86 (d, *J* = 13.0 Hz, 1H, H^9), 7.44 – 7.33 (m, 5H, $H^1 + H^2 + H^3$), 7.29 – 7.25 (m, 1H, H^8), 7.13 (d, *J* = 8.5 Hz, 2H, H^{12}), 6.90 (d, *J* = 8.5 Hz, 2H, H^{11}), 5.21 (s, 2H, H^5), 4.90 (d, *J* = 8.0 Hz, 1H, H^7), 2.33 (s, 3H, H^{14}); **^{13}C NMR** (126 MHz, $CDCl_3$) δ 170.1 (C=O), 143.9 (C^8), 138.3 (C^{10}), 136.8 (C^4), 132.3 (C^{13}), 130.2 (C^{12}), 128.6 (C^2), 127.99 (C^1), 127.96 (C^3), 115.5 (C^{11}), 86.3 (C^7), 65.1 (C^5), 20.7 (C^{14}); **HRMS** (ESI): calculated for $C_{17}H_{18}NO_2$ $[M+H]^+$ requires *m/z* 268.1332, found *m/z* 268.1337.

Benzyl (*E*)-3-((4-methoxyphenyl)amino)acrylate (1h):

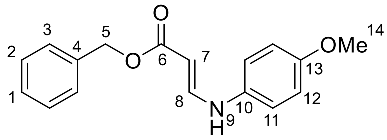

General procedure A: Purification by column chromatography (hexane/EtOAc, 90:10) afforded the title compound (130 mg, 46%) as a colorless solid. **m.p.** = 70 – 72 °C (hexane/EtOAc); **IR (thin film)** ν_{max}/cm^{-1}: 3272 (br), 1668 (s), 1617 (s), 1482 (s), 1185 (s), 780 (s); **^{1}H NMR** (500 MHz, $CDCl_3$) δ 9.85 (d, *J* = 13.0 Hz, 1H, H^9), 7.45 – 7.34 (m, 5H, $H^1 + H^2 + H^3$), 7.23 – 7.19 (m, 1H, H^8), 6.95 – 6.88 (m, 4H, $H^{11} + H^{12}$), 5.22 (s, 2H, H^5), 4.89 (d, *J* = 8.0 Hz, 1H, H^7), 3.81 (s, 3H, H^{14}); **^{13}C NMR** (126 MHz, $CDCl_3$) δ 170.2 (C=O), 155.7 (C^{13}), 144.6 (C^8), 136.9 (C^4), 134.4 (C^{10}), 128.6 (C^2), 127.99 (C^1), 127.96 (C^3), 117.1 (C^{11}), 115.0 (C^{12}), 85.8 (C^7), 65.1 (C^5), 55.6 (C^{14}); **HRMS** (ESI): calculated for $C_{17}H_{18}NO_3$ $[M+H]^+$ requires *m/z* 284.1281, found *m/z* 284.1284.

Benzyl (*E*)-3-((4-hydroxyphenyl)amino)acrylate (1i):

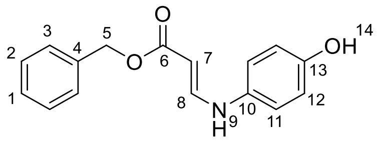

General procedure A: Purification by column chromatography (hexane/EtOAc, 90:10) afforded the title compound (113 mg, 42%) as a colorless solid. **m.p.** = 125 – 127 °C (hexane/EtOAc); **IR (thin film)** ν_{max}/cm^{-1}: 3269 (br), 1652 (s), 1442 (s), 1191 (s), 779 (s); **^{1}H NMR** (500 MHz, CD_3OD) δ 9.72 (d, *J* = 13.0 Hz, 1H, H^9), 7.43 – 7.20 (m, 6H, $H^1 + H^2 + H^3 + H^8$), 6.89 (d, *J* = 8.5 Hz, 2H, H^{11}), 6.75 (d, *J* = 8.5 Hz, 2H, H^{12}), 5.12 (s, 2H, H^5), 4.76 (d, *J* = 8.0 Hz, 1H, H^7); **^{13}C NMR** (126 MHz, CD_3OD) δ 171.5 (C=O), 154.5 (C^{13}), 146.2 (C^8), 138.5 (C^4), 134.7 (C^{10}), 129.4 (C^2), 128.91 (C^3), 128.86 (C^1), 118.1 (C^{11}), 117.2 (C^{12}), 85.6 (C^7), 65.8 (C^5); **HRMS** (ESI): calculated for $C_{16}H_{16}NO_3$ $[M+H]^+$ requires *m/z* 270.1125, found *m/z* 270.1129.

Benzyl (*E*)-3-((4-fluorophenyl)amino)acrylate (1j):

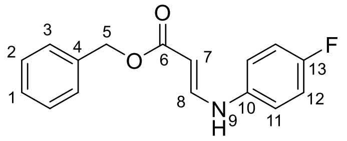

General procedure A: Purification by column chromatography (hexane/EtOAc, 95:5) afforded the title compound (152 mg, 56%) as a colorless solid. **m.p.** = 69 – 71 °C (hexane/EtOAc); **IR (thin film)** ν_{max}/cm^{-1}: 3318 (br), 1667 (s), 1480 (s), 1120 (s), 824 (s); **^{1}H NMR** (500 MHz, $CDCl_3$) δ 9.89 (d, *J* = 13.5 Hz, 1H, H^9), 7.57 – 7.33 (m, 5H, H^1 + H^2 + H^3), 7.22 – 7.18 (m, 1H, H^8), 7.05 – 6.92 (m, 4H, H^{11} + H^{12}), 5.21 (s, 2H, H^5), 4.93 (d, *J* = 8.5 Hz, 1H, H^7); **^{13}C NMR** (126 MHz, $CDCl_3$) δ 170.1 (C=O), 158.7 (d, *J* = 242.0 Hz, C^{13}), 143.9 (C^8), 137.0 (d, *J* = 2.5 Hz, C^{10}), 136.8 (C^4), 128.6 (C^2), 128.1 (C^1), 128.0 (C^3), 116.9 (d, *J* = 7.5 Hz, C^{11}), 116.4 (d, *J* = 22.5 Hz, C^{12}), 87.0 (C^7), 65.2 (C^5); ^{19}F NMR (471 MHz, $CDCl_3$) δ -120.8; **HRMS** (ESl): calculated for $C_{16}H_{15}FNO_2$ $[M+H]^+$ requires *m/z* 272.1081, found *m/z* 272.1083.

Benzyl (*E*)-3-((4-chlorophenyl)amino)acrylate (1k):

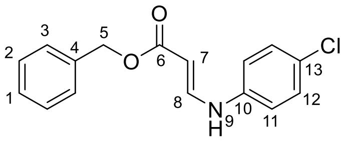

General procedure A: Purification by column chromatography (hexane/EtOAc, 90:10) afforded the title compound (172 mg, 60%) as a colorless solid. **m.p.** = 87 – 89 °C (hexane/EtOAc); **IR (thin film)** ν_{max}/cm^{-1}: 3322 (br), 1622 (s), 1475 (s), 1192 (s), 790 (s); **^{1}H NMR** (500 MHz, $CDCl_3$) δ 9.92 (d, *J* = 11.0 Hz, 1H, H^9), 7.42 – 7.20 (m, 8H, H^1 + H^2 + H^3 + H^8 + H^{12}), 6.91 (d, *J* = 8.5 Hz, 2H, H^{11}), 5.21 (s, 2H, H^5), 4.97 (d, *J* = 8.5 Hz, 1H, H^7); **^{13}C NMR** (126 MHz, $CDCl_3$) δ 170.0 (C=O), 143.1 (C^8), 139.3 (C^{10}), 136.6 (C^4), 129.7 (C^{12}), 128.6 (C^2), 128.1 (C^1), 128.0 (C^3), 127.6 (C^{13}), 116.6 (C^{11}), 87.9 (C^7), 65.3 (C^5); **HRMS** (ESl): calculated for $C_{16}H_{15}{}^{35}ClNO_2$ $[M+H]^+$ requires *m/z* 288.0786, found *m/z* 288.0791.

4-Bromobenzyl (*E*)-3-((4-methoxyphenyl)amino)acrylate (1l):

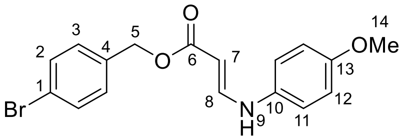

General procedure A: Purification by column chromatography (hexane/EtOAc, 90:10) afforded the title compound (108 mg, 30%) as a colorless solid. **m.p.** = 110 – 113 °C (hexane/EtOAc); **IR (thin film)** ν_{max}/cm^{-1}: 3332 (br), 1615 (s), 1481 (s), 1200 (s), 779 (s); **^{1}H**

NMR (500 MHz, $CDCl_3$) δ 9.80 (d, *J* = 13.0 Hz, 1H, H^9), 7.51 (d, *J* = 8.5 Hz, 2H, H^2), 7.29 (d, *J* = 8.5 Hz, 2H, H^3), 7.20 (dd, *J* = 13.0, 8.0 Hz, 1H, H^8), 6.94 (d, *J* = 9.0 Hz, 2H, ArH), 6.88 (d, *J* = 9.0 Hz, 2H, ArH), 5.14 (s, 2H, H^5), 4.85 (d, *J* = 8.0 Hz, 1H, H^7), 3.80 (s, 3H, H^{14}); **^{13}C NMR** (126 MHz, $CDCl_3$) δ 170.2 (C=O), 155.7 (C^{13}), 144.9 (C^8), 136.0 (C^4), 134.3 (C^{10}), 131.7 (C^2), 129.6 (C^3), 121.9 (C^1), 117.2 (C^{11}), 115.0 (C^{12}), 85.4 (C^7), 64.2 (C^5), 55.6 (C^{14}); **HRMS** (ESl): calculated for $C_{17}H_{17}{}^{79}BrNO_3$ $[M+H]^+$ requires *m/z* 362.0386, found *m/z* 362.0383.

Methyl (*E*)-3-(methyl(phenyl)amino)acrylate (1m):

Acrylate **1b** was synthesized according to the reported procedures.[7]

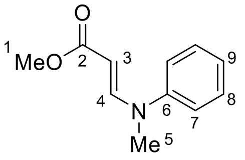

^{1}H NMR (500 MHz, $CDCl_3$) δ 7.95 (d, *J* = 13.0 Hz, 1H, H^4), 7.36 – 7.33 (m, 2H, H^8), 7.14 – 7.12 (m, 3H, H^7 + H^9), 4.95 (d, *J* = 13.0 Hz, 1H, H^3), 3.71 (s, 3H, H^1), 3.23 (s, 3H, H^5).

The spectroscopic properties were consistent with the data available in the literature.[7]

Methyl (*E*)-3-(benzylamino)acrylate (1n):

Acrylate **1n** was synthesized according to the reported procedure.[8]

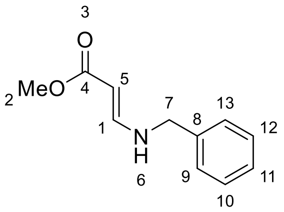

^{1}H NMR (500 MHz, $CDCl_3$) δ 8.15 (br. s, 1H, H^6), 7.38 – 7.27 (m, 5H, $H^{9\text{-}13}$), 6.72 (dd, *J* = 13.0, 8.0 Hz, 1H, H^1), 4.58 (d, *J* = 8.0 Hz, 1H, H^5), 4.38 (d, *J* = 6.0 Hz, 2H, H^7), 3.67 (s, 3H, H^2). (*E/Z* = 3.6:1)

The spectroscopic properties were consistent with the data available in the literature.[8]

Methyl (*E*)-3-acetamidoacrylate (1o):

Acrylate **1o** was synthesized according to the reported procedure.[9]

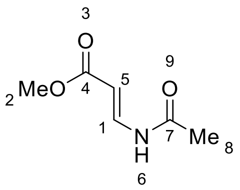

¹H NMR (500 MHz, $CDCl_3$) δ 10.43 (br s., 1H, H^6), 7.51 (dd, *J* = 11.5, 9.0 Hz, 1H, H^1), 5.16 (d, *J* = 9.0 Hz, 1H, H^5), 3.76 (s, 3H, H^2), 2.18 (s, 3H, H^8).

The spectroscopic properties were consistent with the data available in the literature.[9]

Alkenes:

Styrene **2a**, simple substituted styrenes **2b-d**, **2g-h**, **2k-n**, vinyl ferrocene **2q**, 2-vinylnaphthalene **2r** and α-olefins **2s-v** were purchased and used as received. Substituted styrenes **2e-f**, **2i-j**, **2o-p**, **2w** and **2aa** have previously been prepared in our lab.[10]

Alkenes 4-vinylbenzyl (*S*)-2-(6-methoxynaphthalen-2-yl)propanoate **2x**, (3*S*,8*S*,9*S*,10*R*,13*R*,14*S*,17*R*)-10,13-dimethyl-17-((*R*)-6-methylheptan-2-yl)-2,3,4,7,8,9,10,11,12,13,14,15,16,17-tetradecahydro-1*H*-cyclopenta[*a*]phenanthren-3-yl pent-4-enoate **2y** and (8*R*,9*S*,13*S*,14*S*)-13-methyl-3-vinyl-6,7,8,9,11,12,13,14,15,16-decahydro-17*H*-cyclopenta[*a*]phenanthren-17-one **2z** were prepared by reported procedures.[11-13]

4-Vinylbenzyl (*S*)-2-(6-methoxynaphthalen-2-yl)propanoate (2x):

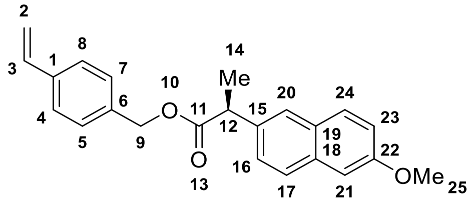

¹H NMR (500 MHz, $CDCl_3$) δ 7.73 – 7.66 (m, 3H, ArH), 7.41 (dd, *J* = 8.5, 2.0 Hz, 1H, ArH), 7.35 (d, *J* = 8.0 Hz, 2H, ArH), 7.22 (d, *J* = 8.0 Hz, 2H, ArH), 7.18 – 7.14 (m, 2H, ArH), 6.71 (dd, *J* = 17.5, 11.0 Hz, 1H, H^3), 5.75 (dd, *J* = 17.5, 1H, H^2), 5.27 (d, *J* = 11.0 Hz, 1H, H^2), 5.10 (q, *J* = 27.0, 12.5 Hz, 2H, H^9), 3.94 – 3.91 (m, 4H, H^{12} + H^{25}), 1.61 (d, *J* = 7.5 Hz, 3H, H^{14}).

The spectroscopic properties were consistent with the data available in the literature.[11]

4-(3*S*,8*S*,9*S*,10*R*,13*R*,14*S*,17*R*)-10,13-Dimethyl-17-((*R*)-6-methylheptan-2-yl)-2,3,4,7,8,9,10,11,12,13,14,15,16,17-tetradecahydro-1*H*-cyclopenta[*a*]phenanthren-3-yl pent-4-enoate (2y):

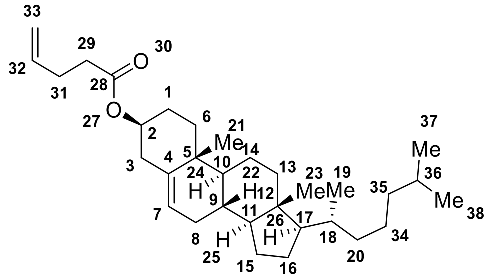

^{1}**H NMR** (500 MHz, $CDCl_3$) δ 5.89 – 5.80 (m, 1H, H^{32}), 5.40 – 5.39 (m, 1H, H^7), 5.10 – 5.06 (m, 1H, H^{33}), 5.03 – 5.01 (m, 1H, H^{33}), 4.68 – 4.61 (m, 1H, H^2), 2.40 – 2.39 (m, 4H), 2.34 – 2.32 (m, 2H), 2.05 – 1.96 (m, 2H), 1.90 – 1.81 (m, 3H), 1.64 – 1.45 (m, 7H), 1.42 – 1.24 (m, 4H), 1.22 – 1.08 (m, 7H), 1.04 (s, 3H), 1.03 – 0.95 (m, 2H), 0.94 – 0.93 (m, 3H), 0.90 – 0.88 (m, 6H, H^{37} + H^{38}), 0.70 (s, 3H).

The spectroscopic properties were consistent with the data available in the literature.[12]

1-(8*R*,9*S*,13*S*,14*S*)-13-Methyl-3-vinyl-6,7,8,9,11,12,13,14,15,16-decahydro-17*H*-cyclopenta[*a*]phenanthren-17-one (2z):

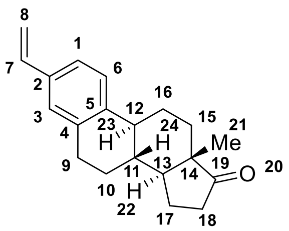

^{1}**H NMR** (500 MHz, $CDCl_3$) 7.29 (d, J = 8.0 Hz, 1H, ArH), 7.24 (d, J = 8.0 Hz, 1H, ArH), 7.17 (s, 1H, H^3), 6.69 (dd, J = 17.5, 11.0 Hz, 1H, H^7), 5.73 (d, J = 17.5 Hz, 1H, H^8), 5.22 (d, J = 11.0 Hz, 1H, H^8), 2.96 – 2.93 (m, 2H), 2.56 – 2.51 (m, 1H), 2.48 – 2.43 (m, 1H), 2.35 – 2.30 (m, 1H), 2.21 – 1.98 (m, 4H), 1.70 – 1.43 (m, 6H), 0.94 (s, 3H, H^{21}).

The spectroscopic properties were consistent with the data available in the literature.[13]

Reaction scope

General procedure B for the asymmetric α-alkylation of acrylate derivatives:

A Schlenk tube was charged with substrate (0.10 mmol, 100 mol%), [Ir(cod)$_2$]BARF (6.36 mg, 5.00 µmol, 5 mol%), (*R*)-OMe-BIPHEP (2.91 mg, 5.00 µmol, 5 mol%) and styrene derivative (if non-volatile, 300 mol%). The Schlenk tube was evacuated and refilled with N_2 (three cycles), then the alkene partner (if volatile, 300-500 mol%) was added followed by anhydrous *o*-DCB (0.2 mL, 0.5 M). The tube was sealed and heated at 100 °C for 24 hours. After cooling to r.t., the solvent was removed under reduced pressure and the crude reaction mixture was purified by FCC. The racemic products were also obtained using the above procedure (*rac*-BINAP was used in place of (*R*)-OMe-BIPHEP).

Benzyl (*R*,*Z*)-3-phenyl-2-((phenylamino)methylene)butanoate (3aa):

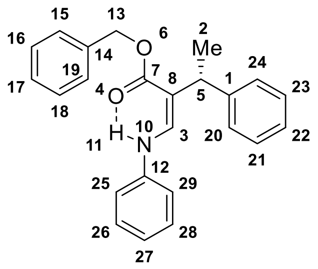

General procedure B: The reaction was carried out with substrate **1a** (25.3 mg, 0.10 mmol, 100 mol%) and styrene (34.0 µL, 0.30 mmol, 300 mol%). Purification of the residue by FCC (hexane/EtOAc 92:8) afforded the title compound (32.1 mg, 90%, >30:1 B:L, *Z/E* > 20:1, e.r. = 96:4) as a pale-yellow oil. ^{1}H NMR analysis of the crude material gave >30:1 B:L and *Z/E* > 20:1. $[\alpha]_D^{25}$ = -71.4 (c = 1.0, $CHCl_3$); **IR (thin film)** ν_{max}/cm^{-1}: 3330 (br), 2963 (s), 1670 (s), 1598 (s), 1201 (s), 697 (s); **^{1}H NMR** (500 MHz, $CDCl_3$) δ 10.08 (d, *J* = 12.5 Hz, 1H, H^{11}), 7.33 – 7.22 (m, 11H, H^{3} + ArH), 7.14 – 7.13 (m, 2H, ArH), 7.02 – 6.99 (m, 1H, H^{27}), 6.94 (d, *J* = 8.0 Hz, 2H, H^{25} + H^{29}), 5.19 – 5.12 (m, 2H, H^{13}), 4.11 (q, *J* = 7.0 Hz, 1H, H^{5}), 1.52 (d, *J* = 7.0 Hz, 3H, H^{2}); **^{13}C NMR** (126 MHz, $CDCl_3$) δ 169.5 (C^{7}), 146.7 (C^{1}), 141.2 (C^{12}), 141.1 (C^{3}), 136.6 (C^{14}), 129.7 (C^{26} + C^{28}), 128.4 (C^{16} + C^{18}), 128.3 (C^{21} + C^{23}), 127.8 (C^{17}), 127.5 (C^{15} + C^{19}), 127.4 (C^{20} + C^{24}), 125.9 (C^{22}), 122.1 (C^{27}), 115.3 (C^{25} + C^{29}), 104.3 (C^{8}), 65.4 (C^{13}), 38.6 (C^{5}), 21.4 (C^{2}); **HRMS** (ESl): calculated for $C_{24}H_{24}NO_2$ $[M+H]^+$ requires *m/z* 358.1802, found *m/z* 358.1805; **Chiral SFC:** DAICEL CHIRALCEL IE column (25 cm), CO_2:*i*-PrOH 95:5, 2.0 mL/min, 254 nm, 140 bar. Retention times: 17.6 mins (minor), 18.8 mins (major), e.r. = 96:4.

SFC analysis of the racemate, prepared using rac-BINAP:

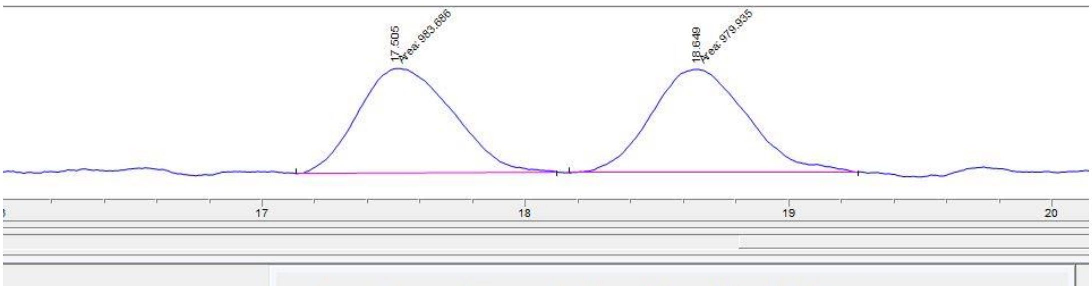

#	Time	Area	Height	Width	Area%	Symmetry
1	17.505	983.7	38.8	0.4228	50.095	0.673
2	18.649	979.9	38.2	0.4271	49.905	0.909

SFC analysis of the enantioenriched material, prepared using (R)-OMe-BIPHEP:

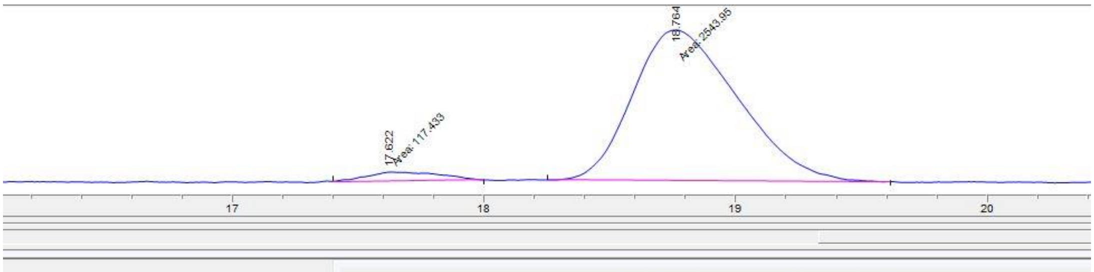

#	Time	Area	Height	Width	Area%	Symmetry
1	17.622	117.4	5.6	0.3487	4.412	0.489
2	18.764	2544	87.7	0.4835	95.588	0.699

Methyl (*R*,*Z*)-3-phenyl-2-((phenylamino)methylene)butanoate (3ba):

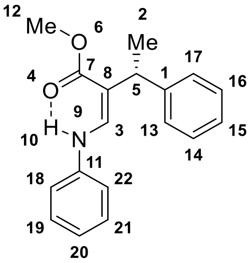

General procedure B: The reaction was carried out with substrate **1b** (17.7 mg, 0.10 mmol, 100 mol%) and styrene (34.0 µL, 0.30 mmol, 300 mol%). Purification of the residue by FCC (hexane/EtOAc 92:8) afforded the title compound (21.1 mg, 75%, >30:1 B:L, *Z/E* > 20:1, e.r. = 92:8) as a colorless solid. ^{1}H NMR analysis of the crude material gave >30:1 B:L and *Z/E* > 20:1. **m.p.** = 65 – 68 °C (hexane/EtOAc); $[\boldsymbol{\alpha}]_{\boldsymbol{D}}^{\mathbf{25}}$ = -58.4 (c = 1.0, $CHCl_3$); **IR (thin film)** ν_{max}/cm^{-1}: 3335 (br), 2946 (s), 1600 (s), 1201 (s), 694 (s); **^{1}H NMR** (500 MHz, $CDCl_3$) δ 10.06 (d, *J* = 12.5 Hz, 1H, H^{10}), 7.35 – 7.28 (m, 6H, ArH), 7.24 – 7.18 (m, 2H, H^{3} + ArH), 7.01 – 6.98 (m, 1H, H^{20}), 6.92 (d, *J* = 7.5 Hz, 2H, H^{18} + H^{22}), 4.08 (q, *J* = 7.0 Hz, 1H, H^{5}), 3.72 (s, 3H, H^{12}), 1.51 (d, *J* = 7.0 Hz, 3H, H^{2}); **^{13}C NMR** (126 MHz, $CDCl_3$) δ 170.3 (C^{7}), 146.4 (C^{1}), 141.3 (C^{11}), 141.0 (C^{3}), 129.7 (C^{19} + C^{21}), 128.2 (C^{14} + C^{16}), 127.4 (C^{13} + C^{17}), 125.9 (C^{15}), 122.0 (C^{20}), 115.2 (C^{18} + C^{22}), 104.7 (C^{8}), 50.9 (C^{12}), 38.4 (C^{5}), 21.4 (C^{2}); **HRMS** (ESI): calculated for $C_{18}H_{20}NO_2$ $[M+H]^+$ requires *m/z* 282.1489, found *m/z* 282.1487; **Chiral SFC:** DAICEL CHIRALCEL IE column (25 cm), CO_2:*i*-PrOH 95:5, 2.0 mL/min, 254 nm, 140 bar. Retention times: 6.2 mins (minor), 6.7 mins (major), e.r. = 92:8.

SFC analysis of the racemate, prepared using rac-BINAP:

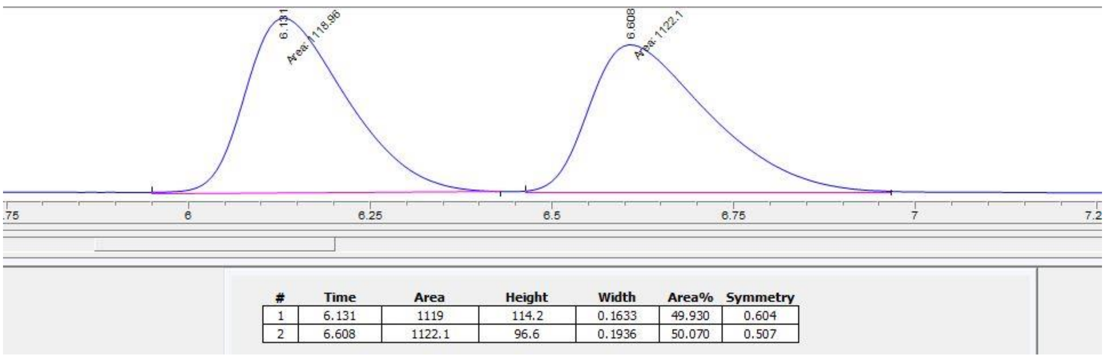

#	Time	Area	Height	Width	Area%	Symmetry
1	6.131	1119	114.2	0.1633	49.930	0.604
2	6.608	1122.1	96.6	0.1936	50.070	0.507

SFC analysis of the enantioenriched material, prepared using (R)-OMe-BIPHEP:

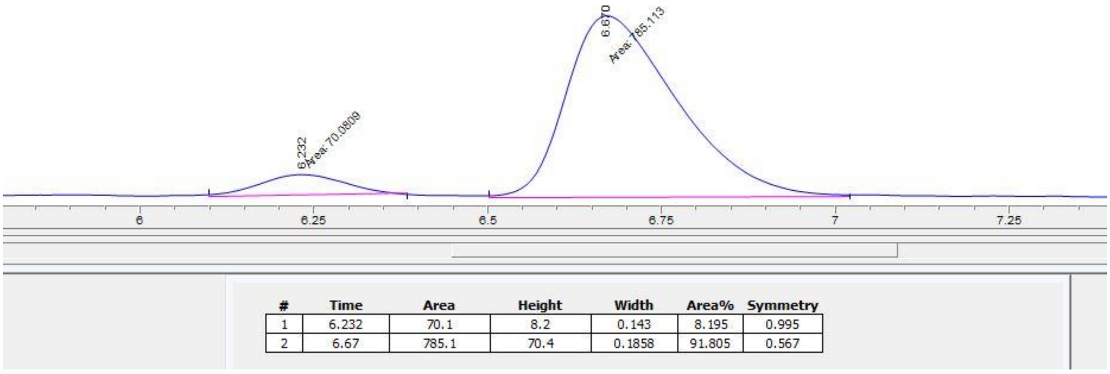

#	Time	Area	Height	Width	Area%	Symmetry
1	6.232	70.1	8.2	0.143	8.195	0.995
2	6.67	785.1	70.4	0.1858	91.805	0.567

Ethyl (*R*,*Z*)-3-phenyl-2-((phenylamino)methylene)butanoate (3ca):

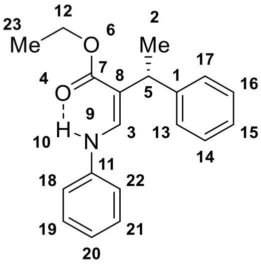

General procedure B: The reaction was carried out with substrate **1c** (19.1 mg, 0.10 mmol, 100 mol%) and styrene (34.0 µL, 0.30 mmol, 300 mol%). Purification of the residue by FCC (hexane/EtOAc 92:8) afforded the title compound (23.3 mg, 79%, >30:1 B:L, *Z/E* > 20:1, e.r. = 93:7) as a colorless solid. ^{1}H NMR analysis of the crude material gave >30:1 B:L and *Z/E* > 20:1. **m.p.** = 67 – 69 °C (hexane/EtOAc); $[\alpha]_D^{25}$ = -99.7 (c = 1.0, $CHCl_3$); **IR (thin film)** ν_{max}/cm^{-1}: 3283 (br), 2972 (s), 1598 (s), 1199 (s), 696 (s); **^{1}H NMR** (500 MHz, $CDCl_3$) δ 10.07 (d, *J* = 12.5 Hz, 1H, H^{10}), 7.35 – 7.28 (m, 6H, ArH), 7.23 – 7.19 (m, 2H, H^3 + ArH), 7.00 – 6.97 (m, 1H, H^{20}), 6.93 (d, *J* = 7.5 Hz, 2H, H^{18} + H^{22}), 4.18 – 4.12 (m, 2H, H^{12}), 4.06 (q, *J* = 7.0 Hz, 1H, H^5), 1.51 (d, *J* = 7.0 Hz, 3H, H^2), 1.21 (t, *J* = 7.0 Hz, 3H, H^{23}); **^{13}C NMR** (126 MHz, $CDCl_3$) δ 169.9 (C^7), 146.6 (C^1), 141.3 (C^{11}), 140.6 (C^3), 129.6 (C^{19} + C^{21}), 128.1 (C^{14} + C^{16}), 127.4 (C^{13} + C^{17}), 125.8 (C^{15}), 121.9 (C^{20}), 115.2 (C^{18} + C^{22}), 104.9 (C^8), 59.5 (C^{12}), 38.6

(C^5), 21.2 (C^2), 14.2 (C^{23}); **HRMS** (ESI): calculated for $C_{19}H_{22}NO_2$ $[M+H]^+$ requires *m/z* 296.1645, found *m/z* 296.1646; **Chiral SFC:** DAICEL CHIRALCEL IE column (25 cm), CO_2:*i*-PrOH 95:5, 2.0 mL/min, 254 nm, 140 bar. Retention times: 7.4 mins (minor), 7.9 mins (major), e.r. = 93:7.

SFC analysis of the racemate, prepared using rac-BINAP:

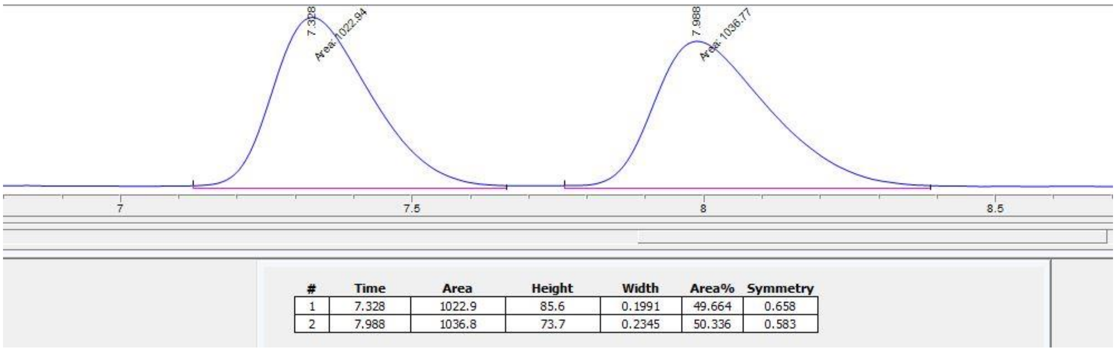

#	Time	Area	Height	Width	Area%	Symmetry
1	7.328	1022.9	85.6	0.1991	49.664	0.658
2	7.988	1036.8	73.7	0.2345	50.336	0.583

SFC analysis of the enantioenriched material, prepared using (R)-OMe-BIPHEP:

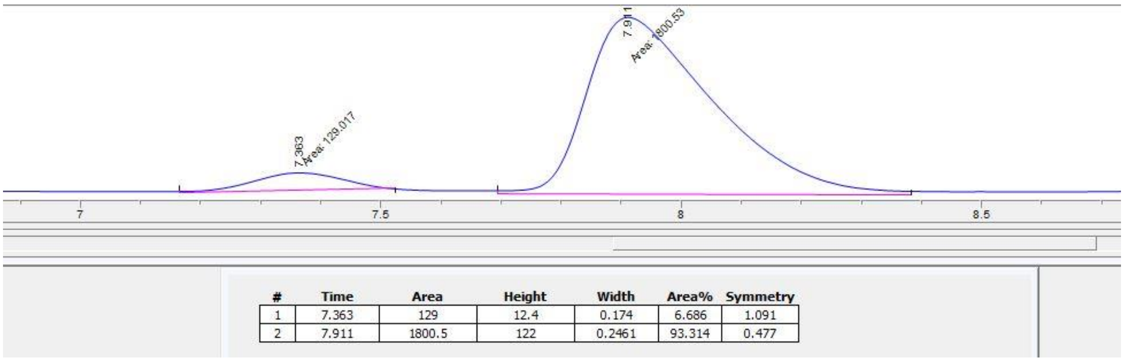

#	Time	Area	Height	Width	Area%	Symmetry
1	7.363	129	12.4	0.174	6.686	1.091
2	7.911	1800.5	122	0.2461	93.314	0.477

Isopropyl (*R*,*Z*)-3-phenyl-2-((phenylamino)methylene)butanoate (3da):

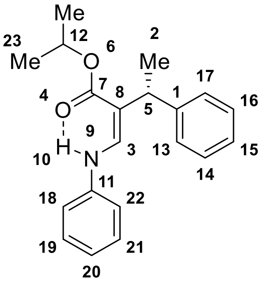

General procedure B: The reaction was carried out with substrate **1d** (20.5 mg, 0.10 mmol, 100 mol%) and styrene (34.0 µL, 0.30 mmol, 300 mol%). Purification of the residue by FCC (hexane/EtOAc 92:8) afforded the title compound (16.1 mg, 52%, >30:1 B:L, *Z/E* > 20:1, e.r. = 95:5) as a pale-yellow oil. ^{1}H NMR analysis of the crude material gave >30:1 B:L and *Z/E* > 20:1. $[\boldsymbol{\alpha}]_{\boldsymbol{D}}^{\mathbf{25}}$ = -92.0 (c = 1.0, $CHCl_3$); **IR (thin film)** ν_{max}/cm^{-1}: 3328 (br), 2927 (s), 1665 (s),

1600 (s), 1204 (s), 697 (s); **^{1}H NMR** (500 MHz, $CDCl_3$) δ 10.08 (d, *J* = 12.5 Hz, 1H, H^{10}), 7.32 – 7.27 (m, 6H, ArH), 7.24 – 7.17 (m, 2H, H^3 + ArH), 6.99 – 6.96 (m, 1H, H^{20}), 6.93 (d, *J* = 7.5 Hz, 2H, H^{18} + H^{22}), 5.05 – 4.96 (m, 1H, H^{12}), 4.01 (q, *J* = 7.0 Hz, 1H, H^5), 1.50 (d, *J* = 7.0 Hz, 3H, H^2), 1.23 (d, *J* = 6.5 Hz, 3H, H^{23}), 1.07 (d, *J* = 6.5 Hz, 3H, H^{23}); **^{13}C NMR** (126 MHz, $CDCl_3$) δ 169.4 (C^7), 146.8 (C^1), 141.4 (C^{11}), 140.3 (C^3), 129.6 (C^{19} + C^{21}), 128.1 (C^{14} + C^{16}), 127.4 (C^{13} + C^{17}), 125.7 (C^{15}), 121.8 (C^{20}), 115.1 (C^{18} + C^{22}), 105.2 (C^8), 66.8 (C^{12}), 38.8 (C^5), 22.0 (C^{23}), 21.8 (C^{23}), 21.1 (C^2); **HRMS** (ESI): calculated for $C_{20}H_{24}NO_2$ $[M+H]^+$ requires *m/z* 310.1802, found *m/z* 310.1803; **Chiral SFC:** DAICEL CHIRALCEL SC column (25 cm), CO_2:*i*-PrOH 99:1, 2.0 mL/min, 254 nm, 165 bar. Retention times: 5.1 mins (minor), 5.6 mins (major), e.r. = 95:5.

SFC analysis of the racemate, prepared using rac-BINAP:

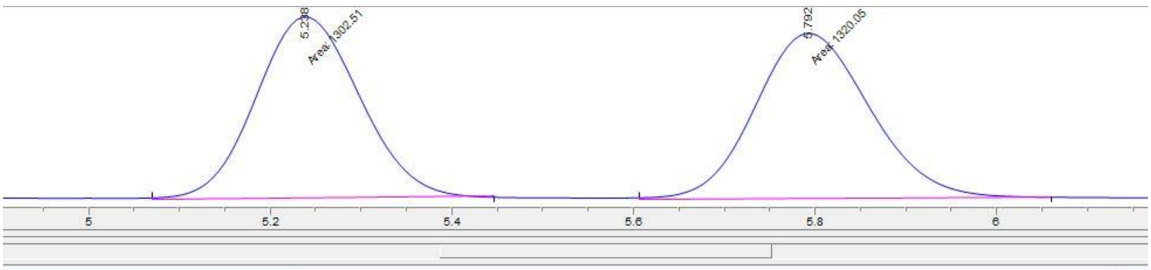

#	Time	Area	Height	Width	Area%	Symmetry
1	5.238	1302.5	164.2	0.1322	49.666	0.91
2	5.792	1320	149.9	0.1467	50.334	0.863

SFC analysis of the enantioenriched material, prepared using (R)-OMe-BIPHEP:

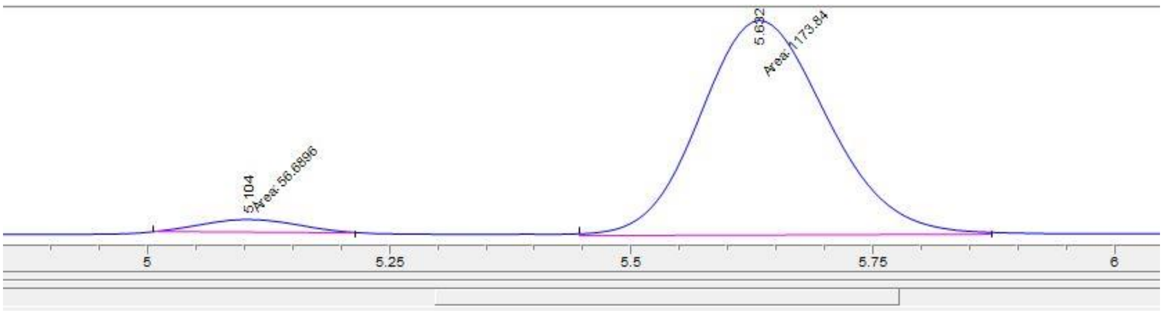

#	Time	Area	Height	Width	Area%	Symmetry
1	5.104	56.7	7.9	0.1193	4.607	0.873
2	5.632	1173.8	127.6	0.1533	95.393	0.86

Benzyl (*R*,*Z*)-3-phenyl-2-((*p*-tolylamino)methylene)butanoate (3ga):

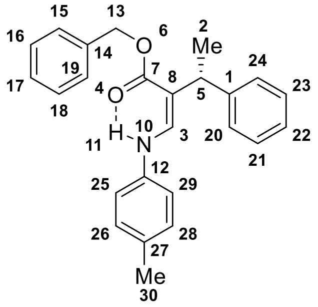

General procedure B: The reaction was carried out with substrate **1g** (26.7 mg, 0.10 mmol, 100 mol%) and styrene (34.0 µL, 0.30 mmol, 300 mol%). Purification of the residue by FCC (hexane/EtOAc 92:8) afforded the title compound (30.8 mg, 83%, >30:1 B:L, *Z/E* > 20:1, e.r. = 95:5) as a pale-yellow solid. ^{1}H NMR analysis of the crude material gave >30:1 B:L and *Z/E* > 20:1. **m.p.** = 53 – 55 °C (hexane/EtOAc); $[\alpha]_D^{25}$ = -80.5 (c = 1.0, $CHCl_3$); **IR (thin film)** ν_{max}/cm^{-1}: 3287 (br), 2957 (s), 1664 (s), 1604 (s), 1144 (s), 696 (s); **^{1}H NMR** (500 MHz, $CDCl_3$) δ 10.03 (d, *J* = 12.5 Hz, 1H, H^{11}), 7.34 – 7.22 (m, 9H, ArH), 7.15 – 7.11 (m, 4H, H^3 + ArH), 6.85 (d, *J* = 8.0 Hz, 2H, H^{25} + H^{29}), 5.19 – 5.12 (m, 2H, H^{13}), 4.11 (q, *J* = 7.0 Hz, 1H, H^5), 2.33 (s, 3H, H^{30}), 1.52 (d, *J* = 7.0 Hz, 3H, H^2); **^{13}C NMR** (126 MHz, $CDCl_3$) δ 169.6 (C^7), 146.8 (C^1), 141.7 (C^3), 138.9 (C^{14}), 136.7 (C^{12}), 131.7 (C^{27}), 130.2 (C^{26} + C^{28}), 128.4 (C^{16} + C^{18}), 128.2 (C^{21} + C^{23}), 127.7 (C^{17}), 127.5 (C^{15} + C^{19}), 127.4 (C^{20} + C^{24}), 125.8 (C^{22}), 115.4 (C^{25} + C^{29}), 103.5 (C^8), 65.3 (C^{13}), 38.6 (C^5), 21.5 (C^2), 20.7 (C^{30}); **HRMS** (ESI): calculated for $C_{25}H_{26}NO_2$ $[M+H]^+$ requires *m/z* 372.1958, found *m/z* 372.1959; **Chiral SFC:** DAICEL CHIRALCEL SC column (25 cm), CO_2:*i*-PrOH 95:5, 2.0 mL/min, 254 nm, 140 bar. Retention times: 10.2 mins (minor), 10.7 mins (major), e.r. = 95:5.

SFC analysis of the racemate, prepared using rac-BINAP:

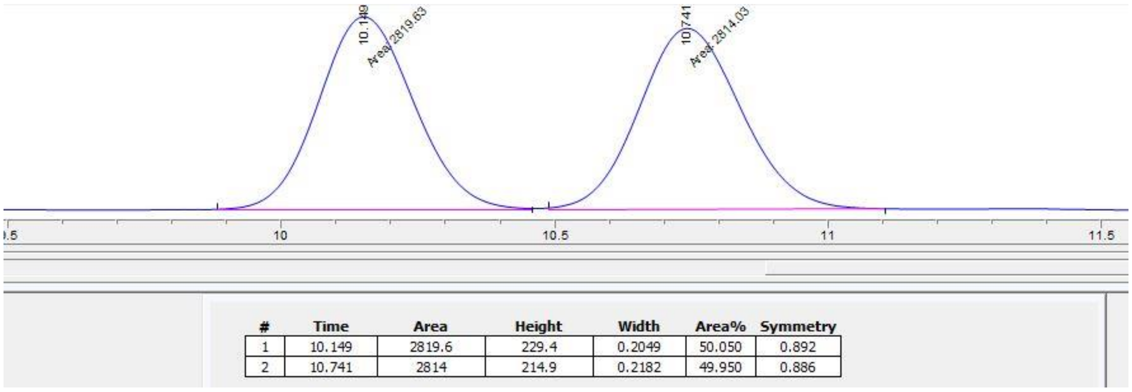

#	Time	Area	Height	Width	Area%	Symmetry
1	10.149	2819.6	229.4	0.2049	50.050	0.892
2	10.741	2814	214.9	0.2182	49.950	0.886

SFC analysis of the enantioenriched material, prepared using (R)-OMe-BIPHEP:

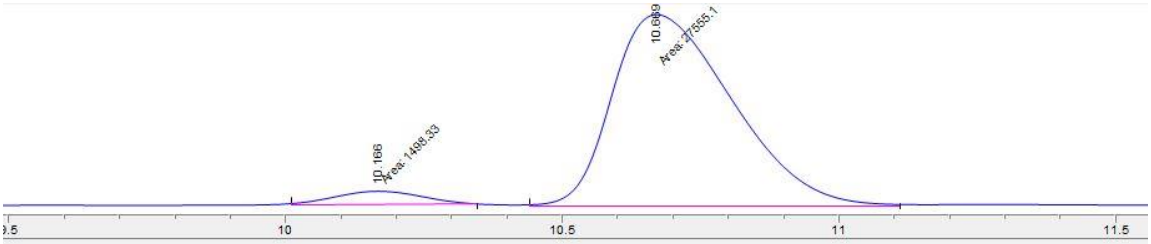

#	Time	Area	Height	Width	Area%	Symmetry
1	10.166	1498.3	132	0.1892	5.157	0.995
2	10.669	27555.1	1815.1	0.253	94.843	0.574

Benzyl (*R*,*Z*)-2-(((4-methoxyphenyl)amino)methylene)-3-phenylbutanoate (3ha):

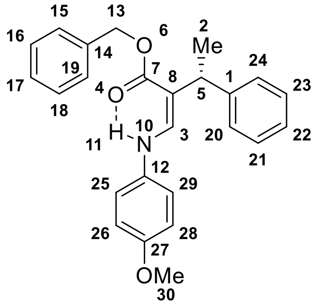

General procedure B: The reaction was carried out with substrate **1h** (28.3 mg, 0.10 mmol, 100 mol%) and styrene (34.0 µL, 0.30 mmol, 300 mol%). Purification of the residue by FCC (hexane/EtOAc 90:10) afforded the title compound (30.2 mg, 78%, >30:1 B:L, *Z/E* > 20:1, e.r. = 96:4) as a pale-yellow solid. ^{1}H NMR analysis of the crude material gave >30:1 B:L and *Z/E* > 20:1. **m.p.** = 74 – 76 °C (hexane/EtOAc); $[\boldsymbol{\alpha}]_{\boldsymbol{D}}^{\mathbf{25}}$ = -71.2 (c = 1.0, $CHCl_3$); **IR (thin film)** ν_{max}/cm^{-1}: 3306 (br), 2956 (s), 1666 (s), 1512 (s), 1172 (s), 696 (s); **^{1}H NMR** (500 MHz, $CDCl_3$) δ 9.98 (d, *J* = 12.5 Hz, 1H, H^{11}), 7.32 – 7.26 (m, 7H, ArH), 7.23 – 7.11 (m, 4H, H^3 + ArH), 6.89 – 6.85 (m, 4H, H^{25} + H^{26} + H^{28} + H^{29}), 5.18 – 5.11 (m, 2H, H^{13}), 4.09 (q, *J* = 7.0 Hz, 1H, H^5), 3.80 (s, 3H, H^{30}), 1.50 (d, *J* = 7.0 Hz, 3H, H^2); **^{13}C NMR** (126 MHz, $CDCl_3$) δ 169.6 (C^7), 155.3 (C^{27}), 146.9 (C^1), 142.4 (C^3), 136.7 (C^{12}), 135.0 (C^{14}), 128.4 (C^{16} + C^{18}), 128.2 (C^{21} + C^{23}), 127.7 (C^{17}), 127.5 (C^{15} + C^{19}), 127.4 (C^{20} + C^{24}), 125.8 (C^{22}), 116.9 (C^{26} + C^{28}), 115.0 (C^{25} + C^{29}), 103.0 (C^8), 65.2 (C^{13}), 55.6 (C^{30}), 38.5 (C^5), 21.5 (C^2); **HRMS** (ESI): calculated for $C_{25}H_{26}NO_3$ $[M+H]^+$ requires *m/z* 388.1907, found *m/z* 388.1905; **Chiral SFC:** DAICEL CHIRALCEL SC column (25 cm), CO_2:*i*-PrOH 95:5, 2.0 mL/min, 254 nm, 170 bar. Retention times: 12.5 mins (minor), 13.5 mins (major), e.r. = 96:4.

SFC analysis of the racemate, prepared using rac-BINAP:

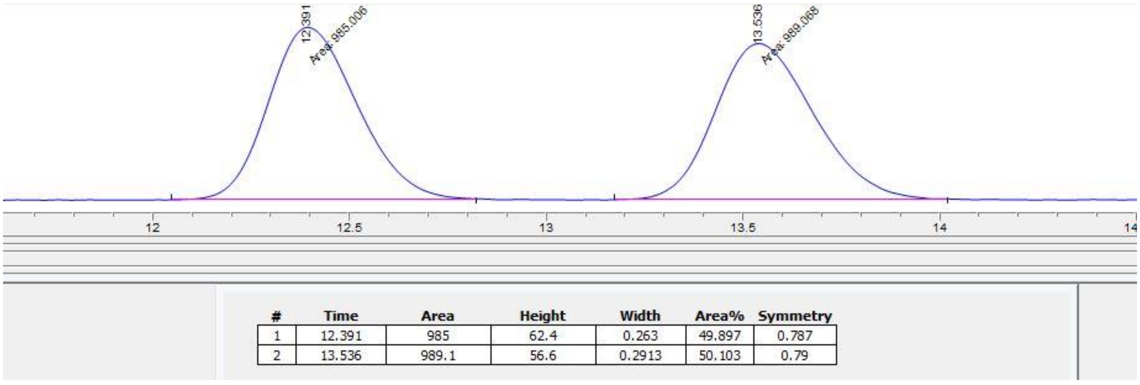

#	Time	Area	Height	Width	Area%	Symmetry
1	12.391	985	62.4	0.263	49.897	0.787
2	13.536	989.1	56.6	0.2913	50.103	0.79

SFC analysis of the enantioenriched material, prepared using (R)-OMe-BIPHEP:

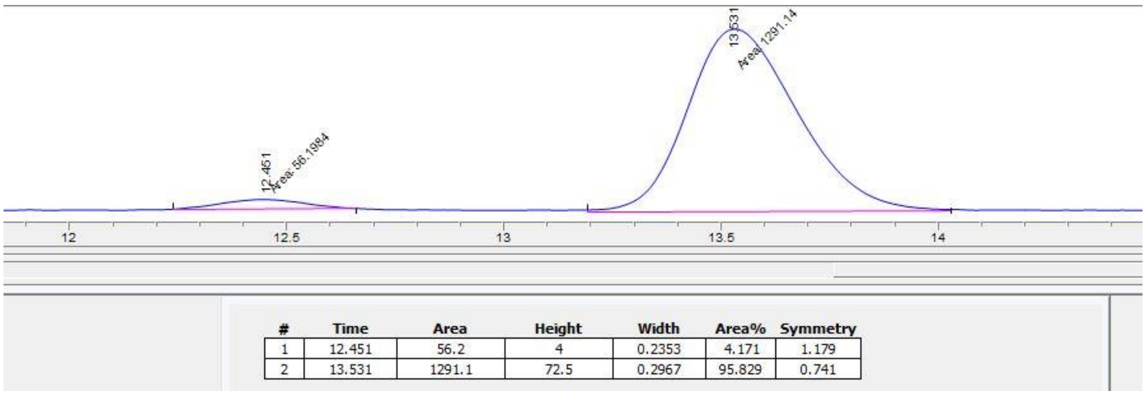

#	Time	Area	Height	Width	Area%	Symmetry
1	12.451	56.2	4	0.2353	4.171	1.179
2	13.531	1291.1	72.5	0.2967	95.829	0.741

Benzyl (*R*,*Z*)-2-(((4-hydroxyphenyl)amino)methylene)-3-phenylbutanoate (3ia):

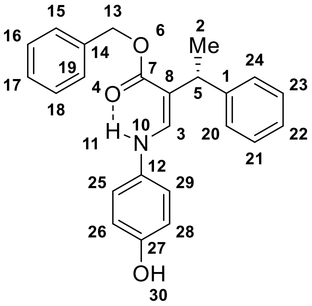

General procedure B: The reaction was carried out with substrate **1i** (26.9 mg, 0.10 mmol, 100 mol%) and styrene (34.0 µL, 0.30 mmol, 300 mol%). Purification of the residue by FCC (hexane/EtOAc 90:10) afforded the title compound (31.3 mg, 84%, >30:1 B:L, *Z/E* > 20:1, e.r. = 95:5) as a pale-yellow oil. ^{1}H NMR analysis of the crude material gave >30:1 B:L and *Z/E* > 20:1. $[\boldsymbol{\alpha}]_{\boldsymbol{D}}^{\mathbf{25}}$ = -61.4 (c = 1.0, $CHCl_3$); **IR (thin film)** ν_{max}/cm^{-1}: 3324 (br), 3028 (s), 2936 (s), 1659 (s), 1517 (s), 1201 (s), 699 (s); **^{1}H NMR** (500 MHz, $CDCl_3$) δ 9.95 (d, *J* = 12.5 Hz, 1H, H^{11}), 7.33 – 7.26 (m, 7H, ArH), 7.23 – 7.12 (m, 4H, H^3 + ArH), 6.83 – 6.78 (m, 4H, H^{25} + H^{26} + H^{28} + H^{29}), 5.18 – 5.11 (m, 2H, H^{13}), 4.82 (br. s, 1H, H^{30}), 4.09 (q, *J* = 7.0 Hz, 1H, H^5), 1.50 (d, *J* = 7.0 Hz, 3H, H^2); **^{13}C NMR** (126 MHz, $CDCl_3$) δ 169.7 (C^7), 151.1 (C^{27}), 146.8 (C^1),

142.4 (C^3), 136.7 (C^{12}), 135.1 (C^{14}), 128.4 (C^{16} + C^{18}), 128.2 (C^{21} + C^{23}), 127.7 (C^{17}), 127.5 (C^{15} + C^{19}), 127.4 (C^{20} + C^{24}), 125.8 (C^{22}), 117.1 (C^{26} + C^{28}), 116.4 (C^{25} + C^{29}), 103.0 (C^8), 65.3 (C^{13}), 38.5 (C^5), 21.5 (C^2); **HRMS** (ESI): calculated for $C_{24}H_{24}NO_3$ $[M+H]^+$ requires *m/z* 374.1751, found *m/z* 374.1753; **Chiral SFC:** DAICEL CHIRALCEL SC column (25 cm), CO_2:*i*-PrOH 90:10, 2.0 mL/min, 254 nm, 172 bar. Retention times: 10.9 mins (minor), 11.8 mins (major), e.r. = 95:5.

SFC analysis of the racemate, prepared using rac-BINAP:

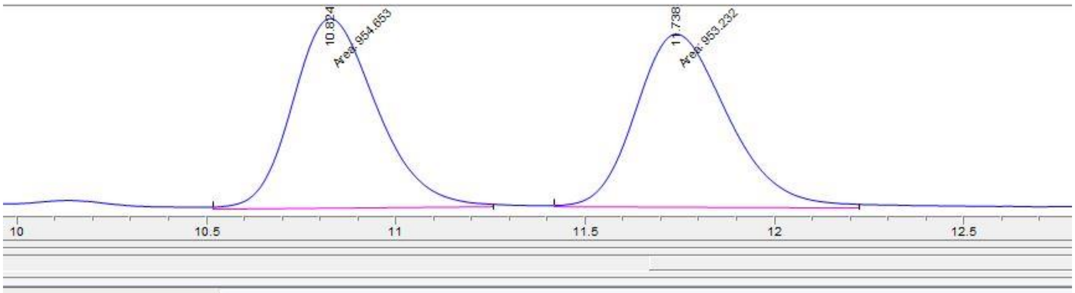

#	Time	Area	Height	Width	Area%	Symmetry
1	10.824	954.7	61.3	0.2595	50.037	0.791
2	11.738	953.2	56	0.2838	49.963	0.761

SFC analysis of the enantioenriched material, prepared using (R)-OMe-BIPHEP:

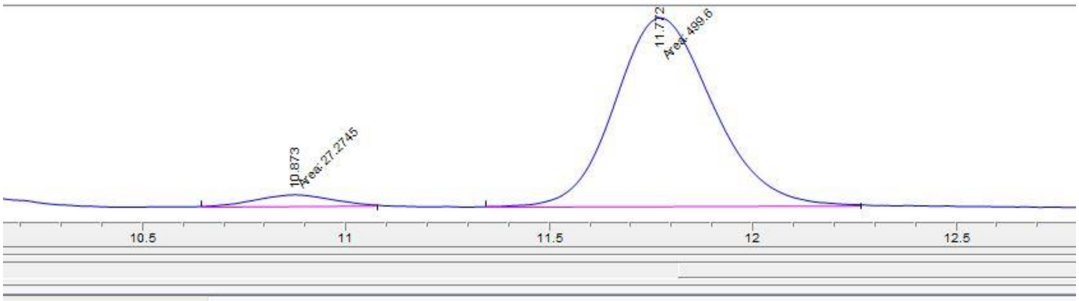

#	Time	Area	Height	Width	Area%	Symmetry
1	10.873	27.3	1.9	0.2358	5.177	1.181
2	11.772	499.6	29.4	0.2834	94.823	0.852

Benzyl (*R*,*Z*)-2-(((4-fluorophenyl)amino)methylene)-3-phenylbutanoate (3ja):

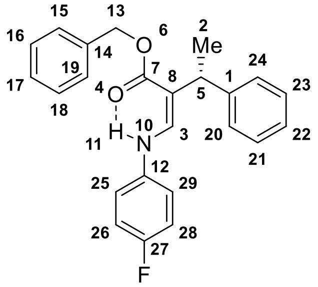

General procedure B: The reaction was carried out with substrate **1j** (27.1 mg, 0.10 mmol, 100 mol%) and styrene (34.0 µL, 0.30 mmol, 300 mol%). Purification of the residue by FCC

(hexane/EtOAc 90:10) afforded the title compound (22.5 mg, 60%, >30:1 B:L, *Z/E* > 20:1, e.r. = 95:5) as a pale-yellow oil. ^{1}H NMR analysis of the crude material gave >30:1 B:L and *Z/E* = 13:1. $[\boldsymbol{\alpha}]_{\boldsymbol{D}}^{\boldsymbol{25}}$ = -55.4 (c = 1.0, $CHCl_3$); **IR (thin film)** ν_{max}/cm^{-1}: 3321 (br), 2959 (s), 1665 (s), 1509 (s), 1197 (s), 693 (s); **^{1}H NMR** (500 MHz, $CDCl_3$) δ 10.04 (d, *J* = 12.5 Hz, 1H, H^{11}), 7.32 – 7.21 (m, 8H, ArH), 7.17 – 7.11 (m, 2H, H^3 + ArH), 7.03 – 6.99 (m, 2H, H^{26} + H^{28}), 6.88 – 6.86 (m, 2H, H^{25} + H^{29}), 5.18 – 5.11 (m, 2H, H^{13}), 4.09 (q, *J* = 7.0 Hz, 1H, H^5), 1.50 (d, *J* = 7.0 Hz, 3H, H^2); **^{13}C NMR** (126 MHz, $CDCl_3$) δ 169.6 (C^7), 158.4 (d, *J* = 238.8 Hz, C^{27}), 146.6 (C^1), 141.6 (C^3), 137.6 (d, *J* = 2.5 Hz, C^{12}), 136.5 (C^{14}), 128.4 (C^{16} + C^{18}), 128.3 (C^{21} + C^{23}), 127.8 (C^{17}), 127.5 (C^{15} + C^{19}), 127.4 (C^{20} + C^{24}), 125.9 (C^{22}), 116.6 (d, *J* = 7.5 Hz, C^{25} + C^{29}), 116.3 (d, *J* = 22.5 Hz, C^{26} + C^{28}), 104.3 (C^8), 65.4 (C^{13}), 38.6 (C^5), 21.4 (C^2); ^{19}F NMR (471 MHz, $CDCl_3$) δ -121.7; **HRMS**: a stable ion was not found in ESI and CI. **Chiral SFC:** DAICEL CHIRALCEL SC column (25 cm), CO_2:*i*-PrOH 95:5, 2.0 mL/min, 254 nm, 170 bar. Retention times: 5.5 mins (minor), 5.8 mins (major), e.r. = 95:5.

SFC analysis of the racemates, prepared using rac-BINAP:

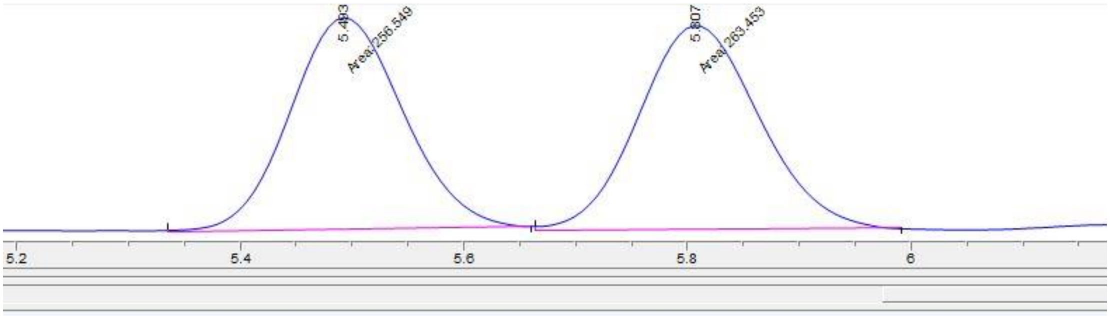

#	Time	Area	Height	Width	Area%	Symmetry
1	5.493	256.5	36.4	0.1175	49.336	0.904
2	5.807	263.5	35	0.1254	50.664	0.899

SFC analysis of the enantioenriched material, prepared using (R)-OMe-BIPHEP:

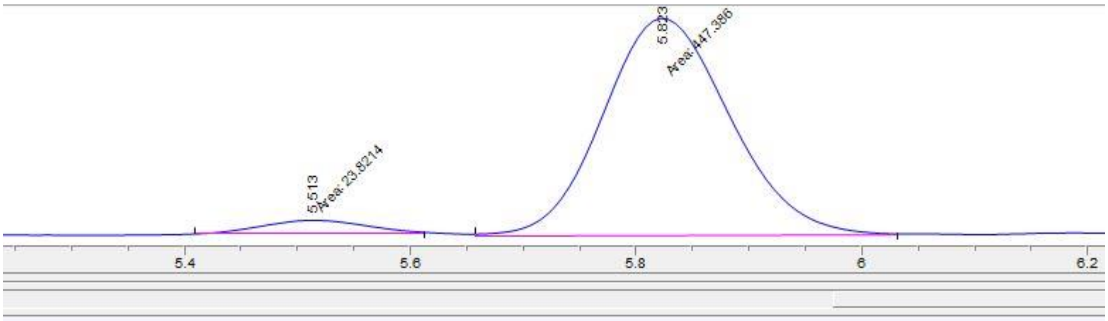

#	Time	Area	Height	Width	Area%	Symmetry
1	5.513	23.8	3.6	0.1103	5.055	0.885
2	5.823	447.4	56.6	0.1316	94.945	0.866

Benzyl (*R*,*Z*)-2-(((4-chlorophenyl)amino)methylene)-3-phenylbutanoate (3ka):

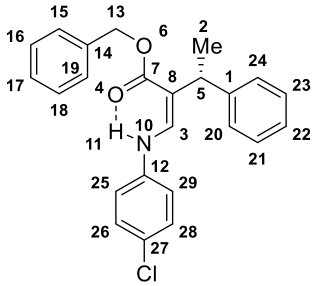

General procedure B: The reaction was carried out with substrate **1k** (28.7 mg, 0.10 mmol, 100 mol%) and styrene (34.0 µL, 0.30 mmol, 300 mol%). Purification of the residue by FCC (hexane/EtOAc 90:10) afforded the title compound (37.2 mg, 95%, >30:1 B:L, *Z/E* > 20:1, e.r. = 95:5) as a pale-yellow solid. ^{1}H NMR analysis of the crude material gave >30:1 B:L and *Z/E* = 13:1. **m.p.** = 74 – 76 °C (hexane/EtOAc); $[\boldsymbol{\alpha}]_{\boldsymbol{D}}^{\mathbf{25}}$ = -73.6 (c = 1.0, $CHCl_3$); **IR (thin film)** ν_{max}/cm^{-1}: 3295 (br), 2961 (s), 1663 (s), 1616(s), 1142 (s), 698 (s); **^{1}H NMR** (500 MHz, $CDCl_3$) δ 10.09 (d, *J* = 12.5 Hz, 1H, H^{11}), 7.33 – 7.22 (m, 10H, ArH), 7.18 (d, *J* = 12.5 Hz, 1H, H^3), 7.13 – 7.12 (m, 2H, ArH), 6.85 (d, *J* = 9.0 Hz, 2H, H^{25} + H^{29}), 5.19 – 5.12 (m, 2H, H^{13}), 4.10 (q, *J* = 7.0 Hz, 1H, H^5), 1.51 (d, *J* = 7.0 Hz, 3H, H^2). **^{13}C NMR** (126 MHz, $CDCl_3$) δ 169.5 (C^7), 146.4 (C^1), 140.6 (C^{12}), 139.9 (C^3), 136.4 (C^{14}), 129.6 (C^{26} + C^{28}), 128.4 (C^{16} + C^{18}), 128.3 (C^{21} + C^{23}), 127.8 (C^{17}), 127.6 (C^{15} + C^{19}), 127.4 (C^{20} + C^{24}), 126.9 (C^{27}), 126.0 (C^{22}), 116.4 (C^{25} + C^{29}), 105.2 (C^8), 65.5 (C^{13}), 38.6 (C^5), 21.4 (C^2); **HRMS** (ESI): calculated for $C_{24}H_{23}{}^{35}Cl NO_2$ $[M+H]^+$ requires *m/z* 392.1412, found *m/z* 392.1414; **Chiral SFC:** DAICEL CHIRALCEL SC column (25 cm), CO_2:*i*-PrOH 95:5, 2.0 mL/min, 254 nm, 170 bar. Retention times: 6.4 mins (minor), 6.7 mins (major), e.r. = 95:5.

SFC analysis of the racemate, prepared using rac-BINAP:

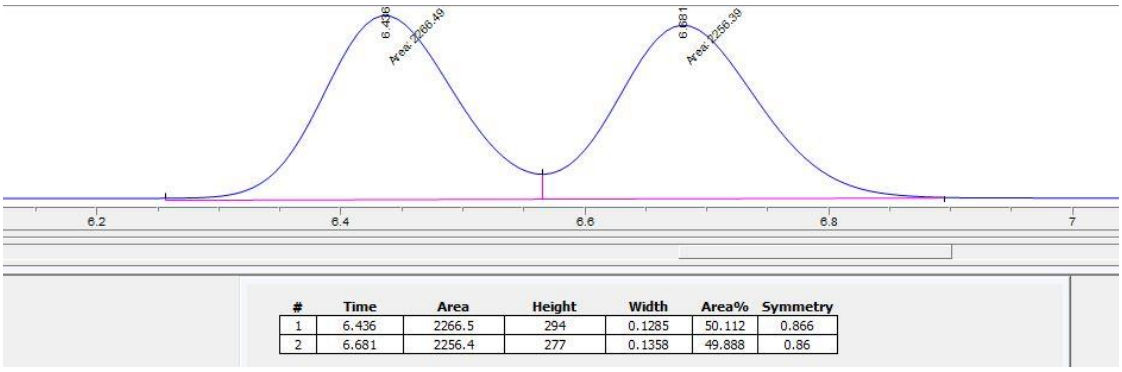

#	Time	Area	Height	Width	Area%	Symmetry
1	6.436	2266.5	294	0.1285	50.112	0.866
2	6.681	2256.4	277	0.1358	49.888	0.86

SFC analysis of the enantioenriched material, prepared using (R)-OMe-BIPHEP:

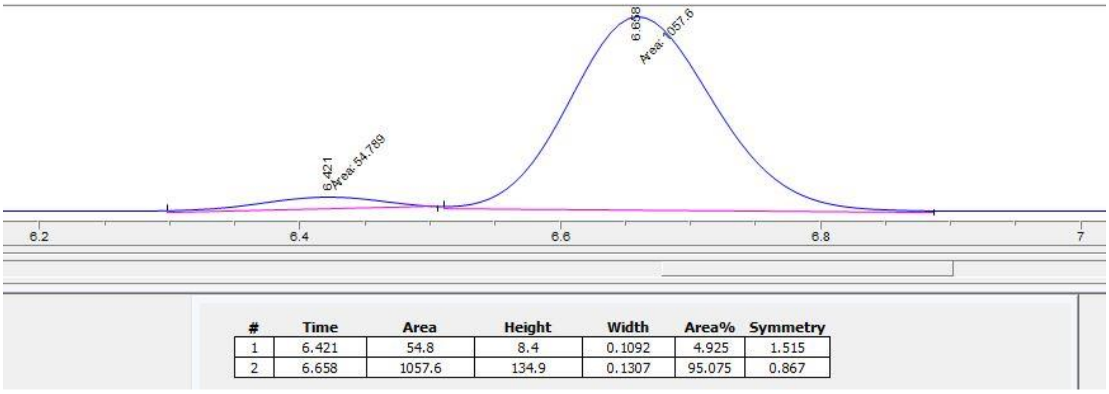

#	Time	Area	Height	Width	Area%	Symmetry
1	6.421	54.8	8.4	0.1092	4.925	1.515
2	6.658	1057.6	134.9	0.1307	95.075	0.867

Benzyl (*R*,*Z*)-2-((phenylamino)methylene)-3-(*p*-tolyl)butanoate (3ab):

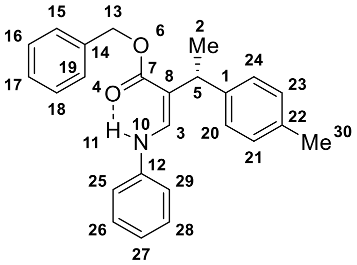

General procedure B: The reaction was carried out with substrate **1a** (25.3 mg, 0.10 mmol, 100 mol%), 1-methyl-4-vinylbenzene **1b** (65.0 µL, 0.50 mmol, 500 mol%), Ir(cod)$_2$BARF (9.54 mg, 7.50 µmol, 7.5 mol%) and (*R*)-OMe-BIPHEP (4.37 mg, 7.50 µmol, 7.5 mol%). Purification of the residue by FCC (hexane/EtOAc 92:8) afforded the title compound (31.2 mg, 84%, >30:1 B:L, *Z/E* > 20:1, e.r. = 95:5) as a pale-yellow oil. ^{1}H NMR analysis of the crude material gave >30:1 B:L and *Z/E* > 20:1. $[\boldsymbol{\alpha}]_{\boldsymbol{D}}^{\mathbf{25}}$ = -78.4 (c = 1.0, $CHCl_3$); **IR (thin film)** ν_{max}/cm^{-1}: 3304 (br), 2924 (s), 1671 (s), 1598 (s), 1138 (s), 689 (s); **^{1}H NMR** (500 MHz, $CDCl_3$) δ 10.07 (d, *J* = 12.5 Hz, 1H, H^{11}), 7.34 – 7.27 (m, 6H, H^3 + ArH), 7.19 – 7.12 (m, 6H, ArH), 7.02 – 6.99 (m, 1H, H^{27}), 6.94 (d, *J* = 7.5 Hz, 2H, H^{25} + H^{29}), 5.21 – 5.14 (m, 2H, H^{13}), 4.08 (q, *J* = 7.0 Hz, 1H, H^5), 2.38 (s, 3H, H^{30}), 1.51 (d, *J* = 7.0 Hz, 3H, H^2); **^{13}C NMR** (126 MHz, $CDCl_3$) δ 169.5 (C^7), 143.6 (C^1), 141.3 (C^{12}), 141.0 (C^3), 136.7 (C^{14}), 135.3 (C^{22}), 129.7 (C^{26} + C^{28}), 128.9 (C^{16} + C^{18}), 128.4 (C^{21} + C^{23}), 127.7 (C^{17}), 127.6 (C^{15} + C^{19}), 127.3 (C^{20} + C^{24}), 122.1 (C^{27}), 115.3 (C^{25} + C^{29}), 104.5 (C^8), 65.3 (C^{13}), 38.2 (C^5), 21.6 (C^2), 21.1 (C^{30}); **HRMS** (ESl): calculated for $C_{25}H_{25}NO_2Na$ [M+Na]$^+$ requires *m/z* 394.1778, found *m/z* 394.1781; **Chiral SFC:** DAICEL CHIRALCEL SC column (25 cm), CO_2:*i*-PrOH 95:5, 2.0 mL/min, 254 nm, 170 bar. Retention times: 7.0 mins (minor), 7.8 mins (major), e.r. = 95:5.

SFC analysis of the racemate, prepared using rac-BINAP:

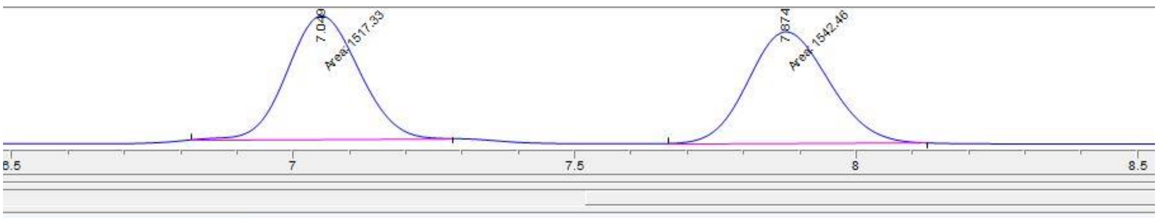

#	Time	Area	Height	Width	Area%	Symmetry
1	7.049	1517.3	166.6	0.1518	49.589	0.909
2	7.874	1542.5	150.7	0.1705	50.411	0.87

SFC analysis of the enantioenriched material, prepared using (R)-OMe-BIPHEP:

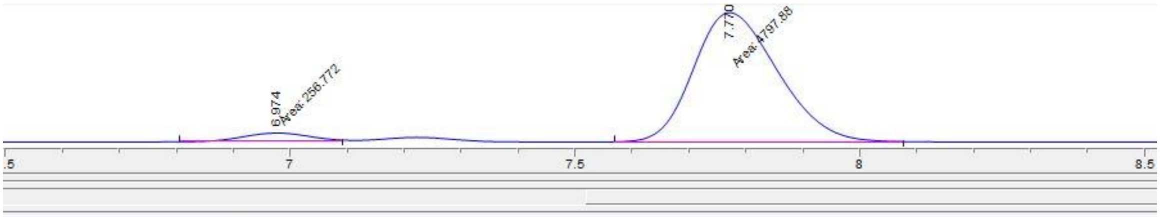

#	Time	Area	Height	Width	Area%	Symmetry
1	6.974	256.8	30.5	0.1405	5.080	1.057
2	7.77	4797.9	459.8	0.1739	94.920	0.734

Benzyl (*R*,*Z*)-3-(4-methoxyphenyl)-2-((phenylamino)methylene)butanoate (3ac):

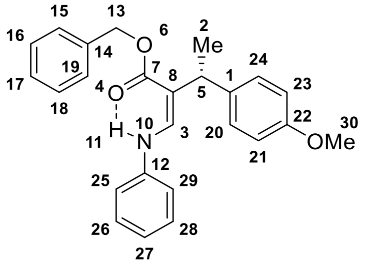

General procedure B: The reaction was carried out with substrate **1a** (25.3 mg, 0.10 mmol, 100 mol%) and 1-methoxy-4-vinylbenzene **1c** (40.0 µL, 0.30 mmol, 300 mol%). Purification of the residue by FCC (hexane/EtOAc 90:10) afforded the title compound (21.7 mg, 56%, >30:1 B:L, *Z/E* > 20:1, e.r. = 95:5) as a colorless solid. ^{1}H NMR analysis of the crude material gave >30:1 B:L and *Z/E* > 20:1. **m.p.** = 51 – 53 °C (hexane/EtOAc); $[\boldsymbol{\alpha}]_{\boldsymbol{D}}^{\mathbf{25}}$ = -100.4 (c = 1.0, $CHCl_3$); **IR (thin film)** ν_{max}/cm^{-1}: 3305 (br), 2928 (s), 1667 (s), 1600 (s), 1176 (s), 695 (s); **^{1}H NMR** (500 MHz, $CDCl_3$) δ 10.03 (d, *J* = 12.5 Hz, 1H, H^{11}), 7.35 – 7.28 (m, 5H, ArH), 7.24 (d, *J* = 12.5 Hz, 1H, H^3), 7.19 – 7.17 (m, 4H, ArH), 7.00 – 6.98 (m, 1H, H^{27}), 6.92 (d, *J* = 8.0 Hz, 2H, H^{25} + H^{29}), 6.85 (d, *J* = 8.5 Hz, 2H, H^{21} + H^{23}), 5.20 – 5.13 (m, 2H, H^{13}), 4.05 (q, *J* = 7.0 Hz, 1H, H^5), 3.83 (s, 3H, H^{30}), 1.49 (d, *J* = 7.0 Hz, 3H, H^2); **^{13}C NMR** (126 MHz, $CDCl_3$) δ 169.5 (C^7), 157.8 (C^{22}), 141.2 (C^1), 141.0 (C^3), 138.7 (C^1), 136.6 (C^{14}), 129.6 (C^{26} + C^{28}), 128.39 (C^{16} + C^{18}), 128.35 (C^{20} + C^{24}), 127.8 (C^{17}), 127.6 (C^{15} + C^{19}), 122.1 (C^{27}), 115.3 (C^{25} + C^{29}), 113.6 (C^{21} + C^{23}), 104.7 (C^8), 65.3 (C^{13}), 55.3 (C^{30}), 37.8 (C^5), 21.6 (C^2); **HRMS** (ESI):

calculated for $C_{25}H_{25}NO_3Na$ $[M+Na]^+$ requires *m/z* 410.1727, found *m/z* 410.1614; **Chiral SFC:** DAICEL CHIRALCEL SC column (25 cm), CO_2:*i*-PrOH 95:5, 2.0 mL/min, 254 nm, 170 bar. Retention times: 10.2 mins (minor), 11.2 mins (major), e.r. = 95:5.

SFC analysis of the racemate, prepared using rac-BINAP:

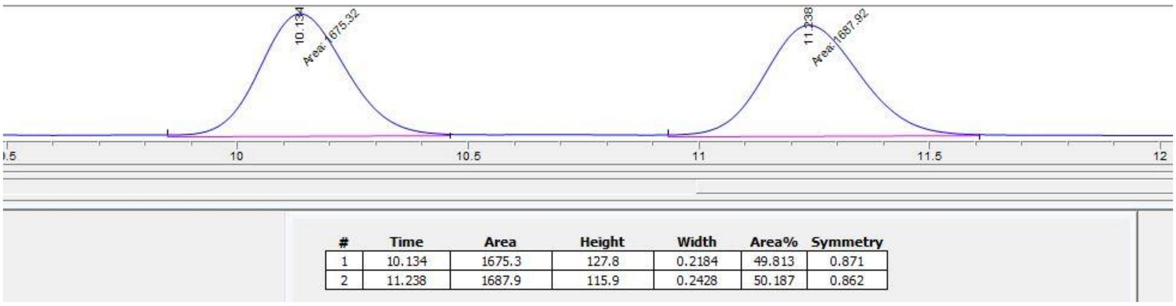

#	Time	Area	Height	Width	Area%	Symmetry
1	10.134	1675.3	127.8	0.2184	49.813	0.871
2	11.238	1687.9	115.9	0.2428	50.187	0.862

SFC analysis of the enantioenriched material, prepared using (R)-OMe-BIPHEP:

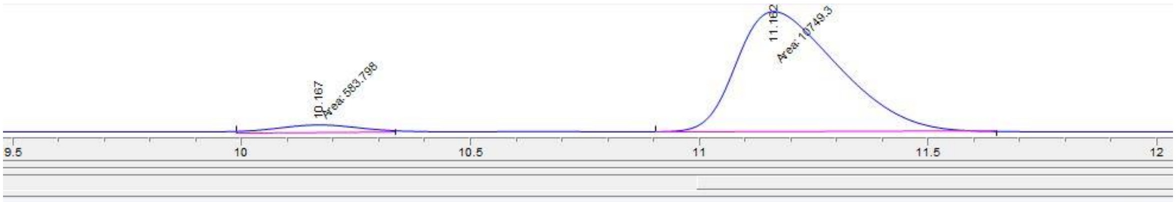

#	Time	Area	Height	Width	Area%	Symmetry
1	10.167	583.8	45.2	0.2151	5.151	1.089
2	11.162	10749.3	699.2	0.2562	94.849	0.569

Benzyl (*R*,*Z*)-3-(4-(*tert*-butyl)phenyl)-2-((phenylamino)methylene)butanoate (3ad):

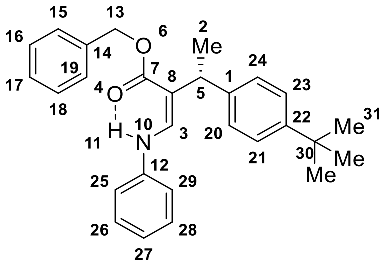

General procedure B: The reaction was carried out with substrate **1a** (25.3 mg, 0.10 mmol, 100 mol%), 1-(*tert*-butyl)-4-vinylbenzene **1d** (90.0 µL, 0.50 mmol, 500 mol%), Ir(cod)$_2$BARF (9.54 mg, 7.50 µmol, 7.5 mol%) and (*R*)-OMe-BIPHEP (4.37 mg, 7.50 µmol, 7.5 mol%). Purification of the residue by FCC (hexane/EtOAc 92:8) afforded the title compound (36.8 mg, 89%, >30:1 B:L, *Z/E* > 20:1, e.r. = 92:8) as a pale-yellow oil. ^{1}H NMR analysis of the crude material gave >30:1 B:L and *Z/E* > 20:1. $[\alpha]_D^{25}$ = -74.0 (c = 1.0, $CHCl_3$); **IR (thin film)** ν_{max}/cm^{-1}: 3295 (br), 2961 (s), 1668 (s), 1600 (s), 1178 (s), 694 (s); **^{1}H NMR** (500 MHz, $CDCl_3$) δ 10.07 (d, *J* = 12.5 Hz, 1H, H^{11}), 7.35 – 7.27 (m, 8H, H^3 + ArH), 7.22 (d, *J* = 8.5 Hz, 2H, H^{20} + H^{24}), 7.15 – 7.13 (m, 2H, ArH), 7.02 – 6.99 (m, 1H, H^{27}), 6.94 (d, *J* = 7.5 Hz, 2H,

H^{25} + H^{29}), 5.22 – 5.13 (m, 2H, H^{13}), 4.09 (q, *J* = 7.0 Hz, 1H, H^{5}), 1.53 (d, *J* = 7.0 Hz, 3H, H^{2}), 1.38 (s, 9H, H^{31}); **^{13}C NMR** (126 MHz, $CDCl_3$) δ 169.6 (C^{7}), 148.6 (C^{22}), 143.5 (C^{1}), 141.3 (C^{12}), 141.1 (C^{3}), 136.7 (C^{14}), 129.7 (C^{26} + C^{28}), 128.4 (C^{16} + C^{18}), 127.7 (C^{17}), 127.5 (C^{15} + C^{19}), 127.1 (C^{20} + C^{24}), 125.1 (C^{21} + C^{23}), 122.1 (C^{27}), 115.3 (C^{25} + C^{29}), 104.6 (C^{8}), 65.4 (C^{13}), 38.1 (C^{5}), 34.4 (C^{30}), 31.5 (C^{31}), 21.4 (C^{2}); **HRMS** (ESl): calculated for $C_{28}H_{31}NO_2Na$ $[M+Na]^+$ requires *m/z* 436.2247, found *m/z* 436.2247; **Chiral SFC:** DAICEL CHIRALCEL SC column (25 cm), CO_2:*i*-PrOH 95:5, 2.0 mL/min, 254 nm, 170 bar. Retention times: 4.7 mins (minor), 5.1 mins (major), e.r. = 92:8.

SFC analysis of the racemate, prepared using rac-BINAP:

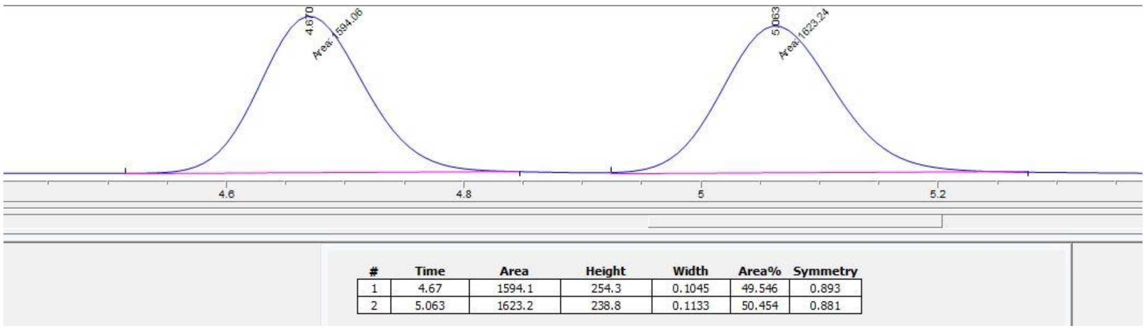

#	Time	Area	Height	Width	Area%	Symmetry
1	4.67	1594.1	254.3	0.1045	49.546	0.893
2	5.063	1623.2	238.8	0.1133	50.454	0.881

SFC analysis of the enantioenriched material, prepared using (R)-OMe-BIPHEP:

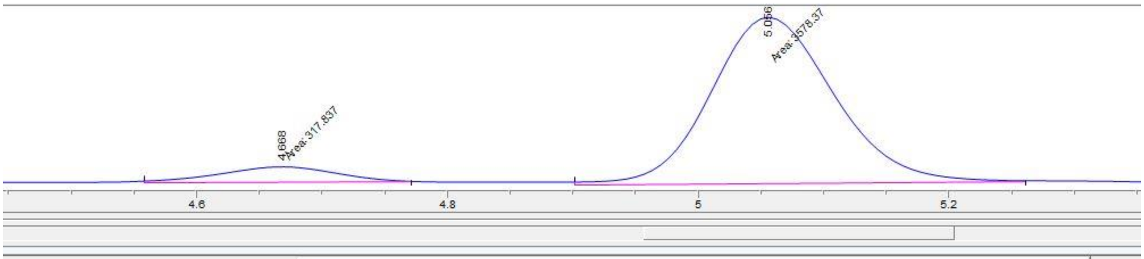

#	Time	Area	Height	Width	Area%	Symmetry
1	4.668	317.8	48.7	0.1087	8.158	1.137
2	5.056	3578.4	523.3	0.114	91.842	0.87

Benzyl (*R,Z*)-2-((phenylamino)methylene)-3-(4-(trimethylsilyl)phenyl)butanoate (3ae):

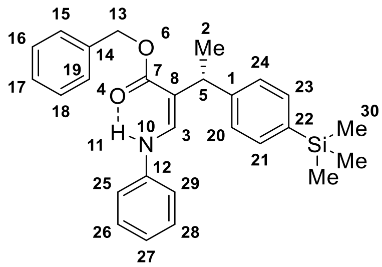

General procedure B: The reaction was carried out with substrate **1a** (25.3 mg, 0.10 mmol, 100 mol%), trimethyl(4-vinylphenyl)silane **1e** (98.0 μL, 0.50 mmol, 500 mol%),

Ir(cod)$_2$BARF (9.54 mg, 7.50 µmol, 7.5 mol%) and (*R*)-OMe-BIPHEP (4.37 mg, 7.50 µmol, 7.5 mol%). Purification of the residue by FCC (hexane/EtOAc 92:8) afforded the title compound (31.8 mg, 74%, >30:1 B:L, *Z/E* > 20:1, e.r. = 95:5) as a pale-yellow solid. ^{1}H NMR analysis of the crude material gave >30:1 B:L and *Z/E* > 20:1. **m.p.** = 72 – 74 °C (hexane/EtOAc); $[\alpha]_D^{25}$ = -96.5 (c = 1.0, $CHCl_3$); **IR (thin film)** ν_{max}/cm^{-1}: 3335 (br), 2956 (s), 1671 (s), 1598 (s), 1189 (s), 836 (s), 693 (s); **^{1}H NMR** (500 MHz, $CDCl_3$) δ 10.08 (d, *J* = 12.5 Hz, 1H, H^{11}), 7.48 (d, *J* = 7.5 Hz, 2H, H^{21} + H^{23}), 7.34 – 7.27 (m, 8H, H^3 + ArH), 7.12 – 7.10 (m, 2H, ArH), 7.03 – 7.00 (m, 1H, H^{27}), 6.96 (d, *J* = 8.0 Hz, 2H, H^{25} + H^{29}), 5.21 – 5.12 (m, 2H, H^{13}), 4.10 (q, *J* = 7.0 Hz, 1H, H^5), 1.53 (d, *J* = 7.0 Hz, 3H, H^2), 0.32 (s, 9H, H^{30}); **^{13}C NMR** (126 MHz, $CDCl_3$) δ 169.5 (C^7), 147.4 (C^1), 141.2 (C^{12}), 141.1 (C^3), 137.4 (C^{22}), 136.6 (C^{14}), 133.3 (C^{21} + C^{23}), 129.7 (C^{26} + C^{28}), 128.4 (C^{16} + C^{18}), 127.7 (C^{17}), 127.5 (C^{15} + C^{19}), 126.9 (C^{20} + C^{24}), 122.1 (C^{27}), 115.4 (C^{25} + C^{29}), 104.2 (C^8), 65.4 (C^{13}), 38.6 (C^5), 21.3 (C^2), -1.0 (C^{30}); **HRMS** (ESl): calculated for $C_{27}H_{31}NO_2SiNa$ $[M+Na]^+$ requires *m/z* 452.2016, found *m/z* 452.2013; **Chiral SFC:** DAICEL CHIRALCEL SC column (25 cm), CO_2:*i*-PrOH 95:5, 2.0 mL/min, 254 nm, 174 bar. Retention times: 5.4 mins (minor), 6.1 mins (major), e.r. = 95:5.

SFC analysis of the of racemate, prepared using rac-BINAP:

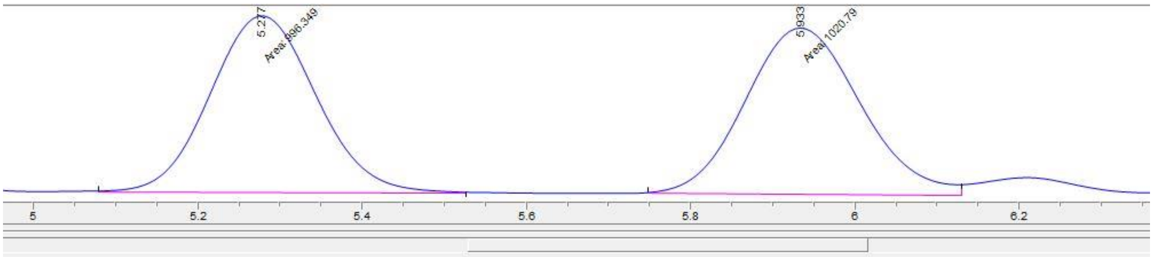

#	Time	Area	Height	Width	Area%	Symmetry
1	5.277	996.3	110.1	0.1508	49.394	0.925
2	5.933	1020.8	103.7	0.164	50.606	0.884

SFC analysis of the enantioenriched material, prepared using (R)-OMe-BIPHEP:

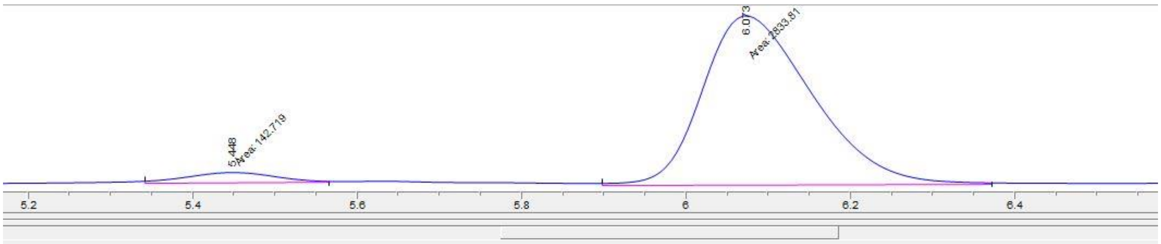

#	Time	Area	Height	Width	Area%	Symmetry
1	5.448	142.7	18.8	0.1268	4.795	1.067
2	6.073	2833.8	304.3	0.1552	95.205	0.648

Benzyl (*R*,*Z*)-3-([1,1'-biphenyl]-4-yl)-2-((phenylamino)methylene)butanoate (3af):

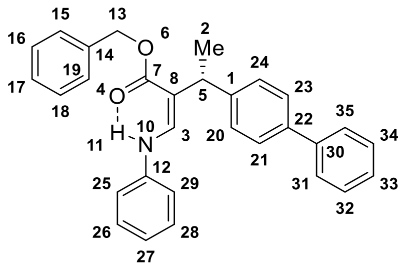

General procedure B: The reaction was carried out with substrate **1a** (25.3 mg, 0.10 mmol, 100 mol%) and 4-vinyl-1,1'-biphenyl **1f** (54.0 mg, 0.30 mmol, 300 mol%). Purification of the residue by FCC (hexane/EtOAc 92:8) afforded the title compound (41.6 mg, 96%, >30:1 B:L, *Z/E* > 20:1, e.r. = 95:5) as a pale-yellow solid. ^{1}H NMR analysis of the crude material gave >30:1 B:L and *Z/E* = 15:1. **m.p.** = 79 – 81 °C (hexane/EtOAc); $[\boldsymbol{\alpha}]_{\boldsymbol{D}}^{\mathbf{25}}$ = -110.0 (c = 1.0, $CHCl_3$); **IR (thin film)** ν_{max}/cm^{-1}: 3315 (br), 2935 (s), 1663 (s), 1597 (s), 1193 (s), 690 (s); **^{1}H NMR** (500 MHz, $CDCl_3$) δ 10.12 (d, *J* = 12.5 Hz, 1H, H^{11}), 7.65 (d, *J* = 7.0 Hz, 2H, H^{31} + H^{35}), 7.56 (d, *J* = 8.2 Hz, 2H, H^{20} + H^{24}), 7.50 – 7.47 (m, 2H, H^{32} + H^{34}), 7.40 – 7.28 (m, 9H, H^3 + ArH), 7.15 – 7.13 (m, 2H, ArH), 7.03 – 7.00 (m, 1H, H^{27}), 6.97 (d, *J* = 7.5 Hz, 2H, H^{25} + H^{29}), 5.23 – 5.13 (m, 2H, H^{13}), 4.15 (q, *J* = 7.0 Hz, 1H, H^5), 1.56 (d, *J* = 7.0 Hz, 3H, H^2); **^{13}C NMR** (126 MHz, $CDCl_3$) δ 169.5 (C^7), 146.0 (C^1), 141.22 (C^{12}), 141.17 (C^3), 141.1 (C^{30}), 138.8 (C^{22}), 136.6 (C^{14}), 129.7 (C^{26} + C^{28}), 128.8 (C^{16} + C^{18}), 128.4 (C^{21} + C^{23}), 127.84 (ArC), 127.78 (C^{17}), 127.6 (C^{15} + C^{19}), 127.04 (ArC), 126.98 (ArC), 122.2 (C^{27}), 115.4 (C^{25} + C^{29}), 104.1 (C^8), 65.4 (C^{13}), 38.4 (C^5), 21.4 (C^2); **HRMS** (ESI): calculated for $C_{30}H_{27}NO_2Na$ $[M+Na]^+$ requires *m/z* 456.1934, found *m/z* 456.1933; **Chiral SFC:** DAICEL CHIRALCEL SC column (25 cm), CO_2:*i*-PrOH 85:15, 2.0 mL/min, 254 nm, 175 bar. Retention times: 6.7 mins (minor), 7.5 mins (major), e.r. = 95:5.

SFC analysis of the of racemate, prepared using rac-BINAP:

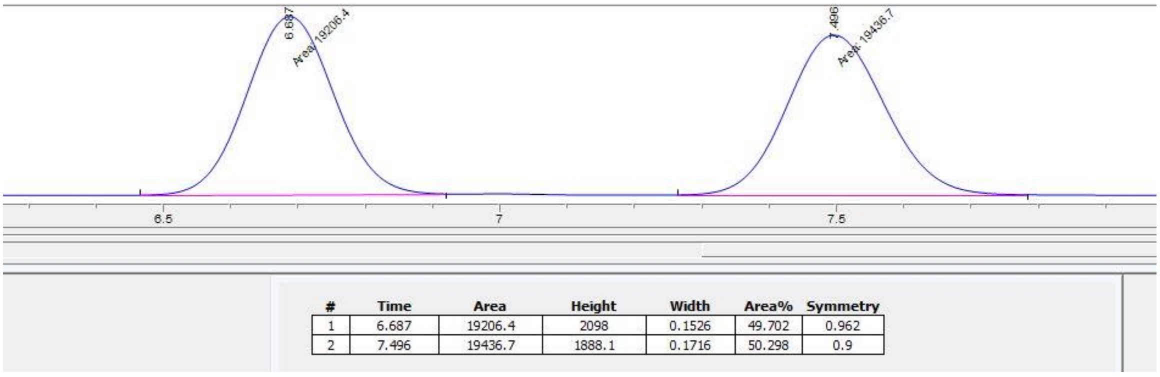

#	Time	Area	Height	Width	Area%	Symmetry
1	6.687	19206.4	2098	0.1526	49.702	0.962
2	7.496	19436.7	1888.1	0.1716	50.298	0.9

SFC analysis of the enantioenriched material, prepared using (R)-OMe-BIPHEP:

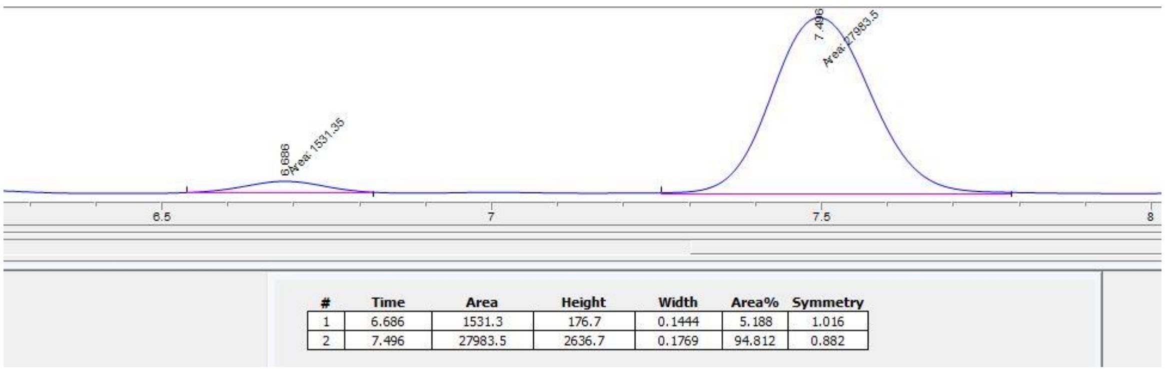

#	Time	Area	Height	Width	Area%	Symmetry
1	6.686	1531.3	176.7	0.1444	5.188	1.016
2	7.496	27983.5	2636.7	0.1769	94.812	0.882

Benzyl (*R*,*Z*)-3-(4-fluorophenyl)-2-((phenylamino)methylene)butanoate (3ag):

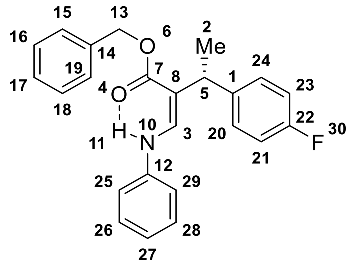

General procedure B: The reaction was carried out with substrate **1a** (25.3 mg, 0.10 mmol, 100 mol%) and 1-fluoro-4-vinylbenzene **1g** (36.0 μL, 0.30 mmol, 300 mol%). Purification of the residue by FCC (hexane/EtOAc 92:8) afforded the title compound (21.0 mg, 56%, >30:1 B:L, *Z/E* > 20:1, e.r. = 95:5) as a pale-yellow oil. ^{1}H NMR analysis of the crude material gave >30:1 B:L and *Z/E* > 20:1. $[\alpha]_D^{25}$ = -57.5 (c = 1.0, $CHCl_3$); **IR (thin film)** ν_{max}/cm^{-1}: 3315 (br), 2963 (s), 1667 (s), 1599 (s), 1177 (s), 693 (s); **^{1}H NMR** (500 MHz, $CDCl_3$) δ 10.07 (d, *J* = 12.5 Hz, 1H, H^{11}), 7.35 – 7.26 (m, 6H, H^3 + ArH), 7.22 – 7.19 (m, 2H, ArH), 7.14 – 7.12 (m, 2H, ArH), 7.03 – 6.94 (m, 5H, H^{25} + H^{27} + H^{29} + ArH), 5.19 – 5.10 (m, 2H, H^{13}), 4.06 (q, *J* = 7.0 Hz, 1H, H^5), 1.49 (d, *J* = 7.0 Hz, 3H, H^2); **^{13}C NMR** (126 MHz, $CDCl_3$) δ 169.4 (C^7), 161.2 (d, *J* = 242.5 Hz, C^{22}), 142.4 (d, *J* = 2.5 Hz, C^1), 141.1 (C^{12}), 141.0 (C^3), 136.4 (C^{14}), 129.7 (C^{26} + C^{28}), 128.8 (d, *J* = 7.5 Hz, C^{20} + C^{24}), 128.4 (C^{16} + C^{18}), 127.9 (C^{17}), 127.6 (C^{15} + C^{19}), 122.2 (C^{27}), 115.3 (C^{25} + C^{29}), 114.9 (d, *J* = 21.3 Hz, C^{21} + C^{22}), 104.1 (C^8), 65.4 (C^{13}), 38.1 (C^5), 21.5 (C^2); ^{19}F NMR (471 MHz, $CDCl_3$) δ -117.9; **HRMS** (ESI): calculated for $C_{24}H_{22}FNO_2Na$ $[M+Na]^+$ requires *m/z* 398.1527, found *m/z* 398.1529; **Chiral SFC:** DAICEL CHIRALCEL SC column (25 cm), CO_2:*i*-PrOH 95:5, 2.0 mL/min, 254 nm, 171 bar. Retention times: 5.6 mins (minor), 5.9 mins (major), e.r. = 95:5.

SFC analysis of the racemate, prepared using rac-BINAP:

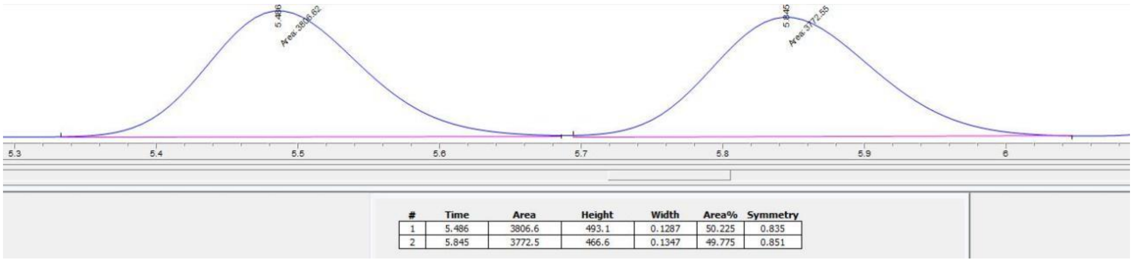

#	Time	Area	Height	Width	Area%	Symmetry
1	5.486	3806.6	493.1	0.1287	50.225	0.835
2	5.845	3772.5	466.6	0.1347	49.775	0.851

SFC analysis of the enantioenriched material, prepared using (R)-OMe-BIPHEP:

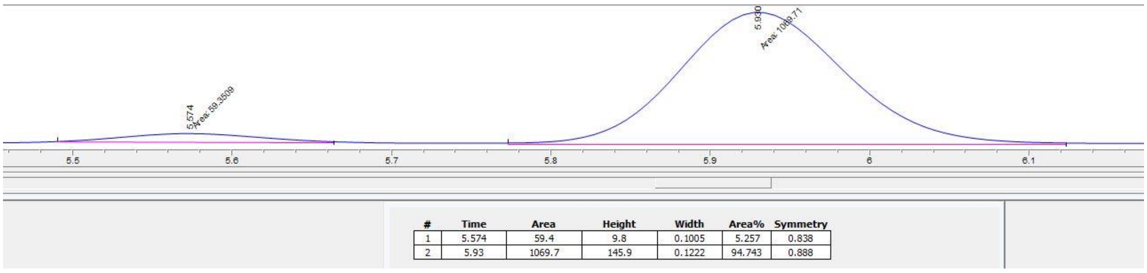

#	Time	Area	Height	Width	Area%	Symmetry
1	5.574	59.4	9.8	0.1005	5.257	0.838
2	5.93	1069.7	145.9	0.1222	94.743	0.888

Benzyl (*R,Z*)-3-(4-bromophenyl)-2-((phenylamino)methylene)butanoate (3ah):

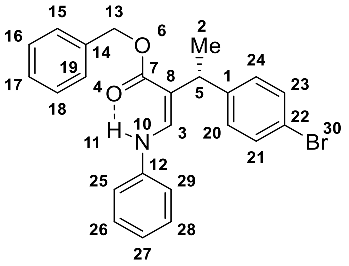

General procedure B: The reaction was carried out with substrate **1a** (25.3 mg, 0.10 mmol, 100 mol%) and 1-bromo-4-vinylbenzene **1h** (41.0 µL, 0.30 mmol, 300 mol%). Purification of the residue by FCC (hexane/EtOAc 92:8) afforded the title compound (18.3 mg, 42%, >30:1 B:L, *Z/E* > 20:1, e.r. = 95:5) as a pale-yellow oil. ^{1}H NMR analysis of the crude material gave >30:1 B:L and *Z/E* > 20:1. $[\boldsymbol{\alpha}]_D^{25}$ = -26.2 (c = 1.0, $CHCl_3$); **IR (thin film)** ν_{max}/cm^{-1}: 3299 (br), 2963 (s), 1669 (s), 1600 (s), 1486 (s), 1008 (s), 822 (s); **^{1}H NMR** (500 MHz, $CDCl_3$) δ 10.07 (d, *J* = 12.5 Hz, 1H, H^{11}), 7.38 (d, *J* = 8.5 Hz, 2H, H^{21} + H^{23}), 7.34 – 7.28 (m, 6H, H^3 + ArH), 7.12 – 7.07 (m, 4H, H^{20} + H^{24} + ArH), 7.03 – 7.00 (m, 1H, H^{27}), 6.95 (d, *J* = 7.5 Hz, 2H, H^{25} + H^{29}), 5.17 – 5.06 (m, 2H, H^{13}), 4.01 (q, *J* = 7.0 Hz, 1H, H^5), 1.47 (d, *J* = 7.0 Hz, 3H, H^2); **^{13}C NMR** (126 MHz, $CDCl_3$) δ 169.3 (C^7), 146.0 (C^1), 141.1 (C^3), 136.4 (C^{14}), 131.2 (C^{21} + C^{23}), 129.7 (C^{26} + C^{28}), 129.1 (C^{20} + C^{24}), 128.4 (C^{16} + C^{18}), 127.9 (C^{17}), 127.6 (C^{15} + C^{19}), 122.3 (C^{27}), 119.4 (C^{22}), 115.4 (C^{25} + C^{29}), 103.5 (C^8), 65.4 (C^{13}), 38.4 (C^5), 21.2 (C^2); **HRMS** (ESl): calculated for $C_{24}H_{22}{}^{79}BrNO_2Na$ $[M+Na]^+$ requires *m/z* 458.0726, found *m/z* 458.0727; **Chiral SFC:** DAICEL CHIRALCEL SC column (25 cm), CO_2:*i*-PrOH 95:5, 2.0 mL/min, 254 nm, 171 bar. Retention times: 9.9 mins (minor), 10.7 mins (major), e.r. = 95:5.

SFC analysis of the racemate, prepared using rac-BINAP:

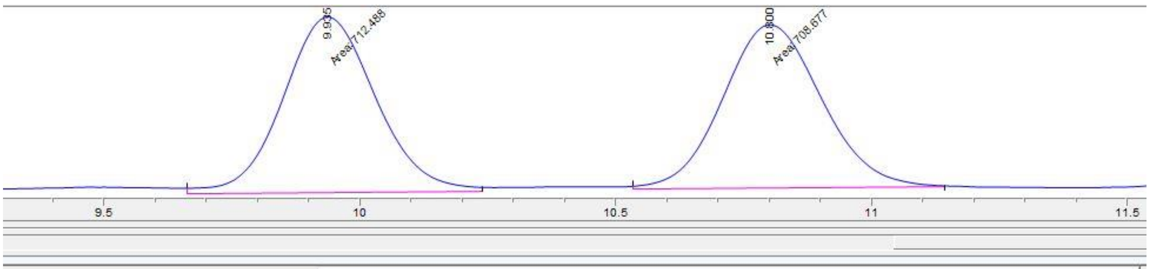

#	Time	Area	Height	Width	Area%	Symmetry
1	9.935	712.5	56.3	0.211	50.134	0.914
2	10.8	708.7	52.4	0.2255	49.866	0.898

SFC analysis of the enantioenriched material, prepared using (R)-OMe-BIPHEP:

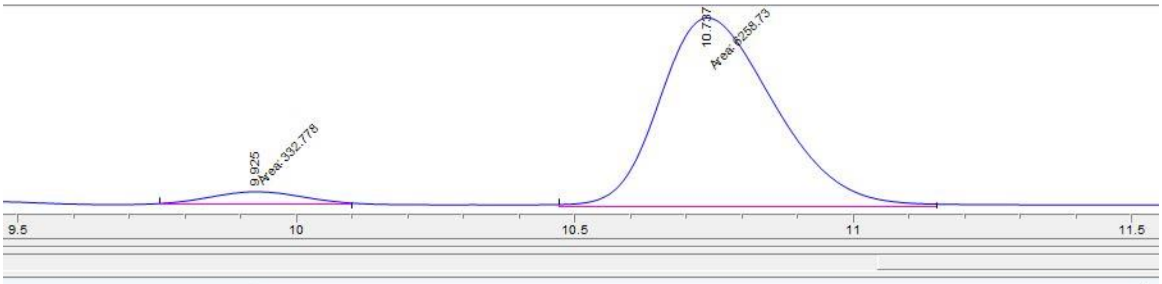

#	Time	Area	Height	Width	Area%	Symmetry
1	9.925	332.8	28.7	0.193	5.049	0.948
2	10.737	6258.7	433.5	0.2406	94.951	0.702

Benzyl (*R*,*Z*)-2-((phenylamino)methylene)-3-(4-(4,4,5,5-tetramethyl-1,3,2-dioxaborolan-2-yl)phenyl)butanoate (3ai):

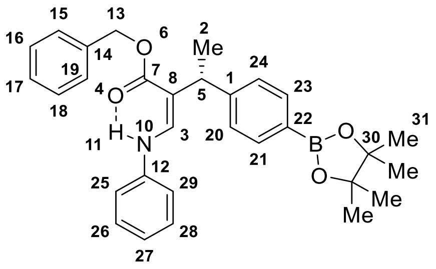

General procedure B: The reaction was carried out with substrate **1a** (25.3 mg, 0.10 mmol, 100 mol%) and 4,4,5,5-tetramethyl-2-(4-vinylphenyl)-1,3,2-dioxaborolane **1i** (69.0 mg, 0.30 mmol, 300 mol%). Purification of the residue by FCC (hexane/EtOAc 92:8) afforded the title compound (20.3 mg, 42%, >30:1 B:L, *Z/E* > 20:1, e.r. = 95:5) as a pale-yellow oil. ^{1}H NMR analysis of the crude material gave >30:1 B:L and *Z/E* = 18:1. $[\boldsymbol{\alpha}]_{\boldsymbol{D}}^{\mathbf{25}}$ = -77.8 (c = 1.0, $CHCl_3$); **IR (thin film)** ν_{max}/cm^{-1}: 3305 (br), 2977 (s), 1669 (s), 1600 (s), 1357 (s), 658 (s); **^{1}H NMR** (500 MHz, $CDCl_3$) δ 10.08 (d, *J* = 12.5 Hz, 1H, H^{11}), 7.76 (d, *J* = 8.1 Hz, 2H, H^{21} + H^{23}), 7.33 – 7.27 (m, 8H, H^3 + ArH), 7.12 – 7.10 (m, 2H, ArH), 7.01 – 6.98 (m, 1H, H^{27}), 6.94 (d, *J* = 7.5 Hz, 2H, H^{25} + H^{29}), 5.16 – 5.10 (m, 2H, H^{13}), 4.10 (q, *J* = 7.0 Hz, 1H, H^5), 1.50 (d, *J* = 7.0 Hz,

3H, H[2]), 1.38 (s, 12H, H[31]); **^{13}C NMR** (126 MHz, $CDCl_3$) δ 169.5 (C[7]), 150.2 (C[1]), 141.2 (C[3]), 136.5 (C[14]), 134.9 (C[21] + C[23]), 129.7 (C[26] + C[28]), 128.4 (C[16] + C[18]), 127.8 (C[17]), 127.6 (C[15] + C[19]), 126.9 (C[20] + C[24]), 122.1 (C[27]), 115.3 (C[25] + C[29]), 104.0 (C[8]), 83.7 (C[30]), 65.4 (C[13]), 38.8 (C[5]), 24.89 (C[31]), 24.88 (C[31]), 21.3 (C[2]); **HRMS** (ESI): calculated for $C_{30}H_{34}BNO_4Na$ $[M+Na]^+$ requires *m/z* 506.2473, found *m/z* 506.2485; **Chiral SFC:** DAICEL CHIRALCEL SC column (25 cm), CO_2:*i*-PrOH 95:5, 2.0 mL/min, 254 nm, 156 bar. Retention times: 8.9 mins (minor), 10.0 mins (major), e.r. = 92:8.

SFC analysis of the racemate, prepared using rac-BINAP:

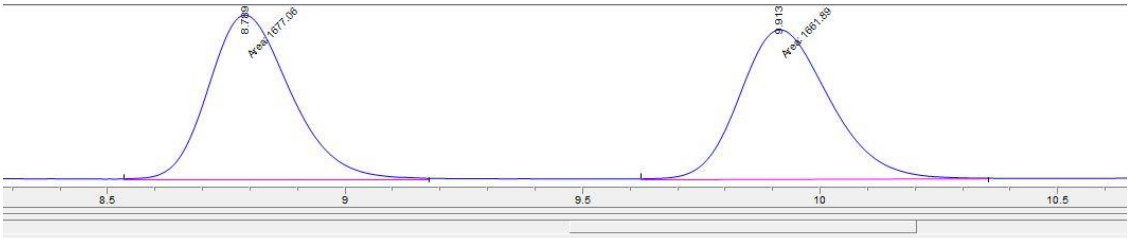

#	Time	Area	Height	Width	Area%	Symmetry
1	8.789	1677.1	137.3	0.2035	50.227	0.777
2	9.913	1661.9	124.7	0.2221	49.773	0.795

SFC analysis of the enantioenriched material, prepared using (R)-OMe-BIPHEP:

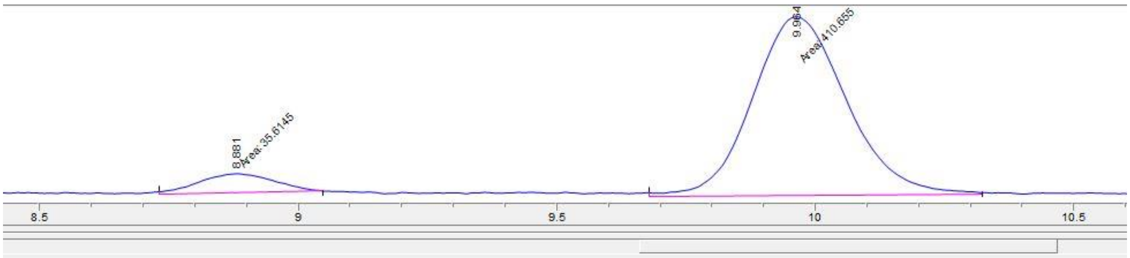

#	Time	Area	Height	Width	Area%	Symmetry
1	8.881	35.6	3.5	0.1706	7.980	1.077
2	9.964	410.7	32.1	0.2134	92.020	0.872

Benzyl (*R*,*Z*)-3-(4-(*N*,*N*-diethylsulfamoyl)phenyl)-2-((phenylamino)methylene)butanoate (3aj):

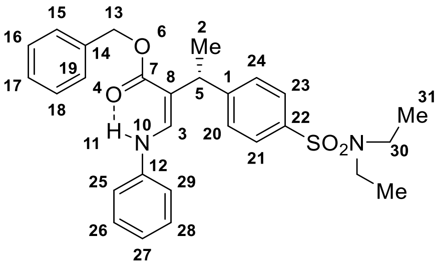

General procedure B: The reaction was carried out with substrate **1a** (25.3 mg, 0.10 mmol, 100 mol%) and *N*,*N*-diethyl-4-vinylbenzenesulfonamide **1j** (71.7 mg, 0.30 mmol, 300 mol%).

Purification of the residue by FCC (hexane/EtOAc 90:10) afforded the title compound (20.7 mg, 42%, >30:1 B:L, *Z/E* > 20:1, e.r. = 90:10) as a pale-yellow oil. ^{1}H NMR analysis of the crude material gave >30:1 B:L and *Z/E* > 20:1. $[\boldsymbol{\alpha}]_D^{25}$ = -74.9 (c = 1.0, $CHCl_3$); **IR (thin film)** ν_{max}/cm^{-1}: 3299 (br), 2926 (s), 1668 (s), 1600 (s), 1180 (s), 697 (s); **^{1}H NMR** (500 MHz, $CDCl_3$) δ 10.10 (d, *J* = 12.5 Hz, 1H, H^{11}), 7.70 (d, *J* = 8.5 Hz, 2H, H^{21} + H^{23}), 7.36 – 7.28 (m, 8H, H^3 + ArH), 7.11 – 7.09 (m, 2H, ArH), 7.04 – 7.01 (m, 1H, H^{27}), 6.96 (d, *J* = 8.0 Hz, 2H, H^{25} + H^{29}), 5.12 – 5.06 (m, 2H, H^{13}), 4.09 (q, *J* = 7.0 Hz, 1H, H^5), 3.24 (q, *J* = 7.0 Hz, 4H, H^{30}), 1.50 (d, *J* = 7.0 Hz, 3H, H^2), 1.14 (t, *J* = 7.0 Hz, 6H, H^{31}); **^{13}C NMR** (126 MHz, $CDCl_3$) δ 169.2 (C^7), 151.8 (C^1), 141.4 (C^3), 141.0 (C^{12}), 137.8 (C^{22}), 136.2 (C^{14}), 129.7 (C^{26} + C^{28}), 128.5 (C^{16} + C^{18}), 128.0 (C^{17}), 127.9 (ArC), 127.7 (ArC), 127.0 (ArC), 122.5 (C^{27}), 115.4 (C^{25} + C^{29}), 103.0 (C^8), 65.6 (C^{13}), 42.1 (C^{30}), 38.8 (C^5), 21.0 (C^{31}), 14.2 (C^{31}); **HRMS** (ESI): calculated for $C_{28}H_{32}N_2O_4SNa$ $[M+Na]^+$ requires *m/z* 515.1975, found *m/z* 515.1981; **Chiral SFC:** DAICEL CHIRALCEL SC column (25 cm), CO_2:*i*-PrOH 80:20, 3.0 mL/min, 254 nm, 185 bar. Retention times: 9.5 mins (minor), 10.0 mins (major), e.r. = 90:10.

SFC analysis of the racemate, prepared using rac-BINAP:

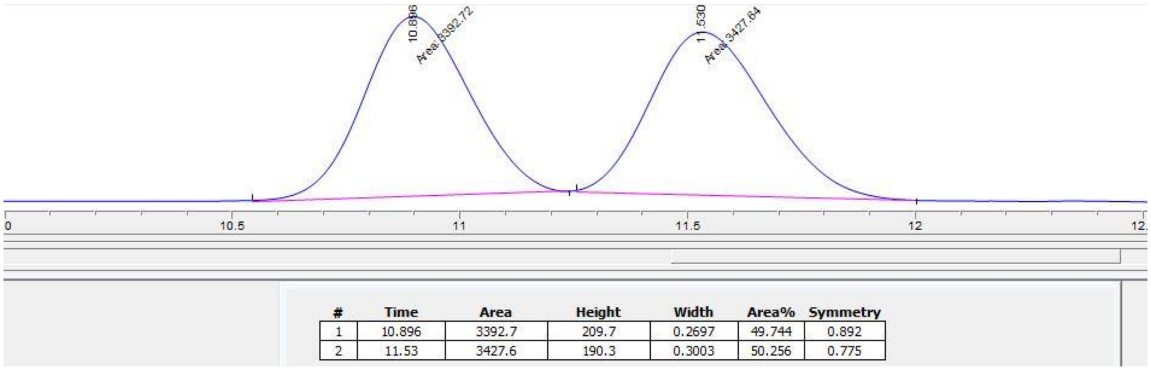

#	Time	Area	Height	Width	Area%	Symmetry
1	10.896	3392.7	209.7	0.2697	49.744	0.892
2	11.53	3427.6	190.3	0.3003	50.256	0.775

SFC analysis of the enantioenriched material, prepared using (R)-OMe-BIPHEP:

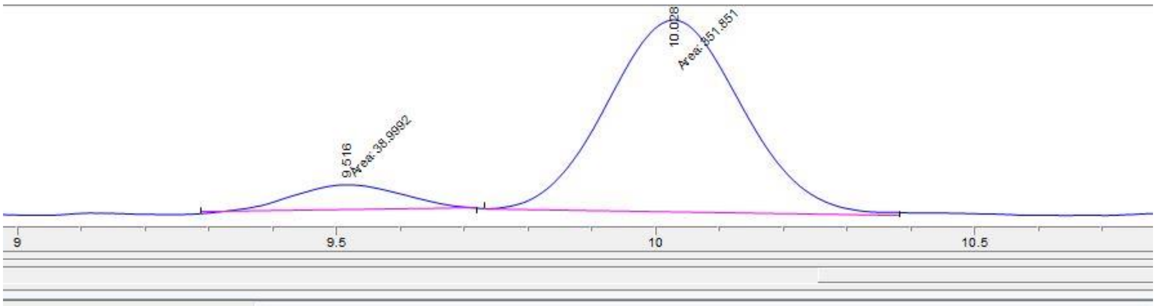

#	Time	Area	Height	Width	Area%	Symmetry
1	9.516	39	3.2	0.2041	9.978	0.995
2	10.028	351.9	23.8	0.2462	90.022	0.956

Benzyl (*R*,*Z*)-3-(3-chlorophenyl)-2-((phenylamino)methylene)butanoate (3ak):

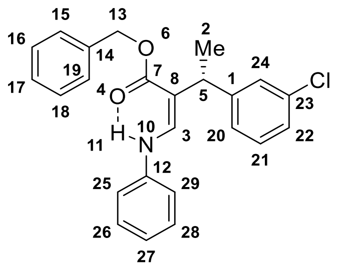

General procedure B: The reaction was carried out with substrate **1a** (25.3 mg, 0.10 mmol, 100 mol%) and 1-chloro-3-vinylbenzene **1k** (37.0 µL, 0.30 mmol, 300 mol%). Purification of the residue by FCC (hexane/EtOAc 92:8) afforded the title compound (26.6 mg, 68%, >30:1 B:L, *Z/E* > 20:1, e.r. = 96:4) as a pale-yellow oil. ^{1}H NMR analysis of the crude material gave >30:1 B:L and *Z/E* > 20:1. $[\alpha]_D^{25}$ = -64.3 (c = 1.0, $CHCl_3$); **IR (thin film)** ν_{max}/cm^{-1}: 3235 (br), 2966 (s), 1668 (s), 1599 (s), 1179 (s), 693 (s); **^{1}H NMR** (500 MHz, $CDCl_3$) δ 10.11 (d, *J* = 12.5 Hz, 1H, H^{11}), 7.35 – 7.31 (m, 6H, H^3 + ArH), 7.24 – 7.18 (m, 3H, ArH), 7.14 – 7.11 (m, 3H, ArH), 7.04 – 7.01 (m, 1H, H^{27}), 6.97 (d, *J* = 8.0 Hz, 2H, H^{25} + H^{29}), 5.17 – 5.08 (m, 2H, H^{13}), 4.04 (q, *J* = 7.0 Hz, 1H, H^5), 1.49 (d, *J* = 7.0 Hz, 3H, H^2); **^{13}C NMR** (126 MHz, $CDCl_3$) δ 169.3 (C^7), 149.1 (C^1), 141.2 (C^3), 141.1 (C^{12}), 136.3 (C^{14}), 134.0 (C^{23}), 129.7 (C^{26} + C^{28}), 129.5 (ArC), 128.5 (C^{16} + C^{18}), 127.9 (C^{17}), 127.7 (C^{15} + C^{19}), 127.5 (ArC), 126.0 (ArC), 125.6 (ArC), 122.3 (C^{27}), 115.4 (C^{25} + C^{29}), 103.4 (C^8), 65.5 (C^{13}), 38.6 (C^5), 21.2 (C^2); **HRMS** (ESl): calculated for $C_{24}H_{22}{}^{35}ClNO_2Na$ $[M+Na]^+$ requires *m/z* 414.1231, found *m/z* 414.1235; **Chiral SFC:** DAICEL CHIRALCEL SC column (25 cm), CO_2:*i*-PrOH 95:5, 2.0 mL/min, 254 nm, 171 bar. Retention times: 7.5 mins (minor), 7.8 mins (major), e.r. = 96:4.

SFC analysis of the racemate, prepared using rac-BINAP:

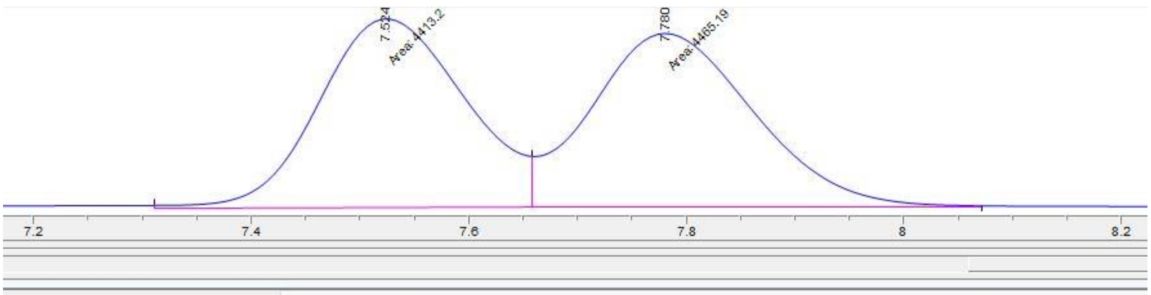

#	Time	Area	Height	Width	Area%	Symmetry
1	7.524	4413.2	465.9	0.1579	49.707	0.845
2	7.78	4465.2	429.1	0.1734	50.293	0.802

SFC analysis of the enantioenriched material, prepared using (R)-OMe-BIPHEP:

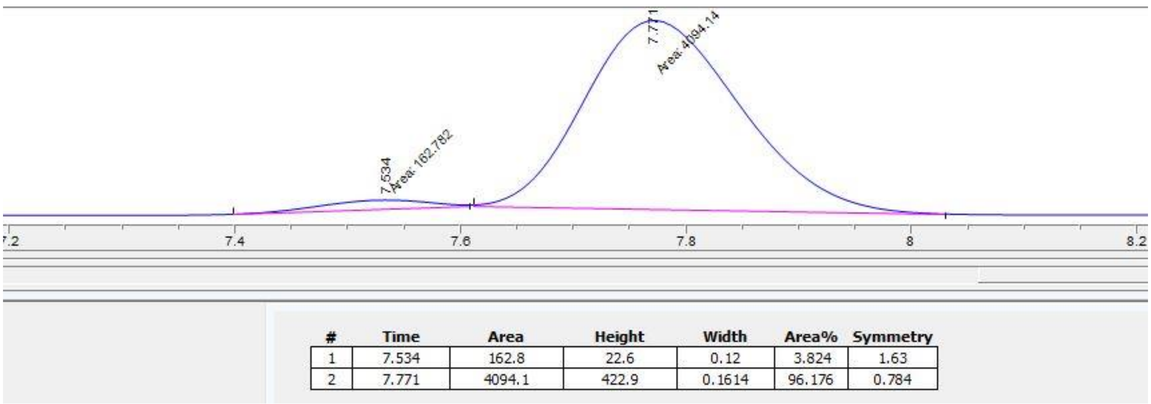

#	Time	Area	Height	Width	Area%	Symmetry
1	7.534	162.8	22.6	0.12	3.824	1.63
2	7.771	4094.1	422.9	0.1614	96.176	0.784

Benzyl (*S*,*Z*)-3-(2-chlorophenyl)-2-((phenylamino)methylene)butanoate (3al):

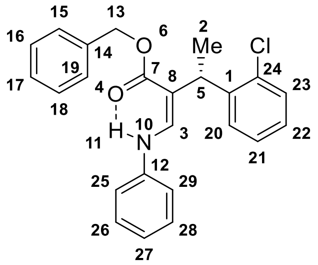

General procedure B: The reaction was carried out with substrate **1a** (25.3 mg, 0.10 mmol, 100 mol%), 1-chloro-2-vinylbenzene **1l** (63.0 µL, 0.50 mmol, 500 mol%), Ir(cod)$_2$BARF (9.54 mg, 7.50 µmol, 7.5 mol%) and (*R*)-OMe-BIPHEP (4.37 mg, 7.50 µmol, 7.5 mol%). Purification of the residue by FCC (hexane/EtOAc 92:8) afforded the title compound (27.8 mg, 71%, >30:1 B:L, *Z/E* > 20:1, e.r. = 93:7) as a pale-yellow oil. ^{1}H NMR analysis of the crude material gave >30:1 B:L and *Z/E* > 20:1. $[\boldsymbol{\alpha}]_{\boldsymbol{D}}^{\mathbf{25}}$ = -36.8 (c = 1.0, CHCl$_3$); **IR (thin film)** ν_{max}/cm^{-1}: 3325 (br), 2964 (s), 1671 (s), 1600 (s), 1179 (s), 750 (s), 695 (s); **^{1}H NMR** (500 MHz, CDCl$_3$) δ 10.12 (d, *J* = 12.5 Hz, 1H, H^{11}), 7.37 – 7.28 (m, 8H, H^3 + ArH), 7.22 – 7.11 (m, 4H, ArH), 7.03 – 7.00 (m, 1H, H^{27}), 6.95 (d, *J* = 8.0 Hz, 2H, H^{25} + H^{29}), 5.21 – 5.08 (m, 2H, H^{13}), 4.56 (q, *J* = 7.0 Hz, 1H, H^5), 1.47 (d, *J* = 7.0 Hz, 3H, H^2); **^{13}C NMR** (126 MHz, CDCl$_3$) δ 169.4 (C^7), 144.1 (C^1), 141.18 (C^3), 141.17 (C^{12}), 136.5 (C^{14}), 133.8 (C^{24}), 129.68 (C^{26} + C^{28}), 129.65 (ArC), 128.4 (C^{16} + C^{18}), 127.8 (ArC), 127.7 (ArC), 127.5 (C^{15} + C^{19}), 127.1 (ArC), 126.8 (ArC), 122.2 (C^{27}), 115.3 (C^{25} + C^{29}), 102.8 (C^8), 65.3 (C^{13}), 35.4 (C^5), 19.9 (C^{31}); **HRMS** (ESI): calculated for $C_{24}H_{22}{}^{35}ClNO_2Na$ [M+Na]$^+$ requires *m/z* 414.1231, found *m/z* 414.1233; **Chiral SFC:** DAICEL CHIRALCEL SB column (25 cm), CO$_2$:*i*-PrOH 95:5, 2.0 mL/min, 254 nm, 170 bar. Retention times: 15.7 mins (major), 16.5 mins (minor), e.r. = 93:7.

SFC analysis of the racemate, prepared using rac-BINAP:

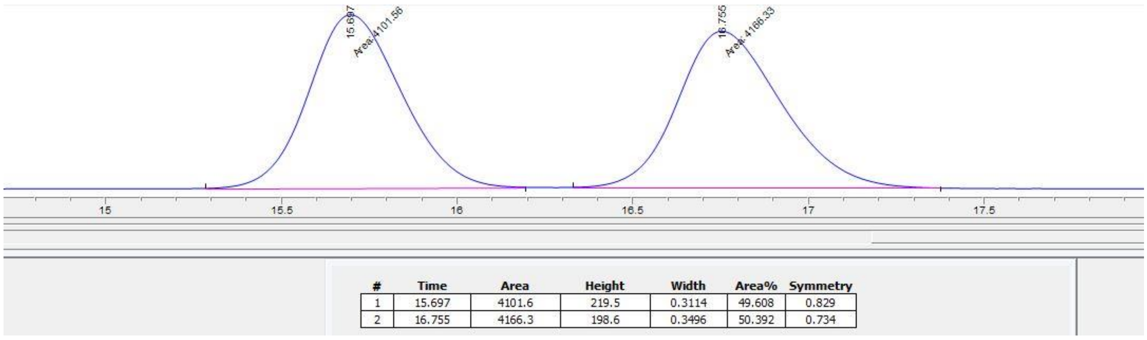

#	Time	Area	Height	Width	Area%	Symmetry
1	15.697	4101.6	219.5	0.3114	49.608	0.829
2	16.755	4166.3	198.6	0.3496	50.392	0.734

SFC analysis of the enantioenriched material, prepared using (R)-OMe-BIPHEP:

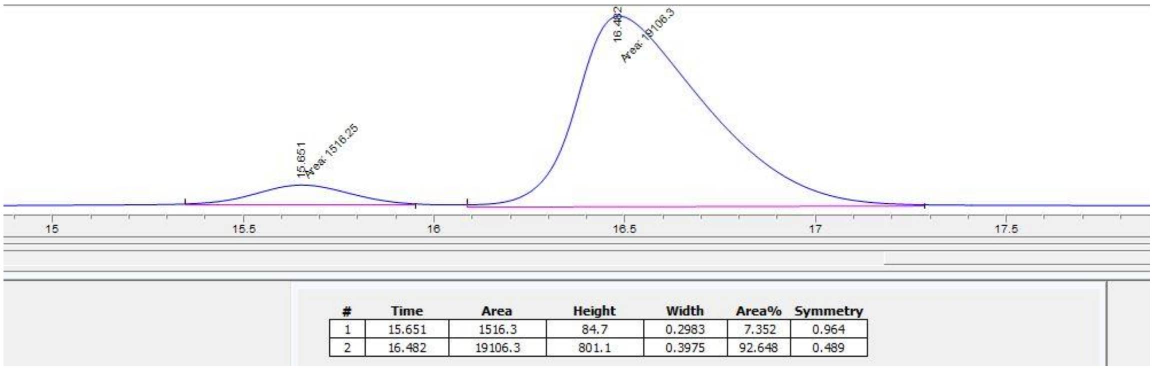

#	Time	Area	Height	Width	Area%	Symmetry
1	15.651	1516.3	84.7	0.2983	7.352	0.964
2	16.482	19106.3	801.1	0.3975	92.648	0.489

Benzyl (*R*,*Z*)-2-((phenylamino)methylene)-3-(*o*-tolyl)butanoate (3am):

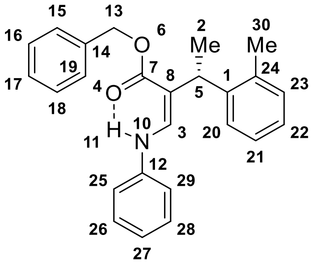

General procedure B: The reaction was carried out with substrate **1a** (25.3 mg, 0.10 mmol, 100 mol%), 1-methyl-2-vinylbenzene **1m** (65.0 µL, 0.50 mmol, 500 mol%), Ir(cod)$_2$BARF (9.54 mg, 7.50 µmol, 7.5 mol%) and (*R*)-OMe-BIPHEP (4.37 mg, 7.50 µmol, 7.5 mol%). Purification of the residue by FCC (hexane/EtOAc 92:8) afforded the title compound (23.0 mg, 62%, >30:1 B:L, *Z/E* > 20:1, e.r. = 95:5) as a pale-yellow solid. ^{1}H NMR analysis of the crude material gave >30:1 B:L and *Z/E* > 20:1. **m.p.** = 79 – 81 °C (hexane/EtOAc); $[\alpha]_D^{25}$ = -61.4 (c = 1.0, CHCl$_3$); **IR (thin film)** ν_{max}/cm^{-1}: 3285 (br), 2969 (s), 1660 (s), 1599 (s), 1194 (s), 695 (s); **^{1}H NMR** (500 MHz, CDCl$_3$) δ 10.03 (d, *J* = 12.5 Hz, 1H, H^{11}), 7.33 – 7.25 (m, 6H, ArH), 7.22 – 7.13 (m, 6H, H^3 + ArH), 6.99 – 6.96 (m, 1H, H^{27}), 6.87 (d, *J* = 7.5 Hz, 2H, H^{25} + H^{29}), 5.22 – 5.12 (m, 2H, H^{13}), 4.29 (q, *J* = 7.0 Hz, 1H, H^5), 2.27 (s, 3H, H^{30}), 1.45 (d, *J* = 7.0 Hz, 3H, H^2); **^{13}C NMR** (126 MHz, CDCl$_3$) δ 169.5 (C^7), 144.5 (C^1), 141.2 (C^{12}), 140.8 (C^3), 136.6 (C^{14}), 135.8 (C^{24}), 130.5 (ArC), 129.6 (C^{26} + C^{28}), 128.4 (C^{16} + C^{18}), 127.8 (C^{17}), 127.6 (C^{15} +

C[19]), 126.0 (ArC), 125.90 (ArC), 125.87 (ArC), 122.0 (C[27]), 115.2 (C[25] + C[29]), 104.0 (C[8]), 65.5 (C[13]), 34.2 (C[5]), 20.8 (C[2]), 19.3 (C[30]); **HRMS** (ESI): calculated for $C_{25}H_{25}NO_2Na$ $[M+Na]^+$ requires *m/z* 394.1778, found *m/z* 394.1778; **Chiral SFC:** DAICEL CHIRALCEL IE column (25 cm), CO_2:*i*-PrOH 95:5, 2.0 mL/min, 254 nm, 172 bar. Retention times: 14.0 mins (minor), 15.2 mins (major), e.r. = 95:5.

SFC analysis of the racemate, prepared using rac-BINAP:

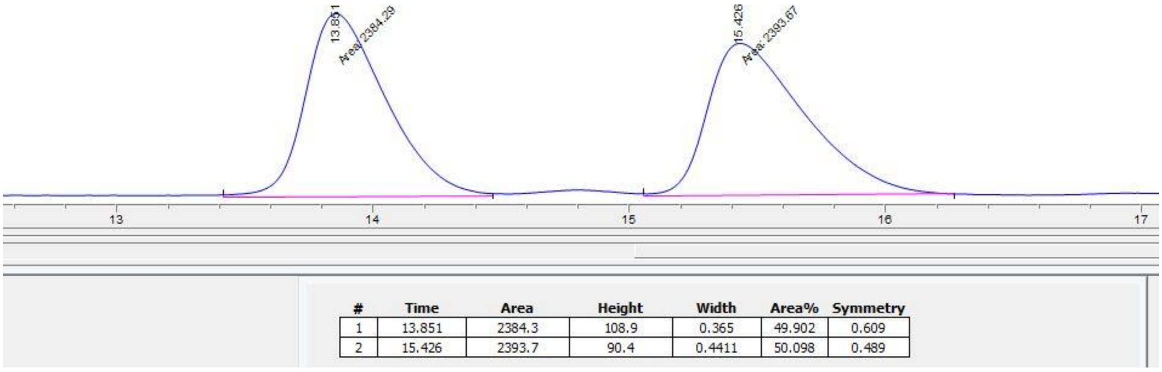

#	Time	Area	Height	Width	Area%	Symmetry
1	13.851	2384.3	108.9	0.365	49.902	0.609
2	15.426	2393.7	90.4	0.4411	50.098	0.489

SFC analysis of the enantioenriched material, prepared using (R)-OMe-BIPHEP:

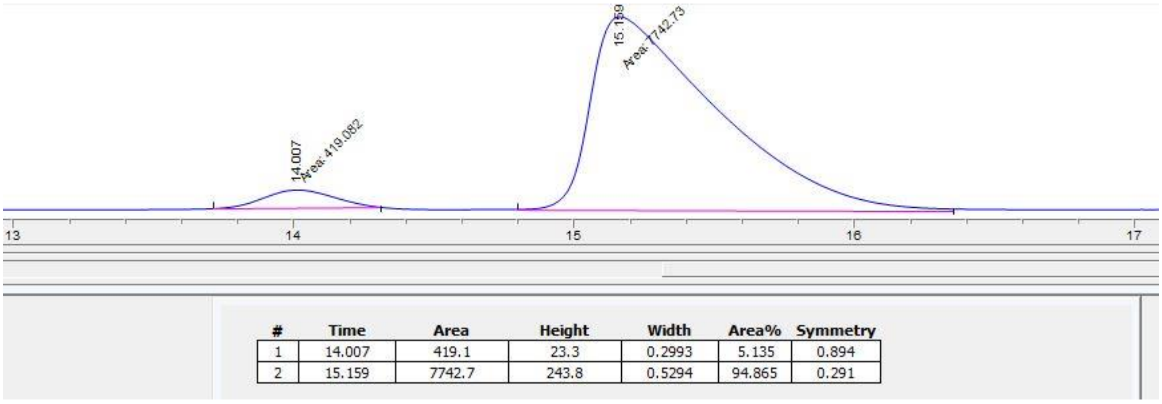

#	Time	Area	Height	Width	Area%	Symmetry
1	14.007	419.1	23.3	0.2993	5.135	0.894
2	15.159	7742.7	243.8	0.5294	94.865	0.291

Benzyl (*S*,*Z*)-3-(perfluorophenyl)-2-((phenylamino)methylene)butanoate (3an):

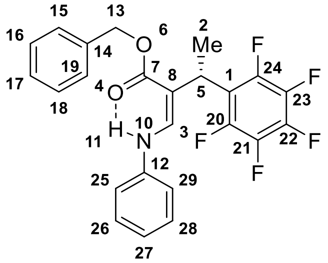

General procedure B: The reaction was carried out with substrate **1a** (25.3 mg, 0.10 mmol, 100 mol%), 1,2,3,4,5-pentafluoro-6-vinylbenzene **1n** (69.0 µL, 0.50 mmol, 500 mol%), $Ir(cod)_2BARF$ (9.54 mg, 7.50 µmol, 7.5 mol%) and (*R*)-OMe-BIPHEP (4.37 mg, 7.50 µmol, 7.5 mol%). Purification of the residue by FCC (hexane/EtOAc 92:8) afforded the title

compound (31.3 mg, 70%, >30:1 B:L, *Z/E* > 20:1, e.r. = 95:5) as a pale-yellow solid. ^{1}H NMR analysis of the crude material gave >30:1 B:L and *Z/E* > 20:1. **m.p.** = 91 – 93 °C (hexane/EtOAc); $[\boldsymbol{\alpha}]_{\boldsymbol{D}}^{\mathbf{25}}$ = -181.9 (c = 1.0, $CHCl_3$); **IR (thin film)** ν_{max}/cm^{-1}: 3298 (br), 2934 (s), 1664 (s), 1497 (s), 1195 (s), 962 (s), 696 (s); **^{1}H NMR** (500 MHz, $CDCl_3$) δ 10.16 (d, *J* = 12.5 Hz, 1H, H^{11}), 7.52 (d, *J* = 12.5 Hz, 1H, H^{3}), 7.37 – 7.34 (m, 5H, ArH), 7.22 – 7.21 (m, 2H, ArH), 7.07 – 7.02 (m, 3H, H^{27} + H^{25} + H^{29}), 5.26 (d, *J* = 12.0 Hz, 1H, H^{13}), 5.02 (d, *J* = 12.0 Hz, 1H, H^{13}), 4.41 (q, *J* = 7.5 Hz, 1H, H^{5}), 1.56 (d, *J* = 7.0 Hz, 3H, H^{2}); **^{13}C NMR** (126 MHz, $CDCl_3$) δ 168.8 (C^{7}), 144.0 (C^{1}), 142.1 (C^{3}), 140.9 (C^{12}), 136.0 (C^{14}), 129.7 (C^{26} + C^{28}), 128.5 (C^{16} + C^{18}), 128.3 (C^{15} + C^{19}), 128.2 (C^{17}), 122.8 (C^{27}), 119.7 (ArC), 115.8 (C^{25} + C^{29}), 99.4 (C^{8}), 65.6 (C^{13}), 28.7 (C^{5}), 17.7 (C^{2}); ^{19}F NMR (471 MHz, $CDCl_3$) δ -144.0, -158.8, -163.3; **HRMS** (ESI): calculated for $C_{24}H_{19}NO_2F_5$ $[M+H]^+$ requires *m/z* 448.1330, found *m/z* 448.1326; **Chiral SFC:** DAICEL CHIRALCEL SB column (25 cm), CO_2:*i*-PrOH 95:5, 2.0 mL/min, 254 nm, 145 bar. Retention times: 5.5 mins (major), 6.2 mins (minor), e.r. = 95:5.

SFC analysis of the racemate, prepared using rac-BINAP:

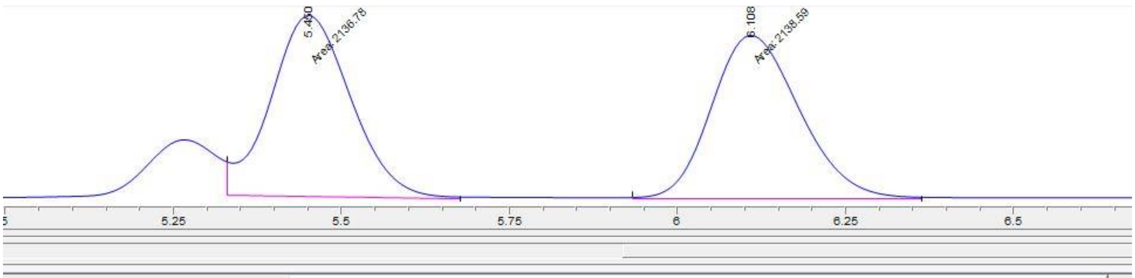

#	Time	Area	Height	Width	Area%	Symmetry
1	5.45	2136.8	257.9	0.1381	49.979	0.863
2	6.108	2138.6	233.1	0.1529	50.021	0.791

SFC analysis of the enantioenriched material, prepared using (R)-OMe-BIPHEP:

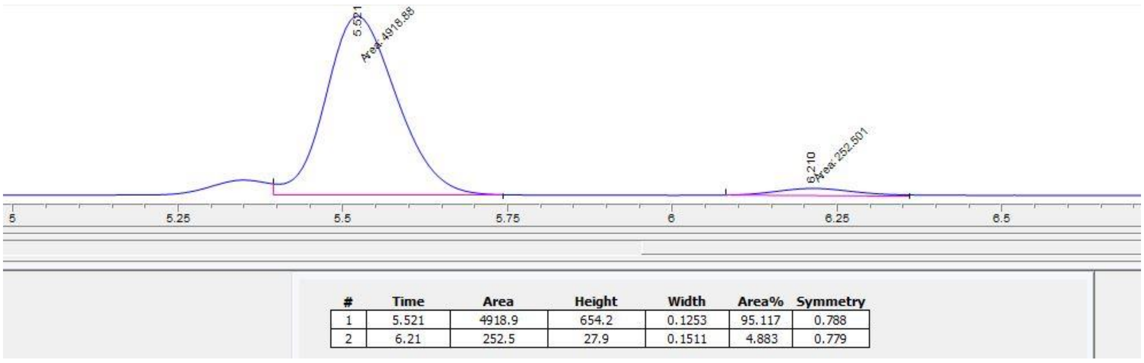

#	Time	Area	Height	Width	Area%	Symmetry
1	5.521	4918.9	654.2	0.1253	95.117	0.788
2	6.21	252.5	27.9	0.1511	4.883	0.779

Benzyl (*R*,*Z*)-2-((phenylamino)methylene)-3-(1-tosyl-1*H*-indol-3-yl)butanoate (3ao):

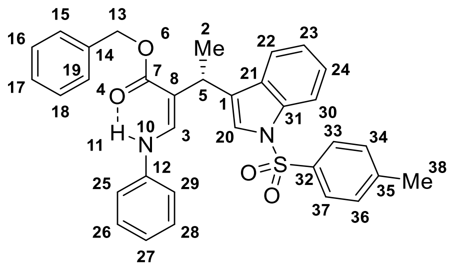

General procedure B: The reaction was carried out with substrate **1a** (25.3 mg, 0.10 mmol, 100 mol%) and 1-tosyl-3-vinyl-1*H*-indole **1o** (89.1 µL, 0.30 mmol, 300 mol%). Purification of the residue by FCC (hexane/EtOAc 90:10) afforded the title compound (35.2 mg, 64%, >30:1 B:L, *Z/E* > 20:1, e.r. = 93:7) as a pale-yellow oil. ^{1}H NMR analysis of the crude material gave >30:1 B:L and *Z/E* > 20:1. $[\boldsymbol{\alpha}]_{\boldsymbol{D}}^{\mathbf{25}}$ = 10.9 (c = 1.0, $CHCl_3$); **IR (thin film)** ν_{max}/cm^{-1}: 3365 (br), 3052 (s), 1669 (s), 1599 (s), 1172 (s), 734 (s), 668 (s); **^{1}H NMR** (500 MHz, $CDCl_3$) δ 9.96 (d, *J* = 12.5 Hz, 1H, H^{11}), 8.02 (d, *J* = 8.5 Hz, 1H, H^{22}), 7.75 (d, *J* = 8.0 Hz, 2H, H^{33} + H^{37}), 7.45 (s, 1H, H^{20}), 7.40 (d, *J* = 8.0 Hz, 1H, ArH), 7.33 – 7.15 (m, 11H, H^{34} + H^{36} + ArH), 7.00 – 6.94 (m, 2H, ArH, H^3 + H^{27}), 6.68 (d, *J* = 8.5 Hz, 2H, H^{25} + H^{29}), 5.24 (s, 2H, H^{13}), 4.28 (q, *J* = 7.0 Hz, 1H, H^5), 2.28 (s, 3H, H^{38}), 1.58 (d, *J* = 7.0 Hz, 3H, H^2); **^{13}C NMR** (126 MHz, $CDCl_3$) δ 169.3 (C^7), 144.7 (C^{32}), 141.7 (C^3), 140.8 (C^{12}), 136.5 (C^{14}), 135.7 (ArC), 135.3 (ArC), 130.2 (ArC), 129.8 (C^{26} + C^{28}), 129.6 (ArC), 128.5 (C^{16} + C^{18}), 128.0 (ArC), 127.9 (ArC), 127.6 (ArC), 126.6 (C^{33} + C^{37}), 124.8 (ArC), 123.2 (ArC), 122.9 (ArC), 122.3 (C^{27}), 120.5 (ArC), 115.4 (C^{25} + C^{29}), 113.8 (C^{22}), 102.4 (C^8), 65.5 (C^{13}), 30.0 (C^5), 21.5 (C^{38}), 21.1 (C^2); **HRMS** (ESl): calculated for $C_{33}H_{30}N_2O_4SNa$ $[M+Na]^+$ requires *m/z* 573.1818, found *m/z* 573.1815; **Chiral SFC:** DAICEL CHIRALCEL SB column (25 cm), CO_2:*i*-PrOH 90:10, 2.0 mL/min, 254 nm, 145 bar. Retention times: 31.5 mins (minor), 33.1 mins (major), e.r. = 93:7.

SFC analysis of the racemate, prepared using rac-BINAP:

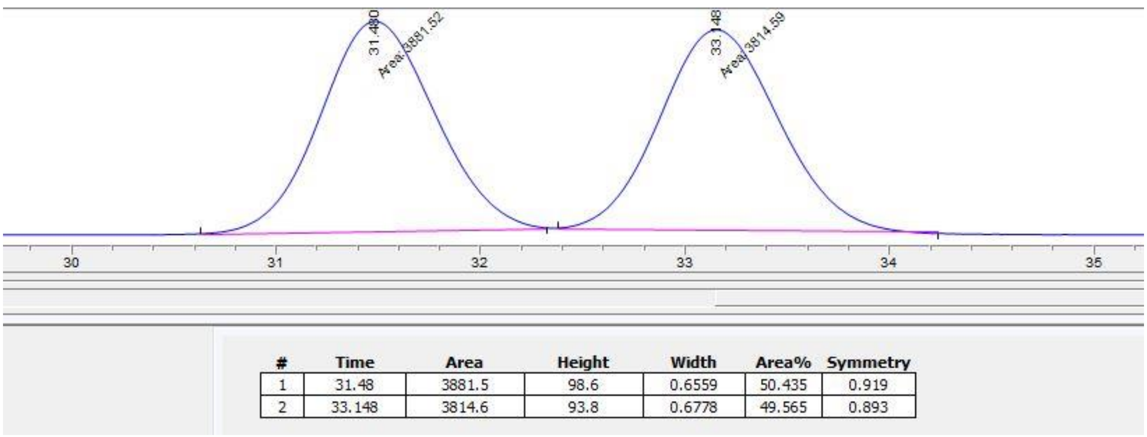

#	Time	Area	Height	Width	Area%	Symmetry
1	31.48	3881.5	98.6	0.6559	50.435	0.919
2	33.148	3814.6	93.8	0.6778	49.565	0.893

SFC analysis of the enantioenriched material, prepared using (R)-OMe-BIPHEP:

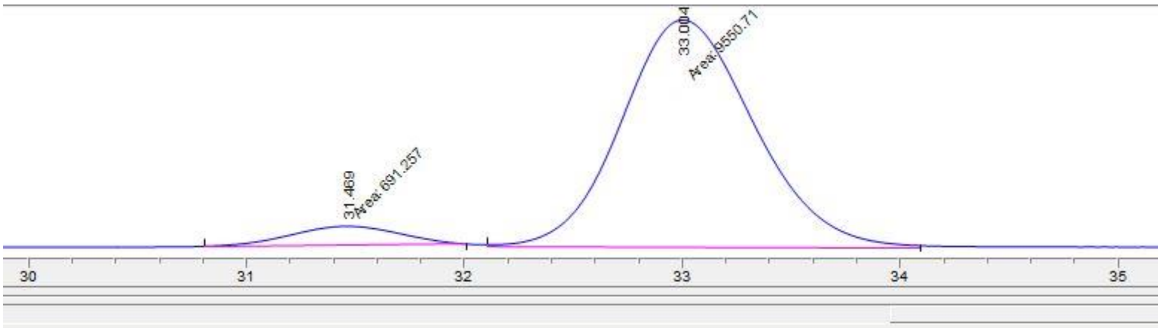

#	Time	Area	Height	Width	Area%	Symmetry
1	31.469	691.3	19.1	0.6027	6.749	1.164
2	33.004	9550.7	222.9	0.7142	93.251	0.845

Benzyl (*R,Z*)-3-(benzofuran-5-yl)-2-((phenylamino)methylene)butanoate (3ap):

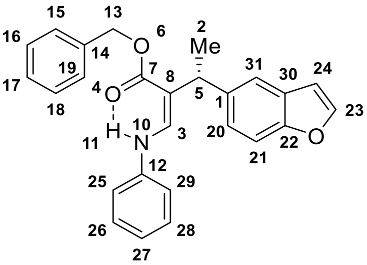

General procedure B: The reaction was carried out with substrate **1a** (25.3 mg, 0.10 mmol, 100 mol%) and 5-vinylbenzofuran **1p** (43.3 mg, 0.30 mmol, 300 mol%). Purification of the residue by FCC (hexane/EtOAc 92:8) afforded the title compound (21.1 mg, 53%, >30:1 B:L, *Z/E* > 20:1, e.r. = 95:5) as a pale-yellow oil. ^{1}H NMR analysis of the crude material gave >30:1 B:L and *Z/E* > 20:1. $[\alpha]_D^{25}$ = -63.0 (c = 1.0, $CHCl_3$); **IR (thin film)** ν_{max}/cm^{-1}: 3298 (br), 2962 (s), 1666 (s), 1599 (s), 1190 (s), 735 (s), 693 (s); **^{1}H NMR** (500 MHz, $CDCl_3$) δ 10.09 (d, *J* = 12.5 Hz, 1H, H^{11}), 7.64 (d, *J* = 2.0 Hz, 1H, H^{31}), 7.47 (d, *J* = 2.0 Hz, 1H, H^{24}), 7.43 (d, *J* = 8.5 Hz, 1H, ArH), 7.34 – 7.20 (m, 7H, H^3 + ArH), 7.08 – 7.07 (m, 2H, ArH), 7.02 – 6.99 (m, 1H, H^{27}), 6.95 (d, *J* = 8.0 Hz, 2H, H^{25} + H^{29}), 6.72 (d, *J* = 2.0 Hz, 1H, H^{23}), 5.19 – 5.09 (m, 2H, H^{13}), 4.20 (q, *J* = 7.0 Hz, 1H, H^5), 1.55 (d, *J* = 7.0 Hz, 3H, H^2); **^{13}C NMR** (126 MHz, $CDCl_3$) δ 169.5 (C^7), 153.6 (C^{22}), 145.1 (C^{23}), 141.4 (ArC), 141.2 (C^1), 141.0 (C^{12}), 136.5 (C^{14}), 129.7 (C^{26} + C^{28}), 128.3 (C^{16} + C^{18}), 127.7 (ArC), 127.6 (C^{15} + C^{19}), 127.3 (ArC), 124.2 (ArC), 122.1 (C^{27}), 119.4 (ArC), 115.3 (C^{25} + C^{29}), 110.9 (ArC), 106.6 (C^{24}), 104.7 (C^8), 65.4 (C^{13}), 38.6 (C^5), 21.9 (C^2); **HRMS** (ESl): calculated for $C_{26}H_{23}NO_3Na$ $[M+Na]^+$ requires *m/z* 420.1570, found *m/z* 420.1569; **Chiral SFC:** DAICEL CHIRALCEL SC column (25 cm), CO_2:*i*-PrOH 95:5, 2.0 mL/min, 254 nm, 173 bar. Retention times: 10.2 mins (minor), 11.1 mins (major), e.r. = 95:5.

SFC analysis of the racemate, prepared using rac-BINAP:

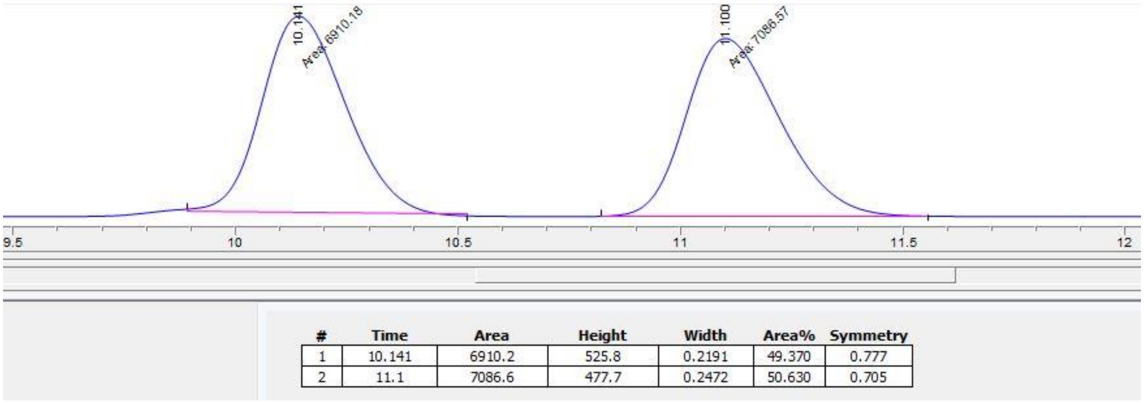

#	Time	Area	Height	Width	Area%	Symmetry
1	10.141	6910.2	525.8	0.2191	49.370	0.777
2	11.1	7086.6	477.7	0.2472	50.630	0.705

SFC analysis of the enantioenriched material, prepared using (R)-OMe-BIPHEP:

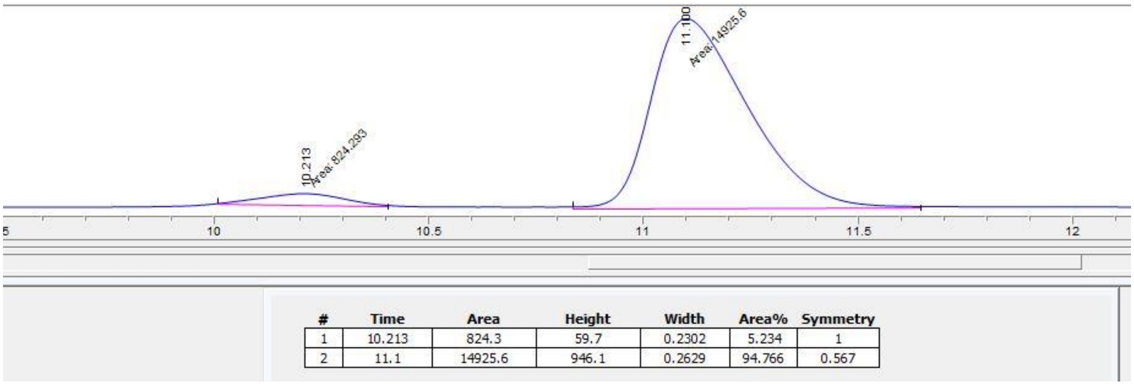

#	Time	Area	Height	Width	Area%	Symmetry
1	10.213	824.3	59.7	0.2302	5.234	1
2	11.1	14925.6	946.1	0.2629	94.766	0.567

Compound (3aq):

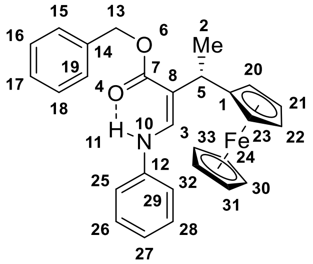

General procedure B: The reaction was carried out with substrate **1a** (25.3 mg, 0.10 mmol, 100 mol%) and vinylferrocene **1q** (63.6 mg, 0.30 mmol, 300 mol%). Purification of the residue by FCC (hexane/EtOAc 92:8) afforded the title compound (34.9 mg, 75%, >30:1 B:L, *Z/E* > 20:1, e.r. = 80:20) as a brown solid. ^{1}H NMR analysis of the crude material gave >30:1 B:L and *Z/E* > 20:1. **m.p.** = 66 – 68 °C (hexane/EtOAc); $[\alpha]_D^{25}$ = -51.0 (c = 1.0, $CHCl_3$); **IR (thin film)** ν_{max}/cm^{-1}: 3092 (br), 2928 (s), 1665 (s), 1599 (s), 1189 (s), 816 (s), 696 (s); **^{1}H NMR** (500 MHz, $CDCl_3$) δ 9.83 (d, *J* = 12.5 Hz, 1H, H^{11}), 7.47 – 7.36 (m, 5H, ArH), 7.28 – 7.23 (m, 2H, H^{26} + H^{28}), 6.98 – 6.93 (m, 2H, H^3 + H^{27}), 6.77 (d, *J* = 7.5 Hz, 2H, H^{25} + H^{29}), 5.34 – 5.24 (m, 2H, H^{13}), 4.18 – 4.10 (m, 9H, H^{20-23} + H^{30-33}), 3.89 (q, *J* = 7.0 Hz, 1H, H^5), 1.53 (d, *J* = 7.0

Hz, 3H, H^2); **^{13}C NMR** (126 MHz, $CDCl_3$) δ 169.3 (C^7), 141.6 (C^{12}), 141.1 (C^3), 136.8 (C^{14}), 129.6 (C^{26} + C^{28}), 128.6 (C^{16} + C^{18}), 128.0 (C^{17}), 127.9 (C^{15} + C^{19}), 121.9 (C^{27}), 115.1 (C^{25} + C^{29}), 106.3 (C^8), 93.9 (C^1), 68.6 (C$^{21-23}$ or C$^{30-33}$), 68.1 (C$^{21-23}$ or C$^{30-33}$), 67.7 (C$^{21-23}$ or C$^{30-33}$), 66.9 (C$^{21-23}$ or C$^{30-33}$), 66.6 (C$^{21-23}$ or C$^{30-33}$), 65.4 (C^{13}), 33.2 (C^5), 21.4 (C^2); **HRMS** (ESI): calculated for $C_{28}H_{28}NO_2Fe$ $[M+H]^+$ requires *m/z* 466.1464, found *m/z* 466.1462; **Chiral SFC:** DAICEL CHIRALCEL SC column (25 cm), CO_2:*i*-PrOH 90:10, 2.0 mL/min, 254 nm, 172 bar. Retention times: 11.3 mins (minor), 11.8 mins (major), e.r. = 80:20.

SFC analysis of the racemates, prepared using rac-BINAP:

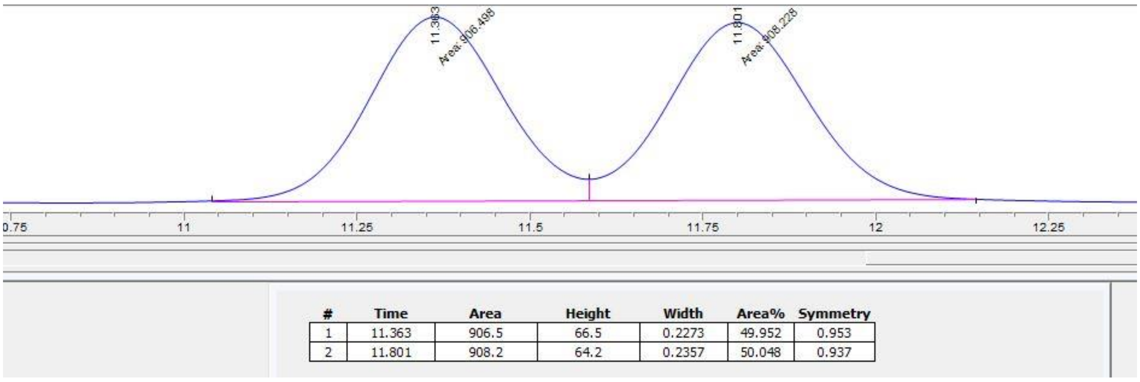

#	Time	Area	Height	Width	Area%	Symmetry
1	11.363	906.5	66.5	0.2273	49.952	0.953
2	11.801	908.2	64.2	0.2357	50.048	0.937

SFC analysis of the enantioenriched material, prepared using (R)-OMe-BIPHEP:

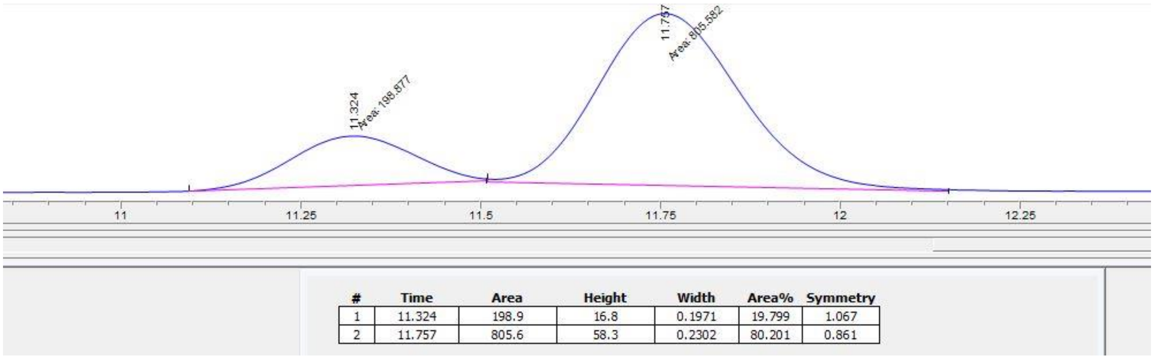

#	Time	Area	Height	Width	Area%	Symmetry
1	11.324	198.9	16.8	0.1971	19.799	1.067
2	11.757	805.6	58.3	0.2302	80.201	0.861

Benzyl (*R*,*Z*)-3-(naphthalen-2-yl)-2-((phenylamino)methylene)butanoate (3ar):

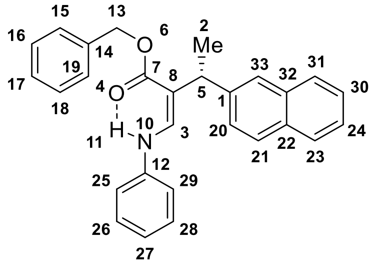

General procedure B: The reaction was carried out with substrate **1a** (25.3 mg, 0.10 mmol, 100 mol%) and 2-vinylnaphthalene **1r** (46.2 mg, 0.30 mmol, 300 mol%). Purification of the

residue by FCC (hexane/EtOAc 92:8) afforded the title compound (37.1 mg, 91%, >30:1 B:L, *Z/E* > 20:1, e.r. = 96:4) as a colorless solid. ^{1}H NMR analysis of the crude material gave >30:1 B:L and *Z/E* > 20:1. **m.p.** = 51 – 53 °C (hexane/EtOAc); $[\boldsymbol{\alpha}]_{\boldsymbol{D}}^{\boldsymbol{25}}$ = -86.2 (c = 1.0, $CHCl_3$); **IR (thin film)** ν_{max}/cm^{-1}: 3301 (br), 2951 (s), 1676 (s), 1599 (s), 1139 (s), 689 (s); **^{1}H NMR** (500 MHz, $CDCl_3$) δ 10.13 (d, *J* = 12.5 Hz, 1H, H^{11}), 7.86 – 7.84 (m, 1H, ArH), 7.79 – 7.77 (m, 2H, ArH), 7.69 (s, 1H, H^{33}), 7.51 – 7.23 (m, 7H, H^3 + ArH), 7.18 – 7.15 (m, 2H, H^{16} + H^{18}), 7.05 (d, *J* = 7.0 Hz, 2H, H^{15} + H^{19}), 7.02 – 6.99 (m, 1H, H^{27}), 6.95 (d, *J* = 7.5 Hz, 2H, H^{25} + H^{29}), 5.17 – 5.09 (m, 2H, H^{13}), 4.26 (q, *J* = 7.0 Hz, 1H, H^5), 1.60 (d, *J* = 7.0 Hz, 3H, H^2); **^{13}C NMR** (126 MHz, $CDCl_3$) δ 169.6 (C^7), 144.2 (C^1), 141.3 (C^{12}), 141.2 (C^3), 136.4 (C^{14}), 133.6 (C^{32}), 132.2 (C^{22}), 129.7 (C^{26} + C^{28}), 128.3 (C^{16} + C^{18}), 127.80 (ArC), 127.75 (ArC), 127.7 (ArC), 127.6 (C^{15} + C^{19}), 126.7 (ArC), 125.8 (ArC), 125.24 (ArC), 125.20 (ArC), 122.2 (C^{27}), 115.4 (C^{25} + C^{29}), 104.1 (C^8), 65.4 (C^{13}), 38.8 (C^5), 21.3 (C^2); **HRMS** (ESI): calculated for $C_{28}H_{25}NO_2Na$ $[M+Na]^+$ requires *m/z* 430.1778, found *m/z* 430.1780; **Chiral SFC:** DAICEL CHIRALCEL SC column (25 cm), CO_2:*i*-PrOH 95:5, 2.0 mL/min, 254 nm, 170 bar. Retention times: 15.9 mins (minor), 17.1 mins (major), e.r. = 96:4.

SFC analysis of the racemate, prepared using rac-BINAP:

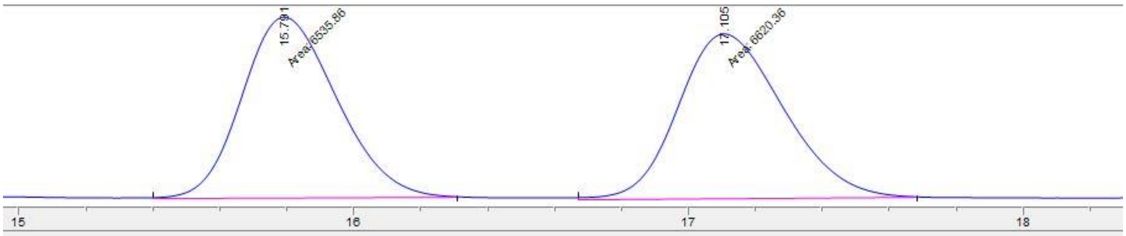

#	Time	Area	Height	Width	Area%	Symmetry
1	15.791	6535.9	328	0.3322	49.679	0.791
2	17.105	6620.4	298.9	0.3691	50.321	0.765

SFC analysis of the enantioenriched material, prepared using (R)-OMe-BIPHEP:

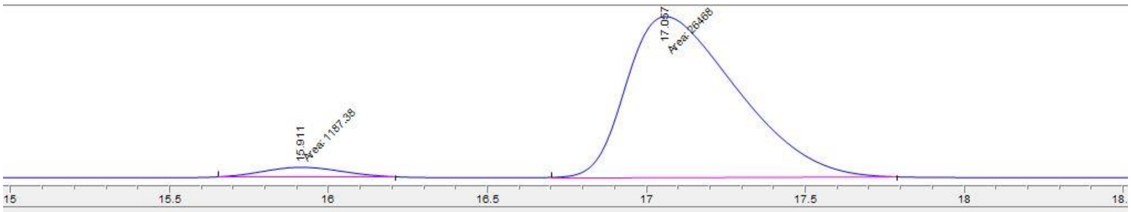

#	Time	Area	Height	Width	Area%	Symmetry
1	15.911	1187.4	67.4	0.2938	4.293	0.943
2	17.057	26468	1088.3	0.4054	95.707	0.534

4-Bromobenzyl (*S*,*Z*)-2-(((4-methoxyphenyl)amino)methylene)-3-(perfluorophenyl)butanoate (3ln):

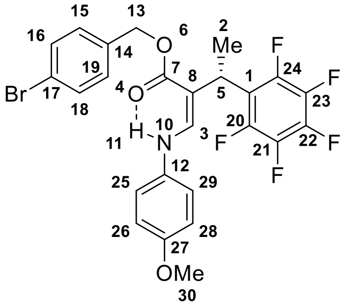

General procedure B: The reaction was carried out with substrate **1l** (36.1 mg, 0.10 mmol, 100 mol%), 1,2,3,4,5-pentafluoro-6-vinylbenzene **1n** (69.0 µL, 0.50 mmol, 500 mol%), Ir(cod)$_2$BARF (9.54 mg, 0.0075 mmol, 7.5 mol%) and (*R*)-OMe-BIPHEP (4.37 mg, 0.0075 mmol, 7.5 mol%). Purification of the residue by FCC (hexane/EtOAc 90:10) afforded the title compound (35.0 mg, 63%, >30:1 B:L, *Z/E* > 20:1, e.r. = 96:4) as a pale-yellow solid. ^{1}H NMR analysis of the crude material gave >30:1 B:L and *Z/E* > 20:1. **m.p.** = 113 – 115 °C (hexane/EtOAc); $[\alpha]_D^{25}$ = -195.1 (c = 1.0, $CHCl_3$); **IR (thin film)** ν_{max}/cm^{-1}: 3299 (br), 2927 (s), 1662 (s), 1193 (s), 695 (s); **^{1}H NMR** (500 MHz, $CDCl_3$) δ 10.05 (d, *J* = 12.5 Hz, 1H, H^{11}), 7.47 (d, *J* = 8.5 Hz, 2H, H^{16} + H^{18}), 7.43 (d, *J* = 12.5 Hz, 1H, H^3), 7.10 (d, *J* = 8.5 Hz, 2H, H^{15} + H^{19}), 6.98 (d, *J* = 9.0 Hz, 2H, H^{25} + H^{29}), 6.91 (d, *J* = 9.0 Hz, 2H, H^{26} + H^{28}), 5.15 – 4.98 (m, 2H, H^{13}), 4.39 (q, *J* = 7.5 Hz, 1H, H^5), 3.82 (s, 3H, H^{30}), 1.55 (d, *J* = 7.5 Hz, 3H, H^2); **^{13}C NMR** (126 MHz, $CDCl_3$) δ 168.8 (C^7), 155.8 (C^{27}), 144.0 (C^1), 143.7 (C^3), 138.3 (C^{12}), 135.2 (ArC), 134.6 (ArC), 131.6 (C^{16} + C^{18}), 129.9 (C^{15} + C^{19}), 122.3 (C^{17}), 117.6 (C^{25} + C^{29}), 115.0 (C^{26} + C^{28}), 97.9 (C^8), 64.7 (C^{13}), 55.6 (C^{30}), 28.5 (C^5), 17.8 (C^2); ^{19}F NMR (471 MHz, $CDCl_3$) δ -143.9, -158.6, -163.2; **HRMS**: a stable ion was not found in ESI and CI. **Chiral SFC:** DAICEL CHIRALCEL SC column (25 cm), CO_2:*i*-PrOH 95:5, 2.0 mL/min, 254 nm, 170 bar. Retention times: 6.5 mins (minor), 7.0 mins (major), e.r. = 97:3.

SFC analysis of the racemate, prepared using rac-BINAP:

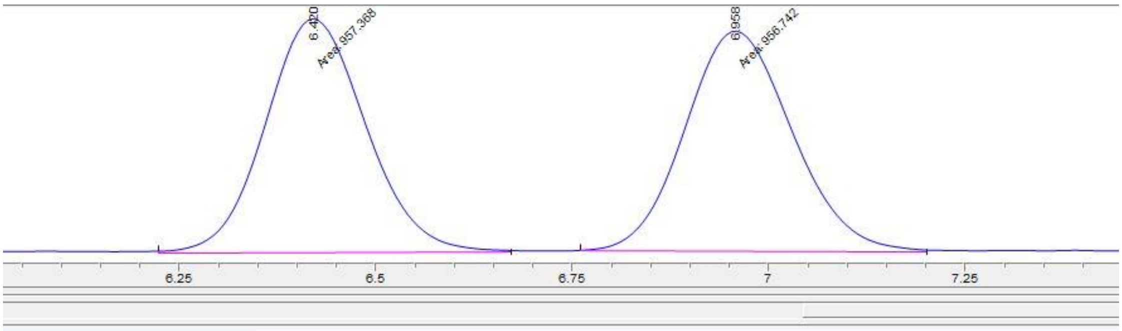

#	Time	Area	Height	Width	Area%	Symmetry
1	6.42	957.4	106.8	0.1494	50.016	0.892
2	6.958	956.7	100.5	0.1587	49.984	0.869

SFC analysis of the enantioenriched material, prepared using (R)-OMe-BIPHEP:

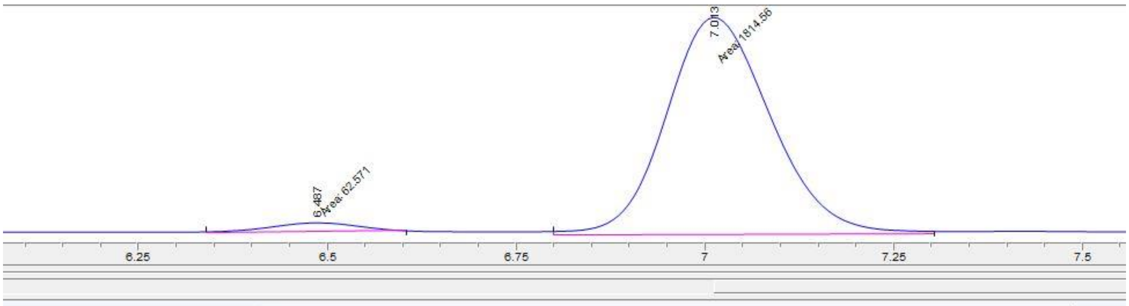

#	Time	Area	Height	Width	Area%	Symmetry
1	6.487	62.6	7.6	0.1369	3.333	1.294
2	7.013	1814.6	186.3	0.1623	96.667	0.832

The structure of this compound was determined by single crystal X-ray diffraction of crystals grown from EtOAc/hexane. See CCDC **2357629**.

Benzyl (*R*,*Z*)-3-methyl-2-((phenylamino)methylene)heptanoate (3as):

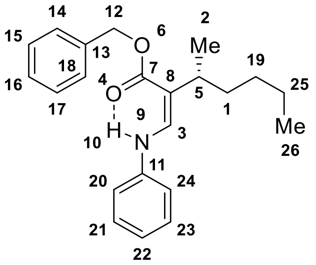

General procedure B: The reaction was carried out with substrate **1a** (25.3 mg, 0.10 mmol, 100 mol%), 1-hexene **2s** (38.0 µL, 0.30 mmol, 300 mol%) and (*R*)-SEGPHOS (3.05 mg, 5.00 µmol, 5.00 mol%). Purification of the residue by FCC (hexane/EtOAc 93:7) afforded the title compound (27.3 mg, 81%, >30:1 B:L, *Z/E* > 20:1, e.r. = 92:8) as a pale-yellow oil. ^{1}H NMR analysis of the crude material gave >30:1 B:L and *Z/E* > 20:1. $[\alpha]_D^{25}$ = -11.1 (c = 1.0, $CHCl_3$); **IR (thin film)** ν_{max}/cm^{-1}: 3323 (br), 2956 (s), 1665 (s), 1599 (s), 1153 (s), 693 (s); **^{1}H NMR** (500 MHz, $CDCl_3$) δ 9.99 (d, *J* = 12.5 Hz, 1H, H^{10}), 7.45 – 7.30 (m, 7H, H$^{14-18}$ + H^{21} + H^{23}),

7.21 (d, J = 12.5 Hz, 1H, H^3), 7.01 – 6.96 (m, 3H, H^{20} + H^{22} + H^{24}), 5.27 (s, 2H, H^{12}), 2.71 – 2.64 (m, 1H, H^5), 1.63 – 1.56 (m, 1H, H^1), 1.46 – 1.39 (m, 1H, H^1), 1.35 – 1.26 (m, 4H, H^{19} + H^{25}), 1.18 (d, J = 7.0 Hz, 3H, H^2), 0.90 (t, J = 7.0 Hz, 3H, H^{26}); **^{13}C NMR** (126 MHz, $CDCl_3$) δ 170.0 (C^7), 141.4 (C^{11}), 140.5 (C^3), 136.9 (C^{13}), 129.6 (C^{21} + C^{23}), 128.5 (C^{15} + C^{17}), 127.9 (C^{16}), 127.7 (C^{14} + C^{18}), 121.9 (C^{22}), 115.2 (C^{20} + C^{24}), 104.7 (C^8), 65.3 (C^{12}), 37.0 (C^1), 33.5 (C^5), 30.0 (C^{19}), 22.9 (C^{25}), 21.4 (C^2), 14.2 (C^{26}); **HRMS** (ESI): calculated for $C_{22}H_{28}NO_2$ $[M+H]^+$ requires *m/z* 338.2115, found *m/z* 338.2120; **Chiral SFC:** DAICEL CHIRALCEL SC column (25 cm), CO_2:*i*-PrOH 95:5, 1.0 mL/min, 254 nm, 111 bar. Retention times: 10.9 mins (minor), 11.3 mins (major), e.r. = 92:8.

SFC analysis of the racemates, prepared using rac-BINAP:

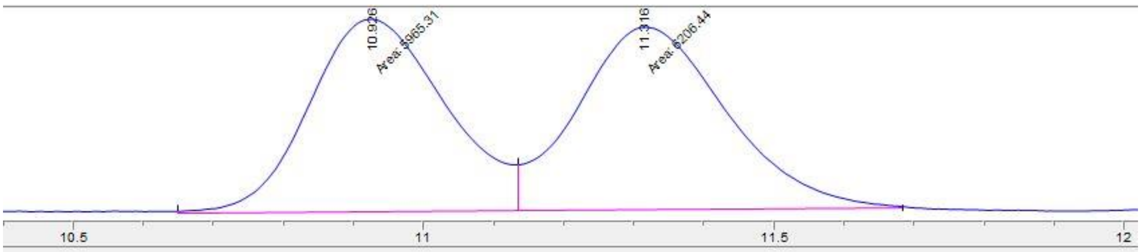

#	Time	Area	Height	Width	Area%	Symmetry
1	10.926	5965.3	439	0.2265	49.009	0.827
2	11.316	6206.4	417.5	0.2478	50.991	0.798

SFC analysis of the enantioenriched material, prepared using (R)-SEGPHOS:

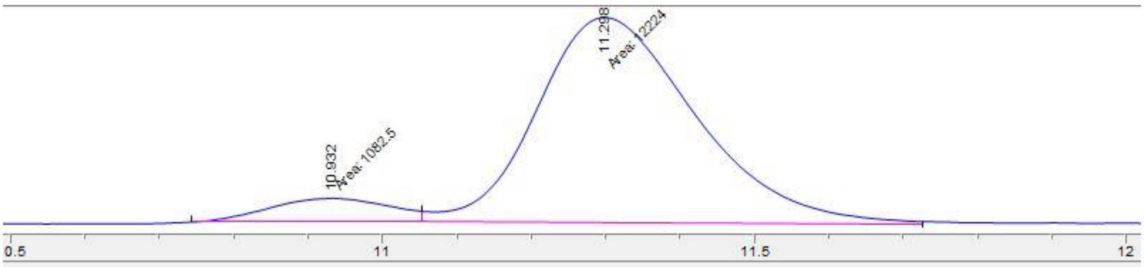

#	Time	Area	Height	Width	Area%	Symmetry
1	10.932	1082.5	95.3	0.1894	8.135	1.013
2	11.298	12224	819.3	0.2487	91.865	0.728

Benzyl (*R*,*Z*)-3-methyl-5-phenyl-2-((phenylamino)methylene)pentanoate (3at):

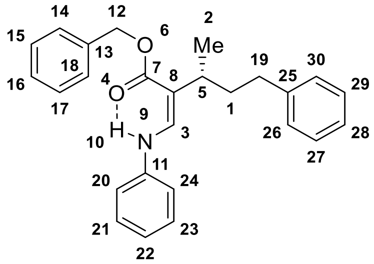

General procedure B: The reaction was carried out with substrate **1a** (25.3 mg, 0.10 mmol, 100 mol%), but-3-en-1-ylbenzene **2t** (45.0 µL, 0.30 mmol, 300 mol%) and (*R*)-SEGPHOS (3.05 mg, 5.00 µmol, 5.00 mol%). Purification of the residue by FCC (hexane/EtOAc 93:7) afforded the title compound (19.3 mg, 50%, >30:1 B:L, *Z/E* > 20:1, e.r. = 90:10) as a pale-yellow oil. ^{1}H NMR analysis of the crude material gave >30:1 B:L and *Z/E* = 15:1. $[\boldsymbol{\alpha}]_D^{25}$ = -9.4 (c = 1.0, $CHCl_3$); **IR (thin film)** ν_{max}/cm^{-1}: 3325 (br), 2930 (s), 1664 (s), 1599 (s), 1173 (s), 699 (s); **^{1}H NMR** (500 MHz, $CDCl_3$) δ 10.03 (d, *J* = 12.5 Hz, 1H, H^{10}), 7.43 – 7.27 (m, 9H, ArH), 7.23 – 7.15 (m, 4H, H^3 + ArH), 7.02 – 6.99 (m, 1H, H^{22}), 6.96 (d, *J* = 7.5 Hz, 2H, H^{20} + H^{24}), 5.27 (s, 2H, H^{12}), 2.73 – 2.67 (m, 1H, H^5), 2.64 (t, *J* = 8.1 Hz, 2H, H^{19}), 2.01 – 1.94 (m, 1H, H^1), 1.81 – 1.73 (m, 1H, H^1), 1.24 (d, *J* = 7.0 Hz, 3H, H^2); **^{13}C NMR** (126 MHz, $CDCl_3$) δ 169.9 (C^7), 142.7 (C^{25}), 141.2 (C^{11}), 140.9 (C^3), 136.8 (C^{13}), 129.7 (C^{21} + C^{23}), 128.6 (C^{15} + C^{17}), 128.4 (C^{27} + C^{29}), 128.3 (C^{26} + C^{30}), 128.0 (C^{16}), 127.9 (C^{14} + C^{18}), 125.6 (C^{28}), 122.0 (C^{22}), 115.3 (C^{20} + C^{24}), 103.9 (C^8), 65.4 (C^{12}), 38.7 (C^1), 34.1 (C^{19}), 33.7 (C^5), 21.3 (C^2); **HRMS** (ESI): calculated for $C_{26}H_{28}NO_2$ $[M+H]^+$ requires *m/z* 386.2115, found *m/z* 386.2118; **Chiral SFC:** DAICEL CHIRALCEL SB column (25 cm), CO_2:*i*-PrOH 95:5, 2.0 mL/min, 254 nm, 171 bar. Retention times: 16.4 mins (minor), 17.0 mins (major), e.r. = 90:10.

SFC analysis of the racemates, prepared using rac-BINAP:

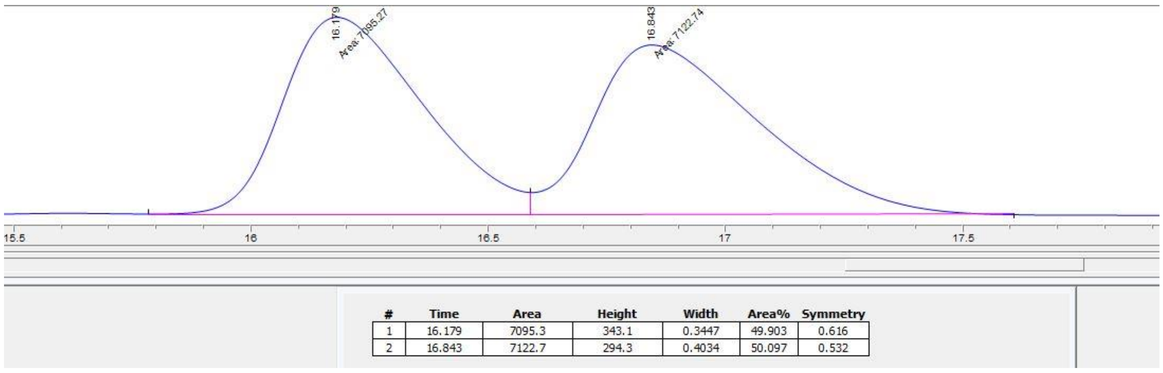

#	Time	Area	Height	Width	Area%	Symmetry
1	16.179	7095.3	343.1	0.3447	49.903	0.616
2	16.843	7122.7	294.3	0.4034	50.097	0.532

SFC analysis of the enantioenriched material, prepared using (R)-SEGPHOS:

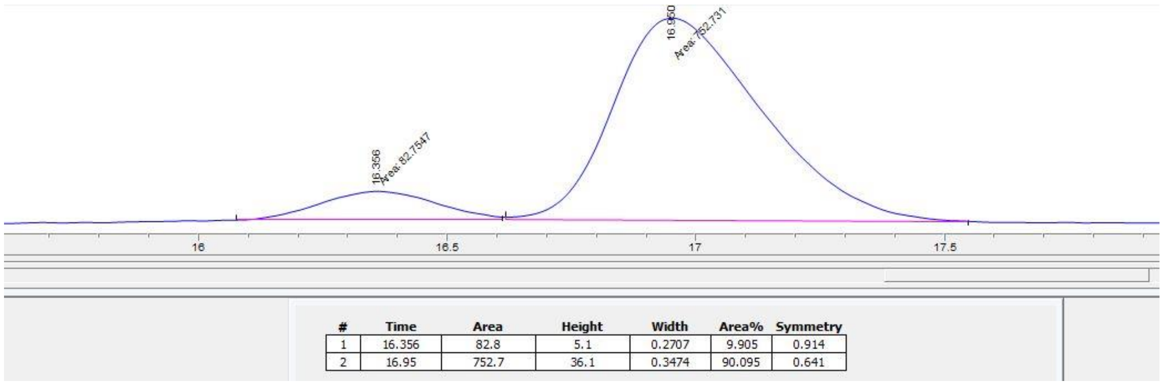

#	Time	Area	Height	Width	Area%	Symmetry
1	16.356	82.8	5.1	0.2707	9.905	0.914
2	16.95	752.7	36.1	0.3474	90.095	0.641

1-Benzyl 7-methyl (*R*,*Z*)-2-(((4-methoxyphenyl)amino)methylene)-3-methylheptanedioate (3hu):

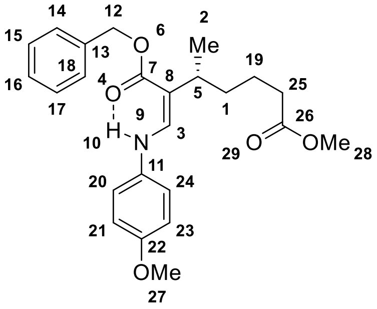

General procedure B: The reaction was carried out with substrate **1h** (28.3 mg, 0.10 mmol, 100 mol%), methyl hex-5-enoate **2u** (42.0 µL, 0.30 mmol, 300 mol%) and (*R*)-SEGPHOS (3.05 mg, 5.00 µmol, 5.00 mol%). Purification of the residue by FCC (hexane/EtOAc 93:7) afforded the title compound (25.5 mg, 62%, >30:1 B:L, *Z/E* > 20:1, e.r. = 91:9) as a pale-yellow oil. ^{1}H NMR analysis of the crude material gave >30:1 B:L and *Z/E* > 20:1. $[\alpha]_D^{25}$ = -12.9 (c = 1.0, $CHCl_3$); **IR (thin film)** ν_{max}/cm^{-1}: 3325 (br), 2926 (s), 1173 (s), 1663 (s), 1515 (s), 1167 (s), 698 (s); **^{1}H NMR** (500 MHz, $CDCl_3$) δ 9.90 (d, *J* = 12.5 Hz, 1H, H^{10}), 7.42 – 7.32 (m, 5H, ArH), 7.11 (d, *J* = 12.5 Hz, 1H, H^3), 6.91 (d, *J* = 9.0 Hz, 2H, H^{20} + H^{24}), 6.87 (d, *J* = 9.0 Hz, 2H, H^{21} + H^{23}), 5.24 (s, 2H, H^{12}), 3.80 (s, 3H, H^{27}), 3.67 (s, 3H, H^{28}), 2.69 – 2.62 (m, 1H, H^5), 2.30 (t, *J* = 7.0 Hz, 2H, H^{25}), 1.67 – 1.56 (m, 3H, H^1 + H^{19}), 1.48 – 1.41 (m, 1H, H^1), 1.17 (d, *J* = 7.0 Hz, 3H, H^2); **^{13}C NMR** (126 MHz, $CDCl_3$) δ 174.2 (C^{26}), 169.9 (C^7), 155.2 (C^{22}), 142.0 (C^3), 136.9 (C^{13}), 135.1 (C^{13}), 128.5 (C^{15} + C^{17}), 127.9 (C^{16}), 127.7 (C^{14} + C^{18}), 116.9 (C^{20} + C^{24}), 115.0 (C^{21} + C^{23}), 102.5 (C^8), 65.2 (C^{12}), 55.6 (C^{27}), 51.5 (C^{28}), 36.6 (C^1), 34.1 (C^{25}), 33.4 (C^5), 23.2 (C^{19}), 21.4 (C^2); **HRMS** (ESl): calculated for $C_{24}H_{29}NO_5Na$ $[M+Na]^+$ requires *m/z* 434.1938, found *m/z* 434.1950; **Chiral SFC:** DAICEL CHIRALCEL AD column (25 cm), CO_2:*i*-PrOH 85:15, 3.0 mL/min, 254 nm, 210 bar. Retention times: 10.8 mins (minor), 13.4 mins (major), e.r. = 91:9.

SFC analysis of the racemates, prepared using rac-BINAP:

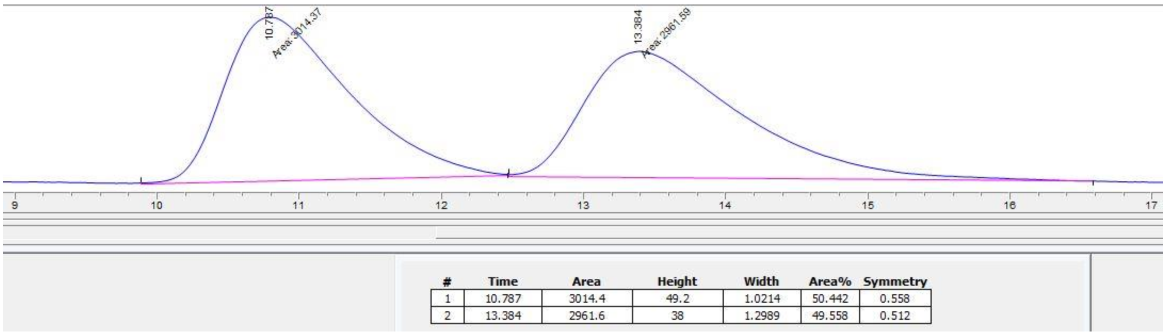

#	Time	Area	Height	Width	Area%	Symmetry
1	10.787	3014.4	49.2	1.0214	50.442	0.558
2	13.384	2961.6	38	1.2989	49.558	0.512

SFC analysis of the enantioenriched material, prepared using (R)-SEGPHOS:

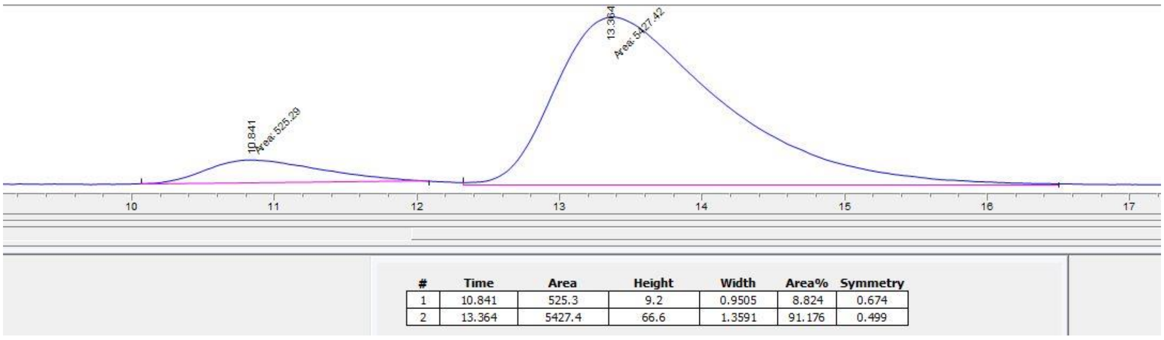

#	Time	Area	Height	Width	Area%	Symmetry
1	10.841	525.3	9.2	0.9505	8.824	0.674
2	13.364	5427.4	66.6	1.3591	91.176	0.499

Benzyl (*R*,*Z*)-5-acetoxy-3-methyl-2-((phenylamino)methylene)pentanoate (3av):

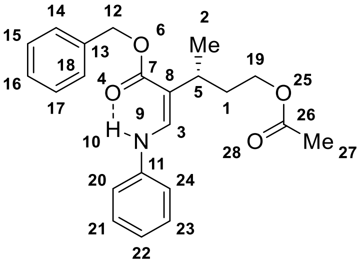

General procedure B: The reaction was carried out with substrate **1a** (25.3 mg, 0.10 mmol, 100 mol%), but-3-en-1-yl acetate **2v** (37.0 µL, 0.30 mmol, 300 mol%) and (*R*)-SEGPHOS (3.05 mg, 5.00 µmol, 5.00 mol%). Purification of the residue by FCC (hexane/EtOAc 93:7) afforded the title compound (20.6 mg, 56%, >30:1 B:L, *Z/E* > 20:1, e.r. = 89:11) as a pale-yellow oil. ^{1}H NMR analysis of the crude material gave >30:1 B:L and *Z/E* = 10:1. $[\boldsymbol{\alpha}]_{\boldsymbol{D}}^{\mathbf{25}}$ = -16.1 (c = 1.0, $CHCl_3$); **IR (thin film)** ν_{max}/cm^{-1}: 3325 (br), 2959 (s), 1734 (s), 1666 (s), 1599 (s), 1195 (s), 696 (s); **^{1}H NMR** (500 MHz, $CDCl_3$) δ 9.99 (d, *J* = 12.5 Hz, 1H, H^{10}), 7.44 – 7.30 (m, 7H, ArH), 7.24 (d, *J* = 12.5 Hz, 1H, H^3), 7.02 – 6.99 (m, 1H, H^{22}), 6.96 (d, *J* = 7.5 Hz, 2H, H^{20} + H^{24}), 5.25 (s, 2H, H^{12}), 4.14 – 4.07 (m, 2H, H^{19}), 2.79 – 2.72 (m, 1H, H^5), 2.01 (s, 3H, H^{27}), 1.99 – 1.93 (m, 3H, H^1 + H^{19}), 1.81 – 1.74 (m, 1H, H^1), 1.23 (d, *J* = 7.0 Hz, 3H, H^2); **^{13}C**

NMR (126 MHz, $CDCl_3$) δ 171.2 (C^{26}), 169.7 (C^7), 141.13 (C^3), 141.09 (C^{11}), 136.6 (C^{13}), 129.7 (C^{21} + C^{23}), 128.6 (C^{15} + C^{17}), 128.0 (C^{16}), 127.8 (C^{14} + C^{18}), 122.2 (C^{22}), 115.4 (C^{20} + C^{24}), 103.1 (C^8), 65.5 (C^{12}), 63.3 (C^{19}), 35.5 (C^1), 31.3 (C^5), 21.3 (C^2), 21.0 (C^{27}); **HRMS** (ESl): calculated for $C_{22}H_{25}NO_4Na$ $[M+Na]^+$ requires *m/z* 390.1676, found *m/z* 390.1689; **Chiral SFC:** DAICEL CHIRALCEL SC column (25 cm), CO_2:*i*-PrOH 95:5, 2.0 mL/min, 254 nm, 170 bar. Retention times: 8.4 mins (minor), 9.0 mins (major), e.r. = 89:11.

SFC analysis of the racemate, prepared using rac-BINAP:

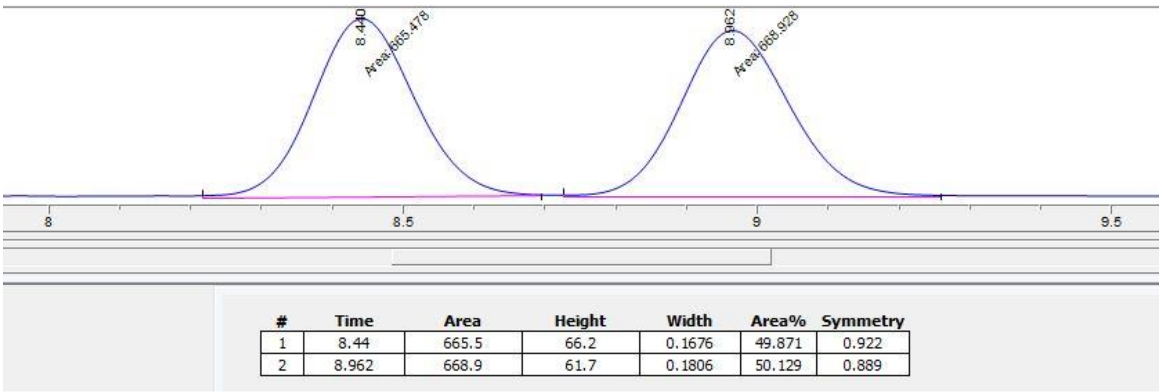

#	Time	Area	Height	Width	Area%	Symmetry
1	8.44	665.5	66.2	0.1676	49.871	0.922
2	8.962	668.9	61.7	0.1806	50.129	0.889

SFC analysis of the enantioenriched material, prepared using (R)-SEGPHOS:

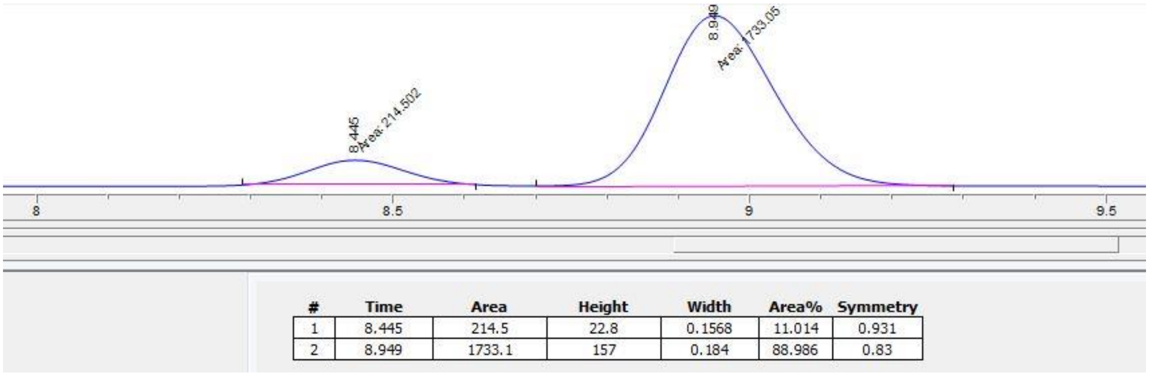

#	Time	Area	Height	Width	Area%	Symmetry
1	8.445	214.5	22.8	0.1568	11.014	0.931
2	8.949	1733.1	157	0.184	88.986	0.83

Benzyl (*R*,*Z*)-7-((*tert*-butyldimethylsilyl)oxy)-3-methyl-2-((phenylamino)methylene)heptanoate (3aw):

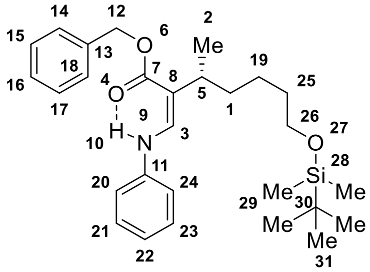

General procedure B: The reaction was carried out with substrate **1a** (25.3 mg, 0.10 mmol, 100 mol%), *tert*-butyl(hex-5-en-1-yloxy)dimethylsilane **2w** (64.3 mg, 0.30 mmol, 300 mol%)

and (*R*)-SEGPHOS (3.05 mg, 5.00 µmol, 5.00 mol). Purification of the residue by FCC (hexane/EtOAc 93:7) afforded the title compound (28.5 mg, 61%, >30:1 B:L, *Z/E* > 20:1, e.r. = 91:9) as a pale-yellow oil. ^{1}H NMR analysis of the crude material gave >30:1 B:L and *Z/E* = 17:1. $[\alpha]_D^{25}$ = -5.3 (c = 1.0, $CHCl_3$); **IR (thin film)** ν_{max}/cm^{-1}: 3365 (br), 2929 (s), 1667 (s), 1600 (s), 1177 (s), 694 (s); **^{1}H NMR** (500 MHz, $CDCl_3$) δ 9.97 (d, *J* = 12.5 Hz, 1H, H^{10}), 7.44 – 7.29 (m, 7H, ArH), 7.19 (d, *J* = 12.5 Hz, 1H, H^3), 7.00 – 6.94 (m, 3H, H^{20} + H^{22} + H^{24}), 5.25 (s, 2H, H^{12}), 3.60 (t, *J* = 6.5 Hz, 2H, H^{26}), 2.70 – 2.63 (m, 1H, H^5), 1.63 – 1.29 (m, 6H, H^1 + H^{19} + H^{25}), 1.17 (d, *J* = 7.0 Hz, 3H, H^2), 0.90 (s, 9H, H^{31}), 0.06 (s, 6H, H^{29}); **^{13}C NMR** (126 MHz, $CDCl_3$) δ 169.9 (C^7), 141.3 (C^{11}), 140.5 (C^3), 136.8 (C^{13}), 129.6 (C^{21} + C^{23}), 128.5 (C^{15} + C^{17}), 127.9 (C^{16}), 127.7 (C^{14} + C^{18}), 121.9 (C^{22}), 115.2 (C^{20} + C^{24}), 104.5 (C^8), 65.3 (C^{12}), 63.3 (C^{26}), 37.0 (C^1), 33.5 (C^5), 33.0 (C^{25}), 26.0 (C^{31}), 24.0 (C^{19}), 21.3 (C^2), 18.4 (C^{30}), -5.2 (C^{29}); **HRMS** (ESI): calculated for $C_{28}H_{42}NO_3Si$ $[M+H]^+$ requires *m/z* 468.2928, found *m/z* 468.2931; **Chiral SFC:** DAICEL CHIRALCEL SC column (25 cm), CO_2:*i*-PrOH 98:2, 1.0 mL/min, 254 nm, 170 bar. Retention times: 7.9 mins (minor), 8.3 mins (major), e.r. = 91:9.

SFC analysis of the racemate, prepared using rac-BINAP:

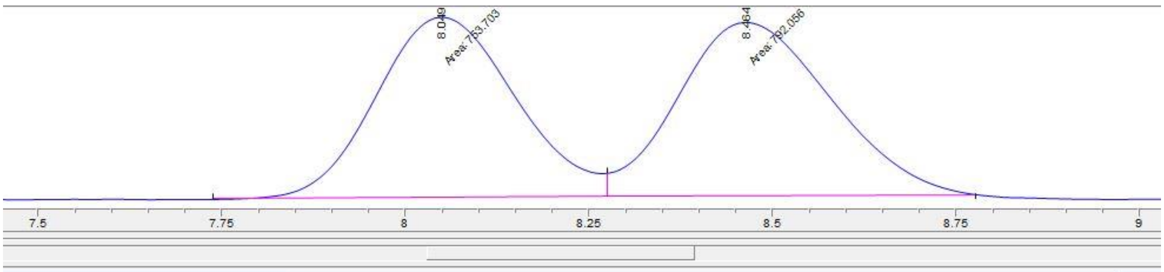

#	Time	Area	Height	Width	Area%	Symmetry
1	8.049	753.7	57	0.2204	48.759	0.871
2	8.464	792.1	54.8	0.2409	51.241	0.787

SFC analysis of the enantioenriched material, prepared using (R)-SEGPHOS:

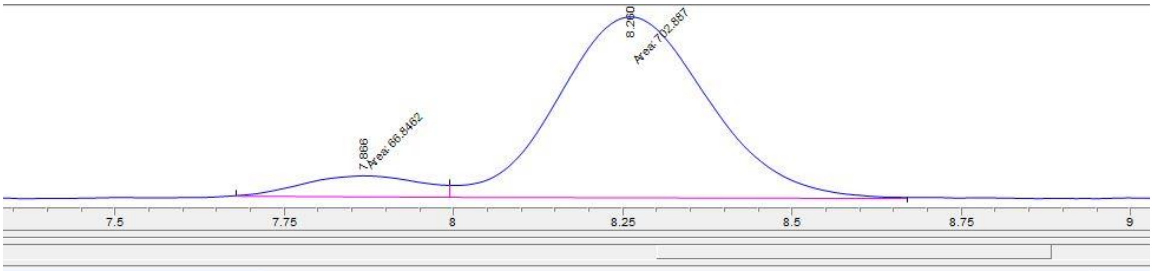

#	Time	Area	Height	Width	Area%	Symmetry
1	7.866	66.8	5.3	0.2085	8.684	1.007
2	8.26	702.9	45.3	0.2583	91.316	0.906

Benzyl (*R*,*Z*)-3-(4-((((*S*)-2-(6-methoxynaphthalen-2-yl)propanoyl)oxy)methyl)phenyl)-2-((phenylamino)methylene)butanoate (3ax):

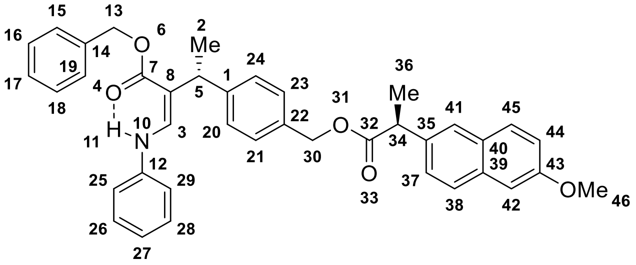

General procedure B: The reaction was carried out with substrate **1a** (25.3 mg, 0.10 mmol, 100 mol%) and 4-vinylbenzyl (*S*)-2-(6-methoxynaphthalen-2-yl)propanoate **2x** (104 mg, 0.30 mmol, 300 mol%). Purification of the residue by FCC (hexane/EtOAc 90:10) afforded the title compound (34.8 mg, 58%, >30:1 B:L, *Z/E* > 20:1, d.r. > 20:1) as a pale-yellow oil. ^{1}H NMR analysis of the crude material gave >30:1 B:L, *Z/E* > 20:1 and d.r. > 20:1. **IR (thin film)** ν_{max}/cm^{-1}: 3315 (br), 2933 (s), 1731 (s), 1668 (s), 1600 (s), 1176 (s), 696 (s); **^{1}H NMR** (500 MHz, $CDCl_3$) δ 10.08 (d, *J* = 12.5 Hz, 1H, H^{11}), 7.73 – 7.69 (m, 3H, ArH), 7.45 (dd, *J* = 8.5, 2.0 Hz, 1H, H^{41}), 7.34 – 7.27 (m, 5H, H^3 + ArH), 7.23 – 7.14 (m, 6H, H^{42} + ArH), 7.10 – 7.08 (m, 2H, ArH), 7.03 – 7.00 (m, 1H, H^{27}), 6.95 (d, *J* = 8.5 Hz, 2H, H^{25} + H^{29}), 5.20 – 5.09 (m, 4H, H^{13} + H^{30}), 4.07 (q, *J* = 7.0 Hz, 1H, H^5), 3.97 – 3.93 (m, 4H, H^{34} + H^{46}), 1.64 (d, *J* = 7.1 Hz, 3H, H^{36}), 1.49 (d, *J* = 7.0 Hz, 3H, H^2); **^{13}C NMR** (126 MHz, $CDCl_3$) δ 174.6 (C^{32}), 169.4 (C^7), 157.7 (C^{43}), 146.9 (C^1), 141.2 (C^{12}), 141.1 (C^3), 136.5 (C^{14}), 135.6 (ArC), 133.7 (ArC), 133.5 (ArC), 129.7 (C^{26} + C^{28}), 129.3 (ArC), 129.0 (ArC), 128.4 (C^{16} + C^{18}), 128.1 (C^{21} + C^{23}), 127.8 (C^{17}), 127.6 (C^{15} + C^{19}), 127.5 (C^{20} + C^{24}), 127.2 (ArC), 126.3 (ArC), 126.0 (ArC), 122.2 (C^{27}), 119.0 (ArC), 115.4 (C^{25} + C^{29}), 105.6 (C^{42}), 104.0 (C^8), 66.5 (C^{30}), 65.4 (C^{13}), 55.3 (C^{46}), 45.5 (C^{34}), 38.5 (C^5), 21.4 (C^2), 18.7 (C^{36}); **HRMS** (ESl): calculated for $C_{39}H_{37}NO_5Na$ $[M+Na]^+$ requires *m/z* 622.2564, found *m/z* 622.2574.

1-Benzyl 6-((3*S*,8*S*,9*S*,10*R*,13*R*,14*S*,17*R*)-10,13-dimethyl-17-((*R*)-6-methylheptan-2-yl)-2,3,4,7,8,9,10,11,12,13,14,15,16,17-tetradecahydro-1*H*-cyclopenta[*a*]phenanthren-3-yl) (*R*,*Z*)-3-methyl-2-((phenylamino)methylene)hexanedioate (3ay):

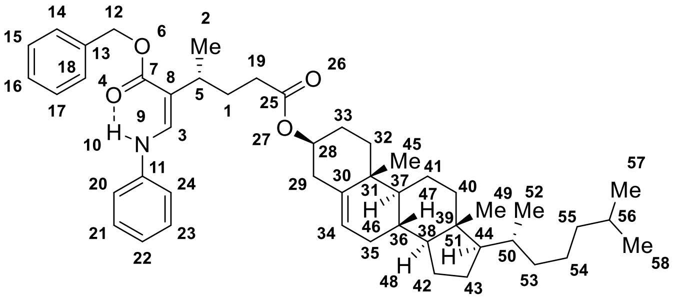

General procedure B: The reaction was carried out with substrate **1a** (25.3 mg, 0.10 mmol, 100 mol%), (3*S*,8*S*,9*S*,10*R*,13*R*,14*S*,17*R*)-10,13-dimethyl-17-((*R*)-6-methylheptan-2-yl)-2,3,4,7,8,9,10,11,12,13,14,15,16,17-tetradecahydro-1*H*-cyclopenta[*a*]phenanthren-3-yl pent-4-enoate **2y** (141 mg, 0.30 mmol, 300 mol%) and (*R*)-SEGPHOS (3.05 mg, 5.00 µmol, 5.00 mol). Purification of the residue by FCC (hexane/EtOAc 90:10) afforded the title compound (46.2 mg, 64%, >30:1 B:L, *Z/E* > 20:1, d.r. > 20:1) as a pale-yellow solid. ^{1}H NMR analysis of the crude material gave >30:1 B:L, *Z/E* > 20:1 and d.r. > 20:1. **m.p.** = 69 – 71 °C (hexane/EtOAc); **IR (thin film)** ν_{max}/cm^{-1}: 3305 (br), 2933 (s), 1728 (s), 1667 (s), 1600 (s), 1169 (s), 695 (s); **^{1}H NMR** (500 MHz, $CDCl_3$) δ 10.01 (d, *J* = 12.5 Hz, 1H, H^{10}), 7.44 – 7.28 (m, 7H, ArH), 7.21 (d, *J* = 12.5 Hz, 1H, H^3), 7.01 – 6.95 (m, 3H, H^{20} + H^{22} + H^{24}), 5.36 (d, *J* = 5.5 Hz, 1H, H^{34}), 5.26 (s, 2H, H^{12}), 4.64 – 4.58 (m, 1H, H^{28}), 2.71 – 2.64 (m, 1H, H^5), 2.31 – 2.28 (m, 4H), 2.05 – 1.80 (m, 7H), 1.64 – 1.32 (m, 11H), 1.22 – 0.99 (m, 15H), 0.95 (d, *J* = 6.5 Hz, 3H), 0.90 (dd, *J* = 6.5, 2.5 Hz, 6H, H^{57} + H^{58}), 0.70 (s, 3H); **^{13}C NMR** (126 MHz, $CDCl_3$) δ 173.3 (C^{25}), 169.8 (C^7), 141.3 (C^{11}), 141.1 (C^3), 139.7 (C^{30}), 136.7 (C^{13}), 129.6 (C^{21} + C^{23}), 128.6 (C^{15} + C^{17}), 128.0 (C^{16}), 127.8 (C^{14} + C^{18}), 122.6 (C^{34}), 122.1 (C^{22}), 115.4 (C^{20} + C^{24}), 102.9 (C^8), 73.8 (C^{28}), 65.5 (C^{12}), 56.7, 56.2, 50.0, 42.3, 39.8, 39.5, 38.2, 37.0, 36.6, 36.2, 35.8, 33.8, 33.0, 31.92, 31.87, 31.85, 28.3, 28.0, 27.9, 24.3, 23.9, 22.9, 22.6, 21.2, 21.0, 19.3, 18.7, 11.9; **HRMS** (ESI): calculated for $C_{48}H_{67}NO_4Na$ [M+Na]$^+$ requires *m/z* 744.4962, found *m/z* 744.4958.

Benzyl (*R*,*Z*)-3-((8*R*,9*S*,13*S*,14*S*)-13-methyl-17-*oxo*-7,8,9,11,12,13,14,15,16,17-decahydro-6*H*-cyclopenta[*a*]phenanthren-3-yl)-2-((phenylamino)methylene)butanoate (3az):

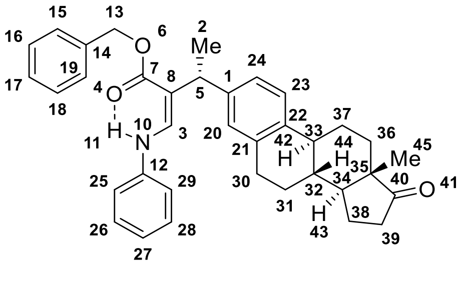

General procedure B: The reaction was carried out with substrate **1a** (25.3 mg, 0.10 mmol, 100 mol%) and (8*R*,9*S*,13*S*,14*S*)-13-methyl-3-vinyl-6,7,8,9,11,12,13,14,15,16-decahydro-17*H*-cyclopenta[*a*]phenanthren-17-one **2z** (84.1 mg, 0.30 mmol, 300 mol%). Purification of the residue by FCC (hexane/EtOAc 92:8) afforded the title compound (36.8 mg, 69%, >30:1 B:L, *Z/E* > 20:1, d.r. > 20:1) as a pale-yellow solid. ^{1}H NMR analysis of the crude material gave >30:1 B:L, *Z/E* > 20:1 and d.r. > 20:1. **m.p.** = 113 – 115 °C (hexane/EtOAc); **IR (thin film)** ν_{max}/cm^{-1}: 3298 (br), 2928 (s), 1733 (s), 1663 (s), 1598 (s), 1191 (s), 694 (s); **^{1}H NMR**

(500 MHz, $CDCl_3$) δ 10.07 (d, *J* = 12.5 Hz, 1H, H^{11}), 7.34 – 7.31 (m, 6H, H^3 + ArH), 7.24 (d, *J* = 8.0 Hz, 1H, H^{23}), 7.16 – 7.15 (m, 2H, ArH), 7.07 (d, *J* = 8.0 Hz, 1H, H^{24}), 7.02 – 6.95 (m, 4H, H^{27} + H^{25} + H^{29} + H^{20}), 5.22 – 5.12 (m, 2H, H^{13}), 4.04 (q, *J* = 7.0 Hz, 1H, H^5), 2.93 – 2.84 (m, 2H, H^{39}), 2.57 – 2.45 (m, 2H), 2.36 – 2.31 (m, 1H), 2.22 – 1.99 (m, 4H), 1.71 – 1.43 (m, 9H, H^2 + H^{30-39} + H^{42-44}), 0.87 (s, 3H, H^{45}); **^{13}C NMR** (126 MHz, $CDCl_3$) δ 169.5 (C^7), 144.2 (C^1), 141.3 (C^{12}), 141.0 (C^3), 137.2 (C^{21}), 136.7 (C^{14}), 136.2 (C^{22}), 129.7 (C^{26} + C^{28}), 128.4 (C^{16} + C^{18}), 128.2 (C^{20}), 127.7 (C^{17}), 127.6 (C^{15} + C^{19}), 125.2 (C^{23}), 124.7 (C^{24}), 122.1 (C^{27}), 115.3 (C^{25} + C^{29}), 104.3 (C^8), 65.3 (C^{13}), 50.6 , 48.1, 44.4 (C^{33}), 38.3, 38.1, 35.9, 31.7, 29.5, 26.7, 25.8, 21.6, 21.5, 13.9 (C^{45}); **HRMS** (ESI): calculated for $C_{36}H_{39}NO_3Na$ $[M+Na]^+$ requires *m/z* 556.2822, found *m/z* 556.2825.

Benzyl (*R,Z*)-3-(4-((2-(1-(4-chlorobenzoyl)-5-methoxy-2-methyl-1*H*-indol-3-yl)acetoxy)methyl)phenyl)-2-((phenylamino)methylene)butanoate (3aaa):

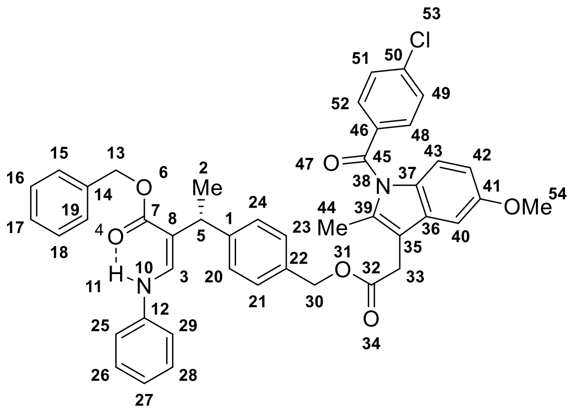

General procedure B: The reaction was carried out with substrate **1a** (25.3 mg, 0.10 mmol, 100 mol%) and 4-vinylbenzyl 2-(1-(4-chlorobenzoyl)-5-methoxy-2-methyl-1*H*-indol-3-yl)acetate **2aa** (142 mg, 0.30 mmol, 300 mol%). Purification of the residue by FCC (hexane/EtOAc 85:15) afforded the title compound (41.4 mg, 57%, >30:1 B:L, *Z/E* > 20:1, e.r. = 97:3) as a pale-yellow oil. ^{1}H NMR analysis of the crude material gave >30:1 B:L and *Z/E* = 10:1. $[\alpha]_D^{25}$ = -37.4 (c = 1.0, $CHCl_3$); **IR (thin film)** ν_{max}/cm^{-1}: 3335 (br), 2930 (s), 1736 (s), 1671 (s), 1600 (s), 1223 (s), 753 (s); **^{1}H NMR** (500 MHz, $CDCl_3$) δ 10.08 (d, *J* = 12.5 Hz, 1H, H^{11}), 7.66 (d, *J* = 8.0 Hz, 2H, H^{48} + H^{52}), 7.47 (d, *J* = 8.0 Hz, 2H, H^{49} + H^{51}), 7.33 – 7.23 (m, 10H, H^3 + ArH), 7.10 – 7.08 (m, 2H, ArH), 7.02 – 6.89 (m, 5H, H^{25} + H^{27} + H^{29} + H^{40} + H^{43}), 6.70 – 6.68 (m, 1H, H^{42}), 5.16 – 5.09 (m, 4H, H^{13} + H^{30}), 4.08 (q, *J* = 7.0 Hz, 1H, H^5), 3.78 (s, 3H, H^{54}), 3.75 (s, 2H, H^{33}), 2.40 (s, 3H, H^{44}), 1.50 (d, *J* = 7.0 Hz, 3H, H^2); **^{13}C NMR** (126 MHz, $CDCl_3$) δ 170.8 (C^{32}), 169.4 (C^7), 168.3 (C^{45}), 156.1 (C^{41}), 147.2 (C^1), 141.14 (C^{12}), 141.10 (C^3), 139.3 (C^{50}), 136.5 (C^{14}), 136.0 (ArC), 133.9 (ArC), 133.2 (ArC), 131.2 (C^{48} +

C[52]), 130.8 (ArC), 130.6 (ArC), 129.7 (C[26] + C[28]), 129.1 (C[49] + C[51]), 128.4 (C[16] + C[18]), 128.2 (ArC), 127.8 (C[17]), 127.6 (C[15] + C[19]), 122.2 (C[27]), 115.4 (C[25] + C[29]), 115.0 (C[43]), 112.6 (C[42]), 111.8 (C[42]), 103.9 (C[8]), 101.3 (C[40]), 66.8 (C[30]), 65.4 (C[13]), 55.6 (C[54]), 38.5 (C[5]), 30.5 (C[33]), 21.4 (C[2]), 13.9 (C[44]); **HRMS** (ESI): calculated for $C_{44}H_{39}{}^{35}ClN_2O_6Na$ $[M+Na]^+$ requires *m/z* 749.2389, found *m/z* 749.2383; **Chiral SFC:** DAICEL CHIRALCEL OD-H column (25 cm), CO_2:*i*-PrOH 70:30, 2.0 mL/min, 254 nm, 195 bar. Retention times: 14.5 mins (major), 16.5 mins (minor), e.r. = 97:3.

SFC analysis of the racemate, prepared using rac-BINAP:

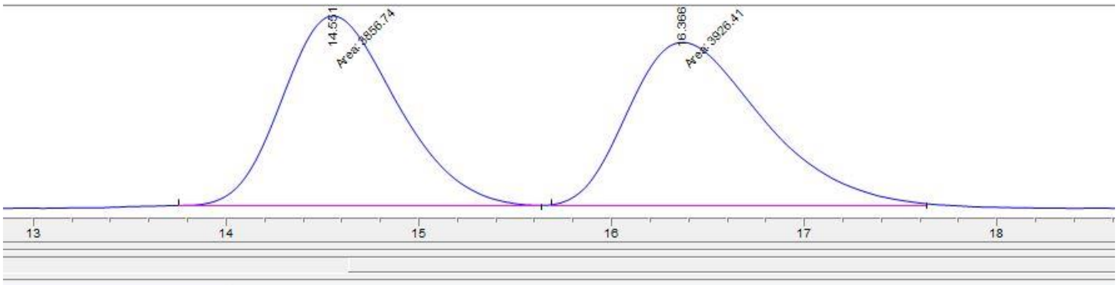

#	Time	Area	Height	Width	Area%	Symmetry
1	14.551	3856.7	93.2	0.6899	49.552	0.771
2	16.366	3926.4	80.2	0.8155	50.448	0.655

SFC analysis of the enantioenriched material, prepared using (R)-OMe-BIPHEP:

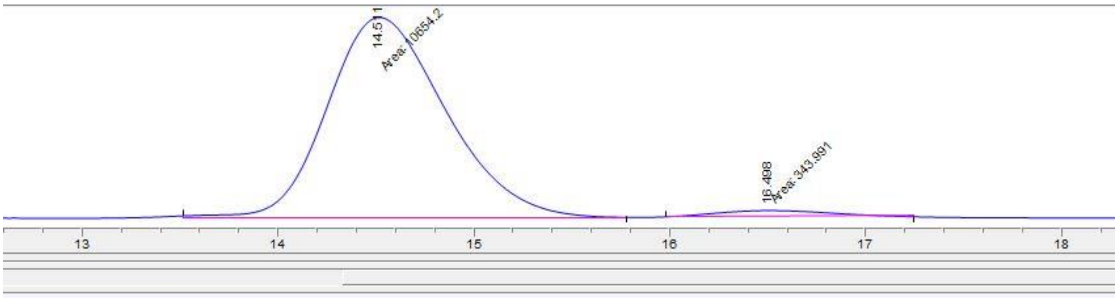

#	Time	Area	Height	Width	Area%	Symmetry
1	14.511	10654.2	249.6	0.7115	96.872	0.772
2	16.498	344	8.3	0.4911	3.128	0.921

Benzyl (*S*,*Z*)-2-(((4-methoxyphenyl)amino)methylene)-3-phenylbutanoate (*ent*-3ha):

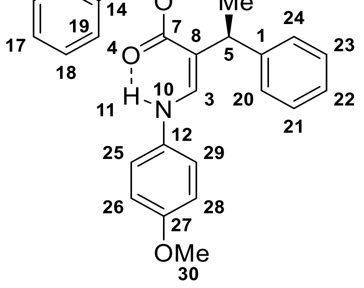

General procedure B: The reaction was carried out with substrate **1h** (28.3 mg, 0.10 mmol, 100 mol%), styrene **2a** (34.0 µL, 0.30 mmol, 300 mol%) and (*S*)-OMe-BIPHEP (2.91 mg, 5.00 µmol, 5 mol%). Purification of the residue by FCC (hexane/EtOAc 90:10) afforded the title compound (30.2 mg, 78%, >30:1 B:L, *Z/E* > 20:1, e.r. = 5.5:95.5) as a pale-yellow oil. ^{1}H NMR analysis of the crude material gave >30:1 B:L and *Z/E* > 20:1. $[\boldsymbol{\alpha}]_D^{25}$ = 40.4 (c = 1.0, $CHCl_3$); **Chiral SFC:** DAICEL CHIRALCEL SC column (25 cm), CO_2:*i*-PrOH 95:5, 2.0 mL/min, 254 nm, 170 bar. Retention times: 11.8 mins (major), 12.9 mins (minor), e.r. = 4.5:95.5. All other analytical data was identical to that listed earlier for **3ha**.

SFC analysis of the racemate, prepared using rac-BINAP:

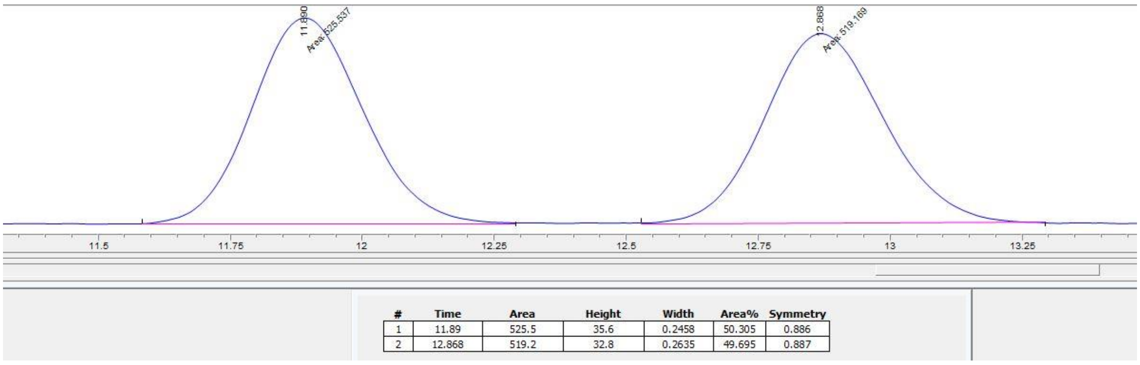

SFC analysis of the enantioenriched material, prepared using (S)-OMe-BIPHEP:

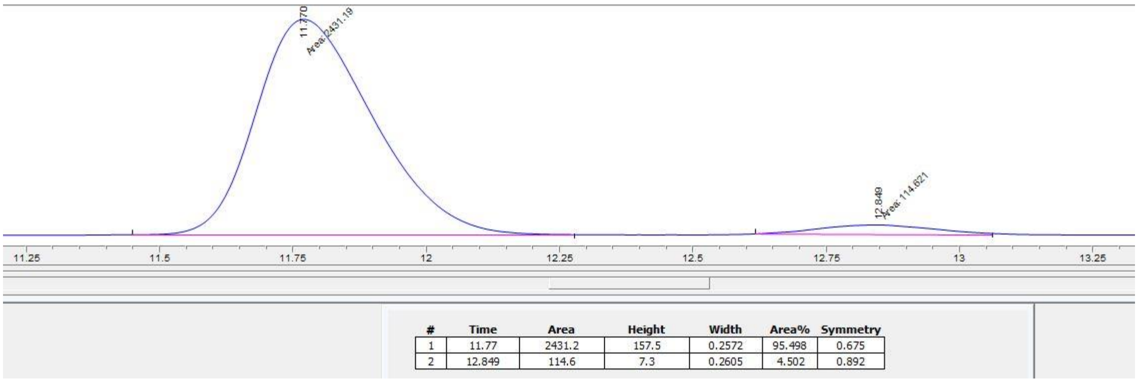

Figure S1

Other alkenes, including (1-cyclopropylvinyl)benzene, 1*H*-indene, (*E*)-prop-1-en-1-ylbenzene, but-3-en-1-yn-1-ylbenzene, 4-vinylpyridine, 6-vinylquinoline, methylenecyclohexane, 4,4,5,5-tetramethyl-2-vinyl-1,3,2-dioxaborolane, 2-ethylbut-3-enenitrile, 3-bromobut-1-ene and cyclohexene, have been evaluated and only led to the formation of the corresponding products in very low yields as yet.

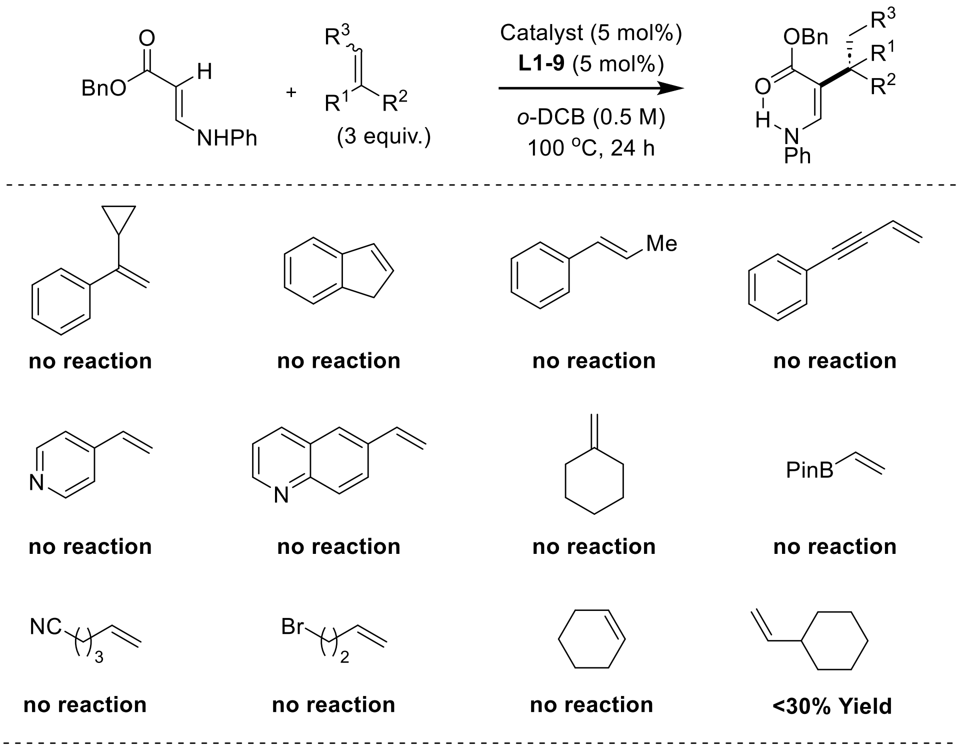

Applications and derivatizations

After extensive investigations, we found that the combination of **Cat. A**[14] and Cl_3SiH provides **4** in 5:1 d.r. and 75% yield.

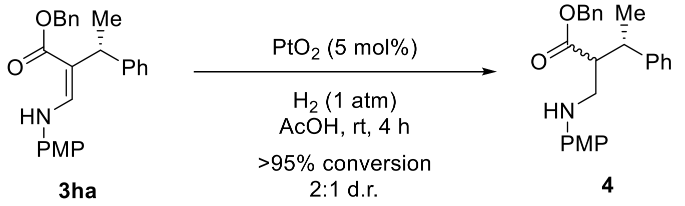

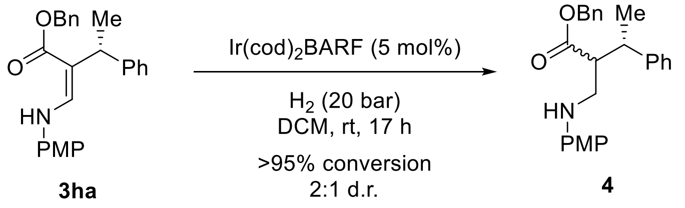

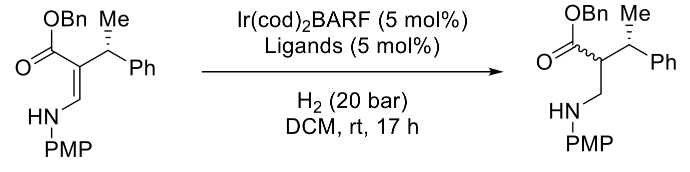

3ha **4**

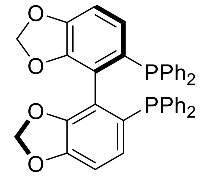

(R)-SEGPHOS
>95% conversion
1:1 d.r.

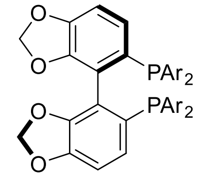

(R)-DTBM-SEGPHOS
~10% conversion
2:1 d.r.

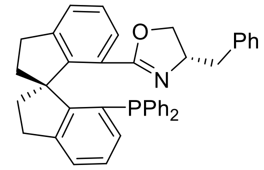

~10% conversion
2:1 d.r.

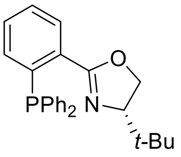

<5% conversion

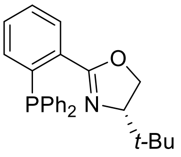

40% conversion
1:1 d.r.

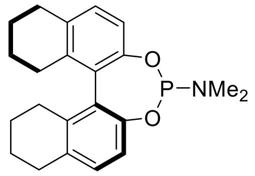

<5% conversion

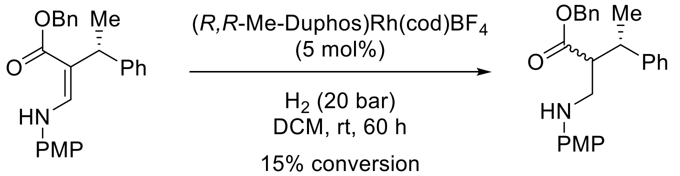

15% conversion
2:1 d.r.

3ha **4**

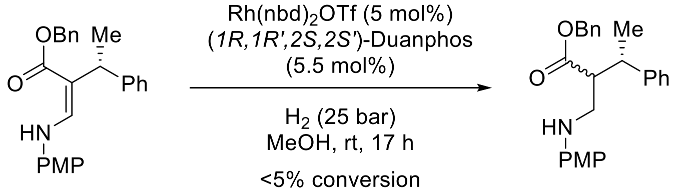

<5% conversion
2:1 d.r.

3ha **4**

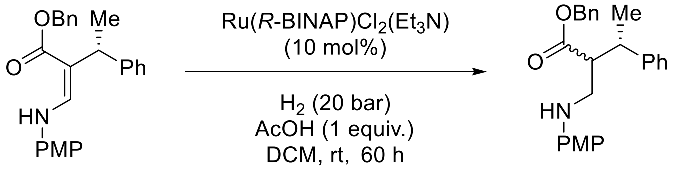

45% conversion
2:1 d.r.

3ha **4**

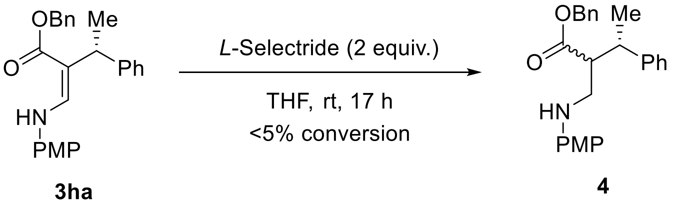

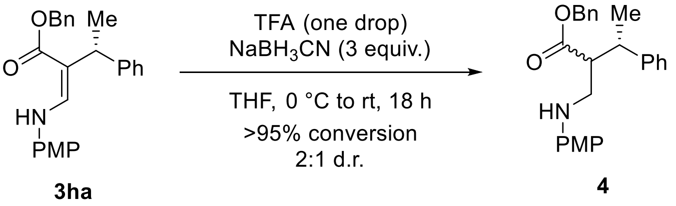

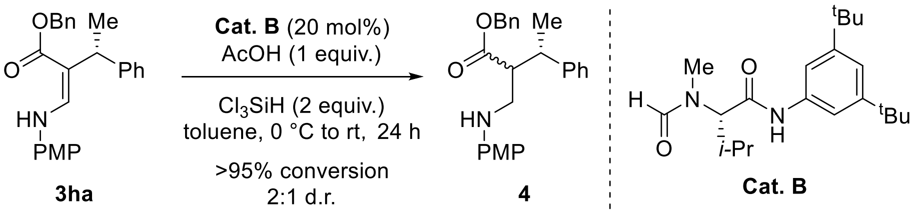

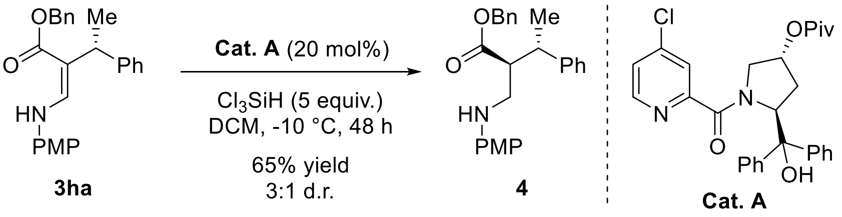

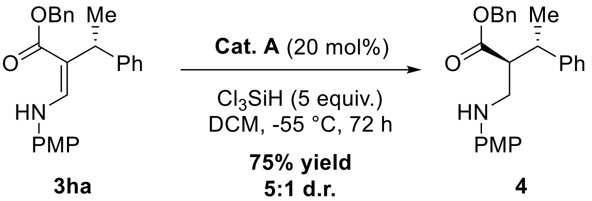

Benzyl (2*S*,3*S*)-2-(((4-methoxyphenyl)amino)methyl)-3-phenylbutanoate (4):

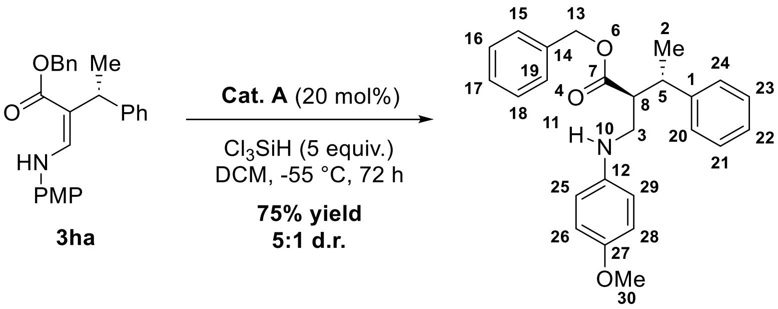

Cl_3SiH (51.0 µL, 0.50 mmol, 500 mol%) in anhydrous DCM (0.2 mL) was slowly added, portion-wise, to a solution of **3ha** (38.7 mg, 0.10 mmol, 100 mol%) and **Cat. A** (9.84 mg, 0.02 mmol, 20 mol%) in anhydrous DCM (0.8 mL) under N_2 at -55 °C in a sealed tube. After the addition was complete, the reaction mixture was stirred for 72 hours at -55 °C and the progress of the reaction was monitored by TLC. Upon completion, a saturated aq. $NaHCO_3$ solution (approx. 0.3 mL) was slowly added to quench excess Cl_3SiH. The resulting suspension was then warmed to r.t. and the reaction mixture was transferred to a separatory funnel and water (approx. 5.0 mL) was added. The aqueous phase was extracted with EtOAc (approx. 3 × 10.0 mL). The combined organic phases were dried over anhydrous $MgSO_4$. The concentration of the filtrate *in vacuo* was followed by FCC (hexane/EtOAc 10:90) to afford the desired product **4** (29.2 mg, 75%, d.r. >20:1) as a colorless oil. ^{1}H NMR analysis of the crude material gave d.r. = 5:1. **IR (thin film)** ν_{max}/cm^{-1}: 3315 (br), 2963 (s), 1726 (s), 1512 (s), 1236 (s), 1161 (s), 700 (s); **^{1}H NMR** (500 MHz, $CDCl_3$) δ 7.41 – 7.33 (m, 7H, H^{15-19} + H^{21} + H^{23}), 7.28 – 7.25 (m, 1H, H^{22}), 7.22 (d, J = 7.0 Hz, 2H, H^{20} + H^{24}), 6.71 (d, J = 9.0 Hz, 2H, H^{26} + H^{28}), 6.31 (d, J = 9.0 Hz, 2H, H^{25} + H^{29}), 5.25 – 5.18 (m, 2H, H^{13}), 3.74 (s, 3H, H^{30}), 3.48 (br. s, 1H, H^{11}), 3.25 – 3.20 (m, 1H, H^3), 3.11 – 3.05 (m, 1H, H^5), 2.99 – 2.92 (m, 2H, H^3 + H^8), 1.28 (d, J = 7.0 Hz, 3H, H^2); **^{13}C NMR** (126 MHz, $CDCl_3$) δ 174.6 (C^7), 152.2 (C^{27}), 143.9 (C^1), 141.5 (C^{12}), 135.8 (C^{14}), 128.8 (ArC), 128.6 (ArC), 128.42 (ArC), 128.36 (C^{17}), 127.3 (C^{20} + C^{24}), 126.9 (C^{22}), 114.8 (C^{26} + C^{28}), 114.3 (C^{25} + C^{29}), 66.5 (C^{13}), 55.8 (C^{30}), 52.6 (C^3), 46.1 (C^5), 40.9 (C^8), 20.7 (C^2); **HRMS** (ESl): calculated for $C_{25}H_{28}NO_3$ $[M+H]^+$ requires m/z 390.2064, found m/z 390.2070.

Benzyl (2*S*,3*S*)-2-(((*N*-(4-methoxyphenyl)-4-methylphenyl)sulfonamido)methyl)-3-phenylbutanoate (5):

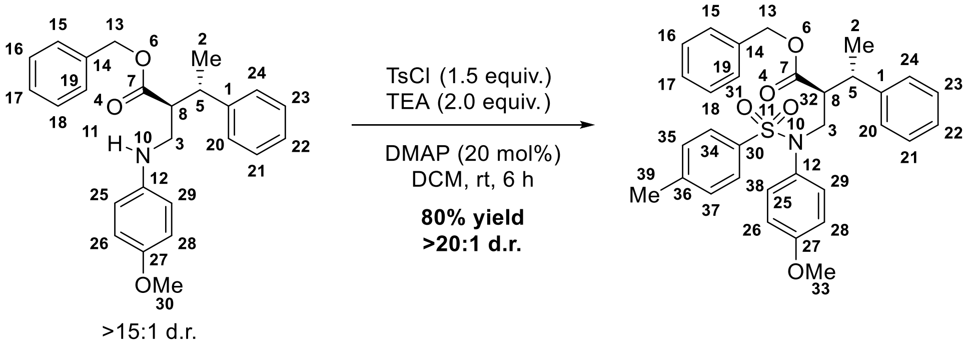

4-Methylbenzenesulfonyl chloride (28.5 mg, 0.15 mmol, 150 mol%) was slowly added to a solution of **4** (38.9 mg, 0.10 mmol, 100 mol%), Et_3N (28.0 µL, 0.20 mmol, 200 mol%), DMAP (2.44 mg, 0.02 mmol, 20 mol%) and CH_2Cl_2 (1.0 mL) at r.t. The reaction was stirred at ambient

temperature for 6 hours. Upon completion, the reaction mixture was diluted with CH_2Cl_2 (5.0 mL) and transferred to a separatory funnel. The mixture was washed with aq. 1N HCl (10.0 mL). The combined organic phases were dried over anhydrous $MgSO_4$. The concentration of the filtrate *in vacuo* was followed by FCC (EtOAc/hexane 10:90) to afford the desired product **5** (43.5 mg, 80%, d.r. >20:1) as a colorless solid. **m.p.** = 110 – 112 °C (hexane/EtOAc); **IR (thin film)** ν_{max}/cm^{-1}: 2931 (s), 1726 (s), 1599 (s), 1523 (s), 1266 (s), 748 (s); **^{1}H NMR** (500 MHz, $CDCl_3$) δ 7.44 – 7.36 (m, 5H, ArH), 7.30 – 7.22 (m, 5H, ArH), 7.15 (d, *J* = 8.5 Hz, 2H, H^{34} + H^{38}), 7.06 (d, *J* = 8.5 Hz, 2H, H^{35} + H^{37}), 6.76 – 6.71 (m, 4H, H^{25} + H^{26} + H^{28} + H^{29}), 5.16 – 5.05 (m, 2H, H^{13}), 3.81 (s, 3H, H^{33}), 3.78 – 3.74 (m, 1H, H^3), 3.22 – 3.19 (m, 1H, H^3), 2.98 – 2.88 (m, 2H, H^5 + H^8), 2.39 (s, 3H, H^{39}), 1.17 (d, *J* = 6.5 Hz, 3H, H^2); **^{13}C NMR** (126 MHz, $CDCl_3$) δ 173.4 (C^7), 159.0 (C^{27}), 143.6 (C^1), 143.3 (C^{36}), 135.7 (C^{14}), 134.7 (C^{30}), 131.9 (C^{12}), 129.9 (C^{35} + C^{37}), 129.3 (C^{25} + C^{29}), 128.7 (ArC), 128.6 (ArC), 128.5 (ArC), 128.3 (ArC), 127.8 (ArC), 127.2 (ArC), 126.8 (ArC), 114.0 (C^{26} + C^{28}), 66.7 (C^{13}), 55.4 (C^{33}), 52.3 (C^3), 52.2 (C^8), 40.7 (C^5), 21.5 (C^{39}), 20.6 (C^2); **HRMS** (ESl): calculated for $C_{32}H_{33}NO_5SNa$ $[M+Na]^+$ requires m/z 566.1972, found m/z 566.1964. The structure of this compound was determined by single crystal X-ray diffraction of crystals grown from EtOAc/hexane. See CCDC **2357628**.

Benzyl (2*R*,3*R*)-2-(((4-methoxyphenyl)amino)methyl)-3-phenylbutanoate (*ent*-4):

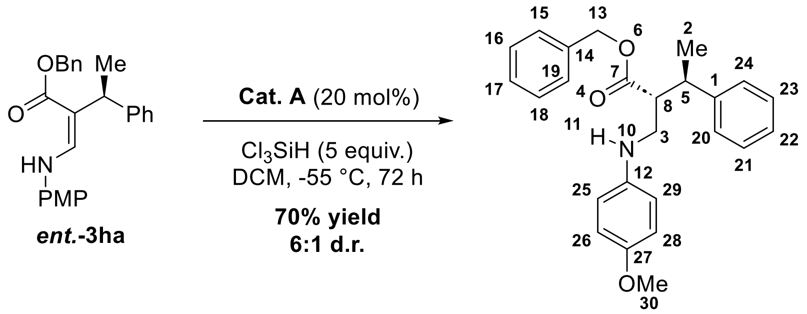

Cl_3SiH (51.0 µL, 0.50 mmol, 500 mol%) in anhydrous DCM (0.2 mL) was slowly added, portion-wise, to a solution of ***ent.*-3ha** (38.7 mg, 0.10 mmol, 100 mol%) and **Cat. A** (9.84 mg, 0.02 mmol, 20 mol%) in anhydrous DCM (0.8 mL) under N_2 at -55 °C in a sealed tube. After the addition was complete, the reaction mixture was stirred for 72 hours at -55 °C and the progress of the reaction was monitored by TLC. Upon completion, a saturated aq. $NaHCO_3$ solution (approx. 0.3 mL) was slowly added to quench excess Cl_3SiH. The resulting suspension was then warmed to r.t. and the reaction mixture was transferred to a separatory funnel and water (approx. 5.0 mL) was added. The aqueous phase was extracted with EtOAc (approx. 3 × 10.0 mL). The combined organic phases were dried over anhydrous $MgSO_4$. The concentration

of the filtrate *in vacuo* was followed by FCC (hexane/EtOAc 10:90) to afford the desired product ***ent*-4** (27.2 mg, 70%, d.r. >20:1) as a colorless oil. ^{1}H NMR analysis of the crude material gave d.r. = 6:1. The spectroscopic data for this compound were identical to those listed for **4**.

Benzyl (2*S*,3*S*)-3-(2-chlorophenyl)-2-((phenylamino)methyl)butanoate (6):

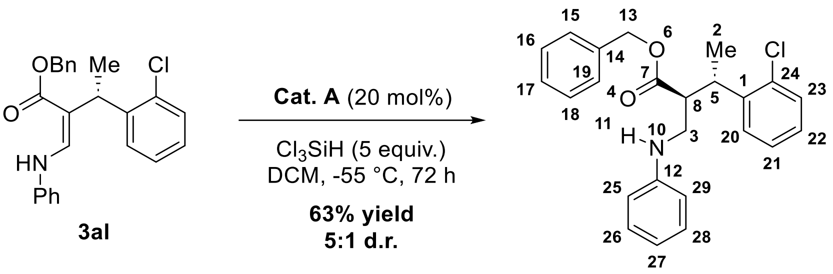

Cl_3SiH (51.0 µL, 0.50 mmol, 500 mol%) in anhydrous DCM (0.2 mL) was slowly added, portion-wise, to a solution of **3al** (39.1 mg, 0.10 mmol, 100 mol%) and **Cat. A** (9.84 mg, 0.02 mmol, 20 mol%) in anhydrous DCM (0.8 mL) under N_2 at -55 °C in a sealed tube. After the addition was complete, the reaction mixture was stirred for 72 hours at -55 °C and the progress of the reaction was monitored by TLC. Upon completion, a saturated aq. $NaHCO_3$ solution (approx. 0.3 mL) was slowly added to quench excess Cl_3SiH. The resulting suspension was then warmed to r.t. and the reaction mixture was transferred to a separatory funnel and water (approx. 5.0 mL) was added. The aqueous phase was extracted with EtOAc (approx. 3 × 10.0 mL). The combined organic phases were dried over anhydrous $MgSO_4$. The concentration of the filtrate *in vacuo* was followed by FCC (hexane/EtOAc 10:90) to afford the desired product **6** (27.2 mg, 63%, d.r. = 6:1) as a colorless oil. ^{1}H NMR analysis of the crude material gave d.r. = 6:1. **IR (thin film)** ν_{max}/cm^{-1}: 3311 (br), 2943 (s), 1725 (s), 1510 (s), 1235 (s), 1158 (s), 701 (s); **^{1}H NMR** (500 MHz, $CDCl_3$) Data for the major diastereomer *a*: δ 7.42 – 7.11 (m, 11H, H^{26} + H^{28} + ArH), 6.74 – 6.68 (m, 1H, H^{27}), 6.40 (d, J = 8.0 Hz, 2H, H^{25} + H^{29}), 5.22 – 5.16 (m, 2H, H^{13}), 3.90 – 3.67 (m, 2H, H^5 + H^{11}), 3.44 – 3.39 (m, 1H, H^3), 3.13 – 3.07 (m, 2H, H^3 + H^8), 1.28 (d, J = 7.0 Hz, 3H, H^2). Characteristic signals for the minor diastereomer *b*: 7.42 – 7.11 (m, 1.5H, H^{26} + H^{28} + ArH), 6.74 – 6.68 (m, 0.15H, H^{27}), 6.48 (d, J = 8.0 Hz, 0.3H, H^{25} + H^{29}), 5.06 – 4.99 (m, 0.33H, H^{13}), 3.57 – 3.52 (m, 0.21H, H^3), 3.23 – 3.19 (m, 0.18H, H^8), 1.34 (d, J = 7.0 Hz, 0.47H, H^2); **^{13}C NMR** (126 MHz, $CDCl_3$) Data for the major diastereomer *a*: δ 174.0 (C^7), 147.3 (C^{12}), 141.3 (C^1), 135.8 (C^{14}), 133.9 (C^{24}), 129.9 (C^{26} + C^{28}), 129.2 (C^{16} + C^{18}), 128.6 (ArC), 128.4 (ArC), 128.3 (ArC), 127.9 (ArC), 127.3 (ArC), 117.7 (C^{27}), 112.9 (C^{25} + C^{29}), 66.5 (C^{13}), 51.9 (C^8), 44.8 (C^3), 36.1 (C^1), 19.2 (C^2). Data for the major diastereomer *b*: δ

173.6 (C^7), 141.2 (C^1), 135.7 (C^{14}), 129.3 (ArC), 128.5 (ArC), 128.2 (ArC), 127.9 (ArC), 126.9 (ArC), 66.4 (C^{13}), 49.5 (C^{13}), 42.8 (C^3), 36.0 (C^1), 17.5 (C^2); **HRMS** (ESI): calculated for $C_{24}H_{25}{}^{35}ClNO_2$ $[M+H]^+$ requires m/z 394.1568, found m/z 394.1576.

Benzyl (3*S*,4*S*)-4-methyl-1-phenyl-1,2,3,4-tetrahydroquinoline-3-carboxylate (7):

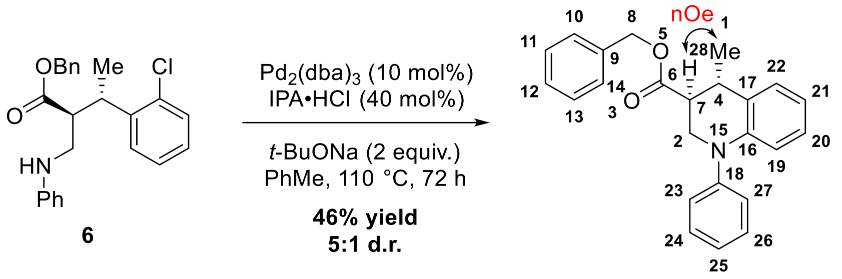

A Schlenk tube was charged with **6** (19.7 mg, 0.05 mmol, 100 mol%), $Pd_2(dba)_3$ (4.58 mg, 5.00 µmol, 10 mol%), IPr•HCl (8.54 mg, 0.02 mmol, 40 mol%) and *t*-BuONa (9.61 mg, 0.10 mmol, 200 mol%). The Schlenk tube was evacuated and refilled with N_2 (three cycles), then anhydrous PhMe (0.3 mL) was added at r.t. The tube was sealed and heated at 110 °C for 72 hours. After cooling to r.t., the concentration of the reaction mixture *in vacuo* was followed by FCC (hexane/EtOAc 10:90) to afford the desired product **7** (16.4 mg, 46%, d.r. = 5:1) as a colorless oil. ^{1}H NMR analysis of the crude material gave d.r. = 5:1. **IR (thin film)** ν_{max}/cm^{-1}: 2962 (s), 1730 (s), 1493 (s), 1161 (s), 746 (s), 695 (s); **^{1}H NMR** (500 MHz, $CDCl_3$) Data for the major diastereomer *a*: δ 7.41 – 7.31 (m, 5H, ArH), 7.26 – 7.09 (m, 6H, ArH), 6.99 – 6.96 (m, 1H, H^{27}), 6.82 – 6.80 (m, 1H, H^{21}), 6.76 (d, *J* = 8.0 Hz, 1H, H^{19}), 5.13 – 5.02 (m, 2H, H^{13}), 3.94 – 3.82 (m, 2H, H^2), 3.48 – 3.43 (m, 1H, H^4), 2.81 – 2.78 (m, 1H, H^7), 1.41 (d, *J* = 7.0 Hz, 3H, H^1). Characteristic signals for the minor diastereomer *b*: 7.41 – 7.31 (m, 1.34H, ArH), 7.26 – 7.09 (m, 0.68H, ArH), 6.99 – 6.96 (m, 0.2H, H^{27}), 6.76 (d, *J* = 8.0 Hz, 0.2H, H^{19}), 5.24 – 5.17 (m, 0.44H, H^{13}), 3.79 – 3.76 (m, 0.18H, H^2), 3.24 – 3.19 (m, 0.22H, H^2), 2.66 – 2.63 (m, 0.17H, H^7), 1.22 (d, *J* = 7.0 Hz, 0.58H, H^1); **^{13}C NMR** (126 MHz, $CDCl_3$) Data for the major diastereomer *a*: δ 173.0 (C^6), 147.7 (C^{18}), 143.1 (C^{16}), 135.7 (C^9), 129.4 (ArC), 128.7 (ArC), 128.5 (ArC), 128.19 (ArC), 128.16 (ArC), 126.5 (ArC), 124.6 (ArC), 123.9 (ArC), 119.1 (C^{21}), 116.1 (C^{19}), 66.6 (C^8), 50.0 (C^2), 46.0 (C^7), 33.1 (C^4), 22.3 (C^1). Data for the major diastereomer *b*: δ 172.3 (C^6), 142.8 (C^{16}), 129.6 (ArC), 129.1 (ArC), 128.6 (ArC), 128.4 (ArC), 127.9 (ArC), 126.9 (ArC), 125.1 (ArC), 124.4 (ArC), 118.5 (C^{21}), 115.9 (C^{19}), 66.4 (C^8), 46.8 (C^2), 42.7 (C^7), 33.8 (C^4), 19.4 (C^1); **HRMS** (ESI): calculated for $C_{24}H_{24}NO_2$ $[M+H]^+$ requires m/z 358.1802, found m/z 358.1805.

General procedure C for the reductive decarboxylation:

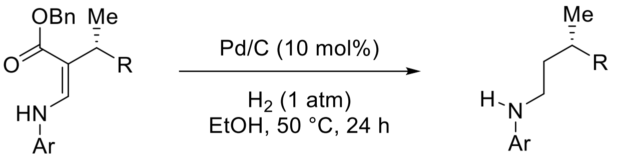

An oven-dried round bottom flask was charged with the deaired product **3ha**, **3af** or **3ao** (0.10 mmol, 100 mol%) and Pd/C (10.6 mg, 0.01 mmol, 10 mol%). The flask was evacuated and refilled with H_2 (three cycles), then EtOH (1.0 mL) were added. After the addition was complete, the reaction mixture was stirred for 24 hours at 50 °C and the progress of the reaction was monitored by TLC. After cooling to r.t., the reaction mixture was filtered and concentrated *in vacuo*. The residue was purified by FCC under the conditions noted.

(*S*)-4-methoxy-*N*-(3-phenylbutyl)aniline (8a):

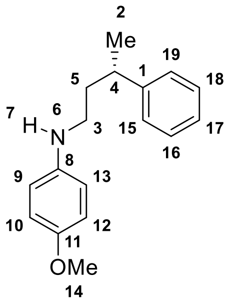

General procedure C: The reaction was carried out with compound **3ha** (38.7 mg, 0.10 mmol, 100 mol%). Purification of the residue by FCC (hexane/EtOAc 90:10) afforded the title compound (24.2 mg, 95%, e.r. = 95:5) as a pale-yellow oil. $[\alpha]_D^{25}$ = 30.8 (c = 1.0, $CHCl_3$); **IR (thin film)** ν_{max}/cm^{-1}: 3325 (br), 2926 (s), 1511 (s), 1233 (s), 1037 (s), 818 (s), 699 (s); **^{1}H NMR** (500 MHz, $CDCl_3$) δ 7.35 – 7.32 (m, 2H, H^{16} + H^{18}), 7.25 – 7.22 (m, 3H, H^{15} + H^{17} + H^{19}), 6.78 (d, *J* = 9.0 Hz, 2H, H^9 + H^{13}), 6.54 (d, *J* = 9.0 Hz, 2H, H^{10} + H^{12}), 3.76 (s, 3H, H^{14}), 3.07 – 2.96 (m, 2H, H^3), 2.91 – 2.84 (m, 1H, H^4), 1.93 (q, *J* = 7.0 Hz, 2H, H^5), 1.32 (d, *J* = 7.0 Hz, 3H, H^2); **^{13}C NMR** (126 MHz, $CDCl_3$) δ 152.3 (C^{11}), 146.7 (C^1), 142.1 (C^8), 128.5 (C^{16} + C^{18}), 126.9 (C^{15} + C^{19}), 126.2 (C^{17}), 114.9 (C^9 + C^{13}), 114.5 (C^{10} + C^{12}), 55.8 (C^{14}), 43.6 (C^3), 38.0 (C^4), 37.9 (C^5), 22.6 (C^2); **HRMS** (ESI): calculated for $C_{17}H_{22}NO$ $[M+H]^+$ requires *m/z* 256.1696, found *m/z* 256.1701; **Chiral SFC:** DAICEL CHIRALCEL OD-H column (25 cm), CO_2:*i*-PrOH 90:10, 1.0 mL/min, 254 nm, 148 bar. Retention times: 22.9 mins (major), 24.8 mins (minor), e.r. = 95:5.

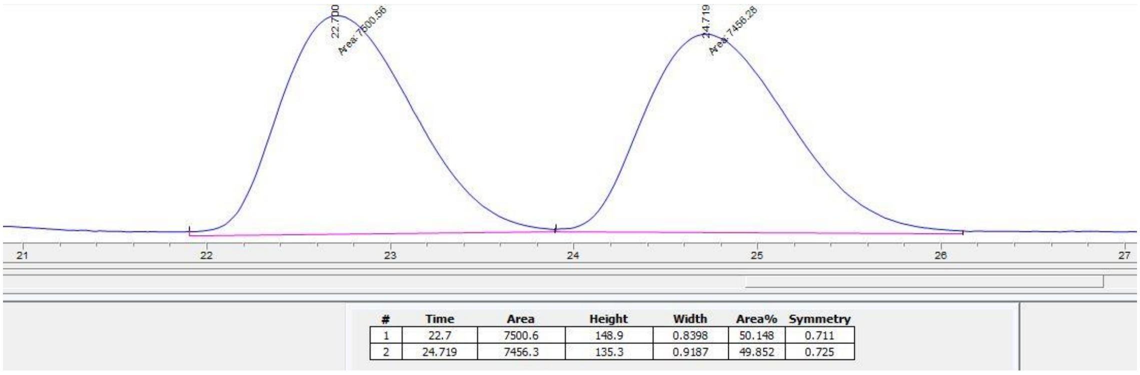

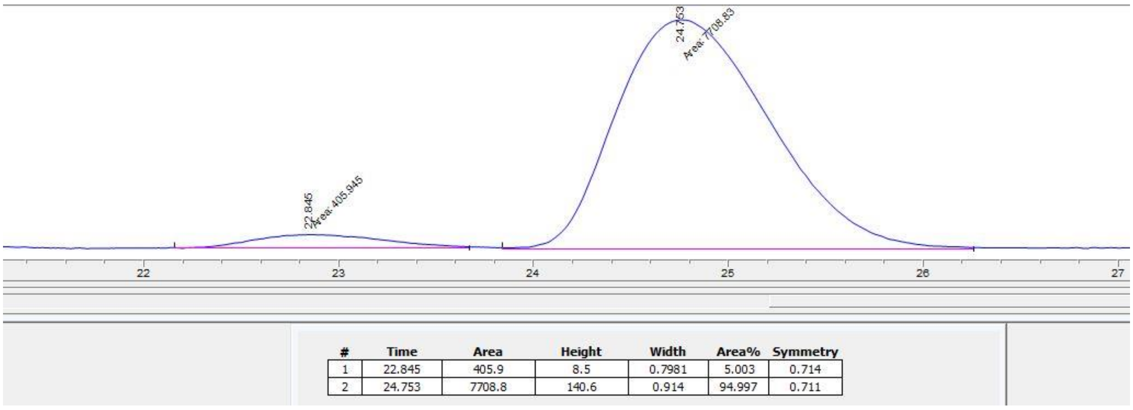

(*S*)-*N*-(3-([1,1'-biphenyl]-4-yl)butyl)aniline (8b):

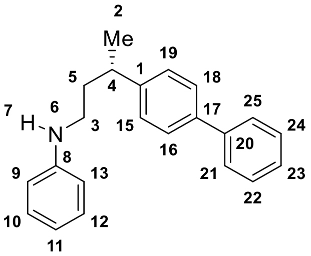

General procedure C: The reaction was carried out with compound **3af** (43.3 mg, 0.10 mmol, 100 mol%). Purification of the residue by FCC (hexane/EtOAc 92:8) afforded the title compound (14.5 mg, 48%, e.r. = 96:4) as a pale-yellow oil. $[\boldsymbol{\alpha}]_{\boldsymbol{D}}^{\mathbf{25}}$ = 34.1 (c = 1.0, $CHCl_3$); **IR (thin film)** ν_{max}/cm^{-1}: 3293 (br), 2923 (s), 2852 (s), 1602 (s), 1486 (s), 1261 (s), 764 (s), 696 (s); **^{1}H NMR** (500 MHz, $CDCl_3$) δ 7.62 (d, *J* = 8.0 Hz, 2H, H^{21} + H^{25}), 7.57 (d, *J* = 8.0 Hz, 2H, H^{15} + H^{19}), 7.48 – 7.45 (m, 2H, H^{22} + H^{24}), 7.38 – 7.33 (m, 1H, H^{23}), 7.32 (d, *J* = 8.0 Hz, 2H, H^{16} + H^{18}), 7.19 – 7.16 (m, 2H, H^{10} + H^{12}), 6.72 – 6.69 (m, 1H, H^{11}), 6.57 (d, *J* = 7.5 Hz, 2H, H^9 + H^{13}), 3.68 (br. s, 1H, H^7), 3.15 – 3.05 (m, 2H, H^3), 2.97 – 2.90 (m, 1H, H^4), 1.93 (q, *J* = 7.0 Hz, 2H, H^5), 1.37 (d, *J* = 7.0 Hz, 3H, H^2); **^{13}C NMR** (126 MHz, $CDCl_3$) δ 148.2 (C^8), 145.8 (C^1), 141.0 (C^{17}), 139.2 (C^{20}), 129.2 (C^{10} + C^{12}), 128.8 (C^{22} + C^{24}), 127.4 (ArC), 127.3 (ArC), 127.1 (C^{23}), 127.0 (ArC),117.3 (C^{11}), 112.8 (C^9 + C^{13}), 42.4 (C^3), 37.9 (C^4), 37.6 (C^5), 22.6 (C^2); **HRMS** (ESl): calculated for $C_{22}H_{24}N$ $[M+H]^+$ requires *m/z* 302.1903, found *m/z*

302.1910; **Chiral SFC:** DAICEL CHIRALCEL IE column (25 cm), CO_2:*i*-PrOH 90:10, 1.0 mL/min, 254 nm, 140 bar. Retention times: 20.4 mins (major), 20.9 mins (minor), e.r. = 96:4.

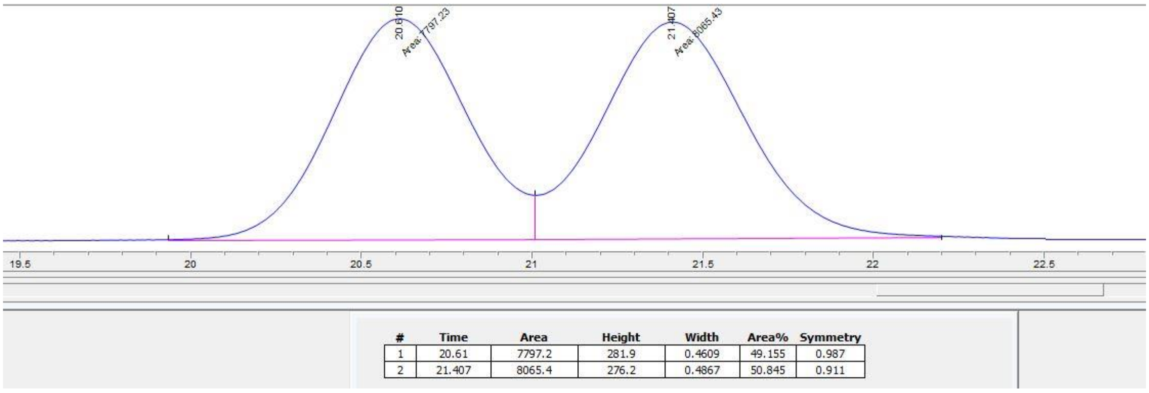

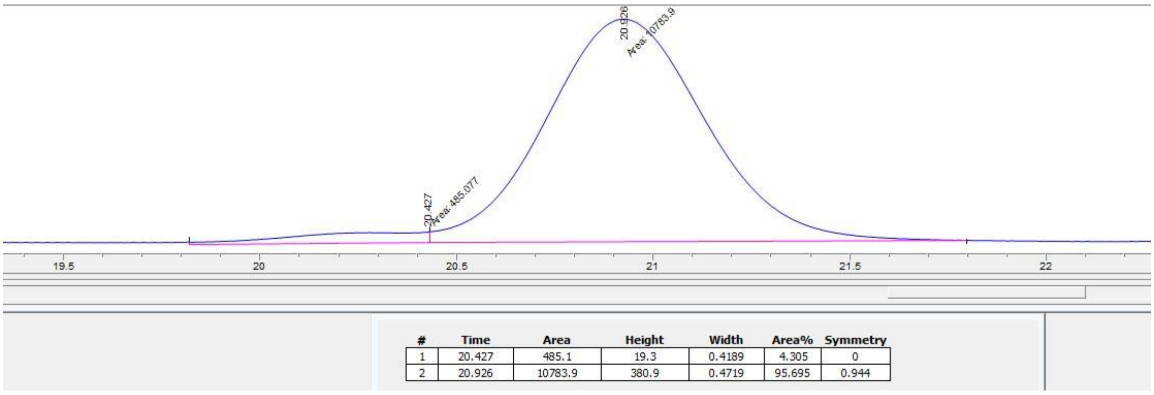

(*S*)-*N*-(3-(1-tosyl-1*H*-indol-3-yl)butyl)aniline (8c):

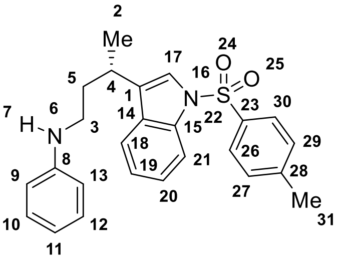

General procedure C: The reaction was carried out with compound **3ao** (55.0 mg, 0.10 mmol, 100 mol%). Purification of the residue by FCC (hexane/EtOAc 85:15) afforded the title compound (28.4 mg, 68%, e.r. = 94:6) as a pale-yellow oil. $[\boldsymbol{\alpha}]_{\boldsymbol{D}}^{\mathbf{25}}$ = 24.4 (c = 1.0, $CHCl_3$); **IR (thin film)** ν_{max}/cm^{-1}: 3412 (br), 2926 (s), 1602 (s), 1447 (s), 1363 (s), 1171 (s), 745 (s), 667 (s); **^{1}H NMR** (500 MHz, $CDCl_3$) δ 8.03 (d, *J* = 8.0 Hz, 1H, H^{18}), 7.76 (d, *J* = 8.5 Hz, 2H, H^{26} + H^{30}), 7.53 (d, *J* = 8.0 Hz, 1H, H^{21}), 7.39 (s, 1H, H^{17}), 7.35 – 7.32 (m, 1H, H^{20}), 7.25 – 7.15 (m, 5H, H^{10} + H^{12} + H^{19} + H^{27} + H^{29}), 6.74 – 6.71 (m, 1H, H^{11}), 6.53 (d, *J* = 9.0 Hz, 2H, H^9 + H^{13}), 3.73 (br. s, 1H, H^7), 3.18 – 3.09 (m, 3H, H^3 + H^4), 2.32 (s, 3H, H^{31}), 2.12 – 2.05 (m, 1H, H^5), 1.99 – 1.92 (m, 1H, H^5), 1.40 (d, *J* = 7.0 Hz, 3H, H^2); **^{13}C NMR** (126 MHz, $CDCl_3$) δ

148.0 (C^{8}), 144.8 (C^{28}), 135.6 (ArC), 135.2 (C^{23}), 130.3 (ArC), 129.8 (C^{10} + C^{12}), 129.2 (C^{27} + C^{29}), 128.1 (C^{1}), 126.7 (C^{26} + C^{30}), 124.7 (ArC), 123.1 (ArC), 122.0 (ArC), 119.8 (ArC), 117.5 (C^{11}), 114.0 (C^{18}), 112.9 (C^{9} + C^{13}), 42.1 (C^{3}), 36.2 (C^{5}), 28.7 (C^{4}), 21.5 (C^{31}), 20.8 (C^{2}); **HRMS** (ESl): calculated for $C_{25}H_{27}N_2O_2S$ $[M+H]^+$ requires *m/z* 419.1788, found *m/z* 419.1798; **Chiral SFC:** DAICEL CHIRALCEL SC column (25 cm), CO_2:*i*-PrOH 80:20, 2.0 mL/min, 254 nm, 177 bar. Retention times: 13.3 mins (major), 14.0 mins (minor), e.r. = 94:6.

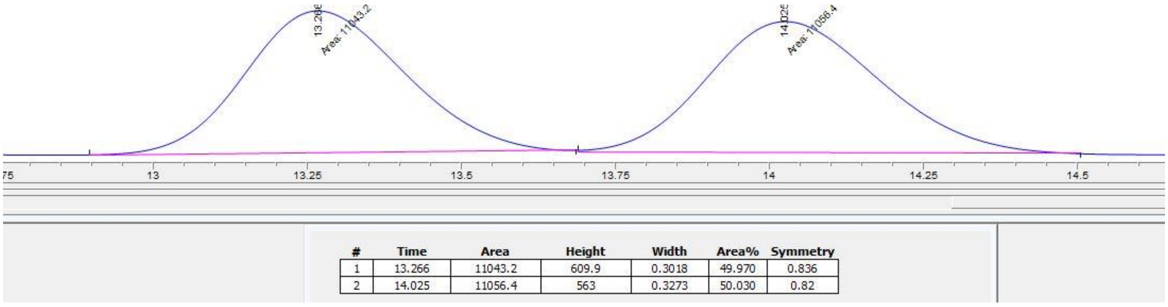

#	Time	Area	Height	Width	Area%	Symmetry
1	13.266	11043.2	609.9	0.3018	49.970	0.836
2	14.025	11056.4	563	0.3273	50.030	0.82

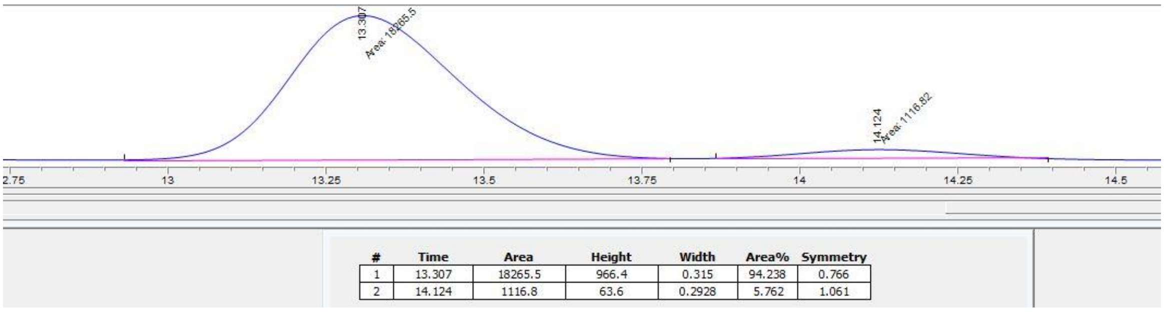

#	Time	Area	Height	Width	Area%	Symmetry
1	13.307	18265.5	966.4	0.315	94.238	0.766
2	14.124	1116.8	63.6	0.2928	5.762	1.061

Evaluation of other protecting groups

Different protecting groups, including benzyl (**1n**) and acetyl (**1o**), have been evaluated and all were less efficient than the NHPh group (**1a**).

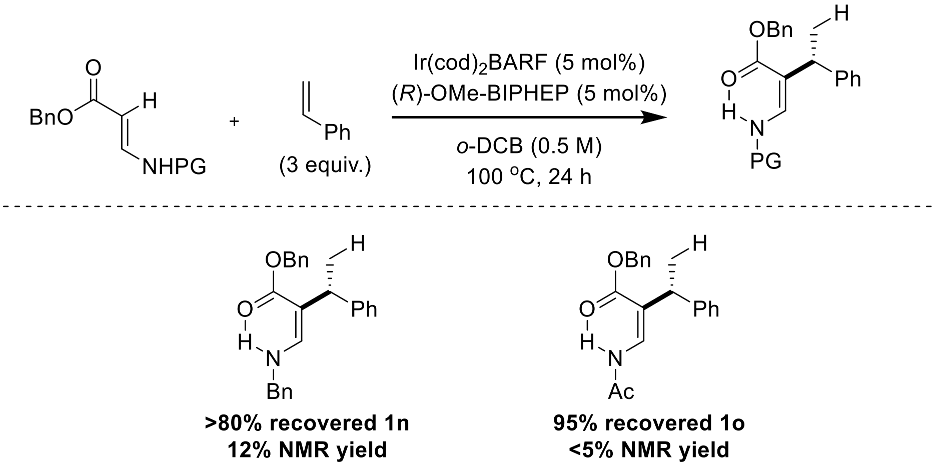

Mechanistic studies

Scheme S1: Control experiments

A series of control experiments have been undertaken to elucidate key requirements for the alkylation process.

Benzyl acrylate **9** (16.2 mg, 0.10 mmol, 100 mol%), Ir(cod)$_2$BARF (6.36 mg, 5.00 μmol, 5 mol%) and *rac*-BINAP (3.11 mg, 5.00 μmol, 5 mol%) were placed in an oven-dried resealable tube equipped with a magnetic stirrer bar and capped with a rubber septum. The tube was evacuated and refilled with N_2 (this operation was repeated three times) and then styrene (34.0 µL, 0.30 mmol, 300 mol%) and *o*-DCB (0.2 mL) were added sequentially. The tube was sealed with a screw cap and heated to 100 °C for 24 hours with vigorous stirring. Upon completion, the solvent was removed under reduced pressure and the crude mixture was purified by FCC (hexane/EtOAc 90:10) to recover benzyl acrylate **9**. *The control experiment shows that the process requires an NHAr unit.*

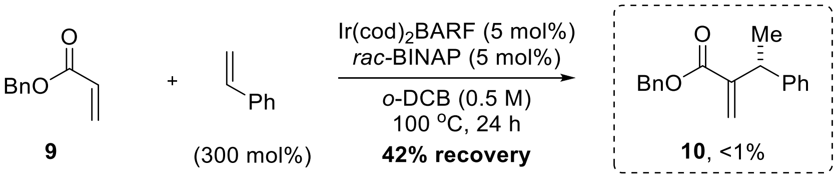

Methyl (*E*)-3-(methyl(phenyl)amino)acrylate **1m** (19.1 mg, 0.10 mmol, 100 mol%), [Ir(cod)$_2$]BARF (6.36 mg, 5.00 μmol, 5 mol%) and *rac*-BINAP (3.11 mg, 5.00 μmol, 5 mol%) were placed in an oven-dried resealable tube equipped with a magnetic stirrer bar and capped with a rubber septum. The tube was evacuated and refilled with N_2 (this operation was repeated three times) and then styrene (34.0 µL, 0.30 mmol, 300 mol%) and *o*-DCB (0.2 mL) were added sequentially. The tube was sealed with a screw cap and heated to 100 °C for 24 hours with vigorous stirring. Upon completion, the solvent was removed under reduced pressure and the crude mixture was purified by FCC (hexane/EtOAc 95:5) to recover amide **1m**. *The control experiment shows that the process requires an NH unit.*

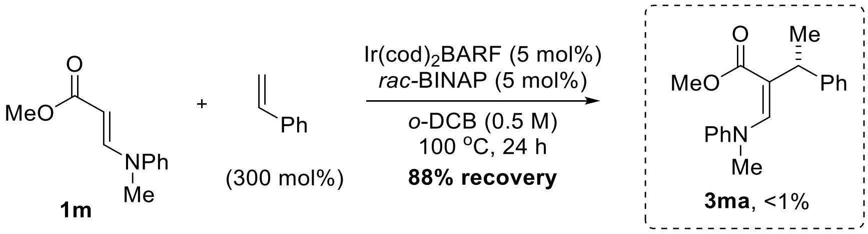

Scheme S2: Deuterium exchange and labelling experiments

Substrate **1a** (25.3 mg, 0.10 mmol, 100 mol%), [Ir(cod)$_2$]BARF (6.36 mg, 5.00 µmol, 5 mol%) and *rac*-BINAP (3.11 mg, 5.00 µmol, 5 mol%) were placed in an oven-dried resealable tube equipped with a magnetic stirrer bar and capped with a rubber septum. The tube was evacuated and refilled with N_2 (this operation was repeated three times) and then *o*-DCB (0.2 mL) and D_2O (18.0 µL, 1.00 mmol, 1000 mol%) were added sequentially. The tube was sealed with a screw cap and heated to 100 °C for 24 hours with vigorous stirring. Upon completion, the solvent was removed under reduced pressure and the crude mixture was purified by FCC (hexane/EtOAc 90:10) to recover substrate **1a**. Deuterium incorporation was calculated by integration of the ^{1}H NMR spectrum.

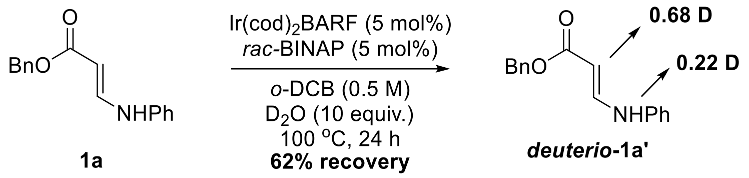

Substrate **1a** (25.3 mg, 0.10 mmol, 100 mol%) and *rac*-BINAP (3.11 mg, 5.00 µmol, 5 mol%) were placed in an oven-dried resealable tube equipped with a magnetic stirrer bar and capped with a rubber septum. The tube was evacuated and refilled with N_2 (this operation was repeated three times) and then *o*-DCB (0.2 mL) and D_2O (18.0 µL, 1.00 mmol, 1000 mol%) were added sequentially. The tube was sealed with a screw cap and heated to 100 °C for 24 hours with vigorous stirring. Upon completion, the solvent was removed under reduced pressure and the crude mixture was purified by FCC (hexane/EtOAc 90:10) to recover substrate **1a**. Deuterium incorporation was calculated by integration of the ^{1}H NMR spectrum.

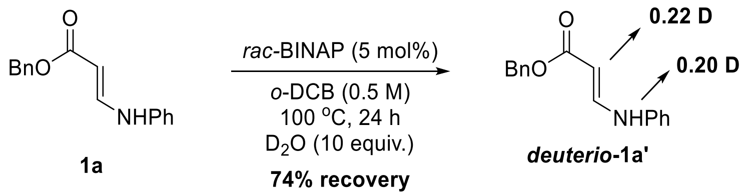

Substrate **1m** (19.1 mg, 0.10 mmol, 100 mol%), [Ir(cod)$_2$]BARF (6.36 mg, 5.00 µmol, 5 mol%) and *rac*-BINAP (3.11 mg, 5.00 µmol, 5 mol%) were placed in an oven-dried resealable tube equipped with a magnetic stirrer bar and capped with a rubber septum. The tube was evacuated and refilled with N_2 (this operation was repeated three times) and then *o*-DCB (0.2 mL) and D_2O (18.0 µL, 1.00 mmol, 1000 mol%) were added sequentially. The tube was sealed with a screw cap and heated to 100 °C for 24 hours with vigorous stirring. Upon completion, the

solvent was removed under reduced pressure and the crude mixture was purified by FCC (hexane/EtOAc 90:10) to recover substrate **1m**. Deuterium incorporation was calculated by integration of the ^{1}H NMR spectrum.

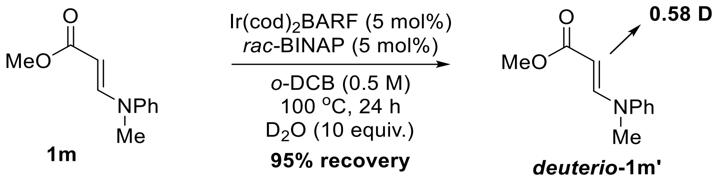

Substrate **1m** (19.1 mg, 0.10 mmol, 100 mol%) and *rac*-BINAP (3.11 mg, 5.00 µmol, 5 mol%) were placed in an oven-dried resealable tube equipped with a magnetic stirrer bar and capped with a rubber septum. The tube was evacuated and refilled with N_2 (this operation was repeated three times) and then *o*-DCB (0.2 mL) and D_2O (18.0 µL, 1.00 mmol, 1000 mol%) were added sequentially. The tube was sealed with a screw cap and heated to 100 °C for 24 hours with vigorous stirring. Upon completion, the solvent was removed under reduced pressure and the crude mixture was purified by FCC (hexane/EtOAc 90:10) to recover substrate **1m**. Deuterium incorporation was calculated by integration of the ^{1}H NMR spectrum.

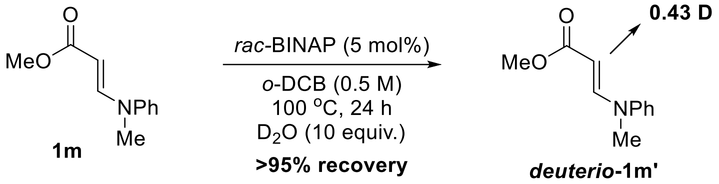

Substrate **1a** (25.3 mg, 0.10 mmol, 100 mol%), [Ir(cod)$_2$]BARF (6.36 mg, 5.00 µmol, 5 mol%), *rac*-BINAP (3.11 mg, 5.00 µmol, 5 mol%) and ***deuterio*-2f**[15] (54.6 mg, 0.30 mmol, 300 mol%) were placed in an oven-dried resealable tube equipped with a magnetic stirrer bar and capped with a rubber septum. The tube was evacuated and refilled with N_2 (this operation was repeated three times) and then *o*-DCB (0.2 mL) was added. The tube was sealed with a screw cap and heated to 100 °C for 24 hours with vigorous stirring. Upon completion, the solvent was removed under reduced pressure and the crude mixture was purified by FCC (hexane/EtOAc 95:5) to obtain two fractions.

- F_1 (Rf = 0.9) contained impure recovered ***deuterio*-2f'**.
- F_2 (Rf = 0.3) contained pure product ***deuterio*-3af'** (22.5 mg, 52%).

Deuterium incorporation was calculated by integration of the ^{1}H NMR spectra.

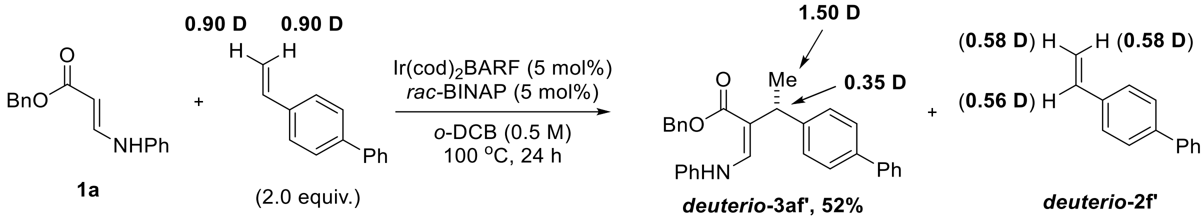

Figure S2: Kinetic experiment

Procedure:

Two oven-dried resealable tubes were charged with substrate **1a** (102 mg, 0.40 mmol, 100 mol%), [Ir(cod)$_2$]BARF (15.3 mg, 12.0 µmol, 3 mol% and 25.4 mg, 20.0 µmol, 5 mol%) and *R*-OMe-BIPHEP (6.99 mg, 12.0 µmol, 3 mol% and 11.7 mg, 20.0 µmol, 5 mol%), respectively. These tubes were evacuated and refilled with N_2 (this operation was repeated three times) and then styrene (138 µL, 1.20 mmol, 300 mol%) and *o*-DCB (0.8 mL) were added, respectively. A portion of each stock solution was transferred to four Schlenk tubes (220 µL in each) under nitrogen. These tubes were sealed with a screw cap, placed into a preheated heating block at 100 °C and stirred for the indicated time. The reaction mixture was cooled to r.t. and then an internal standard (1,3,5-trimethoxybenzene, 16.8 mg) was added. The mixture was concentrated *in vacuo*. The concentration of substrate **1a** was calculated by integration of the ^{1}H NMR spectrum.

Method:

The order in catalyst has been determined applying the Variable Time Normalization Graphical Analysis (VTNGA).[18] Different concentrations of catalyst (3 mol% and 5 mol%, respectively) were used and the concentration of substrate **1a** was plotted against $t[Ir]^n$ where n is the partial order in iridium catalyst, and t is the reaction time. The order in iridium catalyst is that value of n that causes the curves to overlay. The overlap between the temporal reaction profiles with catalyst loadings of 3 mol% and 5 mol% indicates that the order in iridium catalyst is approximately 1.

Experiment 1 [A] 0.510; [Cat] =	0.025	5 mol%	[Ir] (0.0025 M)
time (h)	[A]	[cat]^n	t[cat]^n
0	0.51	0.025	0
1	0.479	0.025	0.025
4	0.325	0.025	0.1
10	0.255	0.025	0.25

Experiment 3 [A] 0.525; [Cat] =	0.015	3 mol%	[Ir] (0.015 M)
	[A]	[cat]^n	t[cat]^n
0	0.525	0.015	0
1	0.489	0.015	0.015
4	0.357	0.015	0.06
10	0.278	0.015	0.15

Order in cat = **1** change this value to check other orders

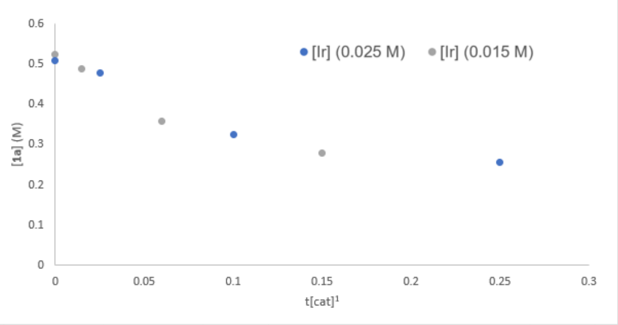

Experiment 1 [A] 0.510; [Cat] =	0.025	5 mol%	[Ir] (0.0025 M)
time (h)	[A]	[cat]^n	t[cat]^n
0	0.51	0.003953	0
1	0.479	0.003953	0.003953
4	0.325	0.003953	0.015811
10	0.255	0.003953	0.039528

Experiment 3 [A] 0.525; [Cat] =	0.015	3 mol%	[Ir] (0.015 M)
	[A]	[cat]^n	t[cat]^n
0	0.525	0.001837	0
1	0.489	0.001837	0.001837
4	0.357	0.001837	0.007348
10	0.278	0.001837	0.018371

Order in cat = **1.5** change this value to check other orders

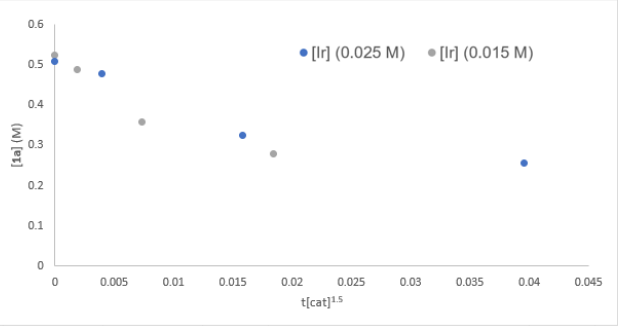

Experiment 1 [A] 0.510; [Cat] =	0.025	5 mol%	[Ir] (0.0025 M)
time (h)	[A]	[cat]^n	t[cat]^n
0	0.51	0.000625	0
1	0.479	0.000625	0.000625
4	0.325	0.000625	0.0025
10	0.255	0.000625	0.00625

Experiment 3 [A] 0.525; [Cat] =	0.015	3 mol%	[Ir] (0.015 M)
	[A]	[cat]^n	t[cat]^n
0	0.525	0.000225	0
1	0.489	0.000225	0.000225
4	0.357	0.000225	0.0009
10	0.278	0.000225	0.00225

Order in cat = **2** change this value to check other orders

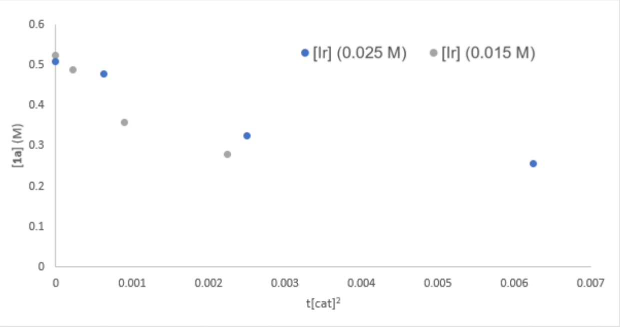

References

1. Tsuchikama, K.; Kasagawa, M.; Endo, K.; Shibata, T. Cationic Ir(I)-Catalyzed sp^3 C−H Bond Alkenylation of Amides with Alkynes. *Org. Lett.* **2009**, *11*, 1821−1823.

2. Dervisi, A.; Carcedo, C.; Ooi, L.-L. Chiral Diphosphine Ddppm-Iridium Complexes: Effective Asymmetric Imine Hydrogenations at Ambient Pressures. *Adv. Synth. Catal.* **2006**, *348*, 175−183.
3. Choi, J.-H; Park, C.-M. Three-Component Synthesis of Quinolines Based on Radical Cascade Visible-Light Photoredox Catalysis. *Adv. Synth. Catal.* **2018**, *360*, 3553−3562.
4. Nayal, O. S.; Thakur, M. S.; Kumar, M.; Shaifali; Upadhyay, R.; Maurya, S. K. Sustainable and Efficient CuI-NPs-Catalyzed Cross-Coupling Approach for the synthesis of Tertiary 3-Aminopropenoates, Triazoles, and Ciprofloxacin. *Asian J. Org. Chem.* **2018**, *7*, 776−780.
5. Reyno, R. S.; Sugunan, A.; Ranganayakuluet, S. A Method for the Preparation of β-Amino-α,β-unsaturated Carbonyl Compounds: Study of Solvent Effect and Mechanism. *Org. Lett.* **2020**, *22*, 1040−1045.
6. Xie, Y.-B.; Ye, S.-P.; Chen, W.-F.; Hu, Y.-L.; Li, D.-J.; Wang, L. Brønsted-Acid-Catalyzed Multicomponent One-Pot Reaction: Efficient Synthesis of Polysubstituted 1,2-Dihydropyridines. *Asian J. Org. Chem.* **2017**, *6*, 746−750.
7. Choi, S.; Park, J.; Yu, E.; Sim, J.; Park, C.-M. Electrosynthesis of Dihydropyrano[4,3-*b*]indoles Based on a Double Oxidative [3 + 3] Cycloaddition. *Angew. Chem., Int. Ed.* **2020**, *59*, 11886−11891.
8. Aleksić, J., Stojanović, M., Bošković, J., Baranac-Stojanović, M. Solid-State Silica Gel-Catalyzed Synthesis of Fluorescent Polysubstituted 1,4- and 1,2-Dihydropyridines. *Org. Biomol. Chem.*, **2023**, *21*, 1187−1205.
9. Liu, Y.; Li, D.; Park, C.-M. Stereoselective Synthesis of Highly Substituted Enamides by an Oxidative Heck Reaction. *Angew. Chem., Int. Ed.* **2011**, *50*, 7333−7336.
10. Hong, F. L.; Aldhous, T. P.; Kemmitt, P. D.; Bower, J. F. A Directed Enolization Strategy Enables the Byproduct Free Construction of Contiguous Stereocenters en route to Complex Amino Acids. *Nat. Chem.* **2024**, DOI:10.1038/s41557-024-01473-5.
11. Lin, C.; Gitsov, I. Preparation and Characterization of Novel Amphiphilic Hydrogels with Covalently Attached Drugs and Fluorescent Markers. *Macromolecules* **2010**, *43*, 10017−10030.

12. Maity, S.; Kancherla, R.; Dhawa, U.; Hoque, E.; Pimparkar, S.; Maiti, D. Switch to Allylic Selectivity in Cobalt-Catalyzed Dehydrogenative Heck Reactions with Unbiased Aliphatic Olefins. *ACS Catal.* **2016**, *6*, 5493−5499.
13. Crespin, L.; Biancalana, L.; Morack, T.; Blakemore, D. C.; Ley, S. V. One-Pot Acid-Catalyzed Ring-Opening/Cyclization/Oxidation of Aziridines with *N*-Tosylhydrazones: Access to 1,2,4-Triazines. *Org. Lett.* **2017**, *19*, 1084−1087.
14. Shu, C.; Hu, X.-Y; Li, S.-S; Yuan, W.-C; Zhang, X.-M. Lewis Base Catalyzed Asymmetric Hydrosilylation of α-Substituted β-Enamino Esters: Facile Access to Enantioenriched β^2-Amino Esters via Dynamic Kinetic Resolution. *Synlett* ***2014**, 25,* 1879−1882.
15. Zhao, Z.; Racicot, L.; Murphy, G. K. Fluorinative Rearrangements of Substituted Phenylallenes Mediated by (Difluoroiodo)toluene: Synthesis of α-(Difluoromethyl)styrenes. *Angew. Chem., Int. Ed.* **2017**, *56*, 11620−11623.
16. Burés, J. A Simple Graphical Method to Determine the Order in Catalyst. *Angew. Chem., Int. Ed.* **2016**, *55*, 2028−2031.

NMR Spectra

Benzyl (*E*)-3-(phenylamino)acrylate (1a):

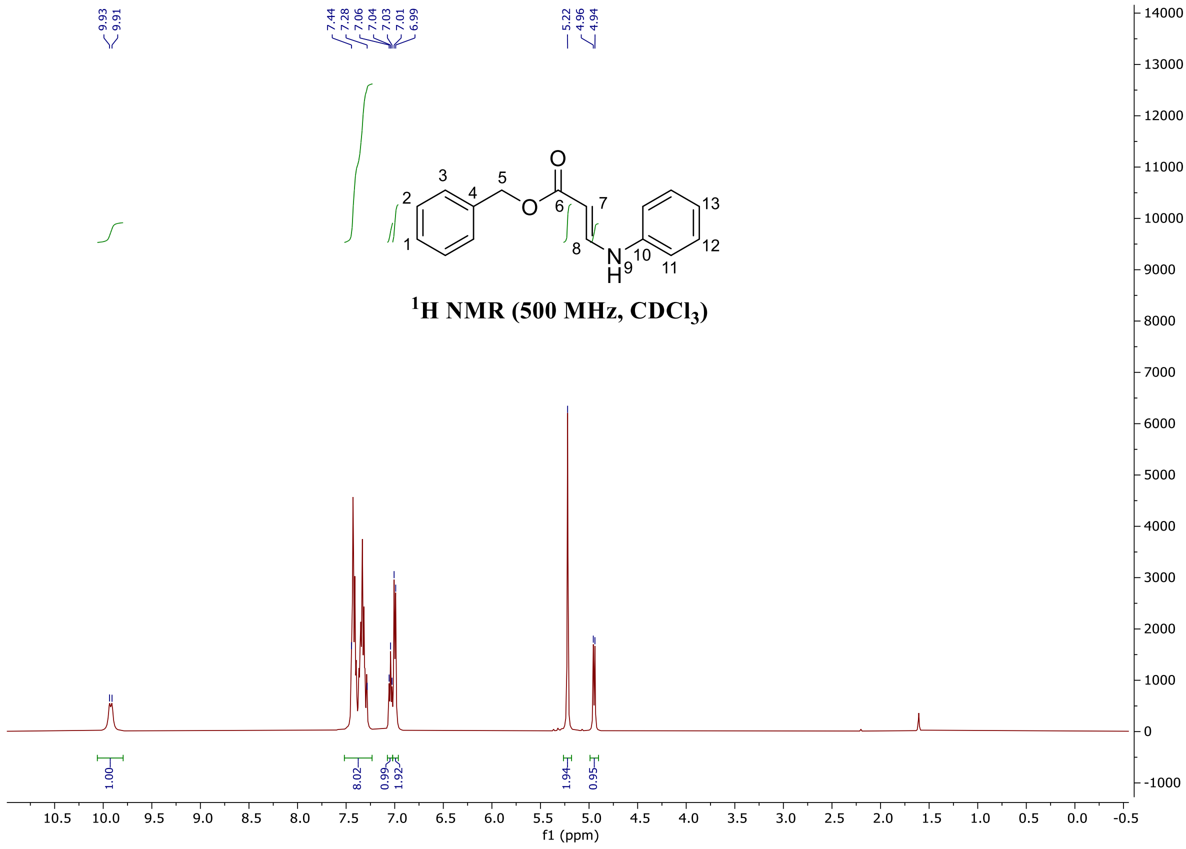

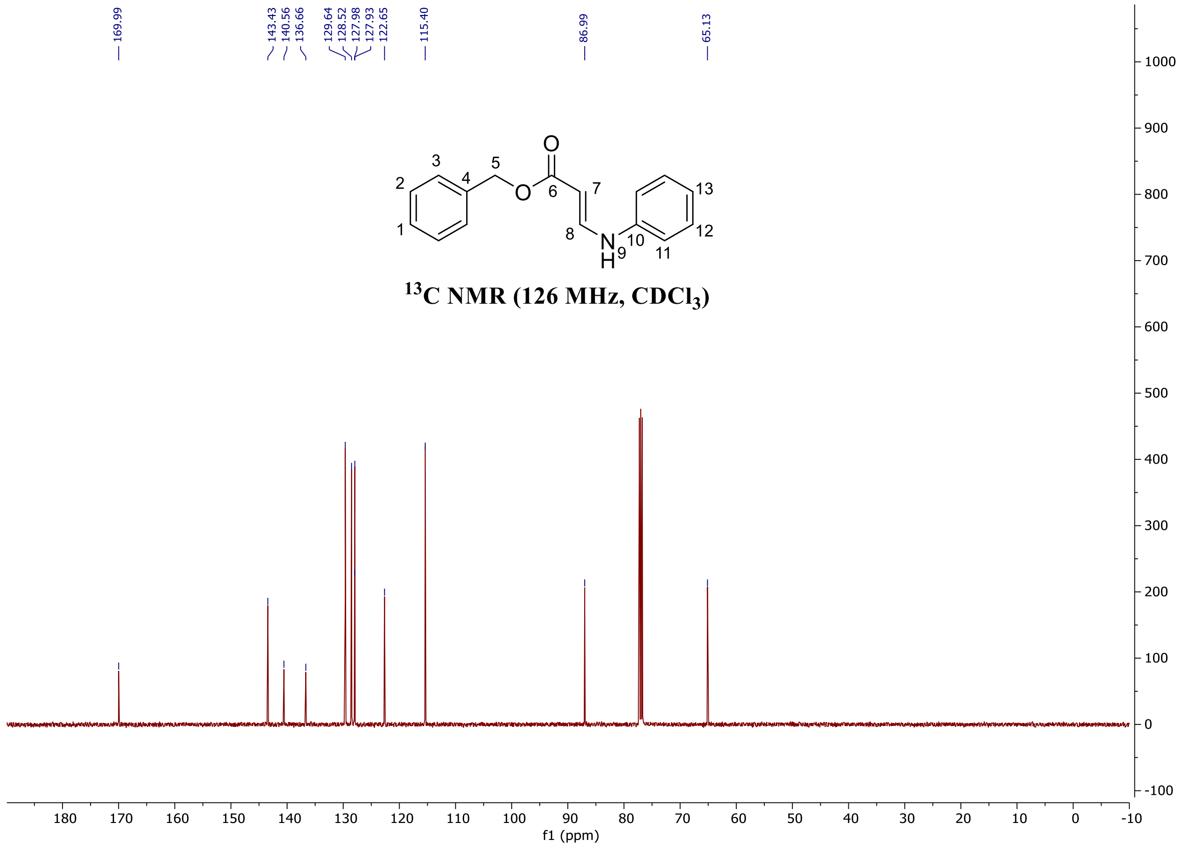

Methyl (*E*)-3-(phenylamino)acrylate (1b):

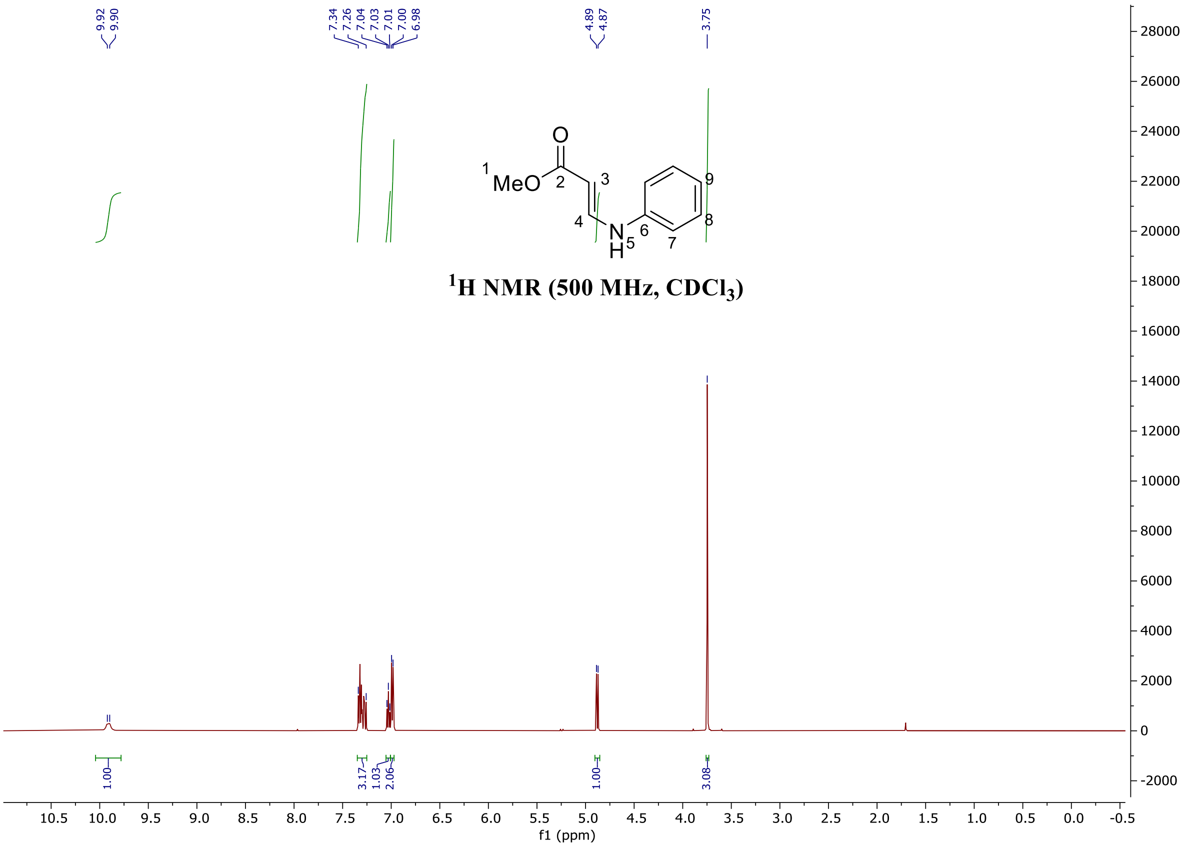

Ethyl (*E*)-3-(phenylamino)acrylate (1c):

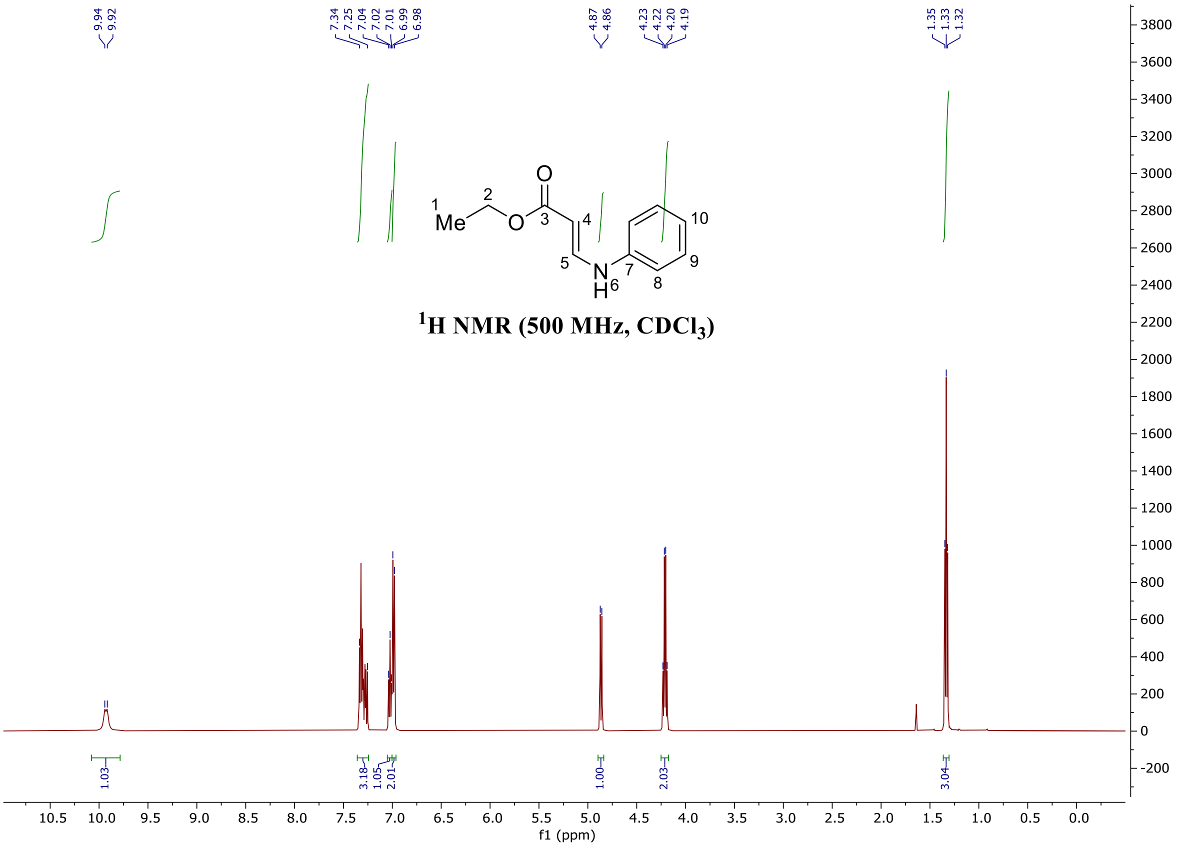

Isopropyl (*E*)-3-(phenylamino)acrylate (1d):

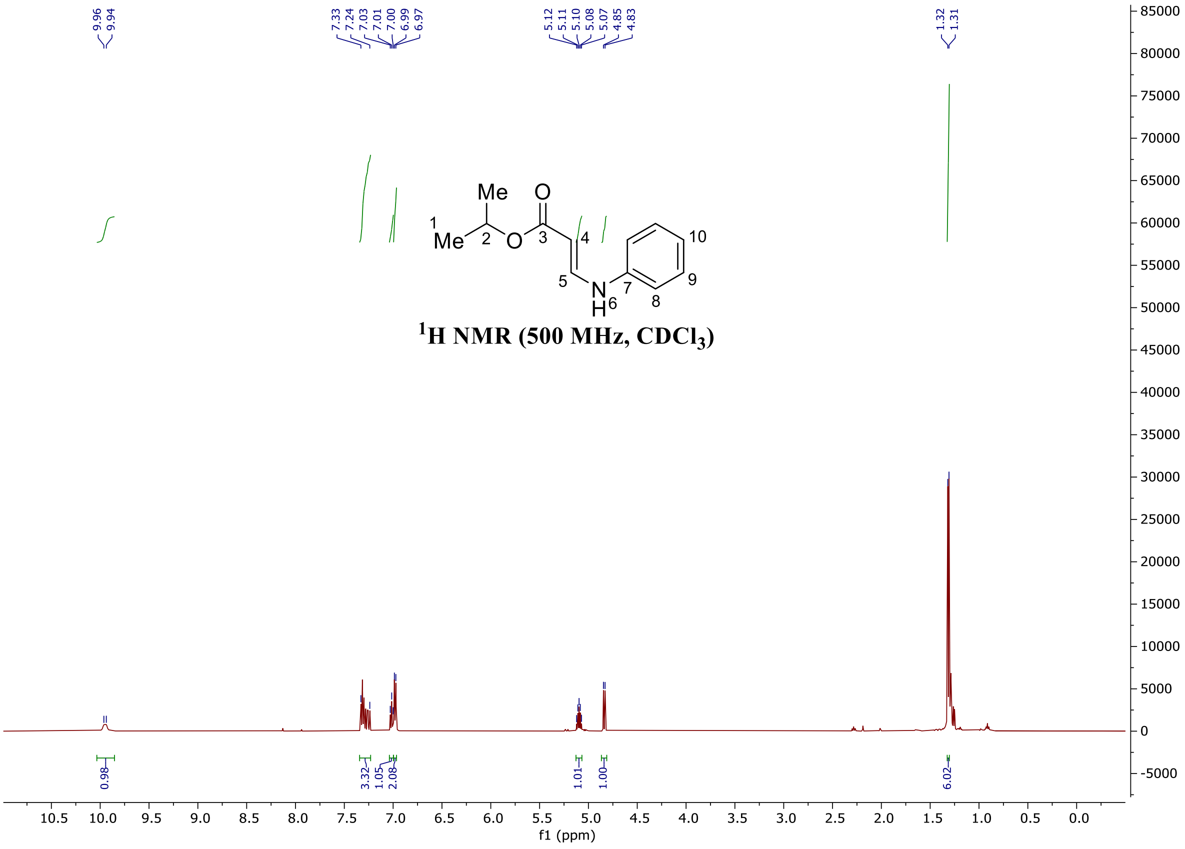

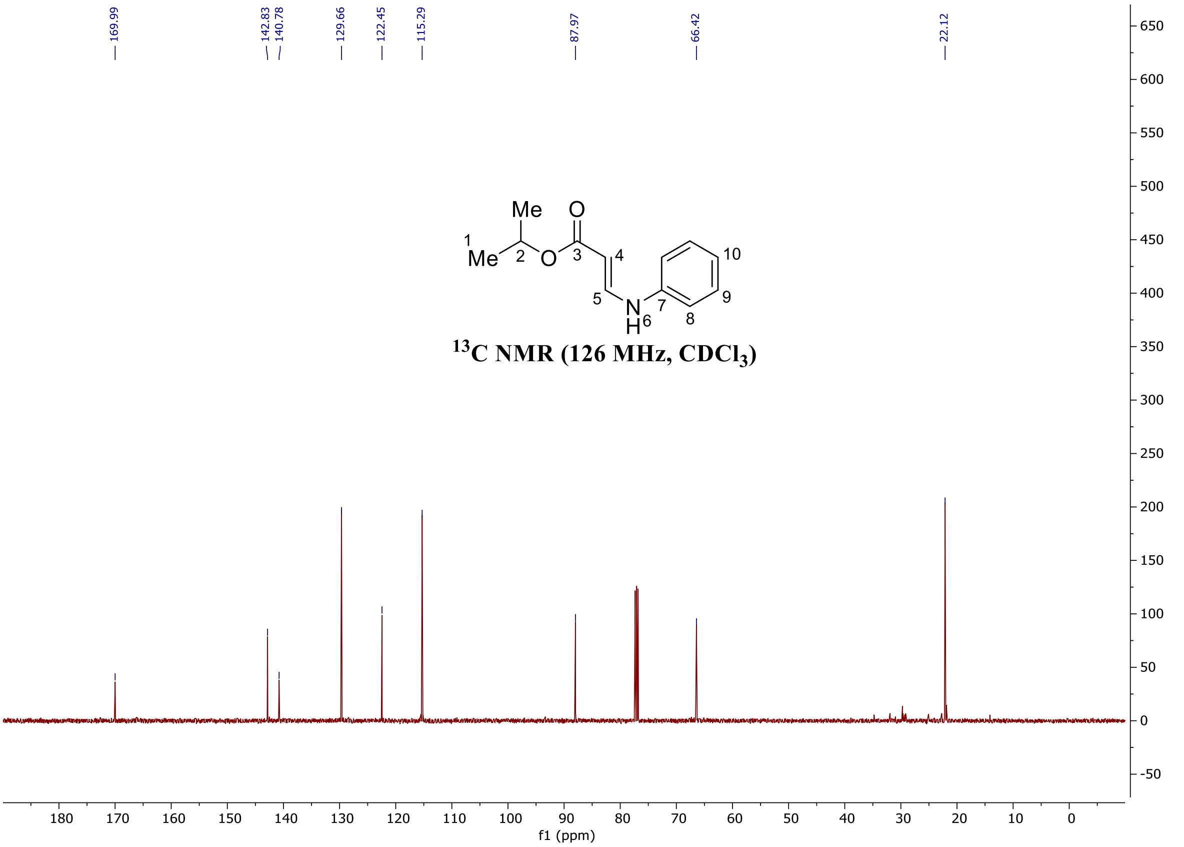

(*E*)-1-Phenyl-3-(phenylamino)prop-2-en-1-one (1e):

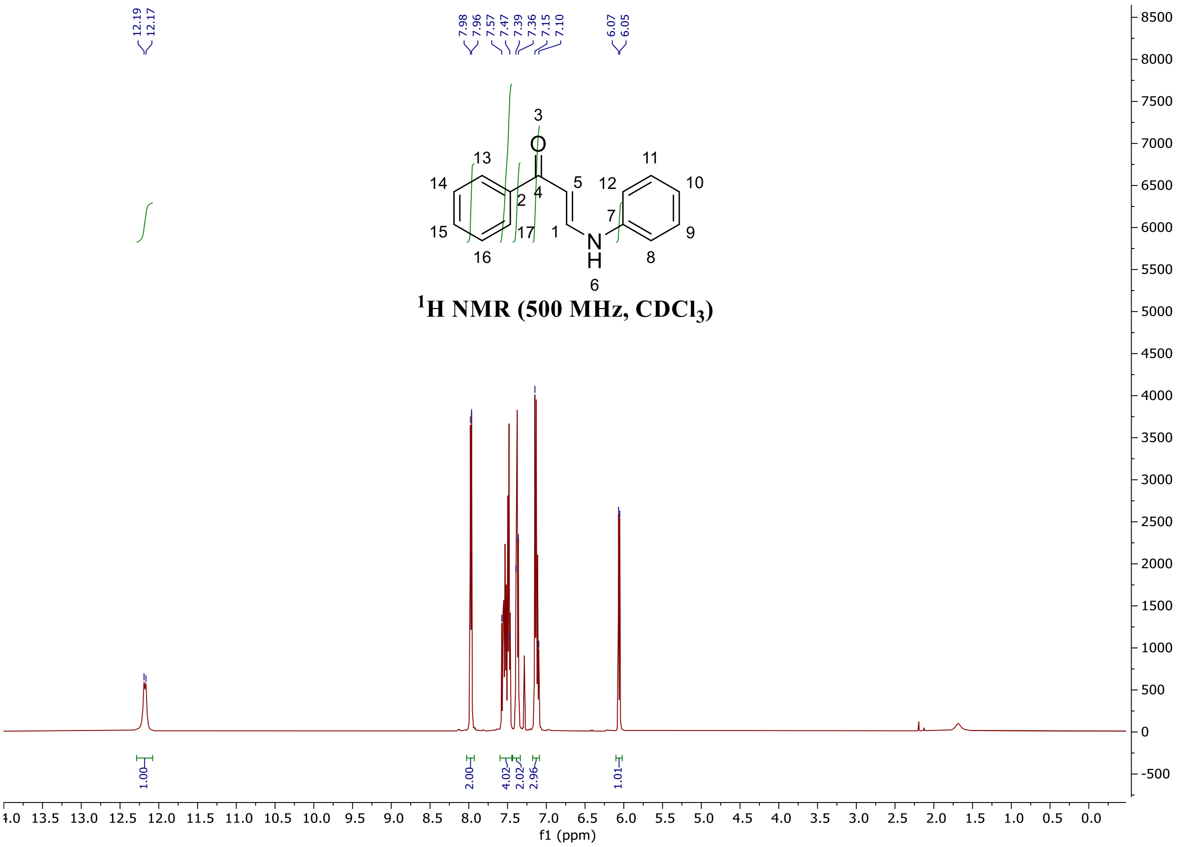

(*E*)-3-(Phenylamino)-1-(pyrrolidin-1-yl)prop-2-en-1-one (1f):

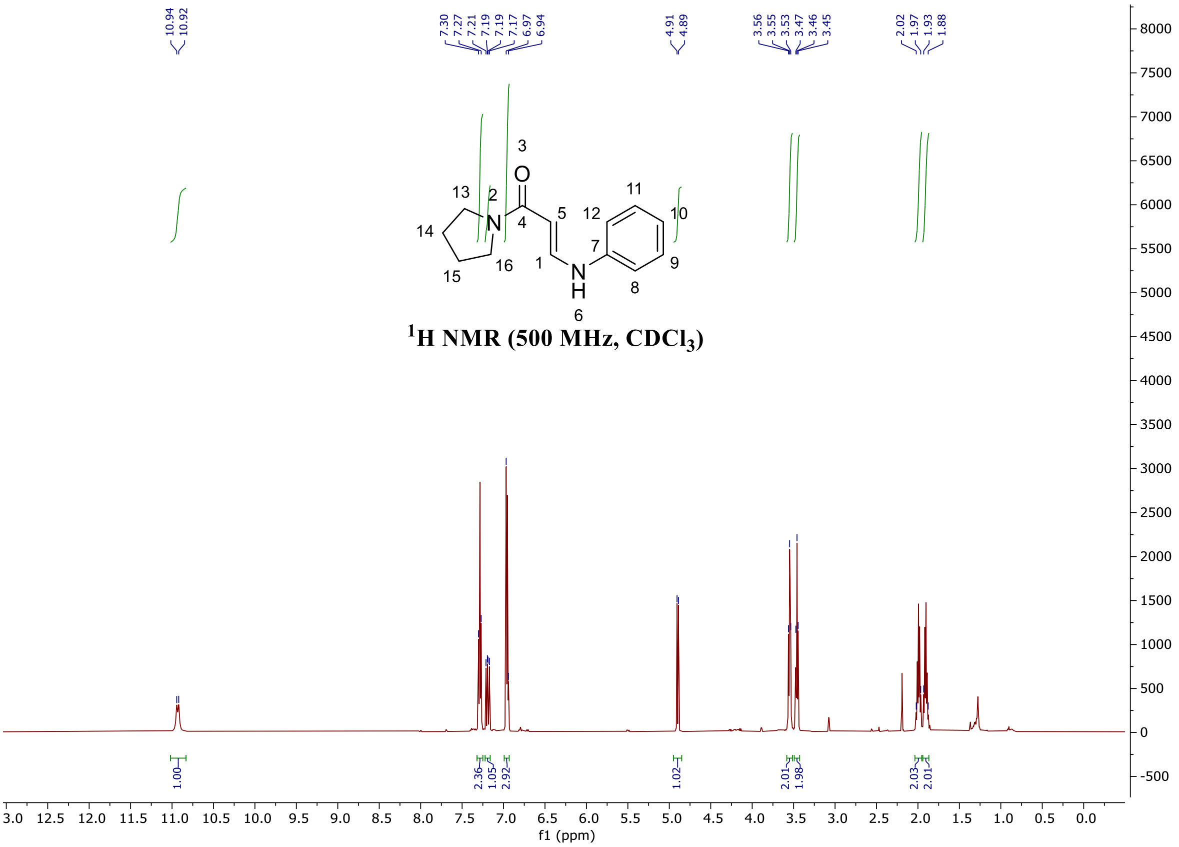

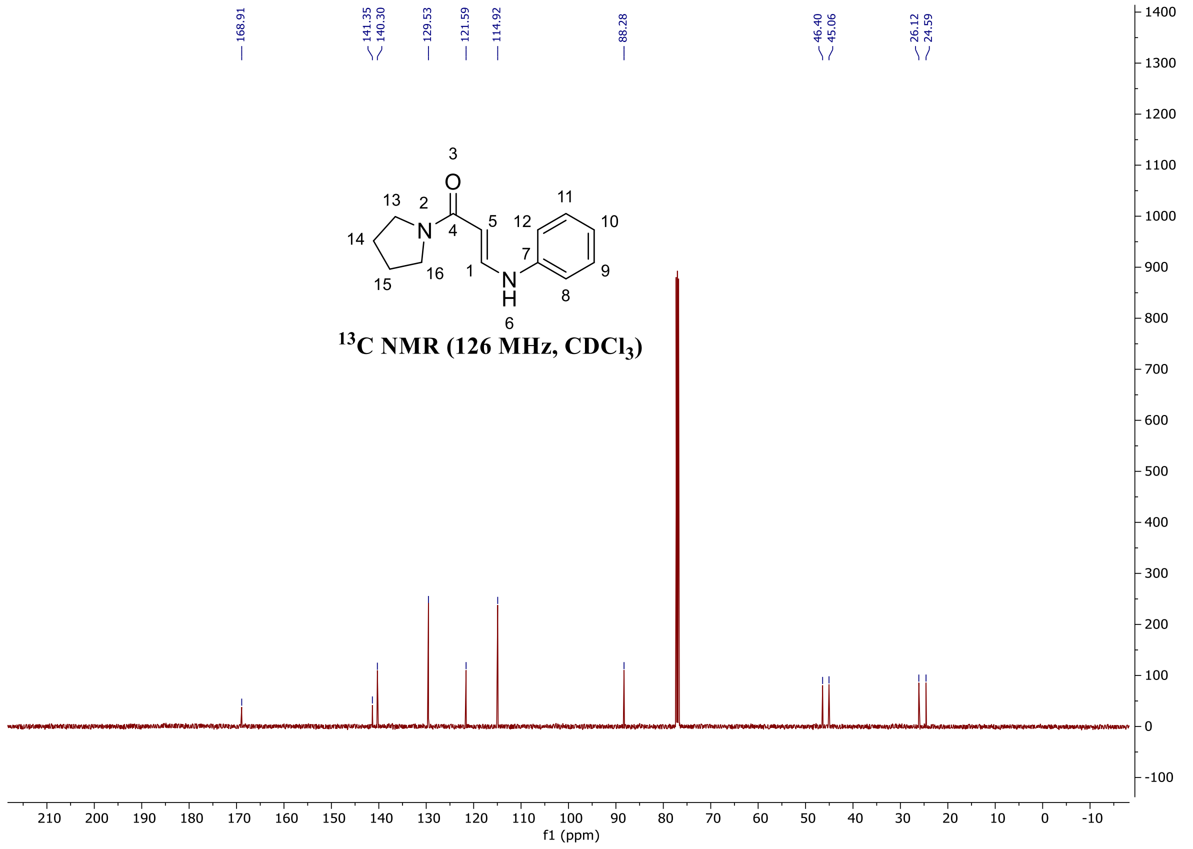

Benzyl (*E*)-3-(*p*-tolylamino)acrylate (1g):

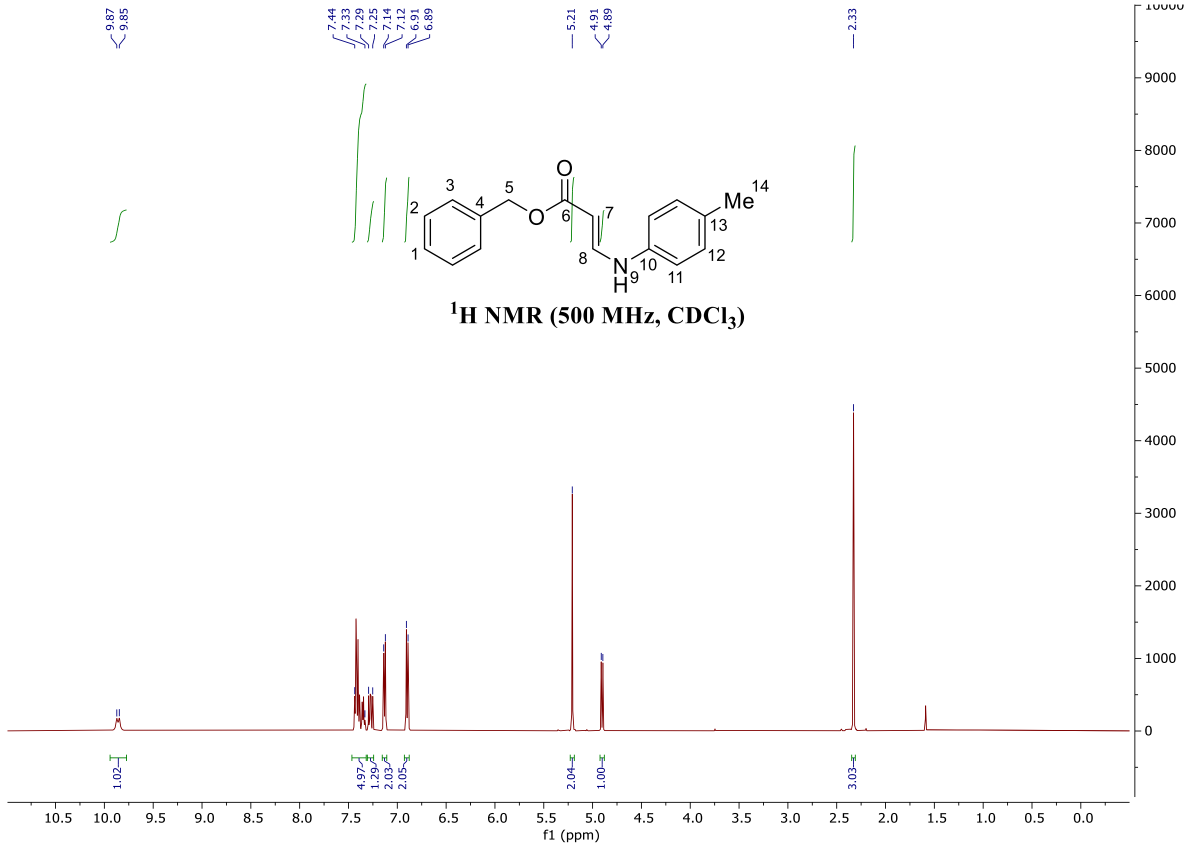

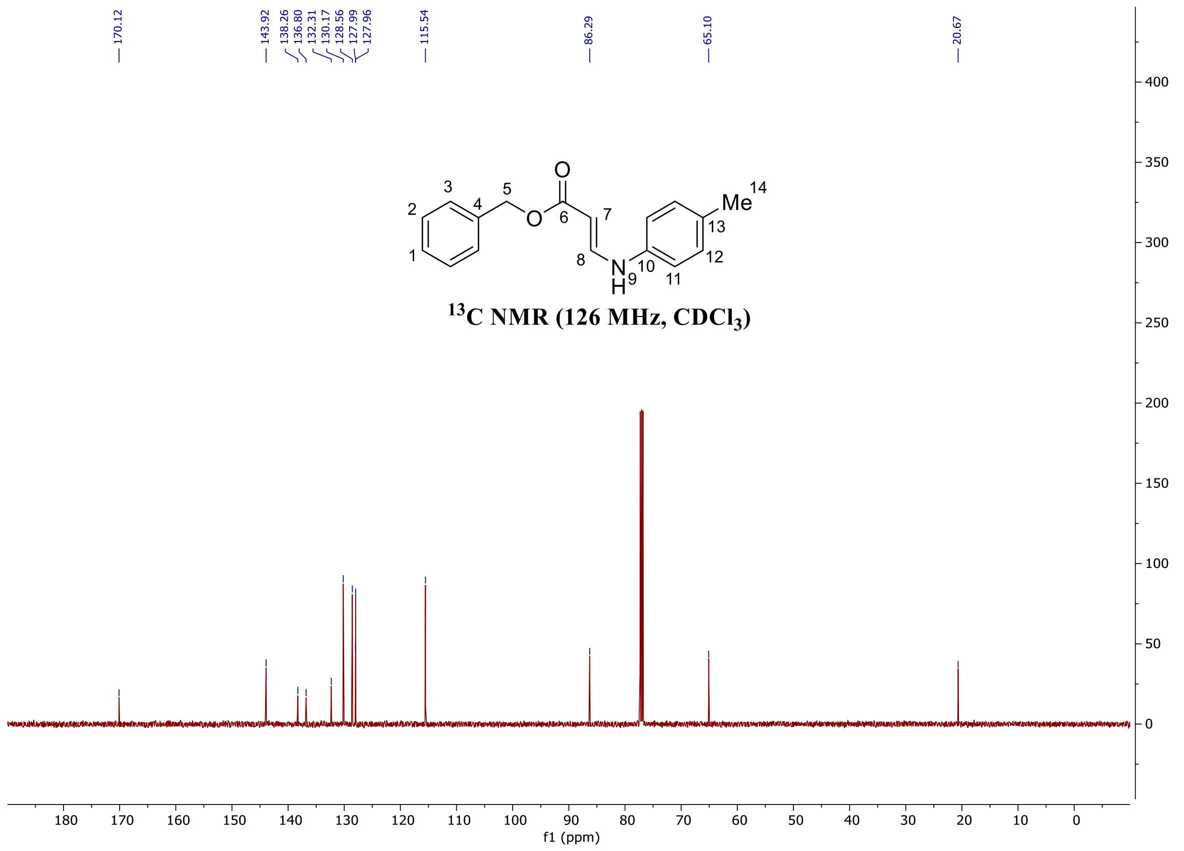

Benzyl (*E*)-3-((4-methoxyphenyl)amino)acrylate (1h):

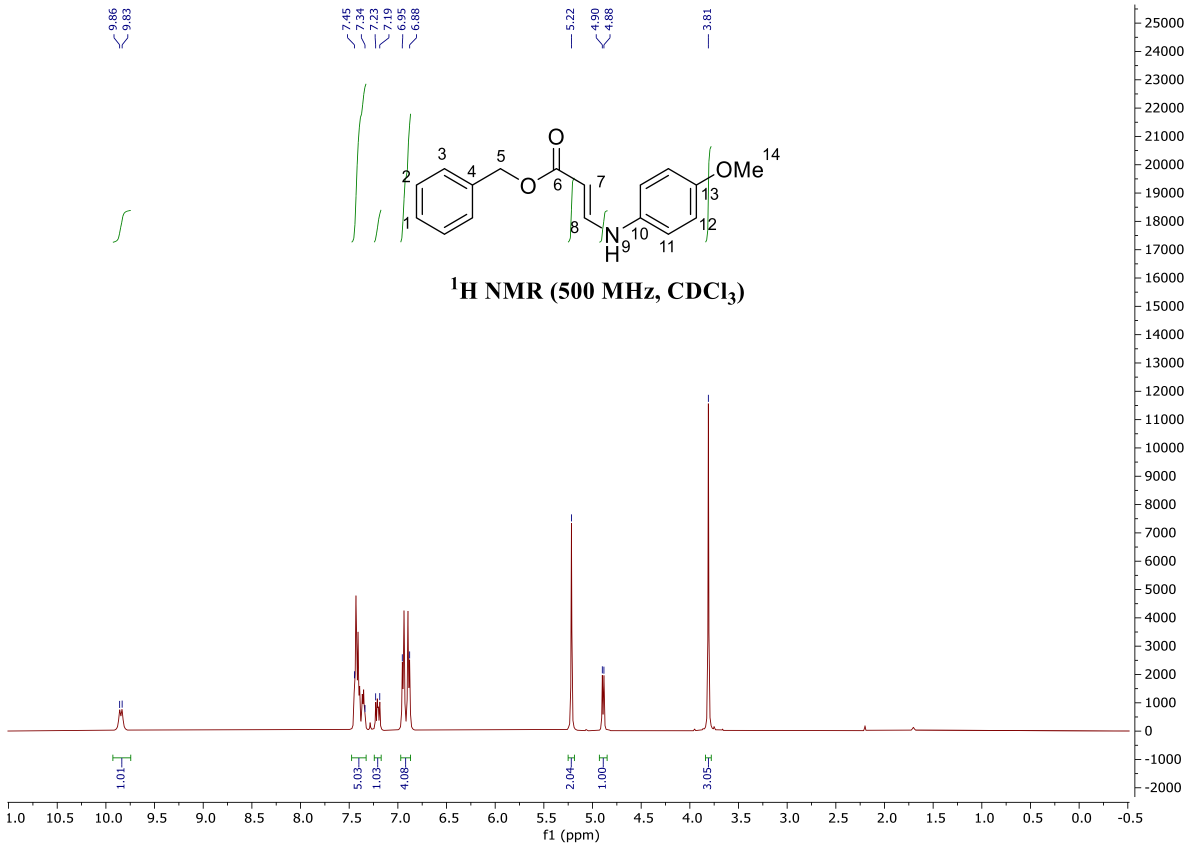

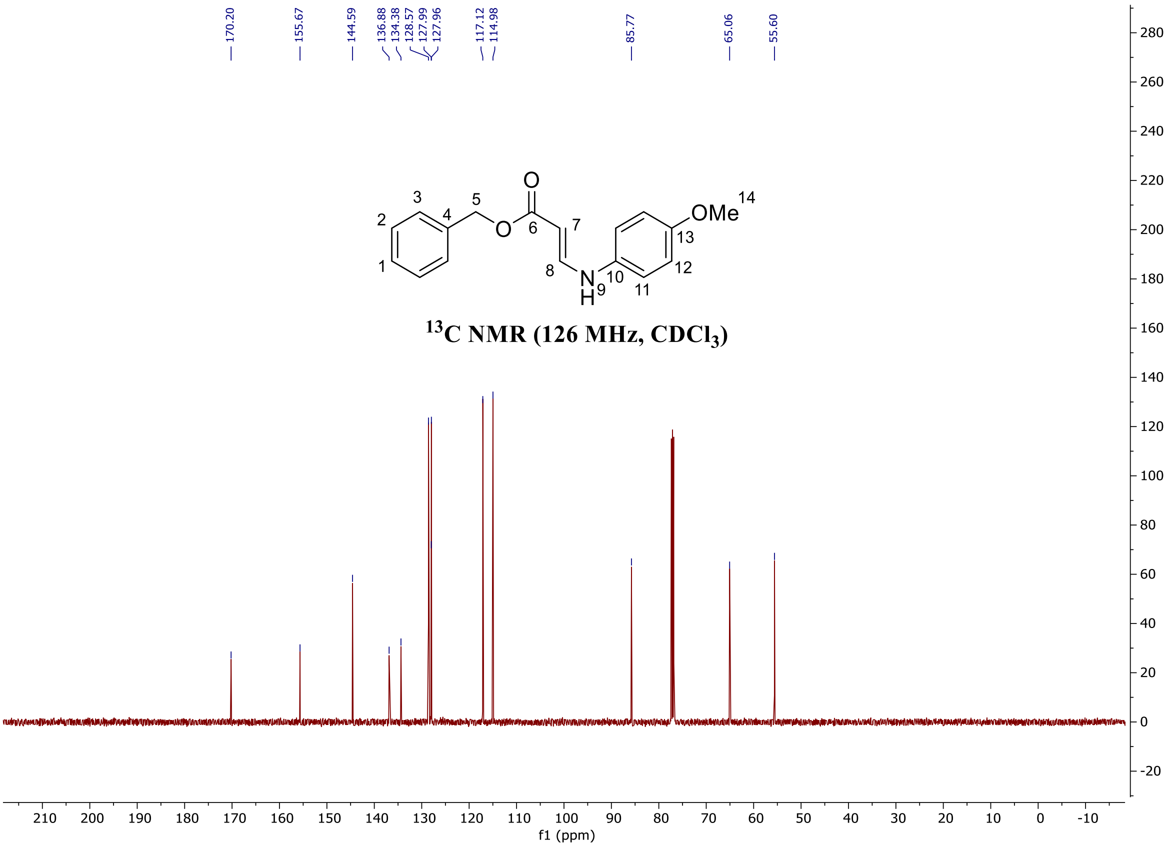

170.20
155.67
144.59
136.88
134.38
128.57
127.99
127.96
117.12
114.98
85.77
65.06
55.60
OMe
13C NMR (126 MHz, CDCl3)
f1 (ppm)

Benzyl (*E*)-3-((4-hydroxyphenyl)amino)acrylate (1i):

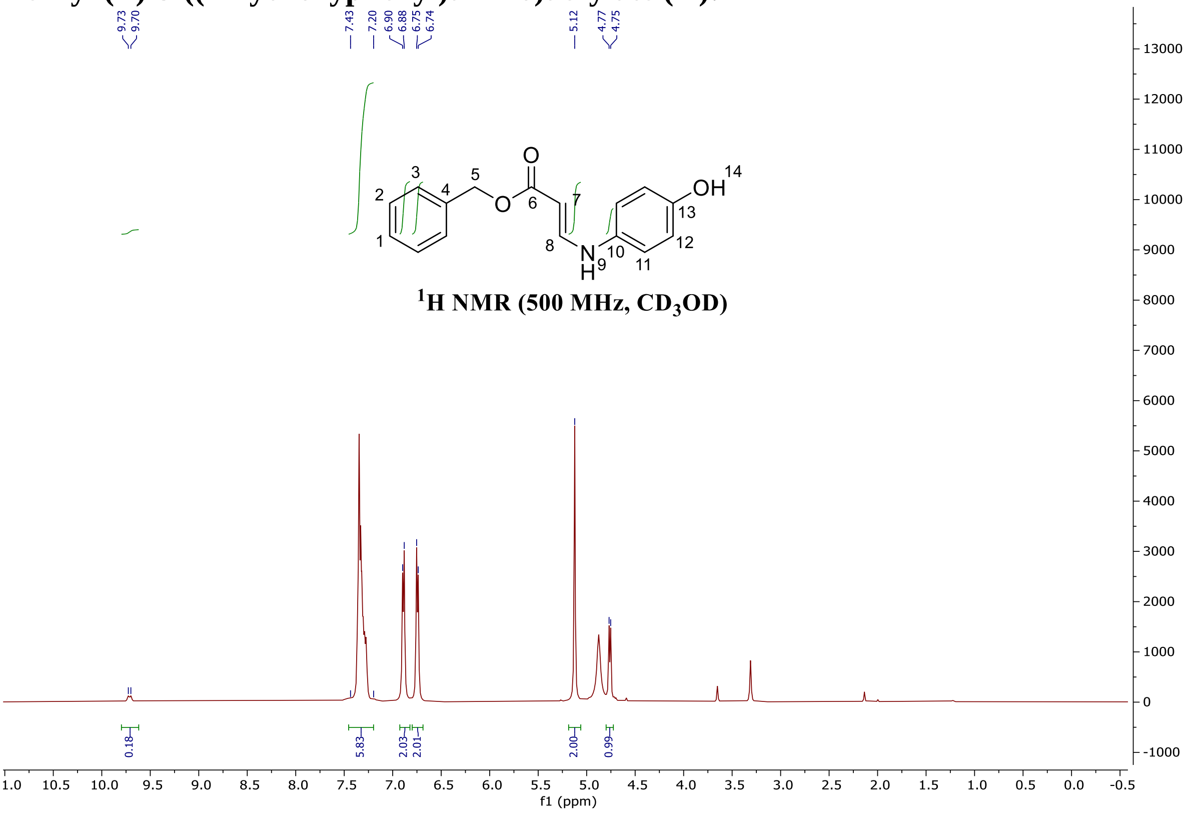

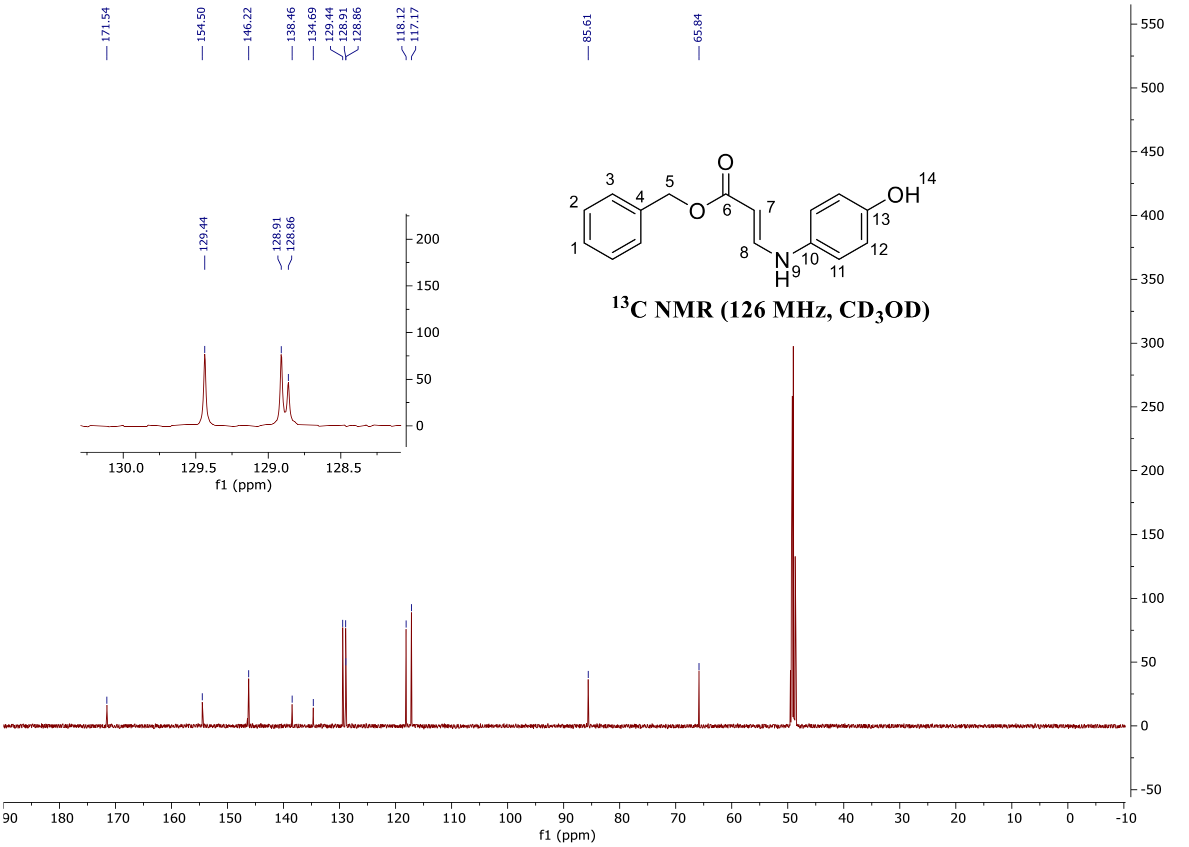

Benzyl (*E*)-3-((4-fluorophenyl)amino)acrylate (1j):

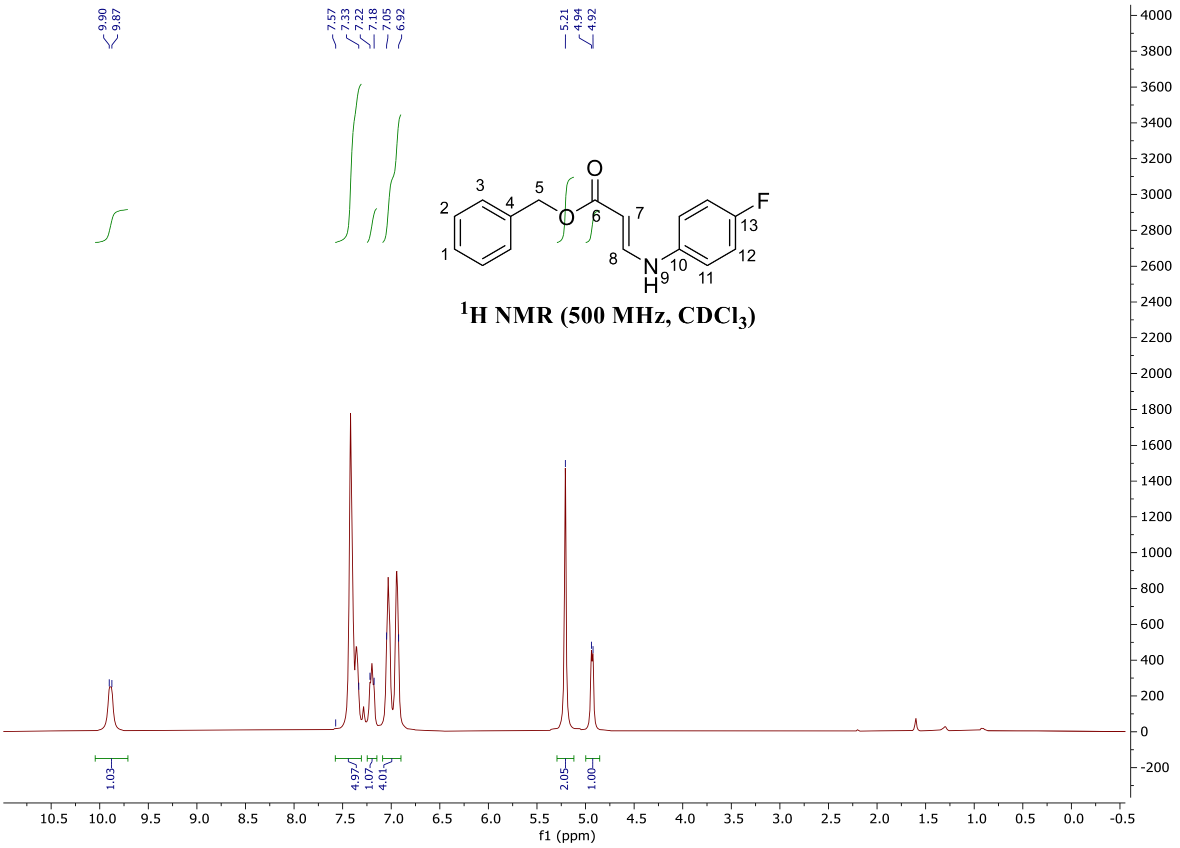

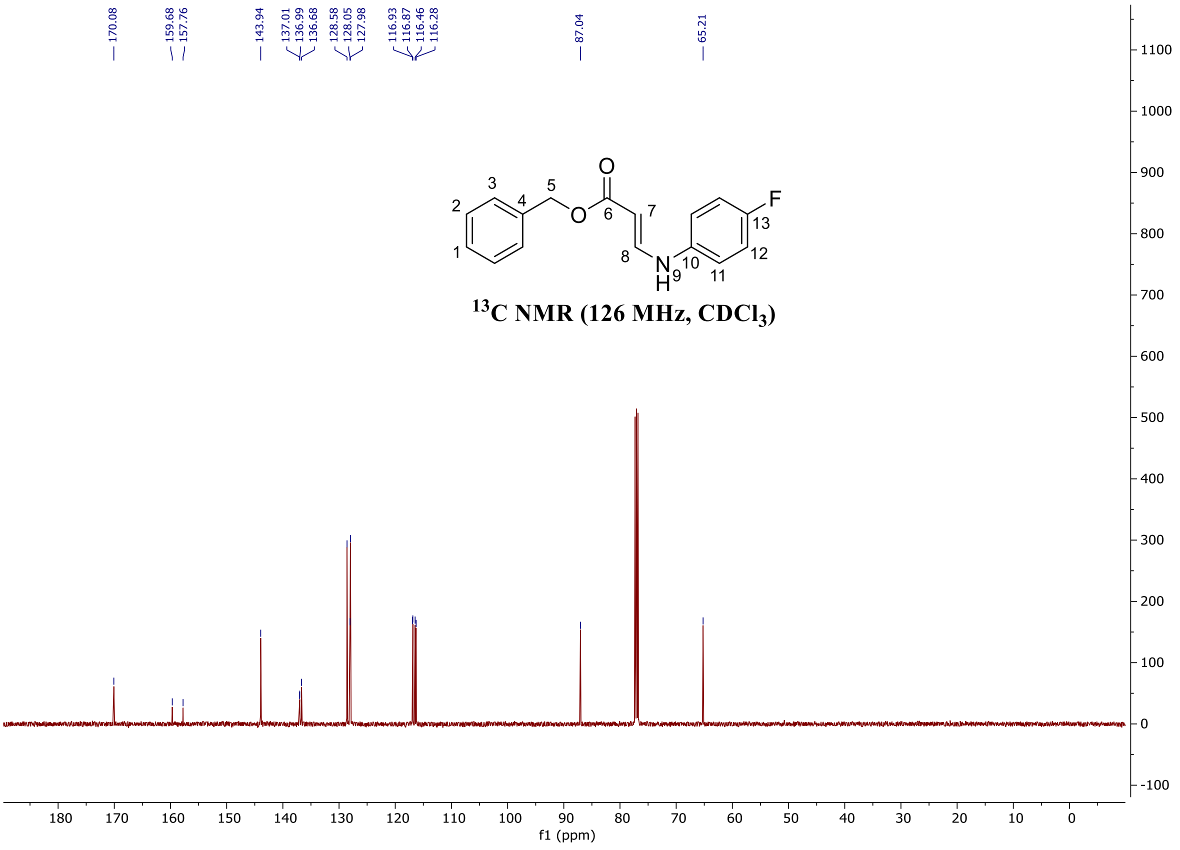

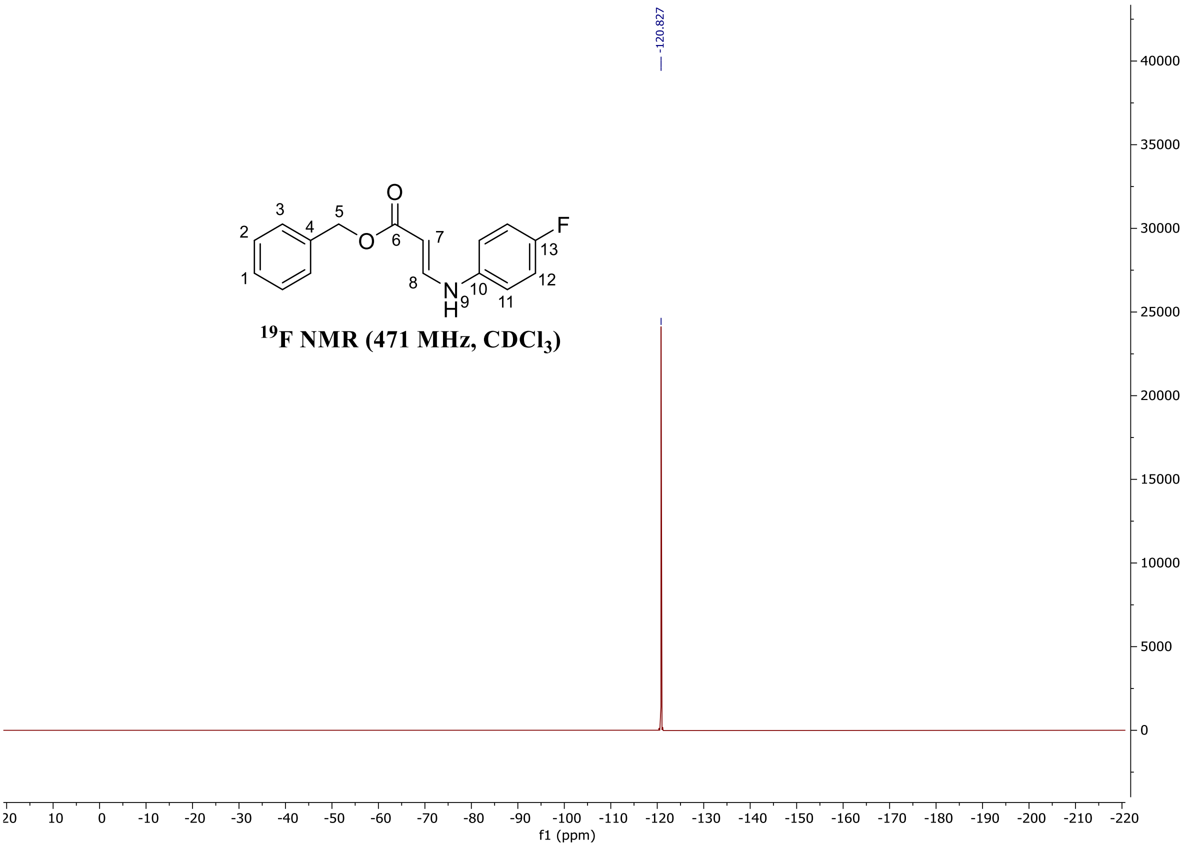

Benzyl (*E*)-3-((4-chlorophenyl)amino)acrylate (1k):

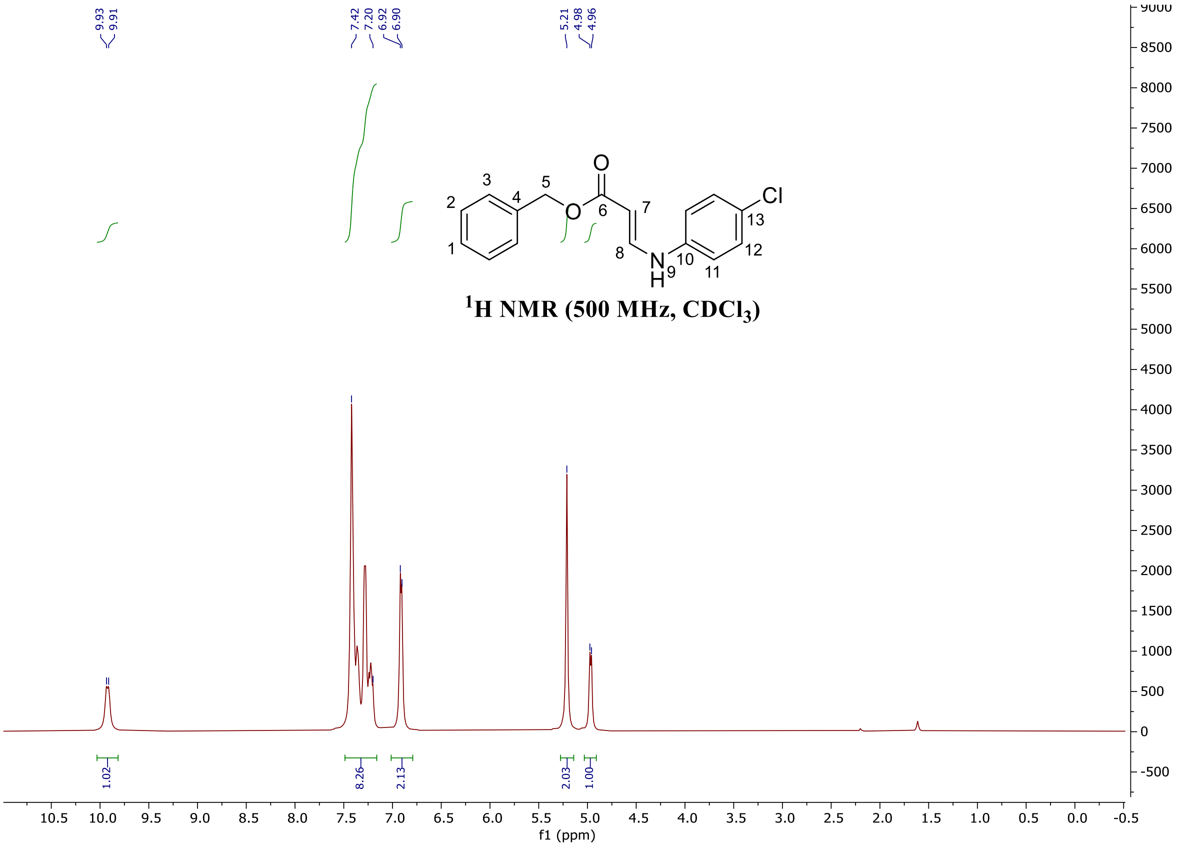

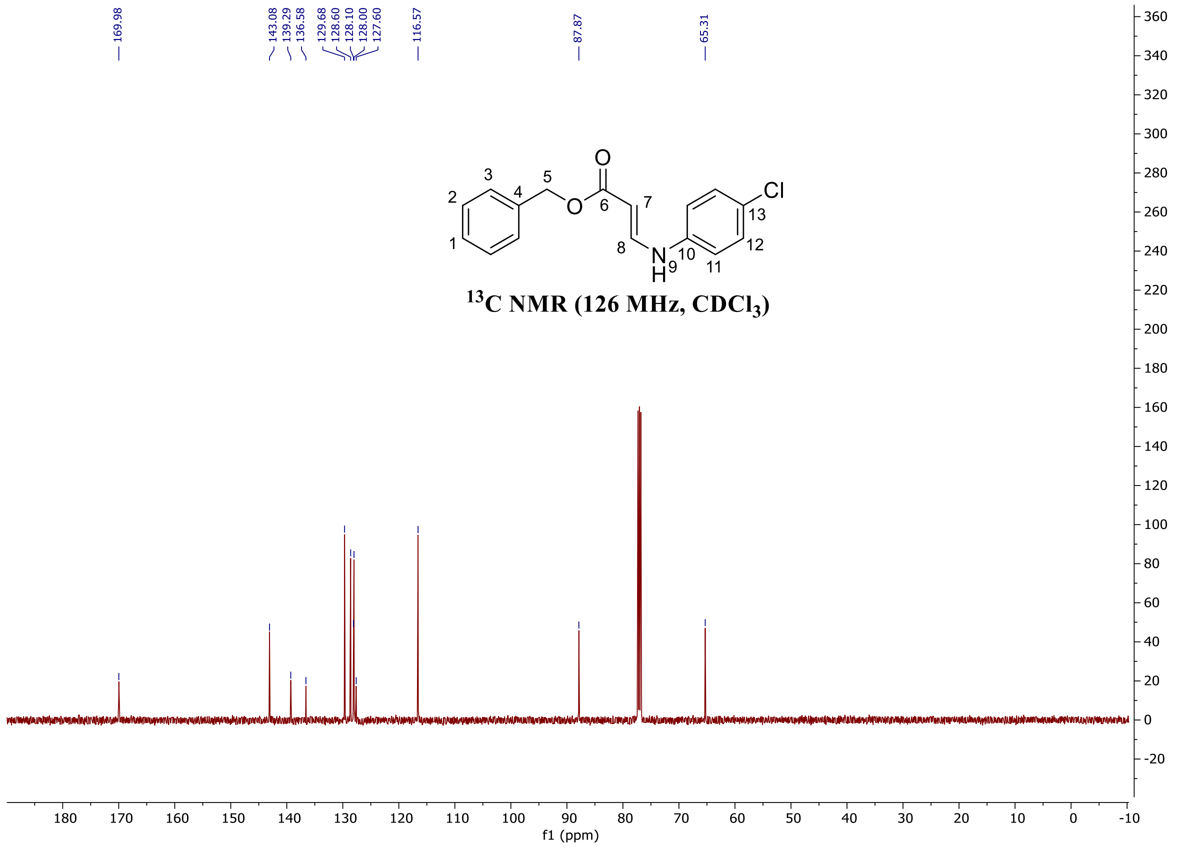

4-Bromobenzyl (*E*)-3-((4-methoxyphenyl)amino)acrylate (1l):

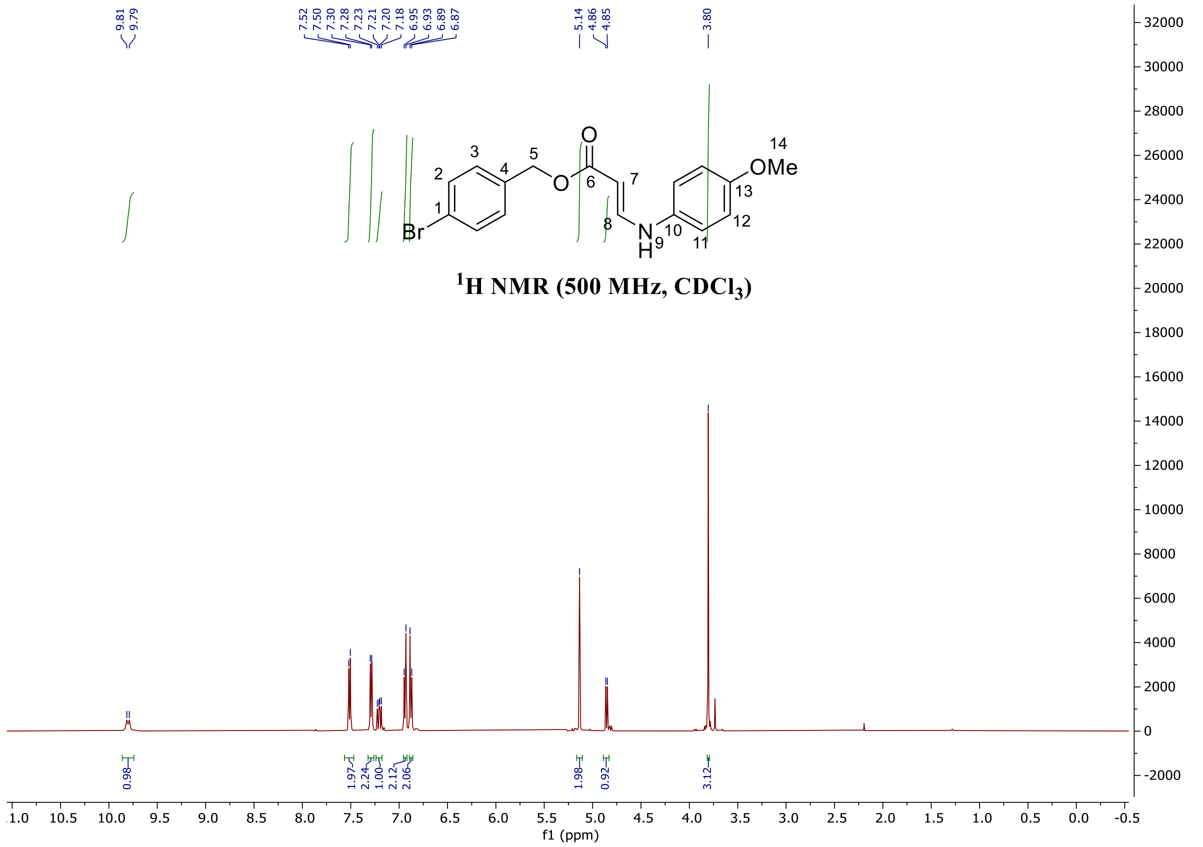

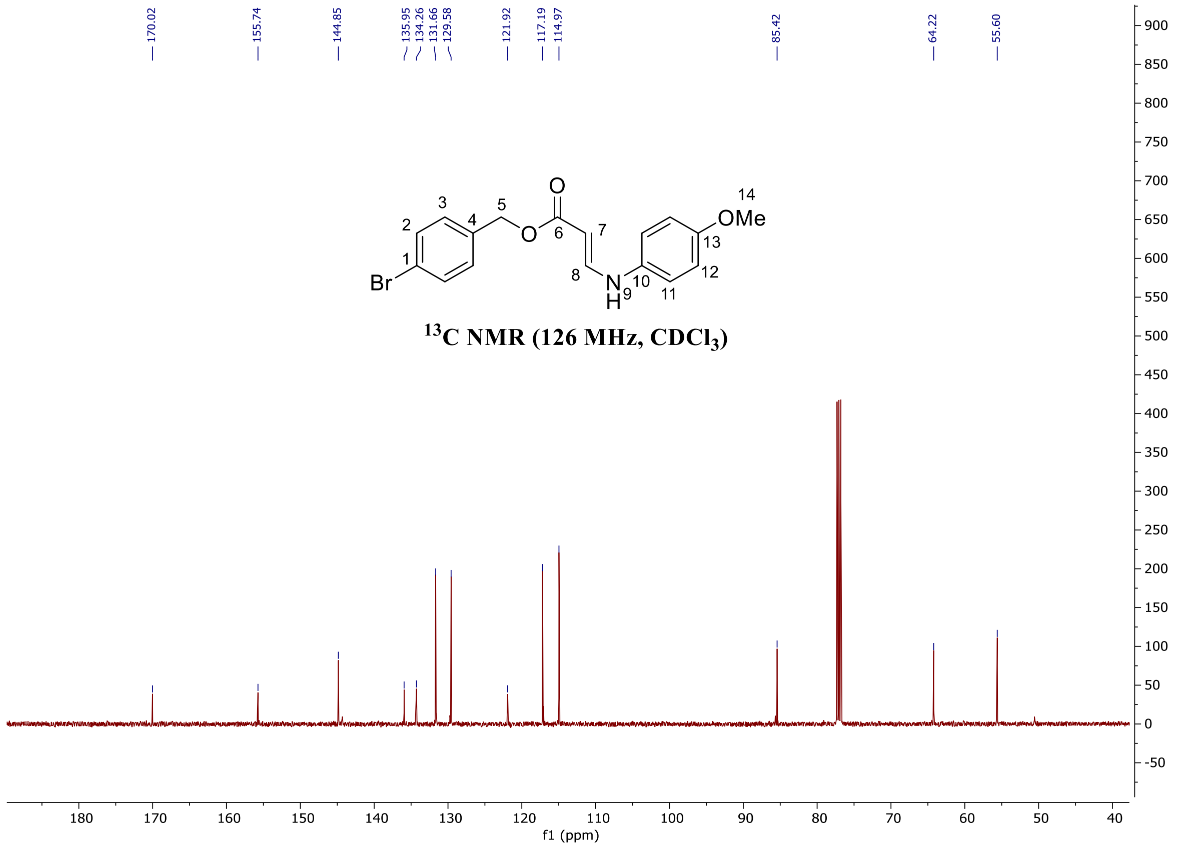

Methyl (*E*)-3-(methyl(phenyl)amino)acrylate (1m):

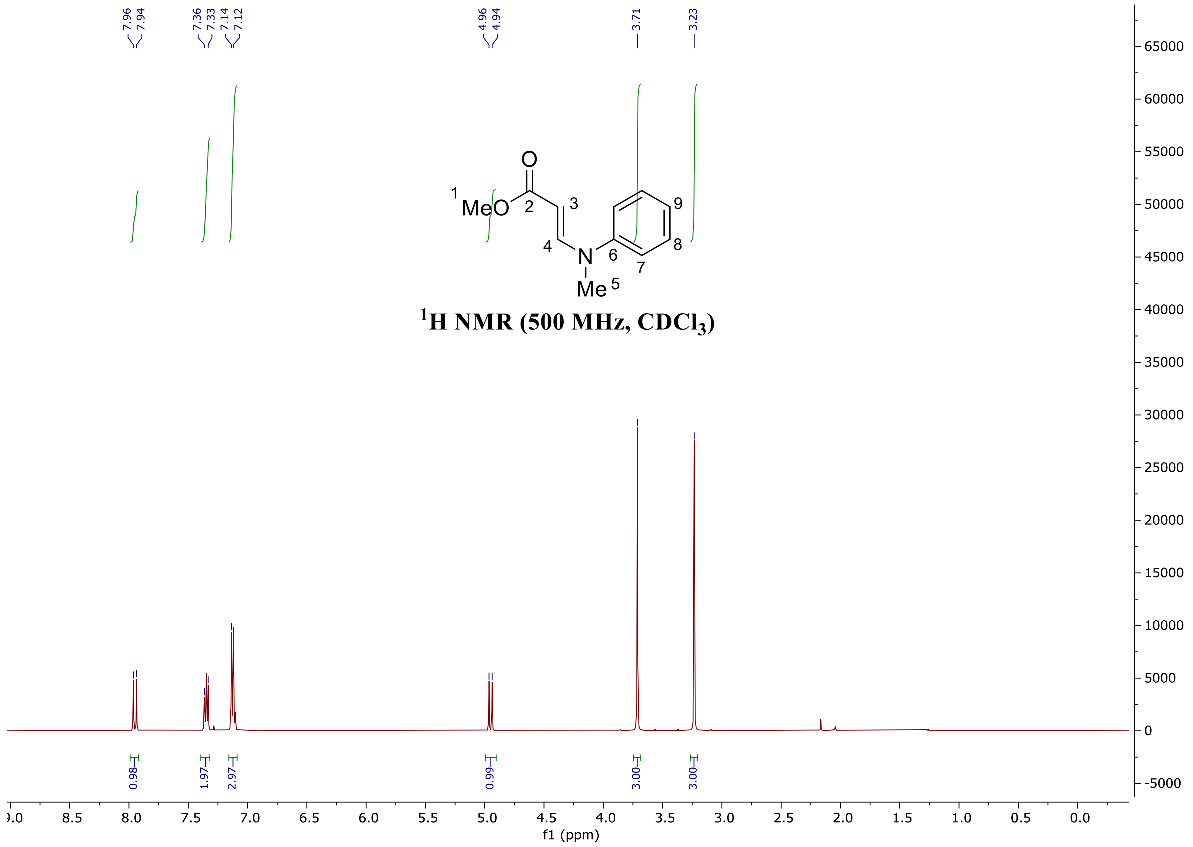

Methyl (*E*)-3-(benzylamino)acrylate (1n):

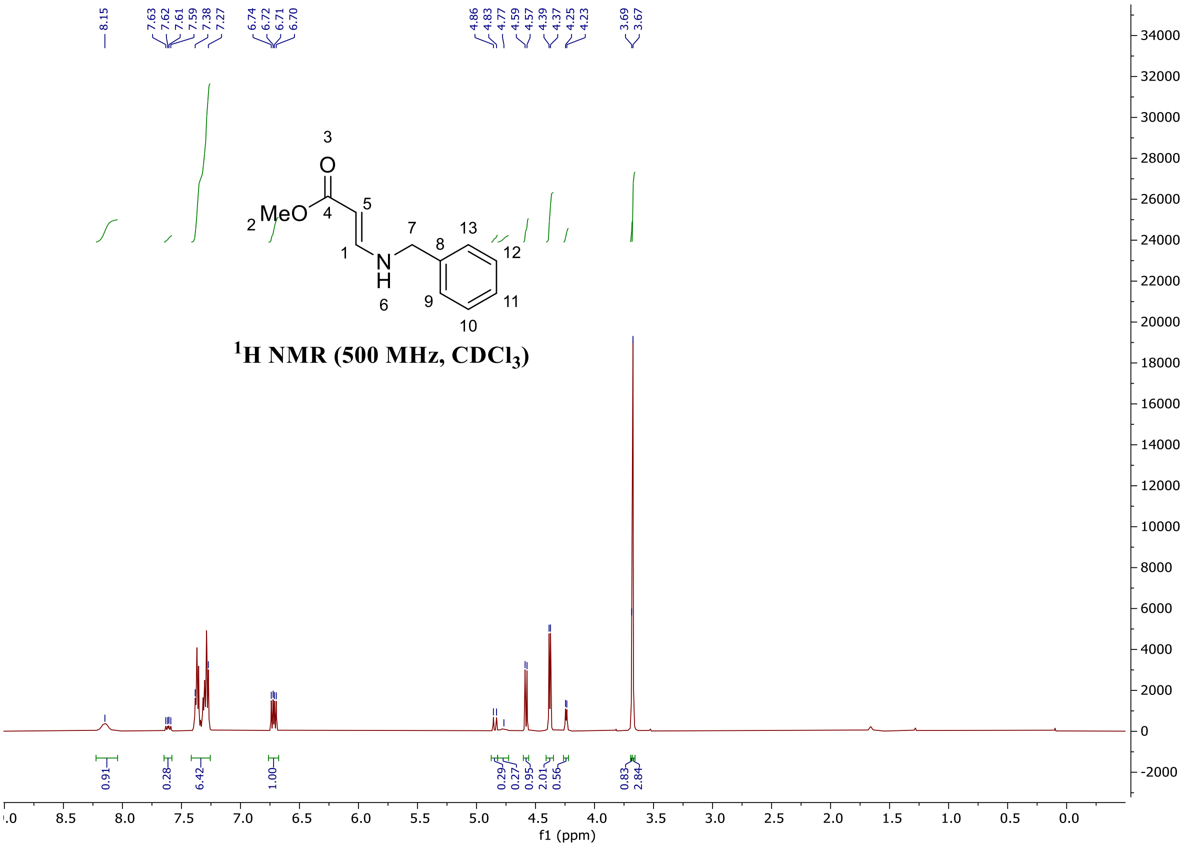

Methyl (*E*)-3-acetamidoacrylate (1o):

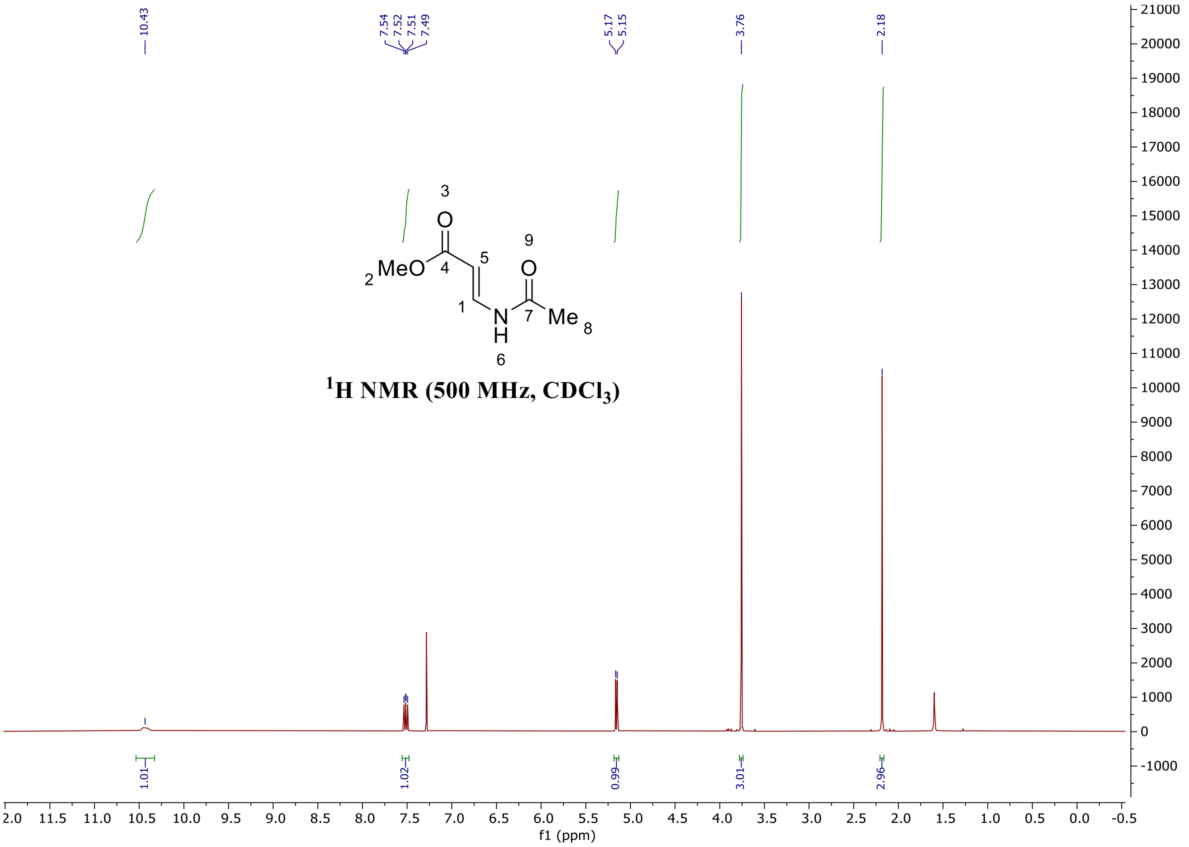

4-Vinylbenzyl (*S*)-2-(6-methoxynaphthalen-2-yl)propanoate (2x):

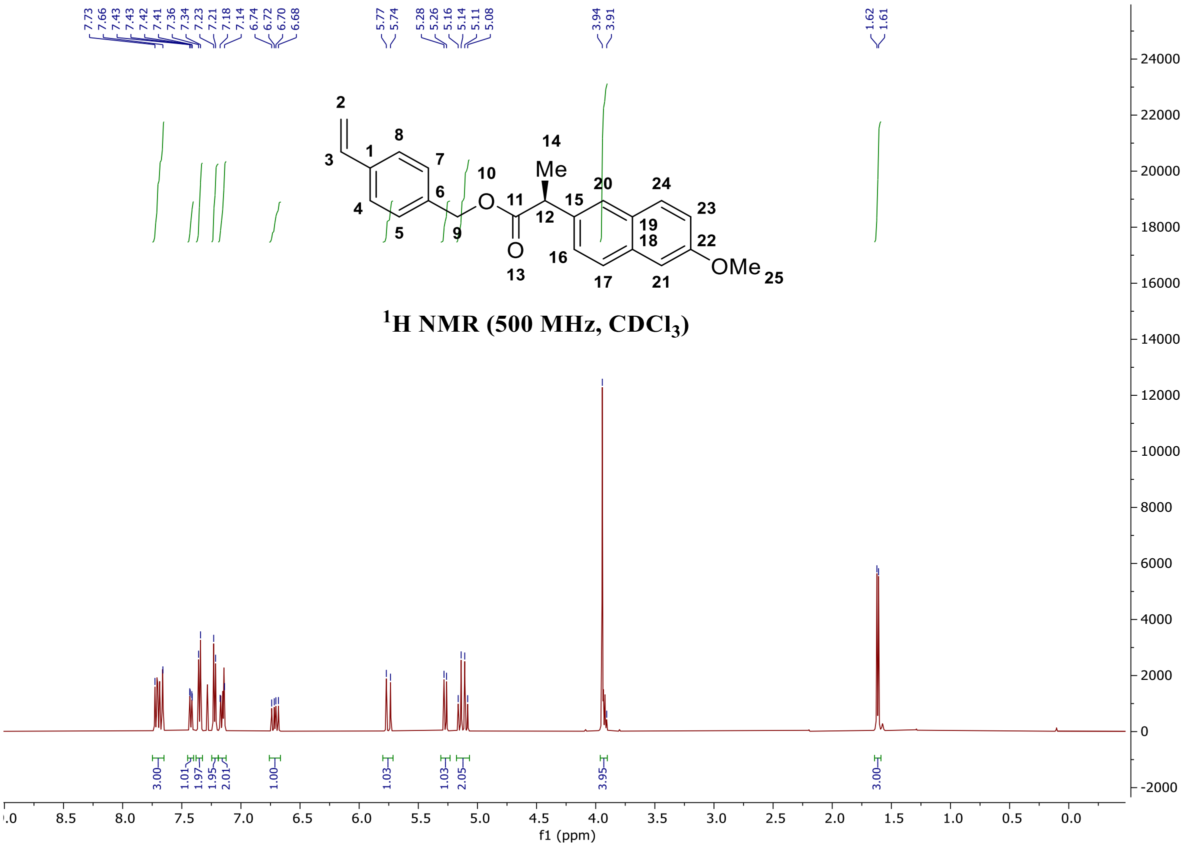

5-(3*S*,8*S*,9*S*,10*R*,13*R*,14*S*,17*R*)-10,13-Dimethyl-17-((*R*)-6-methylheptan-2-yl)-2,3,4,7,8,9,10,11,12,13,14,15,16,17-tetradecahydro-1*H*-cyclopenta[*a*]phenanthren-3-yl pent-4-enoate (2y):

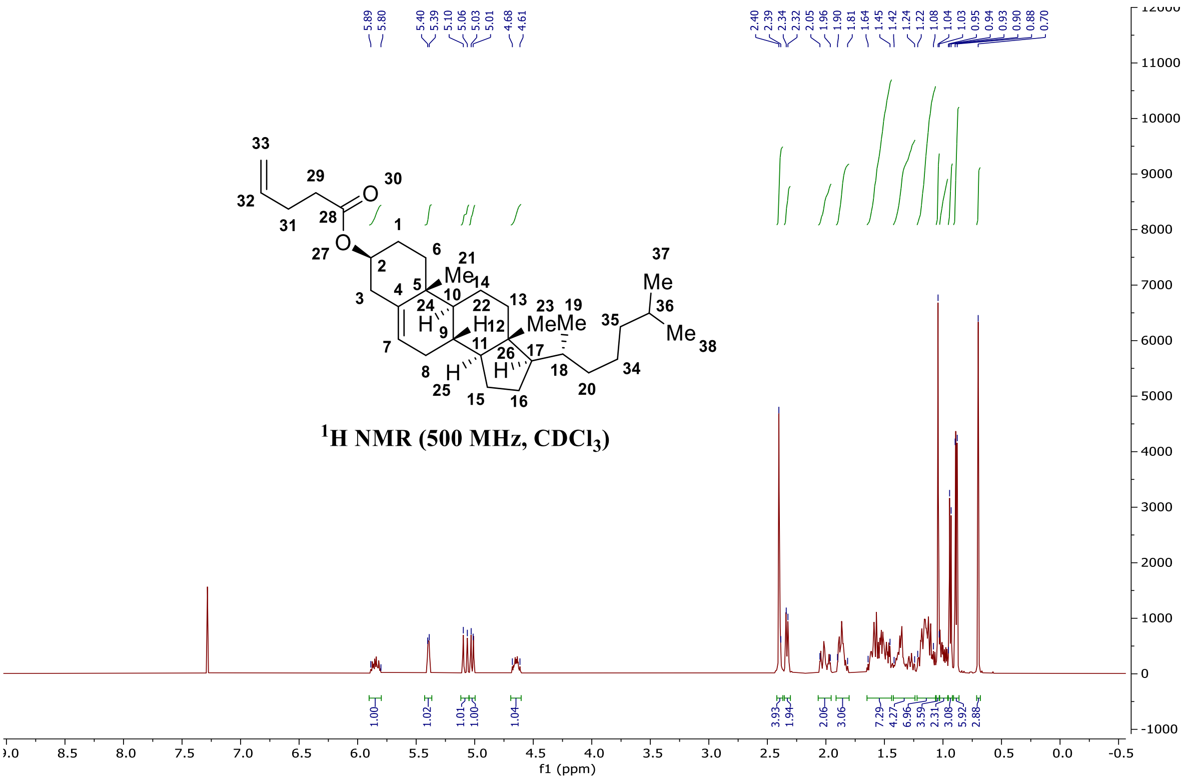

2-(8*R*,9*S*,13*S*,14*S*)-13-Methyl-3-vinyl-6,7,8,9,11,12,13,14,15,16-decahydro-17*H*-cyclopenta[*a*]phenanthren-17-one (2z):

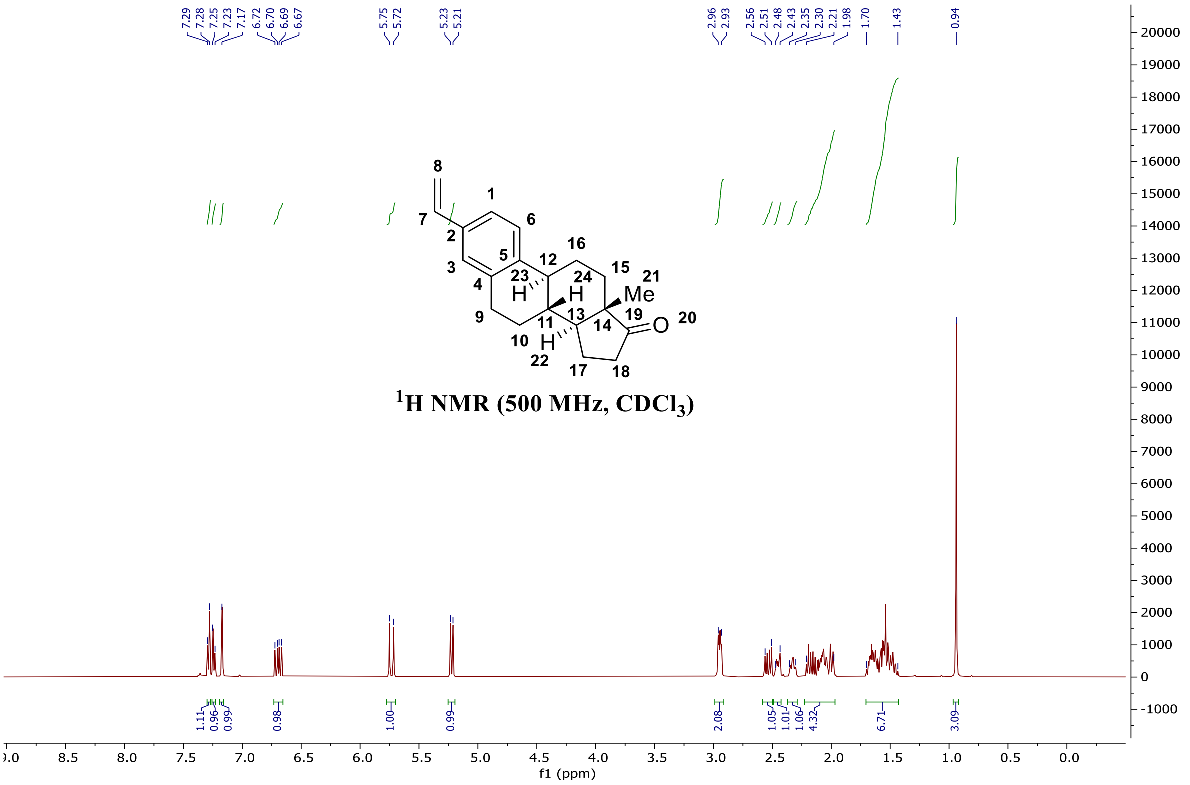

Benzyl (*R*,*Z*)-3-phenyl-2-((phenylamino)methylene)butanoate (3aa):

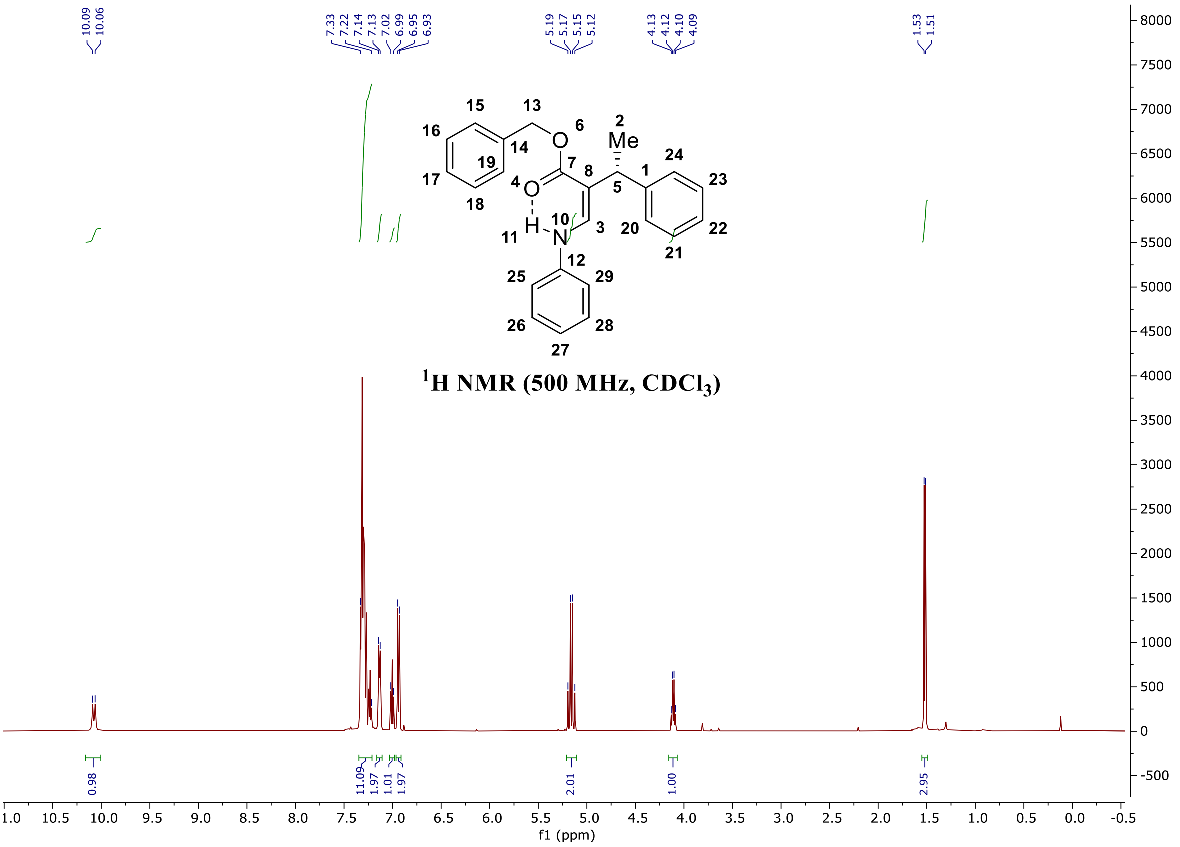

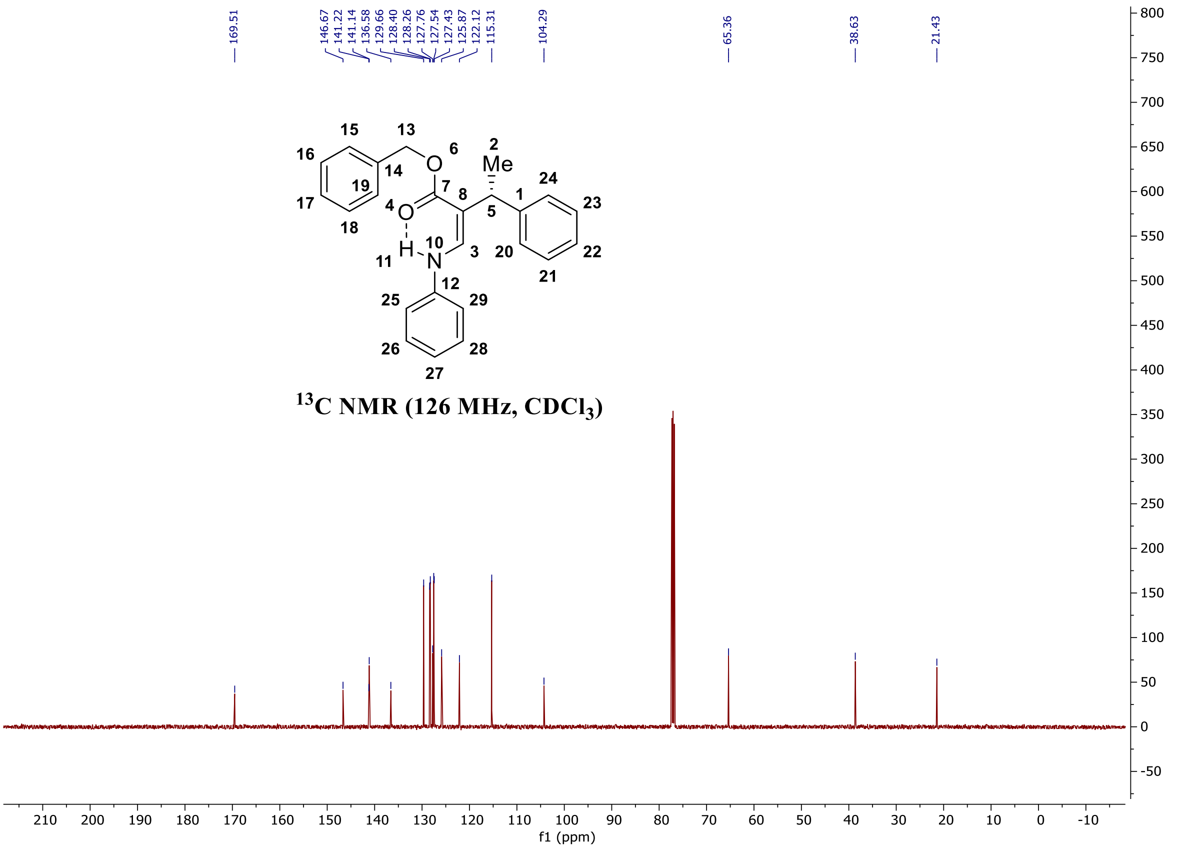

Methyl (*R*,*Z*)-3-phenyl-2-((phenylamino)methylene)butanoate (3ba):

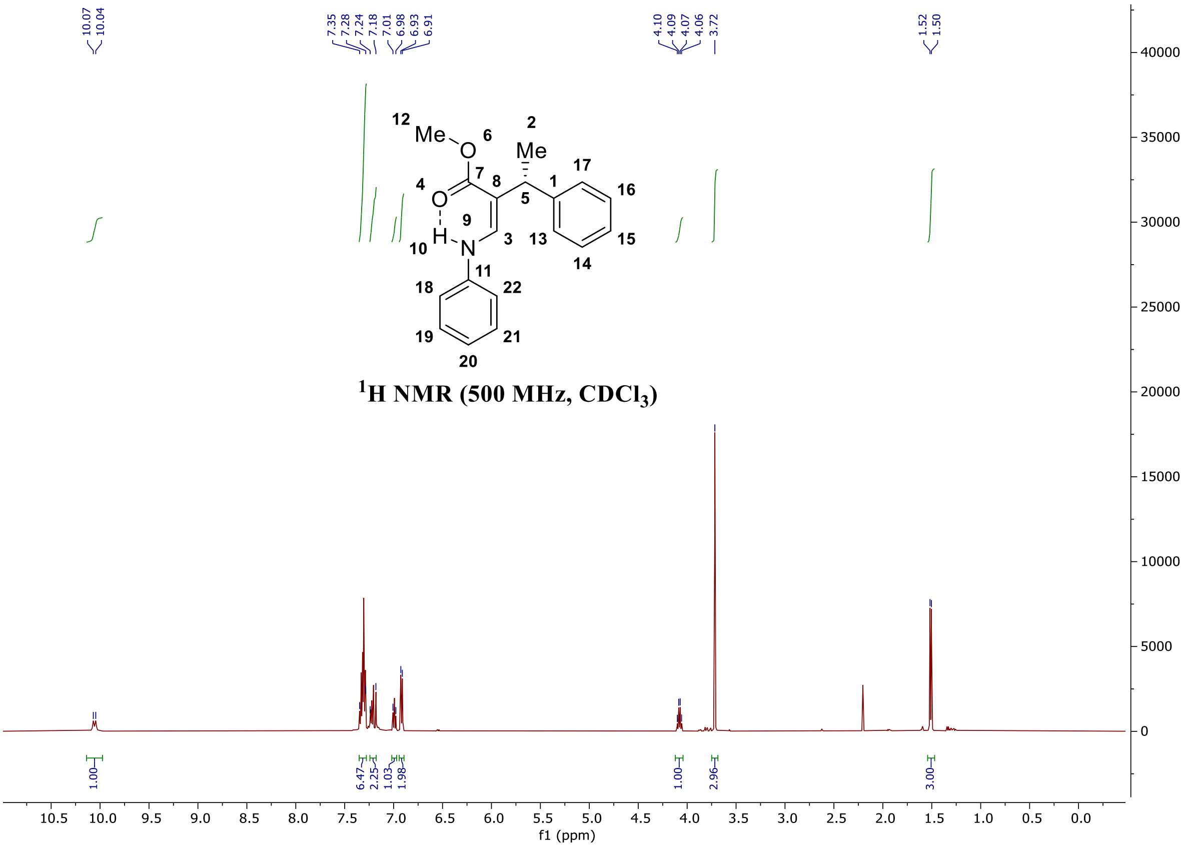

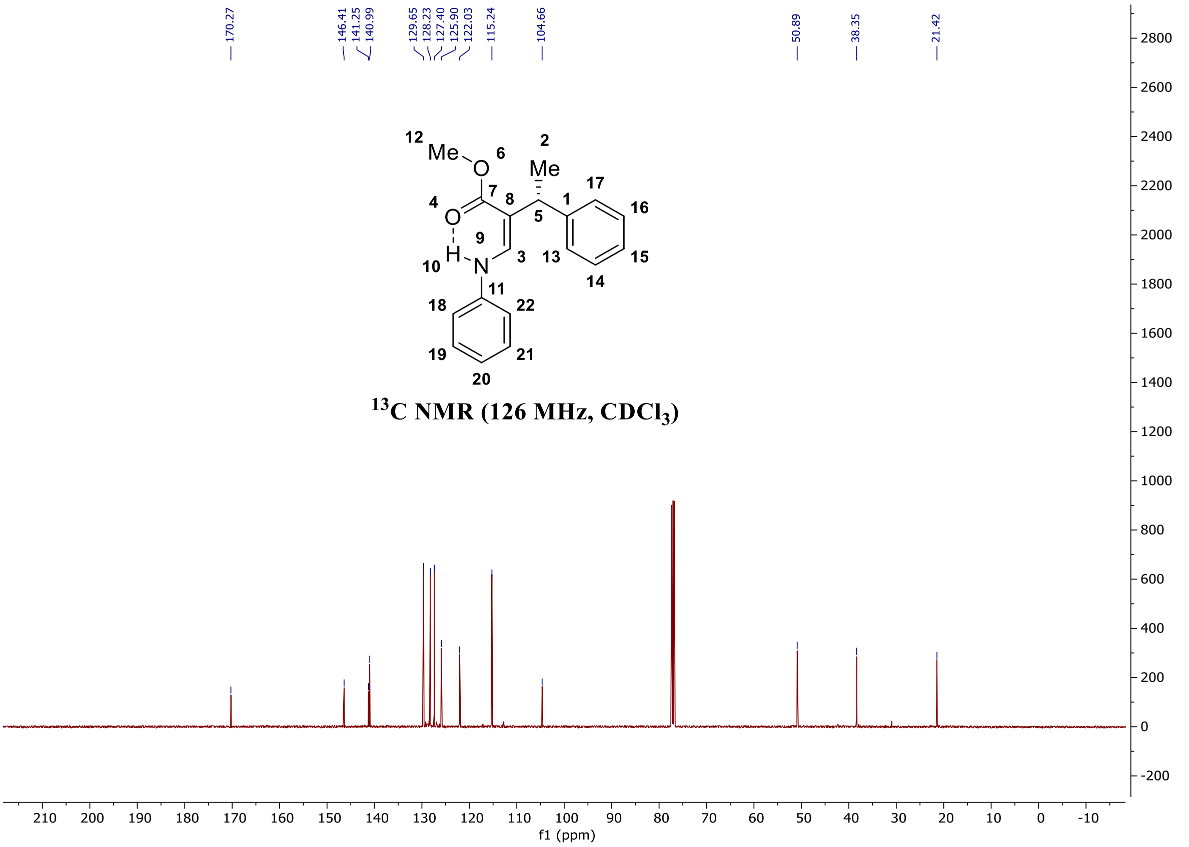

Ethyl (*R*,*Z*)-3-phenyl-2-((phenylamino)methylene)butanoate (3ca):

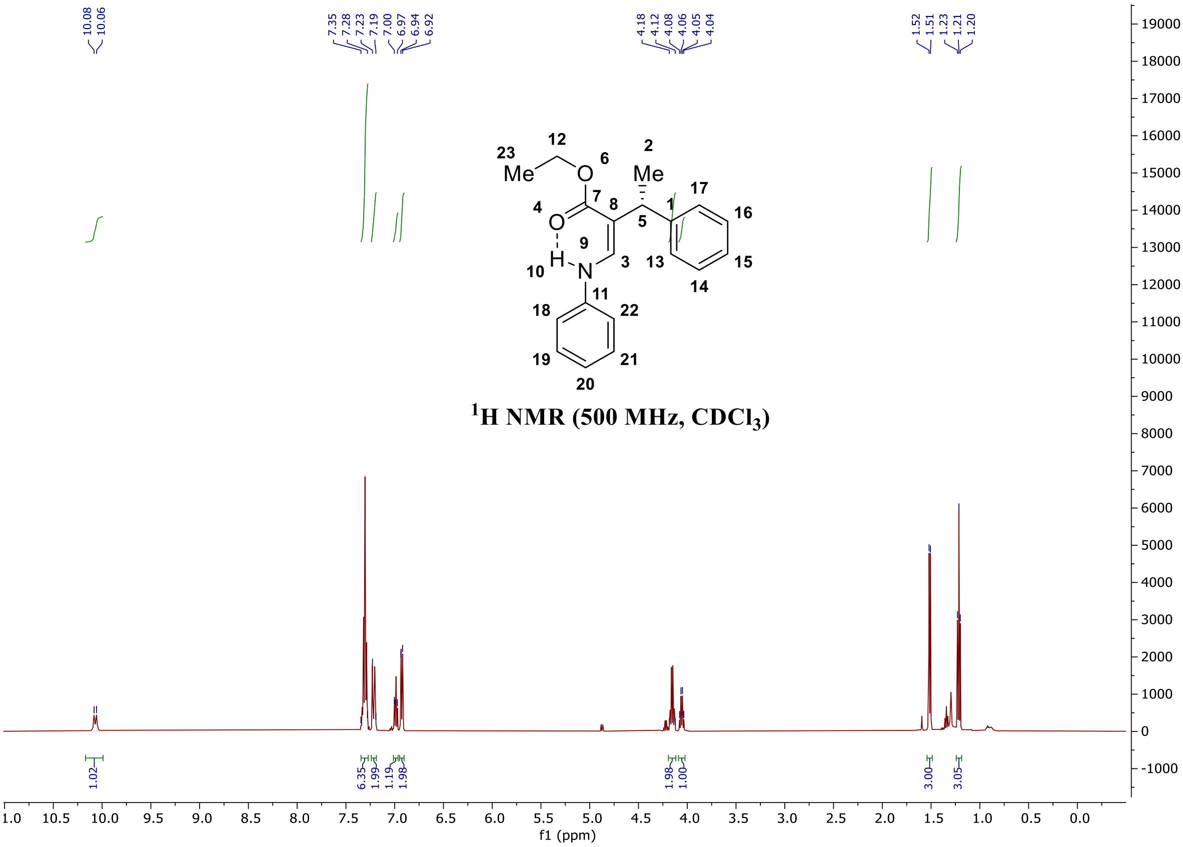

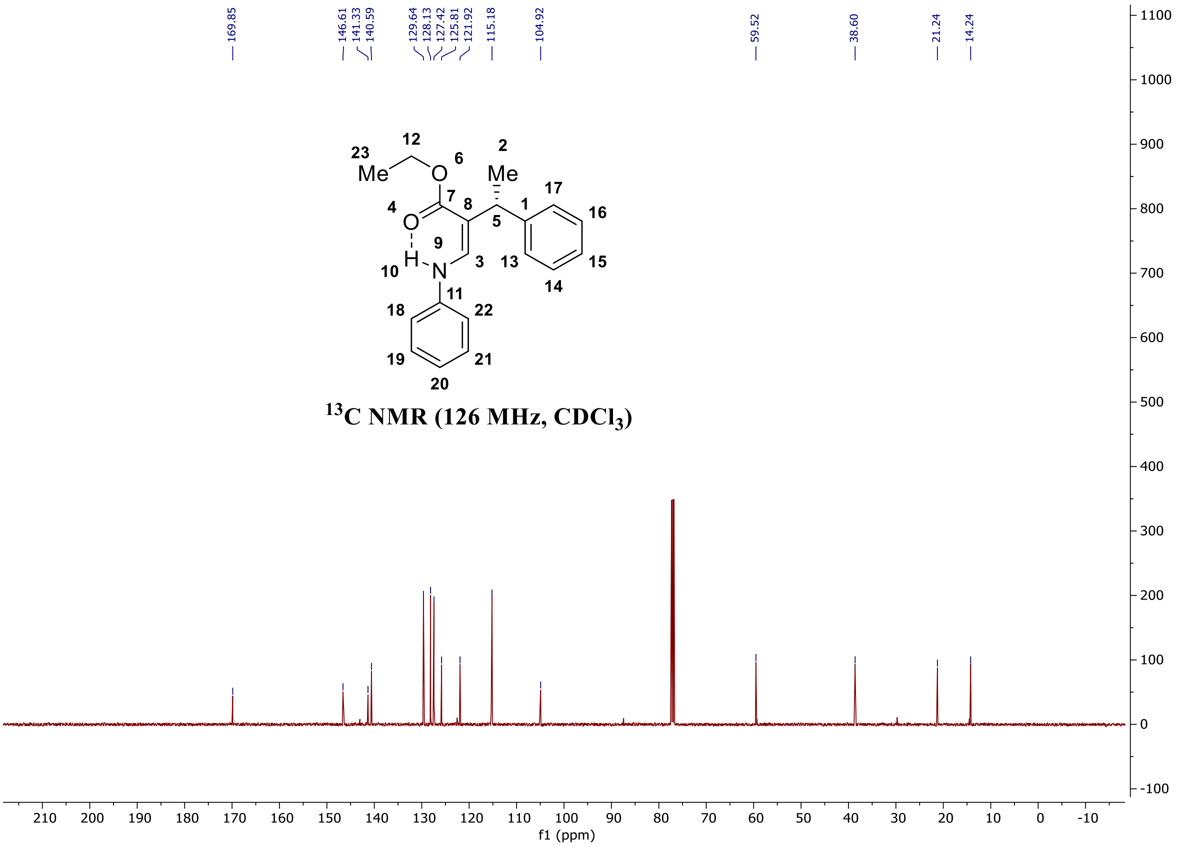

Isopropyl (*R*,*Z*)-3-phenyl-2-((phenylamino)methylene)butanoate (3da):

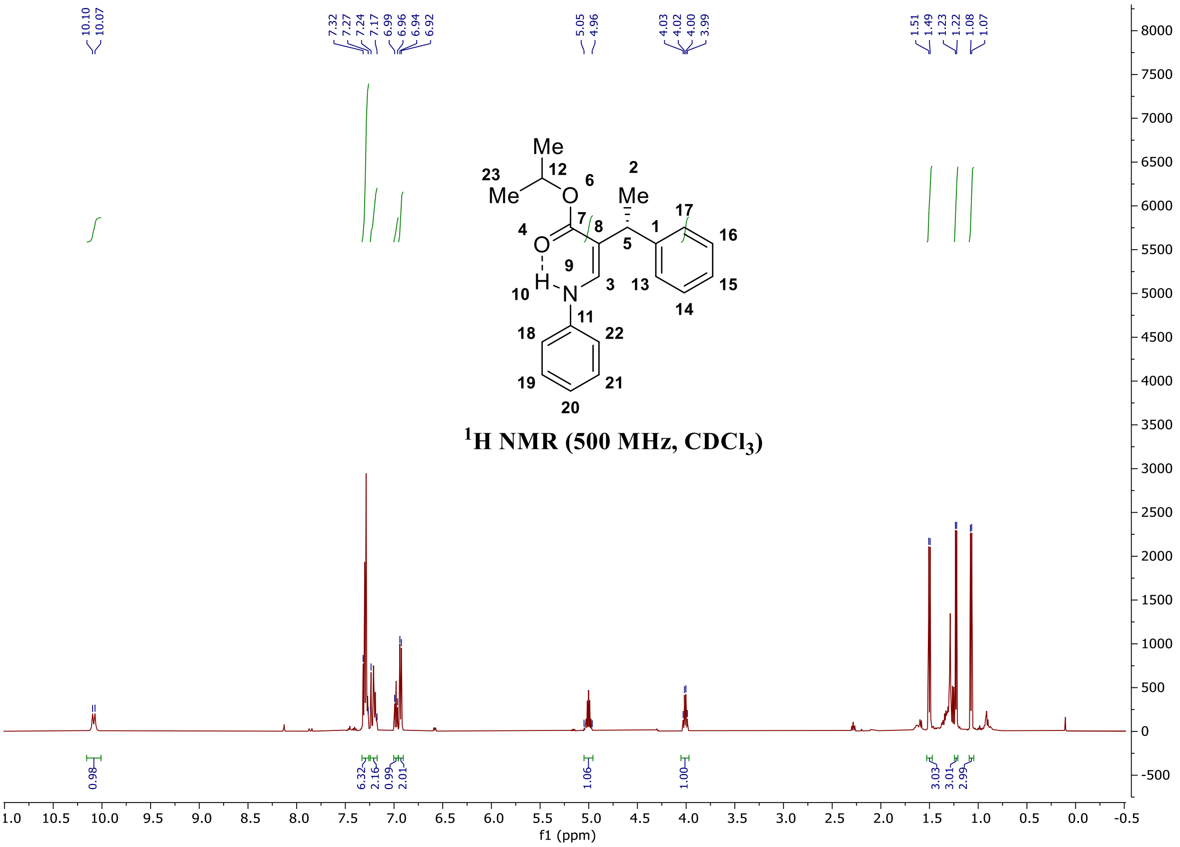

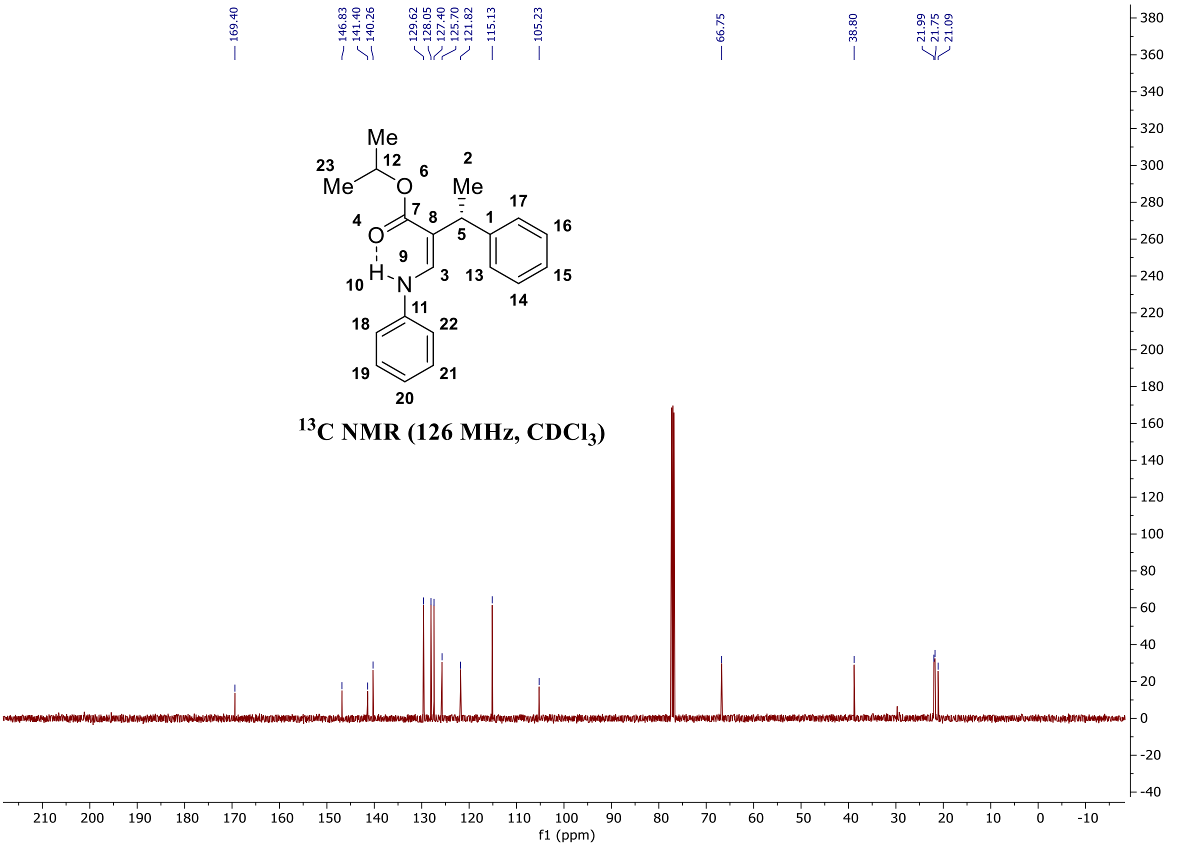

Benzyl (R,Z)-3-phenyl-2-((p-tolylamino)methylene)butanoate (3ga):

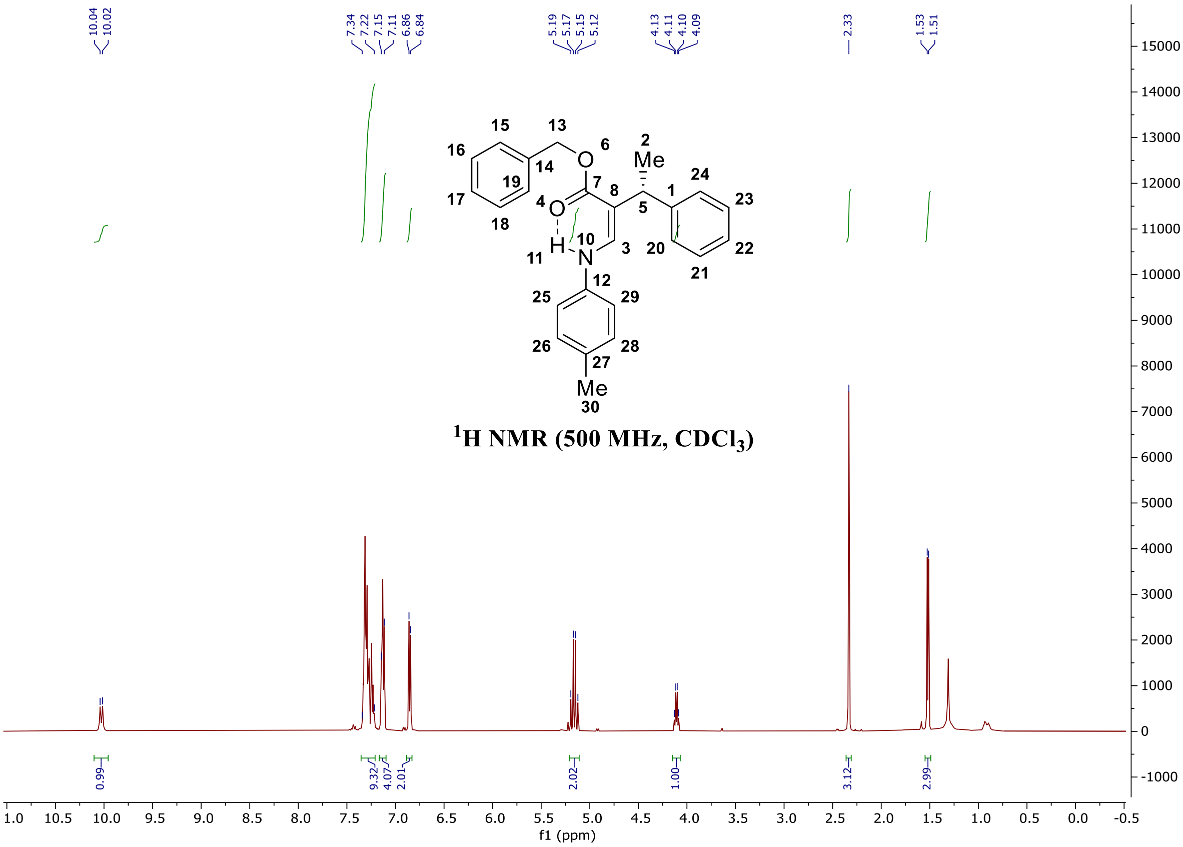

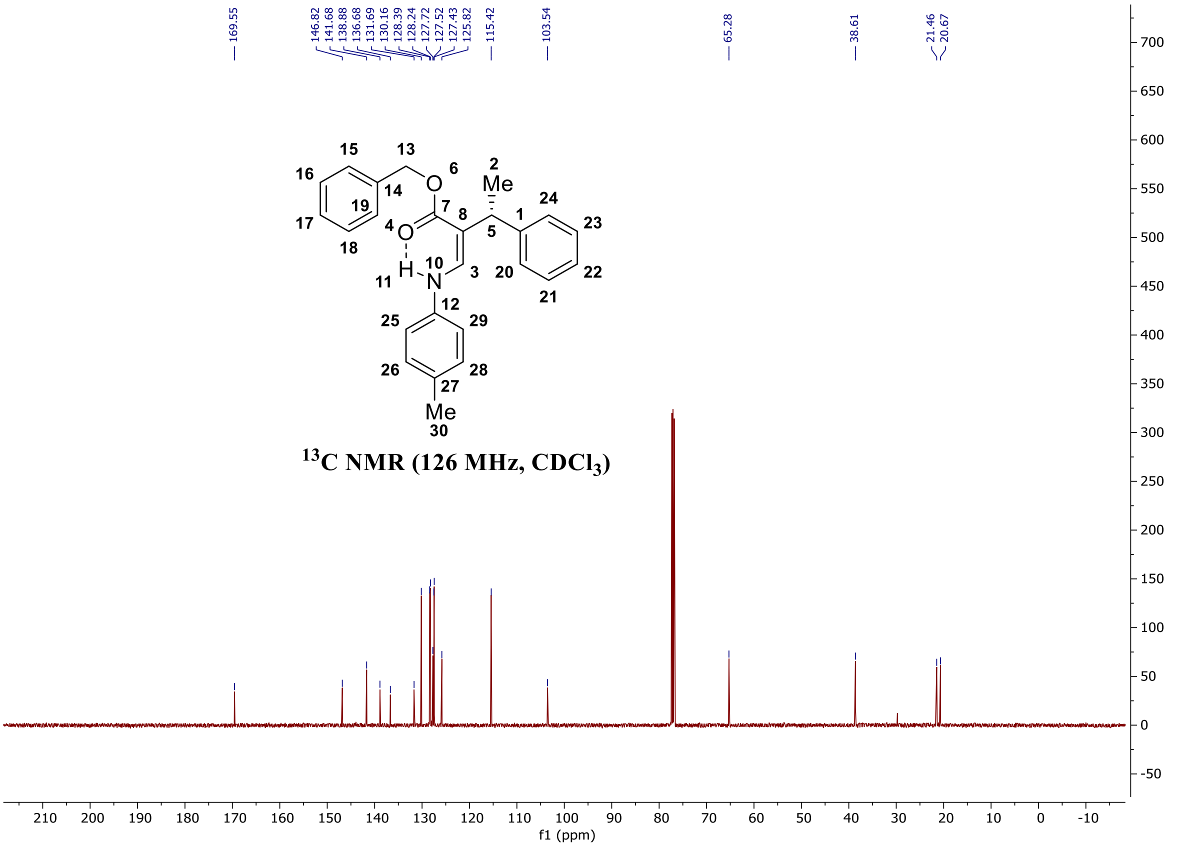

Benzyl (*R*,*Z*)-2-(((4-methoxyphenyl)amino)methylene)-3-phenylbutanoate (3ha):

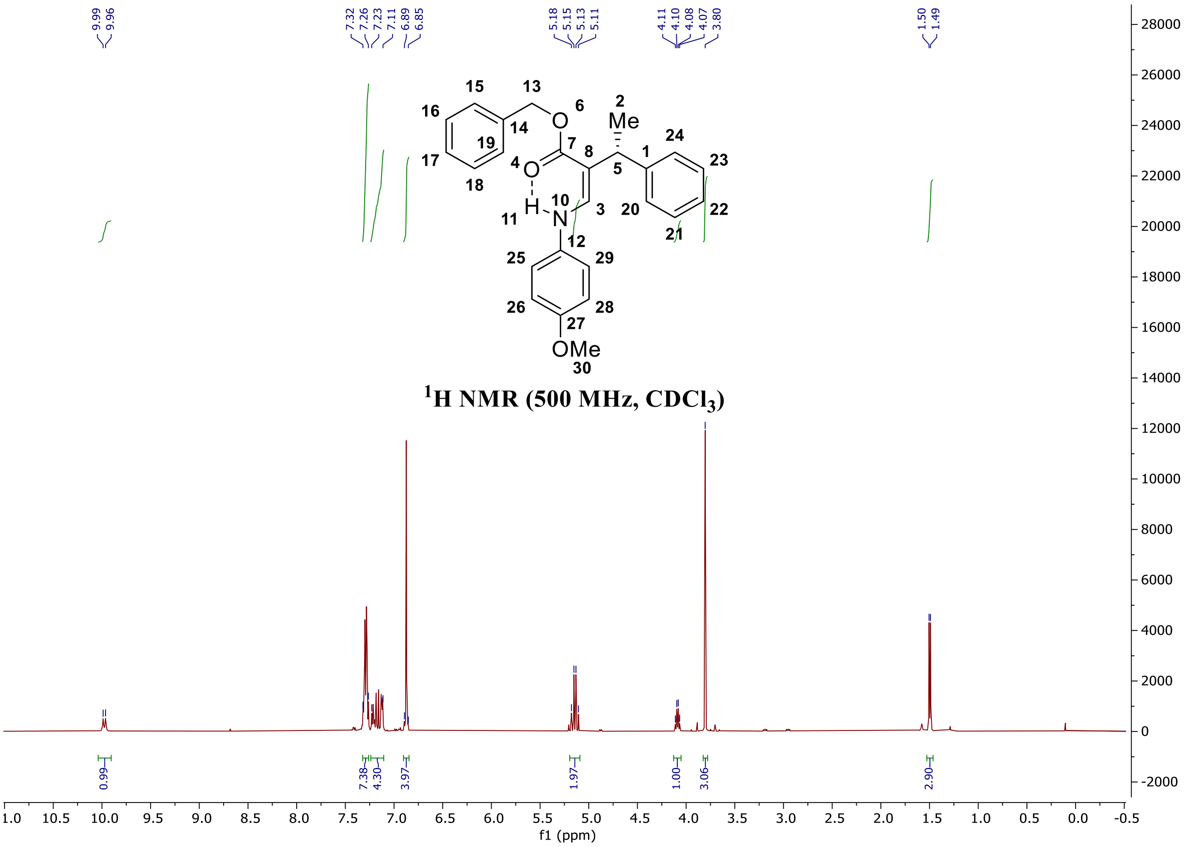

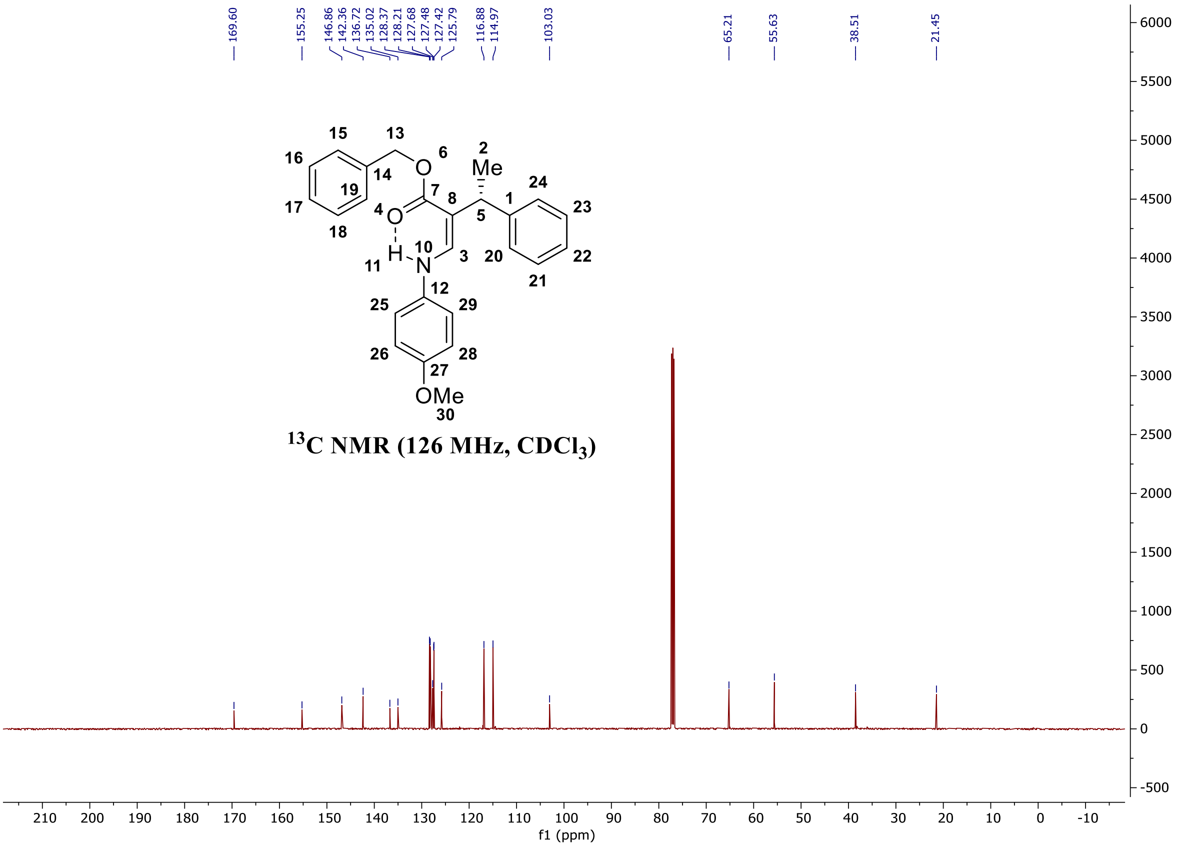

Benzyl (*R*,*Z*)-2-(((4-hydroxyphenyl)amino)methylene)-3-phenylbutanoate (3ia)

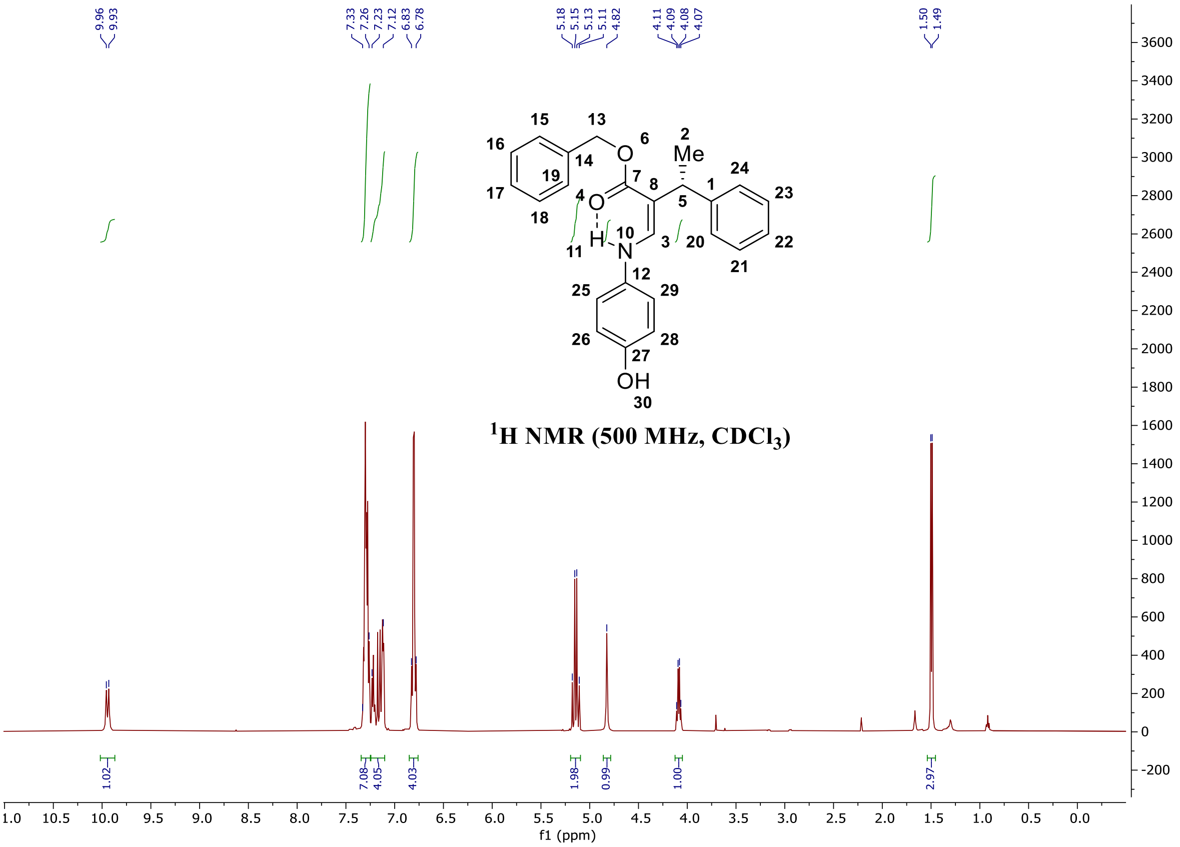

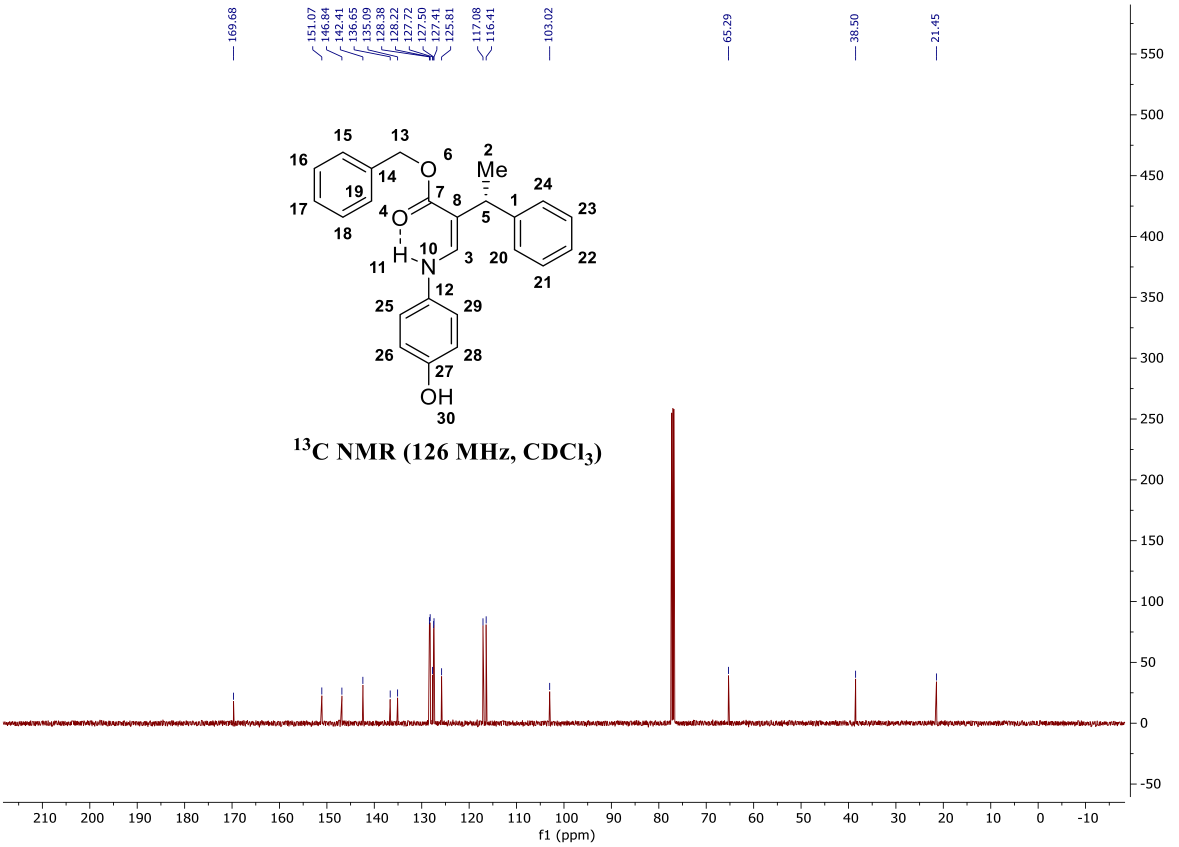

Benzyl (*R*,*Z*)-2-(((4-fluorophenyl)amino)methylene)-3-phenylbutanoate (3ja):

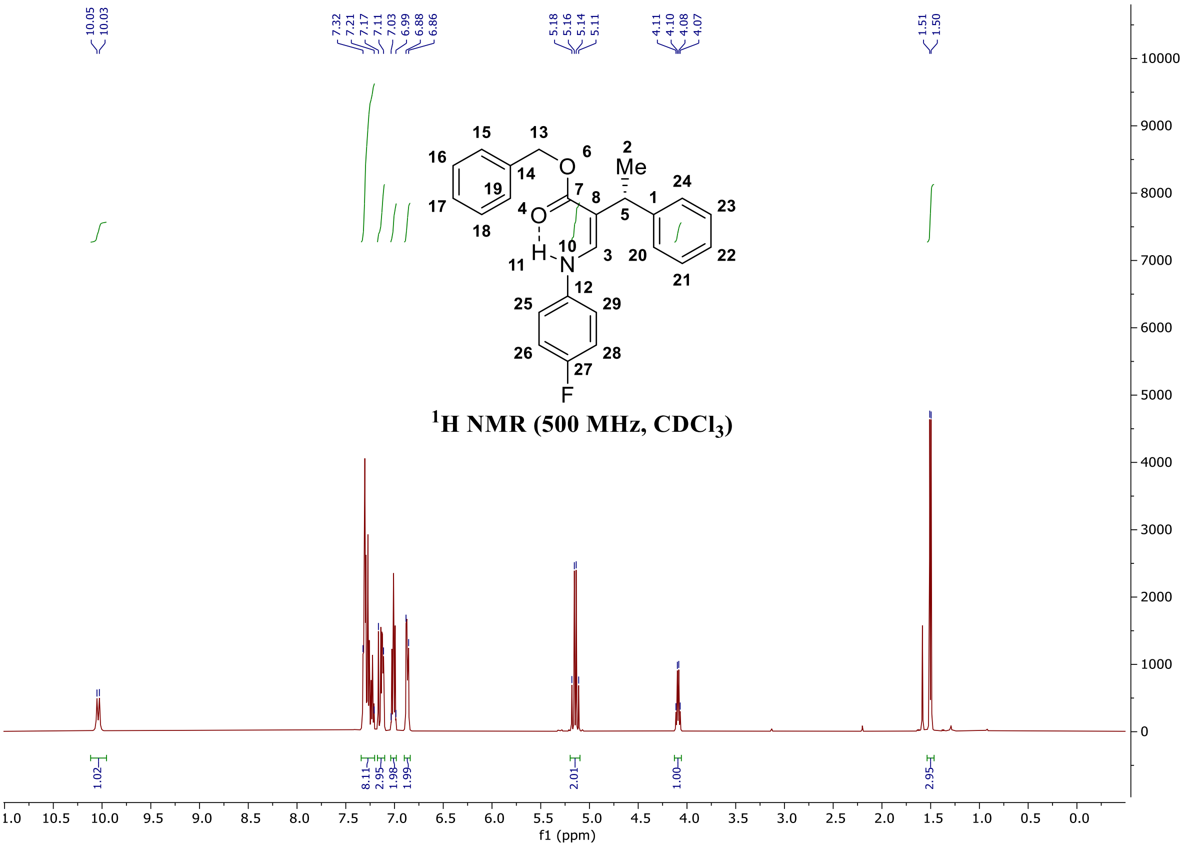

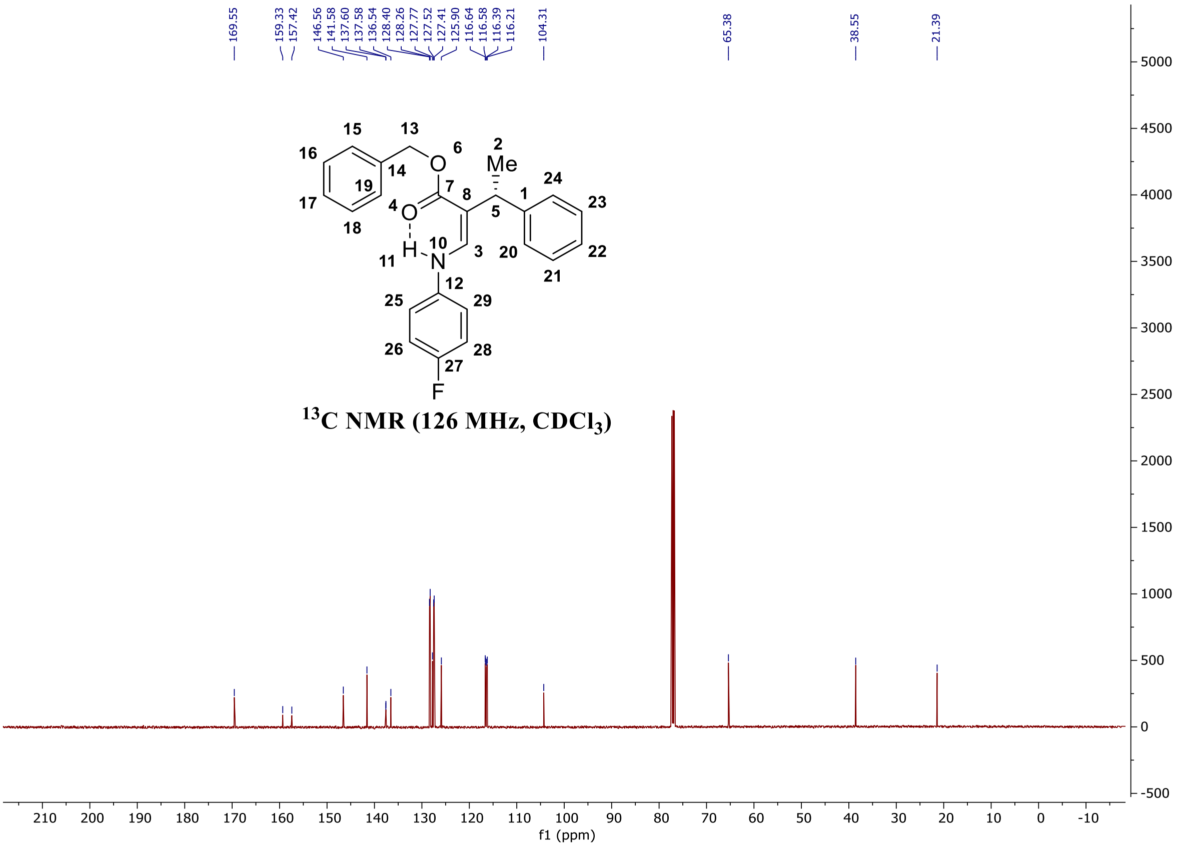

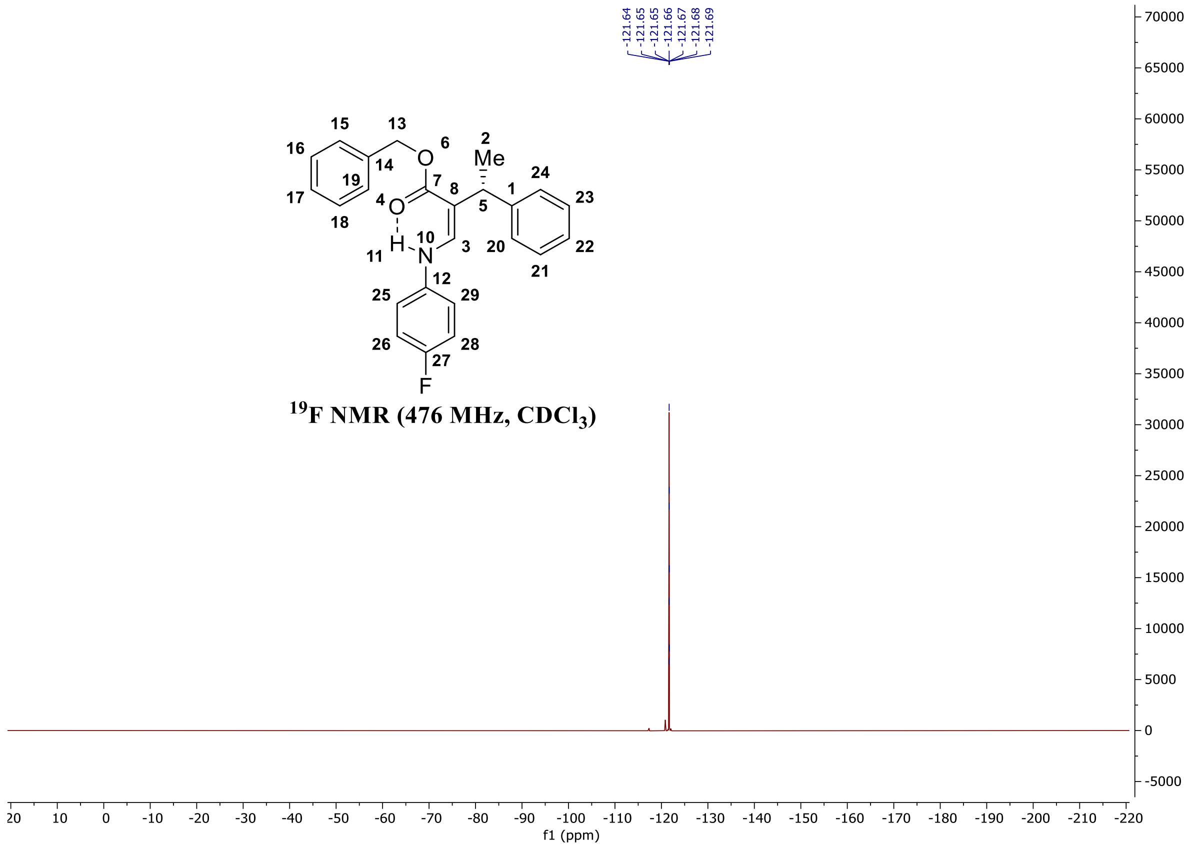

Benzyl (*R*,*Z*)-2-(((4-chlorophenyl)amino)methylene)-3-phenylbutanoate (3ka):

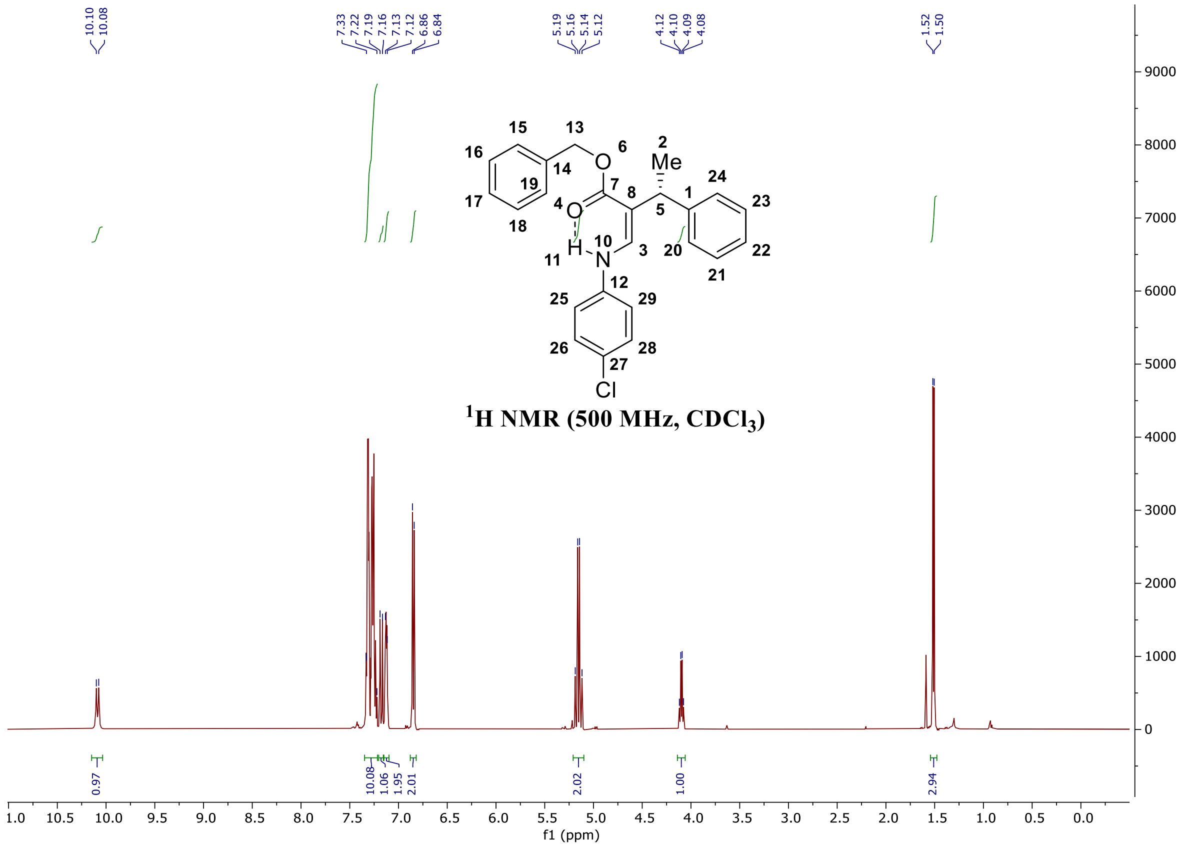

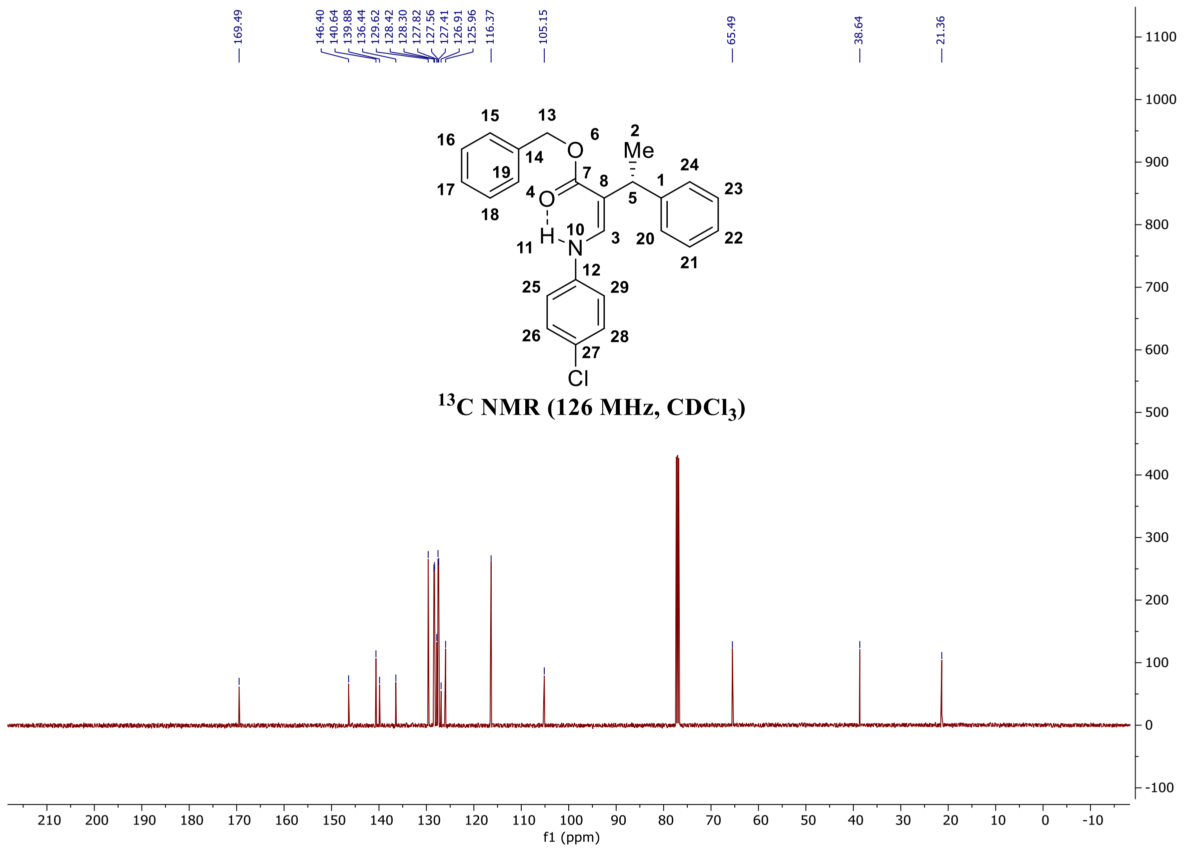

Benzyl (*R*,*Z*)-2-((phenylamino)methylene)-3-(*p*-tolyl)butanoate (3ab):

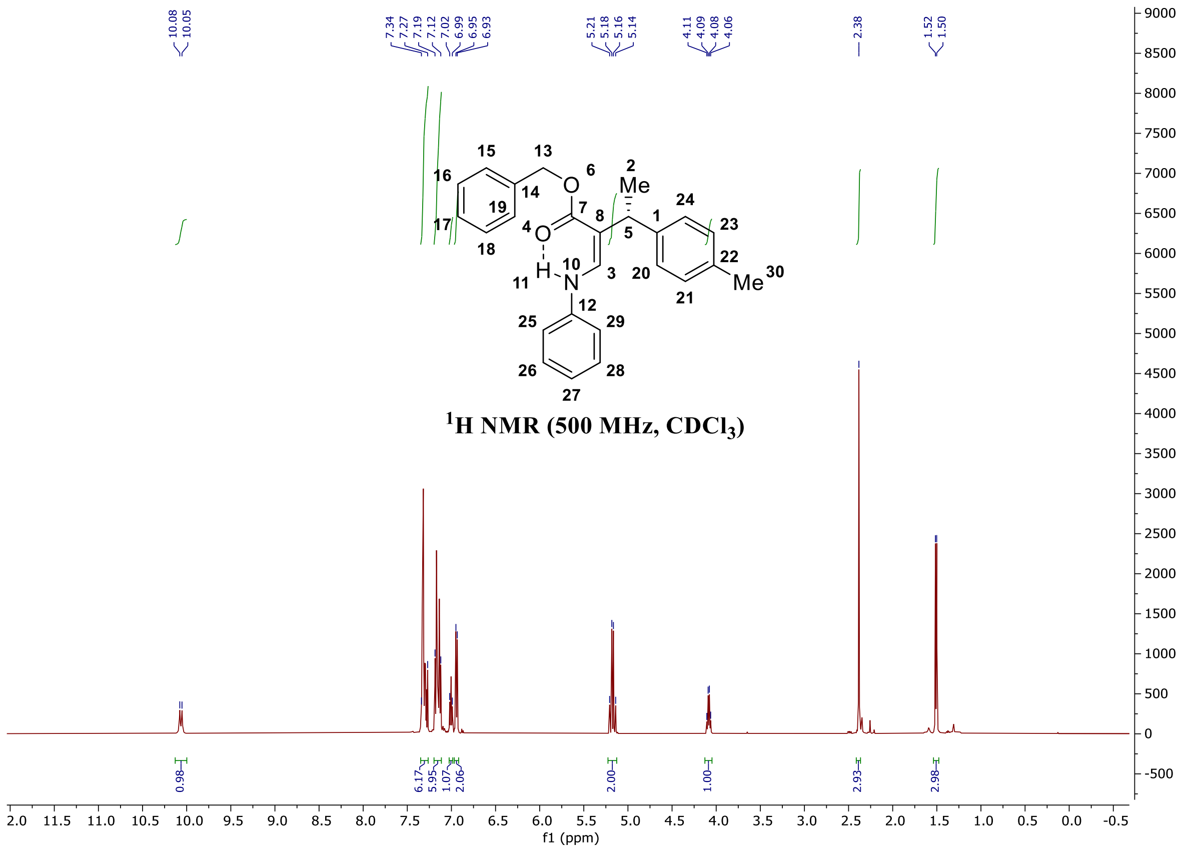

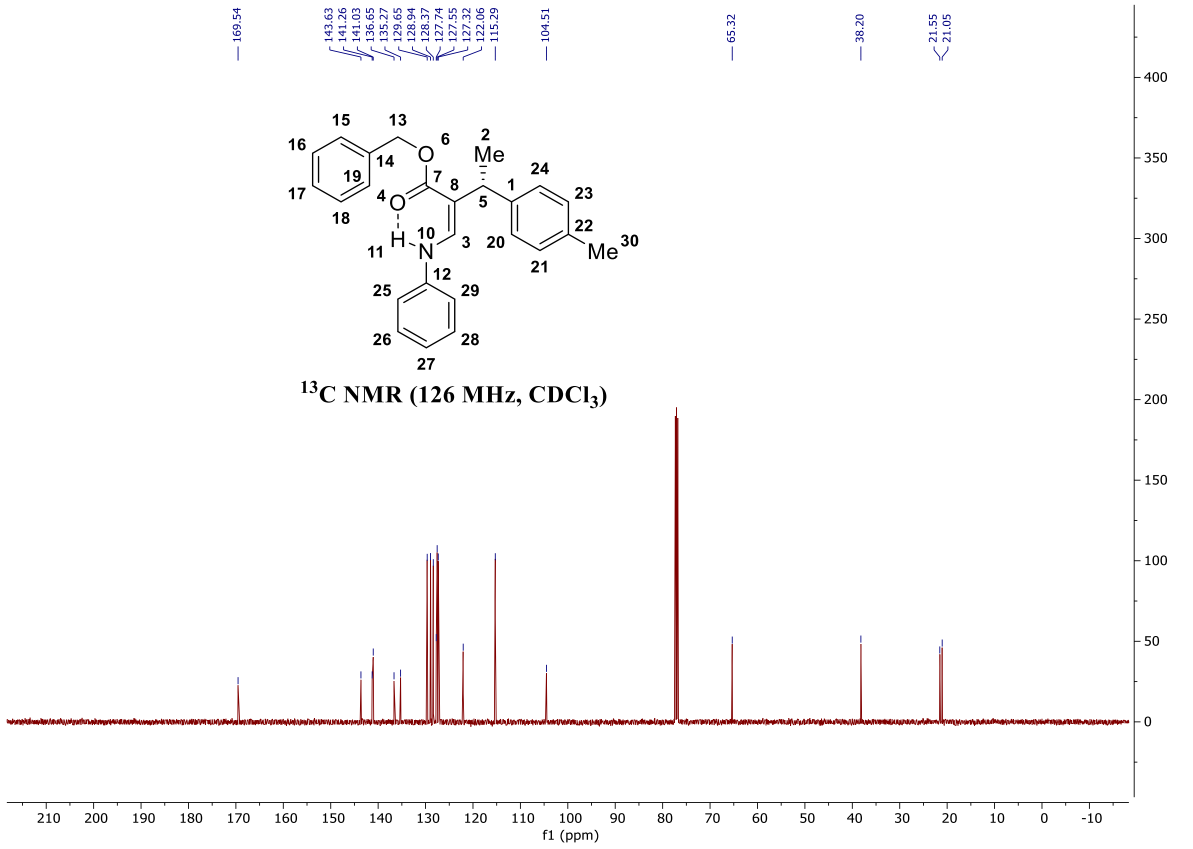

Benzyl (*R*,*Z*)-3-(4-methoxyphenyl)-2-((phenylamino)methylene)butanoate (3ac):

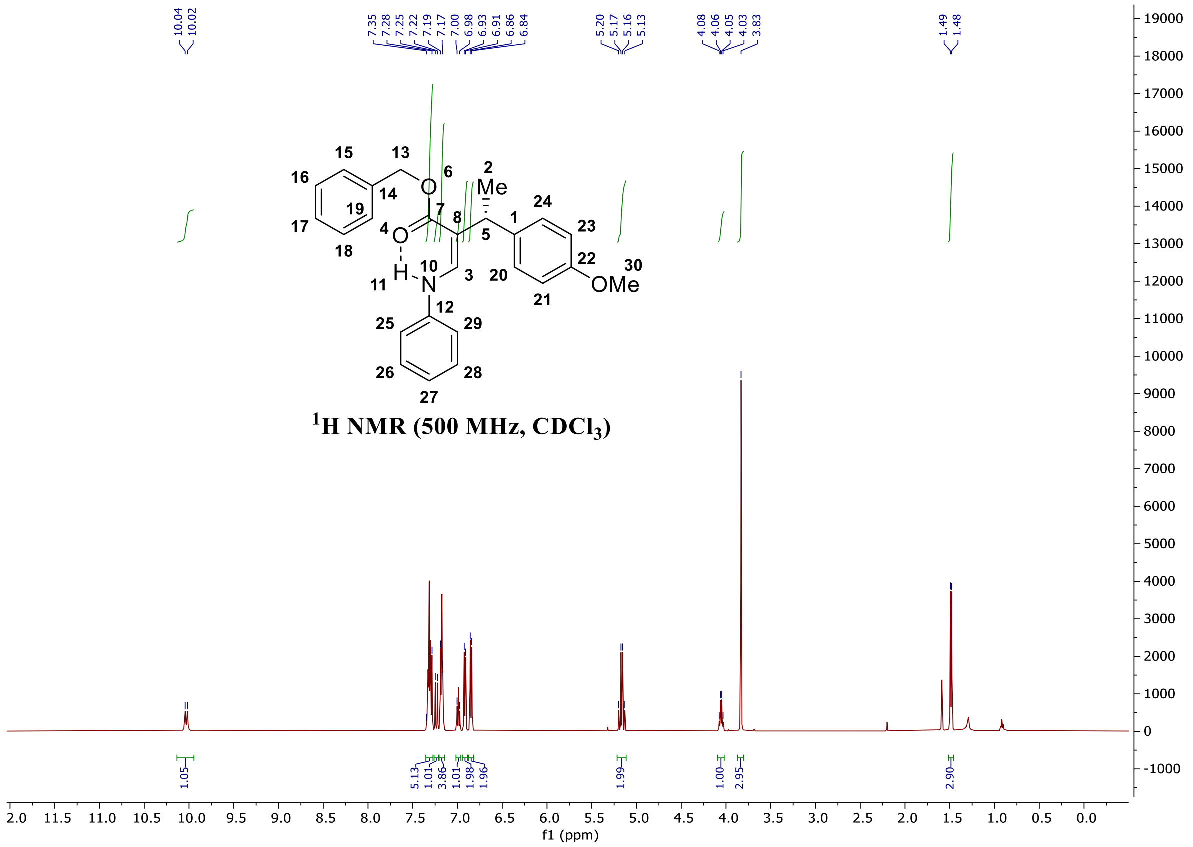

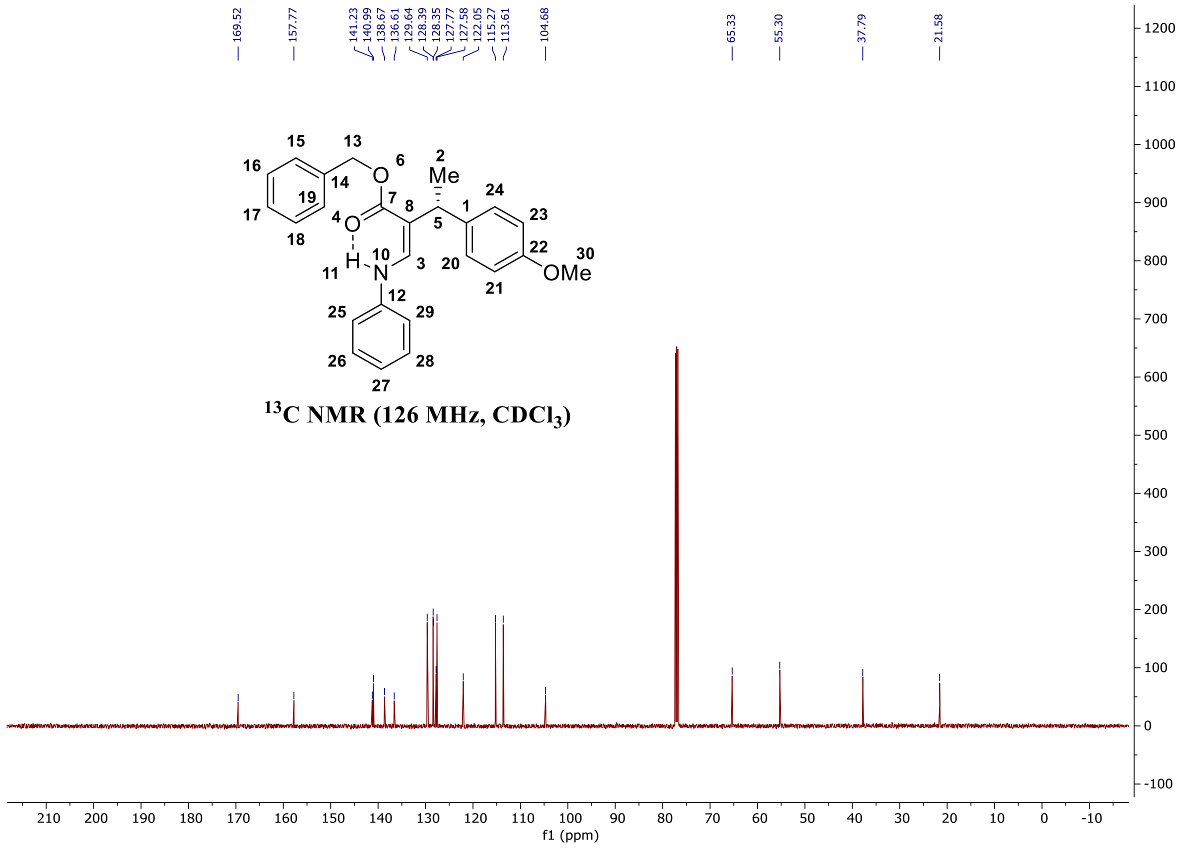

Benzyl (*R*,*Z*)-3-(4-(*tert*-butyl)phenyl)-2-((phenylamino)methylene)butanoate (3ad):

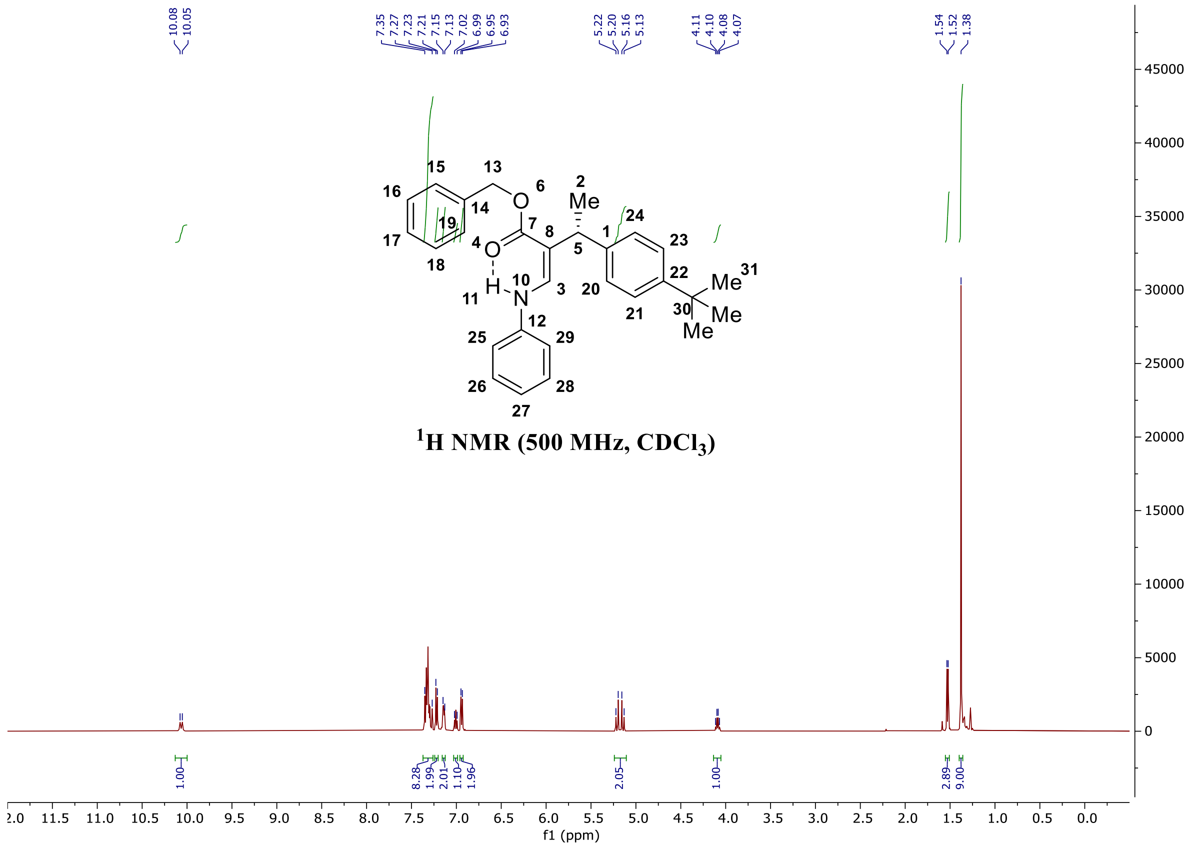

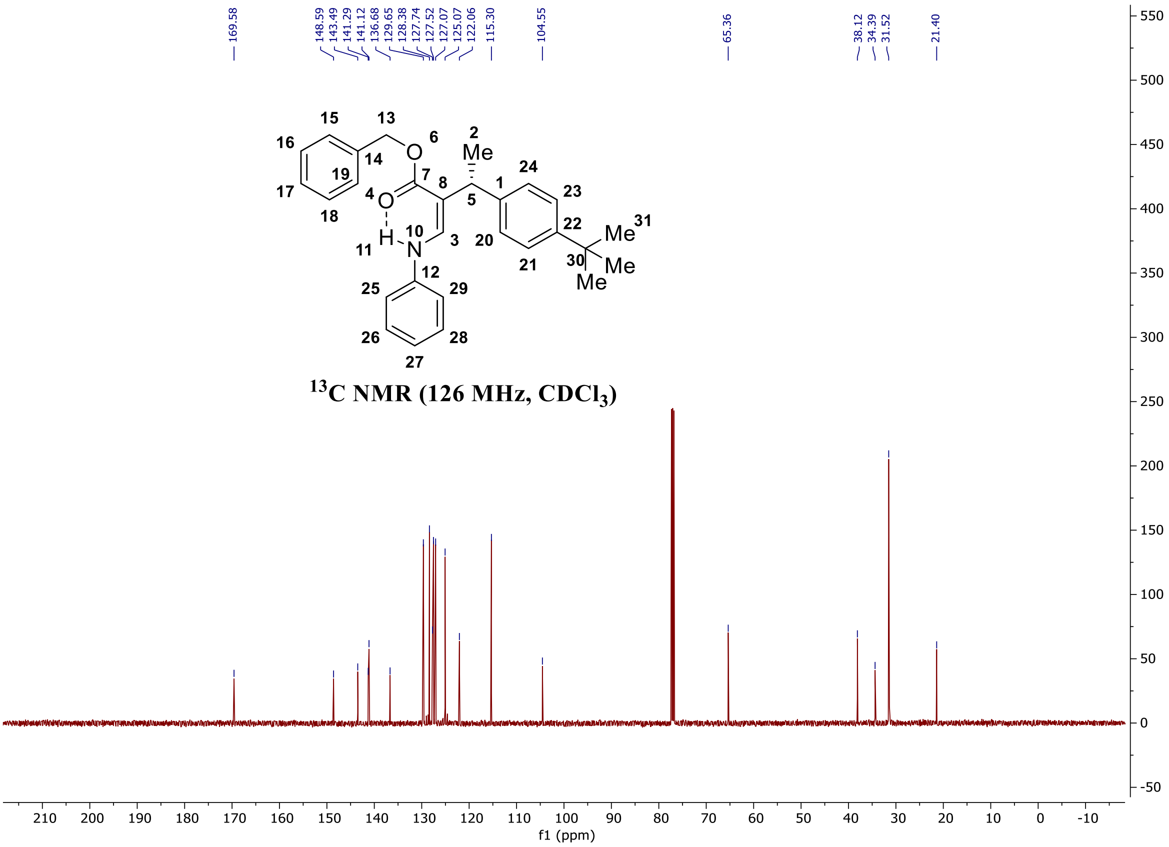

Benzyl (*R*,*Z*)-2-((phenylamino)methylene)-3-(4-(trimethylsilyl)phenyl)butanoate (3ae):

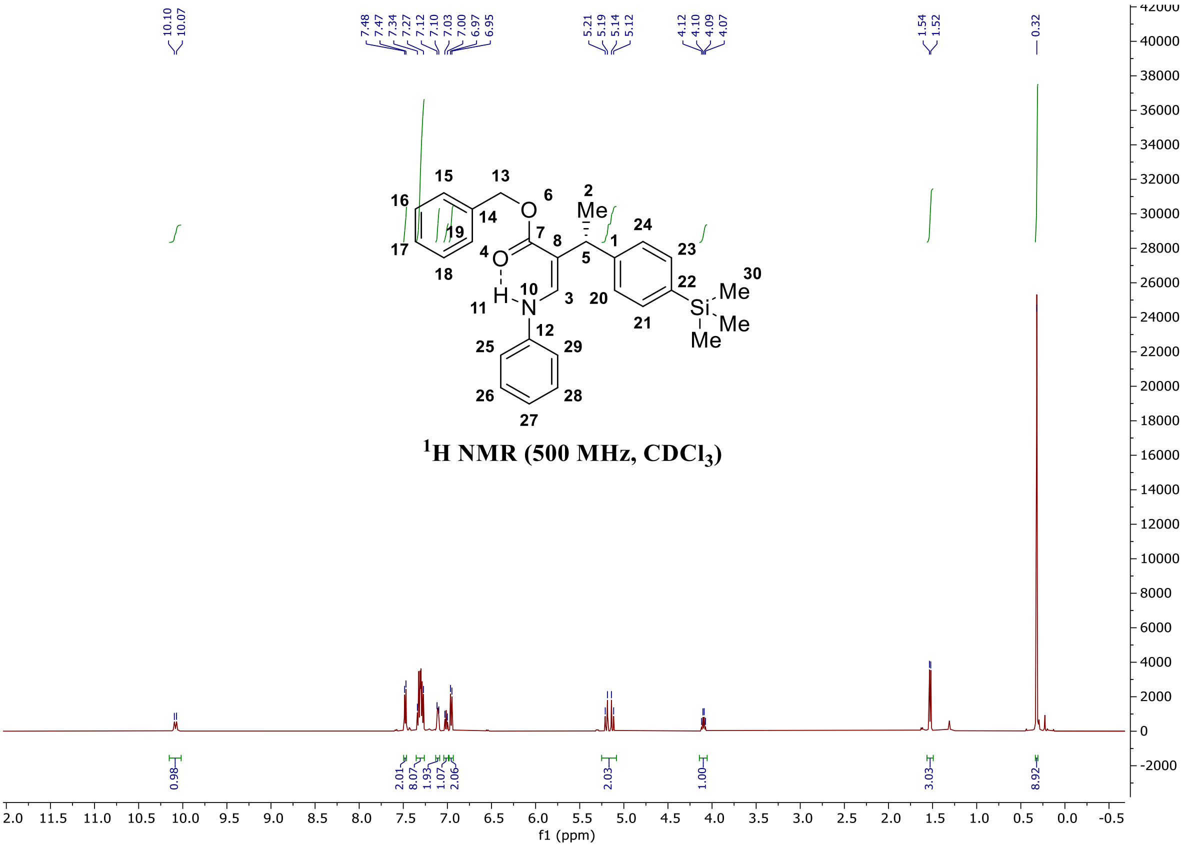

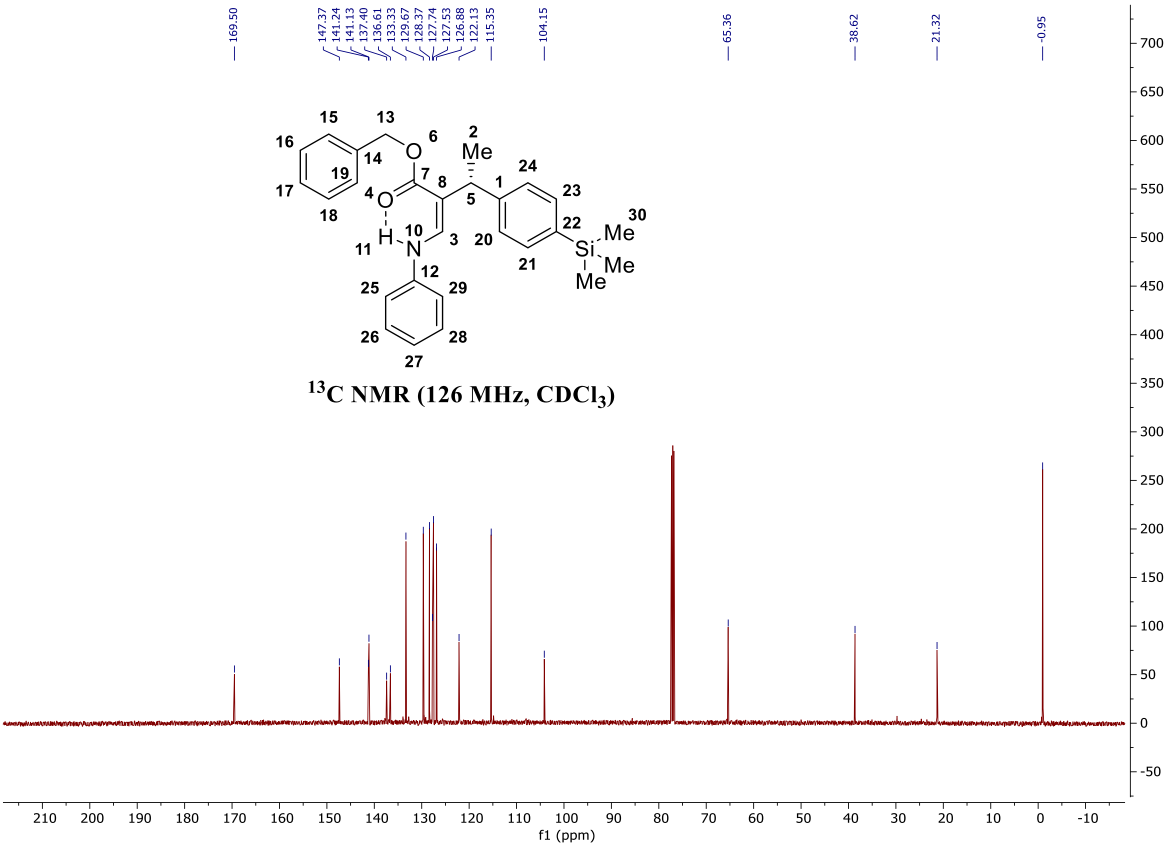

Benzyl (*R*,*Z*)-3-([1,1'-biphenyl]-4-yl)-2-((phenylamino)methylene)butanoate (3af):

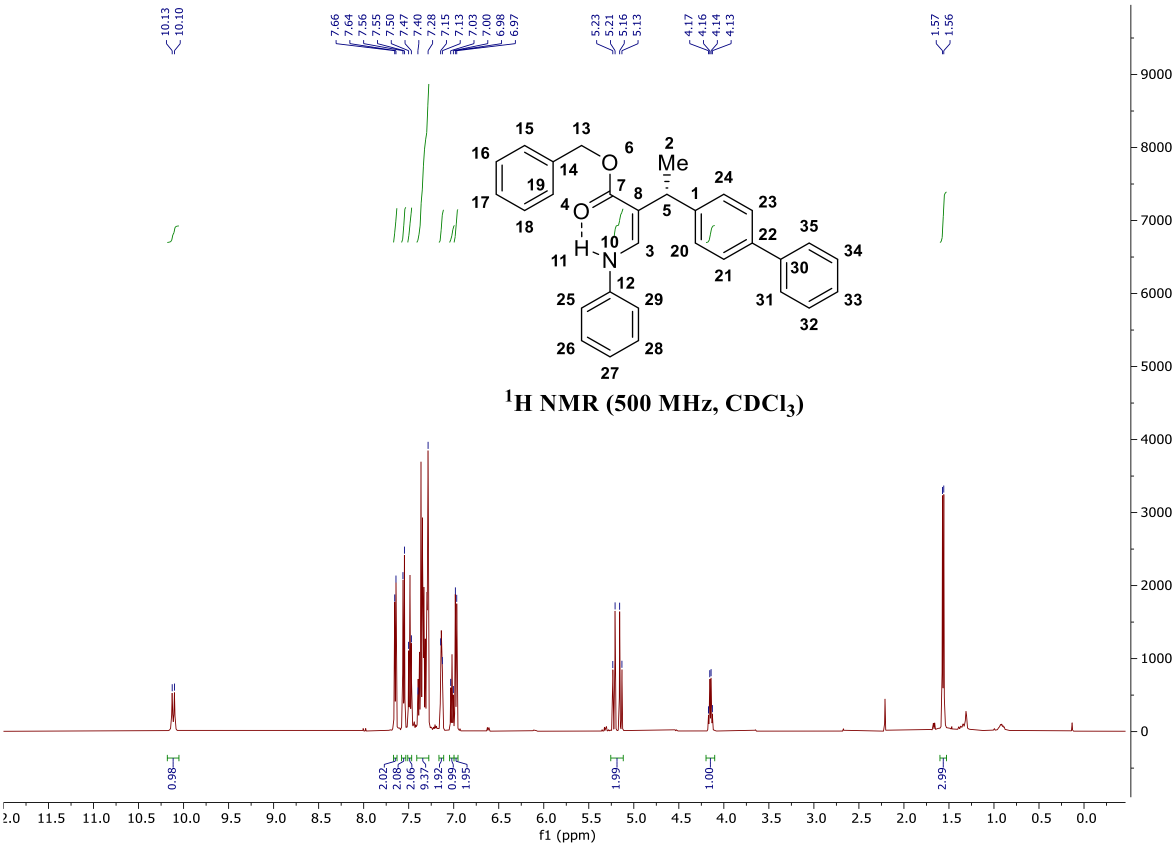

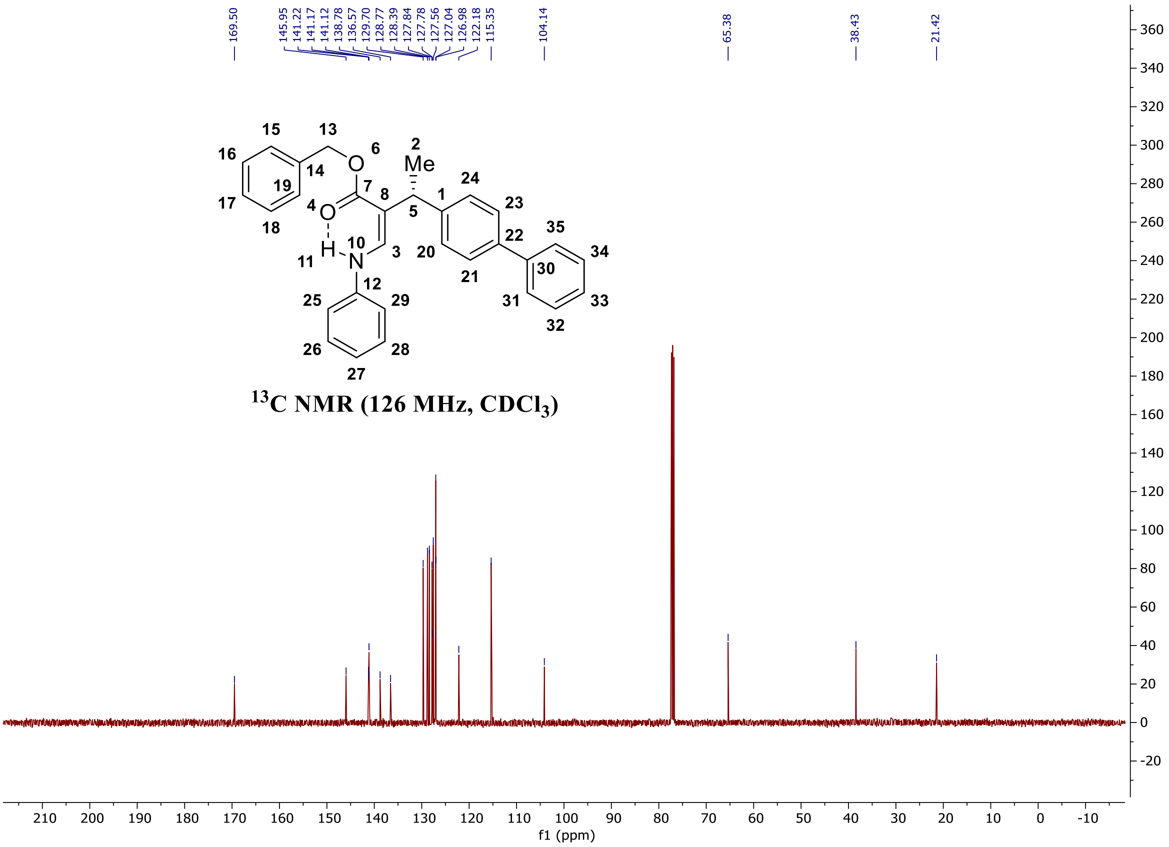

Benzyl (*R*,*Z*)-3-(4-fluorophenyl)-2-((phenylamino)methylene)butanoate (3ag):

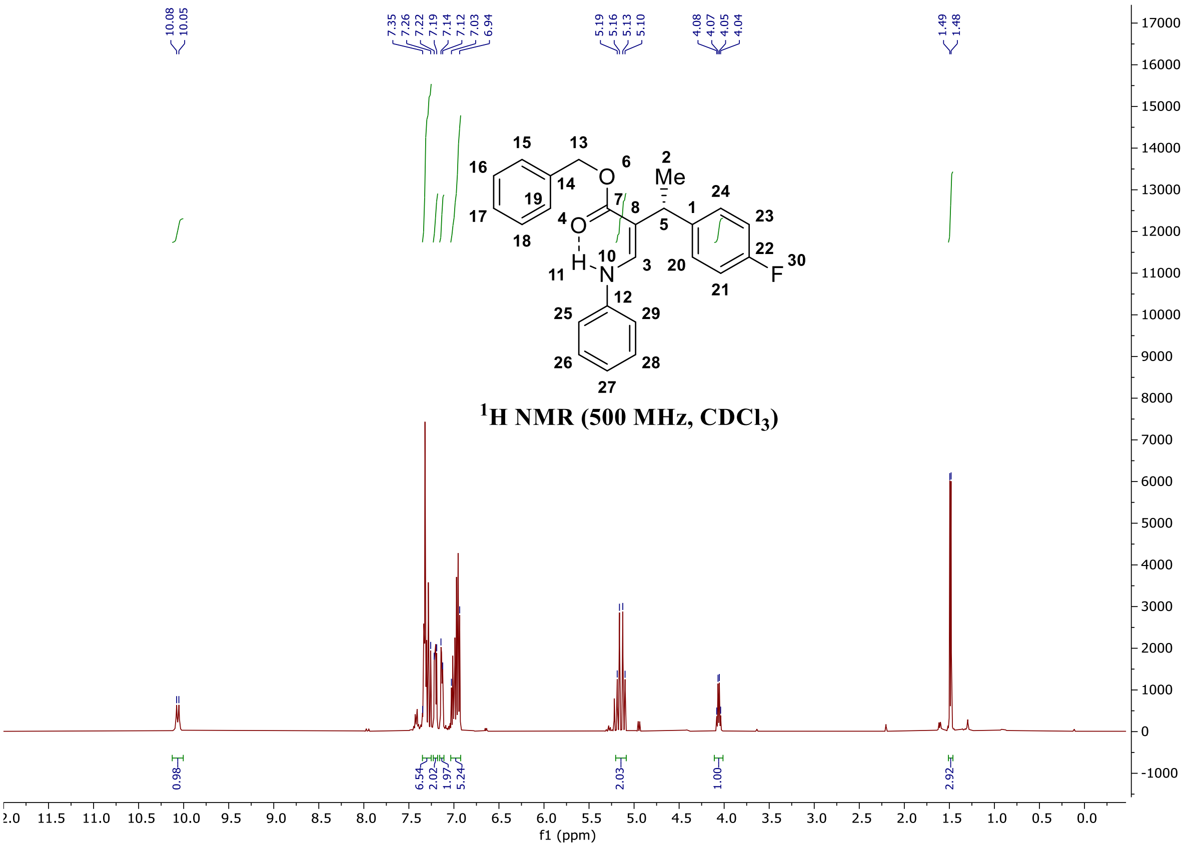

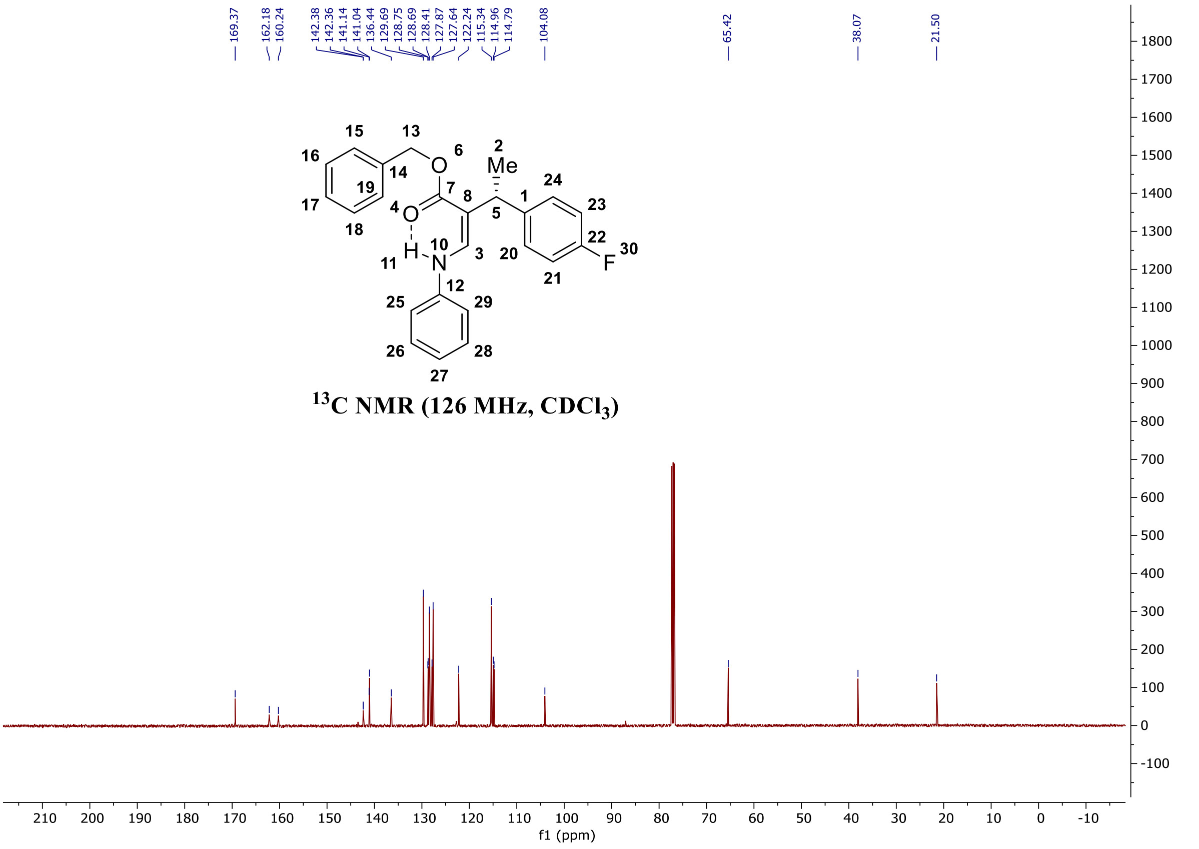
169.37
162.18
160.24
142.38
142.36
141.14
141.04
136.44
129.69
128.75
128.69
128.41
127.87
127.64
122.24
115.34
114.96
114.79
104.08
65.42
38.07
21.50
^{13}C NMR (126 MHz, CDCl$_3$)
f1 (ppm)

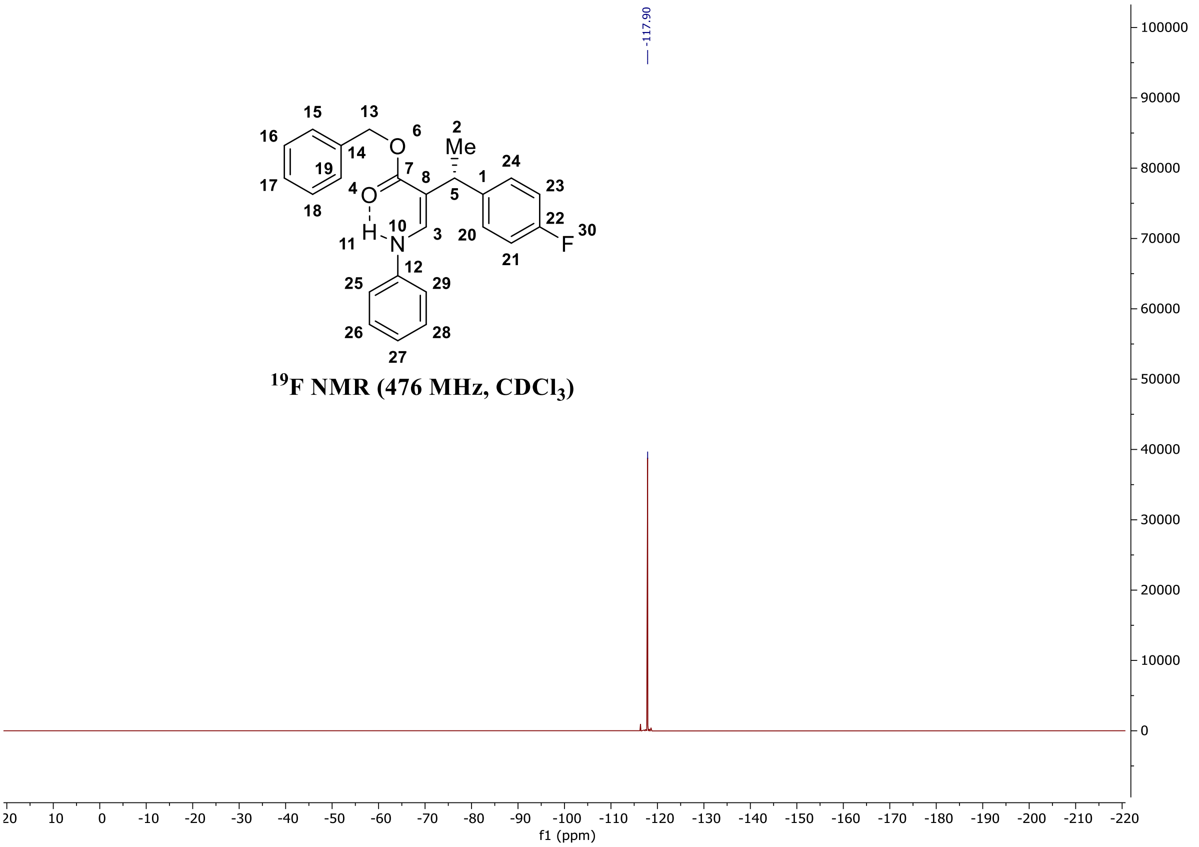
-117.90
^{19}F NMR (476 MHz, CDCl$_3$)
f1 (ppm)

Benzyl (*R*,*Z*)-3-(4-bromophenyl)-2-((phenylamino)methylene)butanoate (3ah):

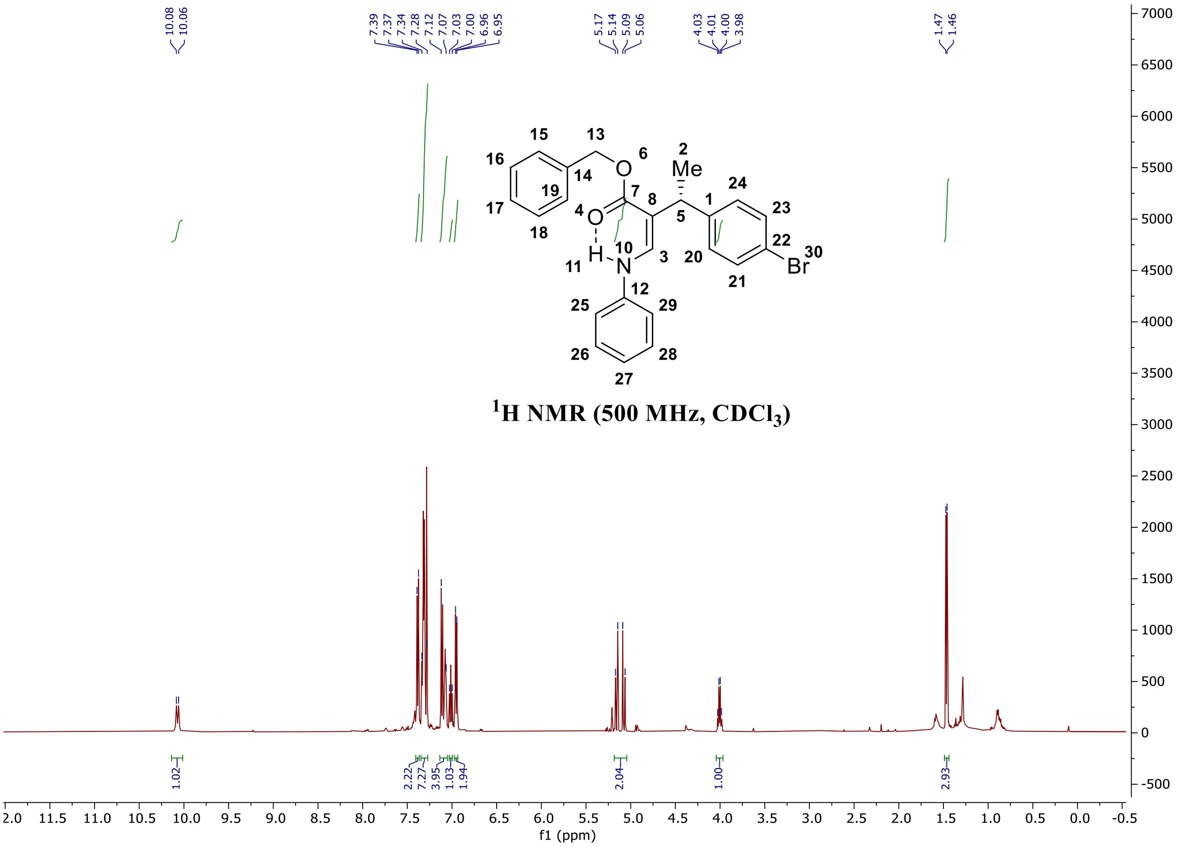

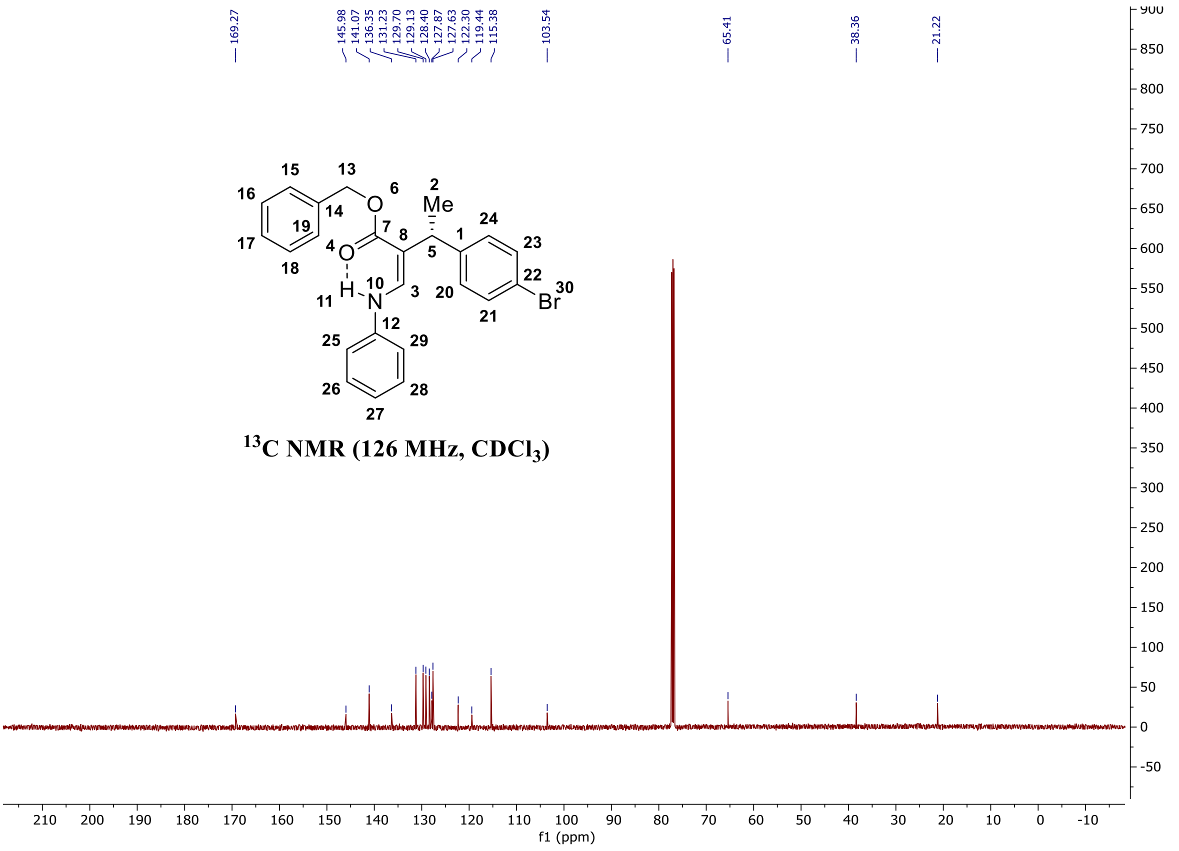

Benzyl (*R*,*Z*)-2-((phenylamino)methylene)-3-(4-(4,4,5,5-tetramethyl-1,3,2-dioxaborolan-2-yl)phenyl)butanoate (3ai):

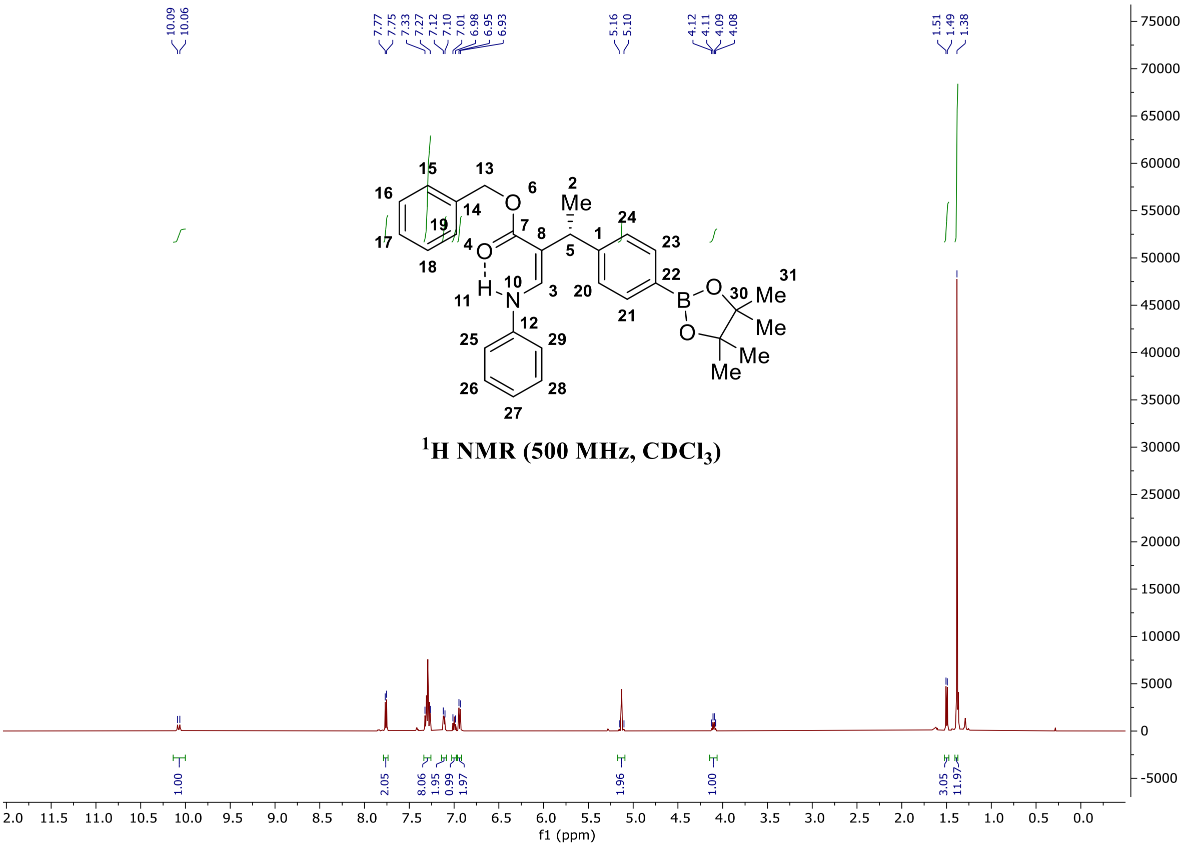

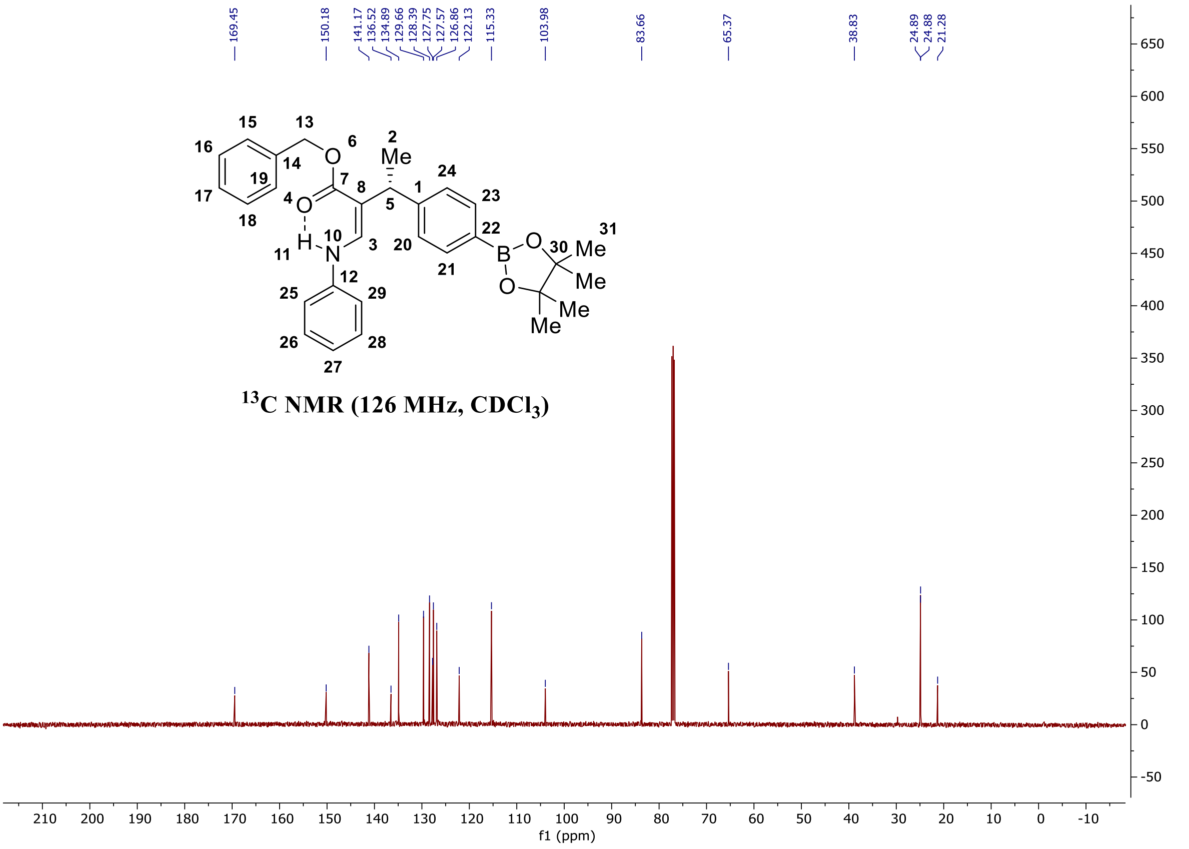

Benzyl (*R*,*Z*)-3-(4-(*N*,*N*-diethylsulfamoyl)phenyl)-2-((phenylamino)methylene)butanoate (3aj):

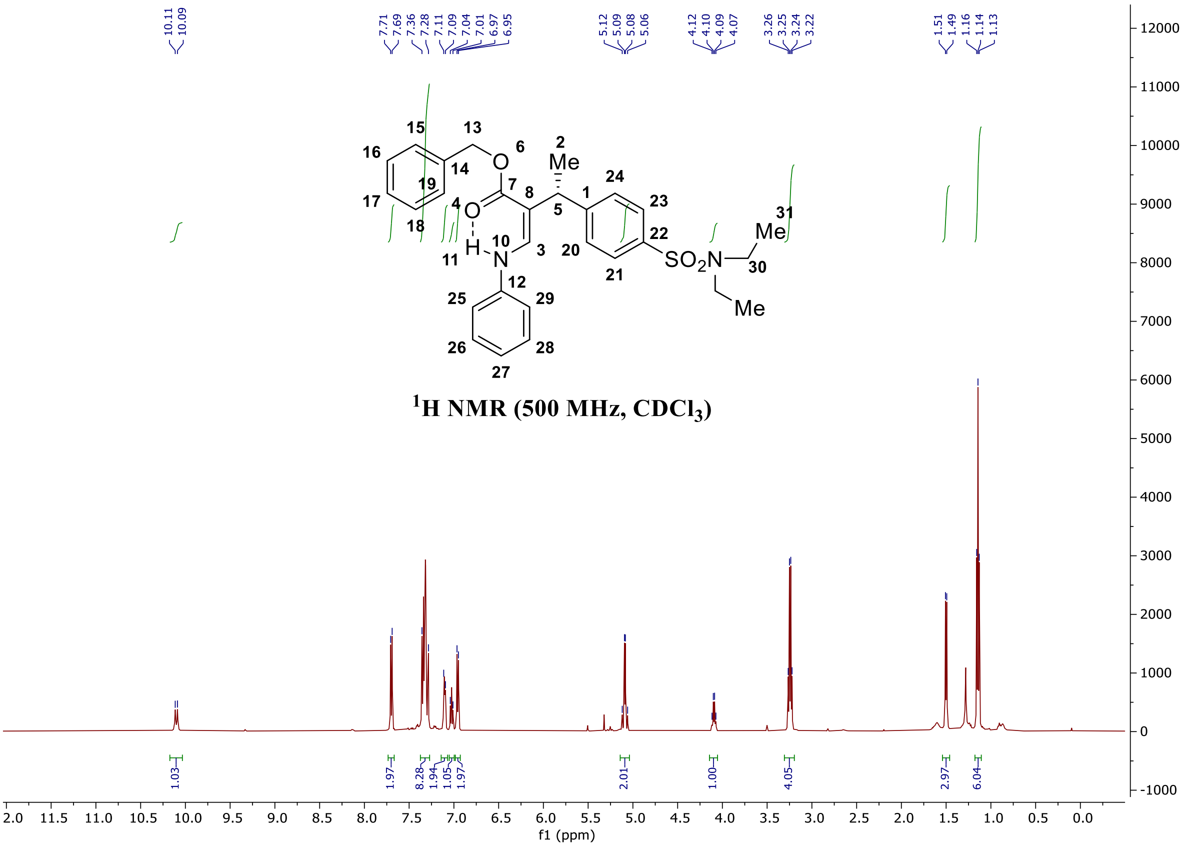

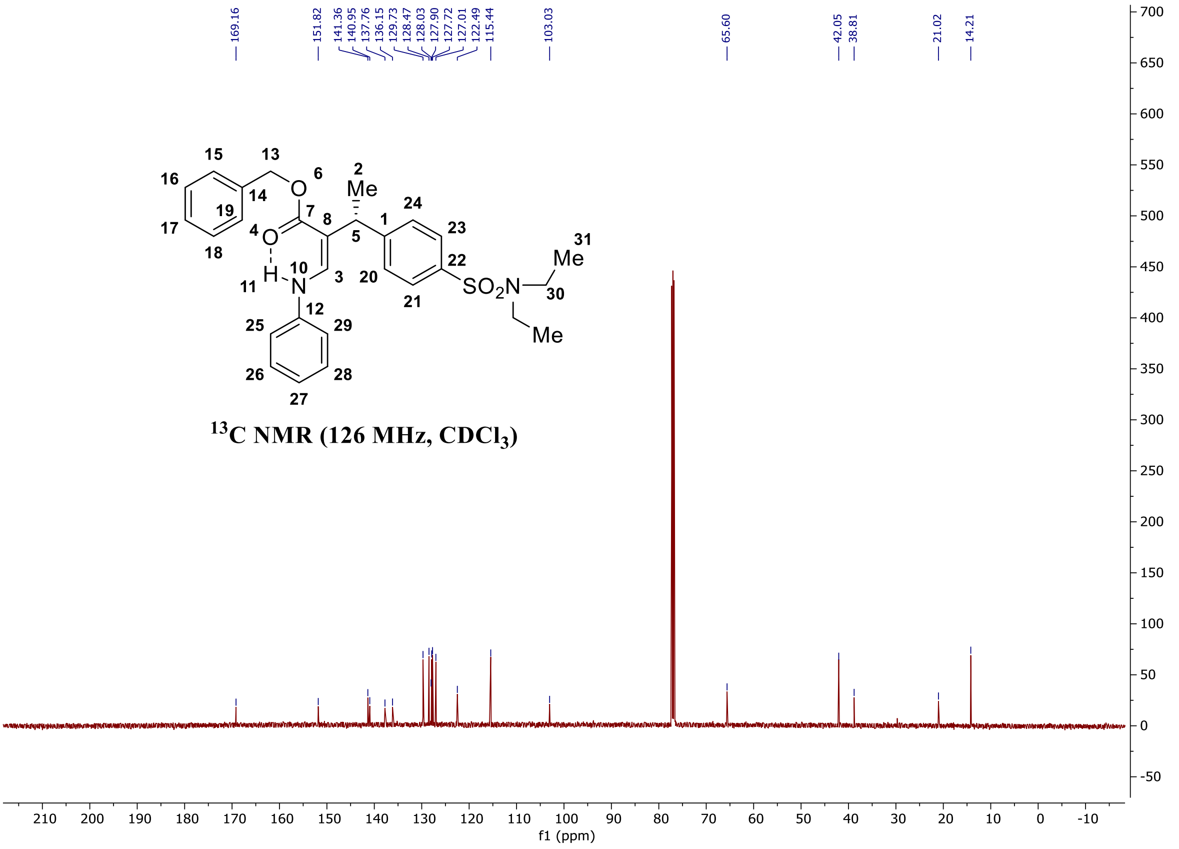

Benzyl (*R*,*Z*)-3-(3-chlorophenyl)-2-((phenylamino)methylene)butanoate (3ak):

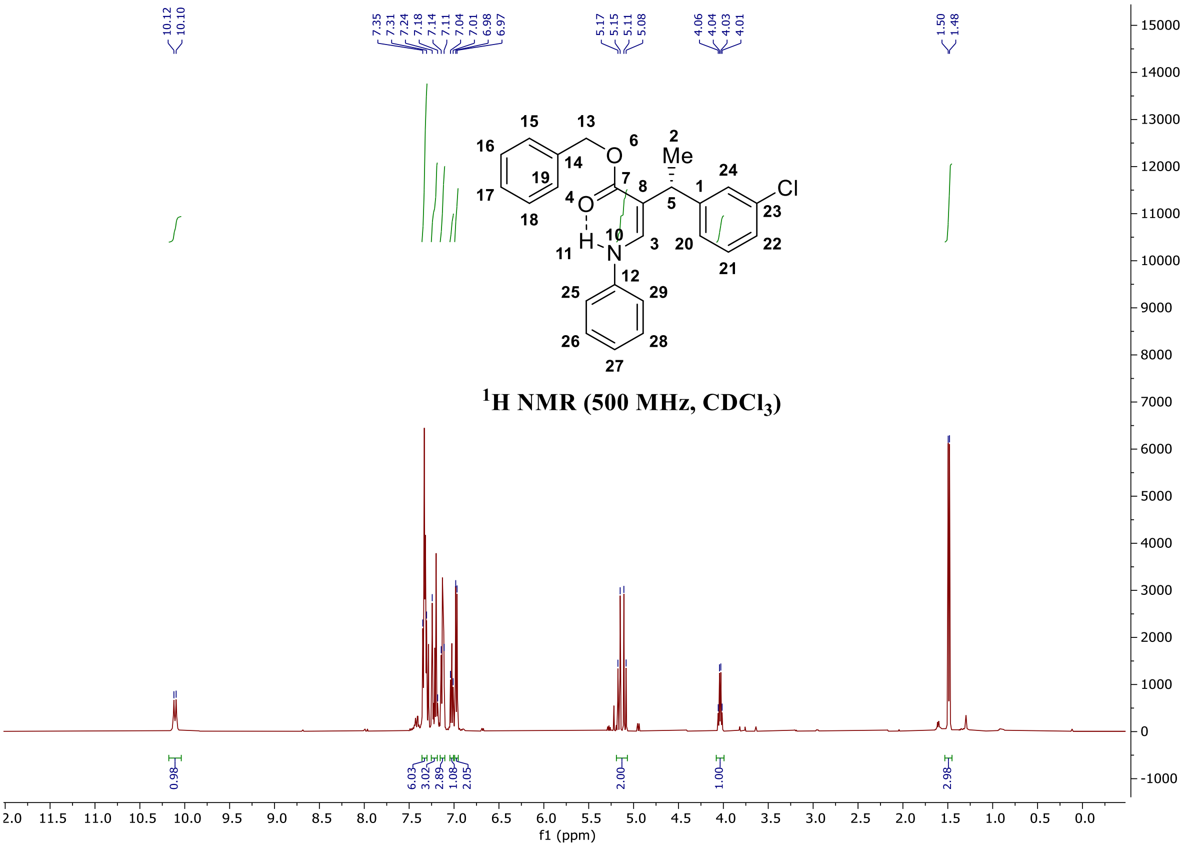

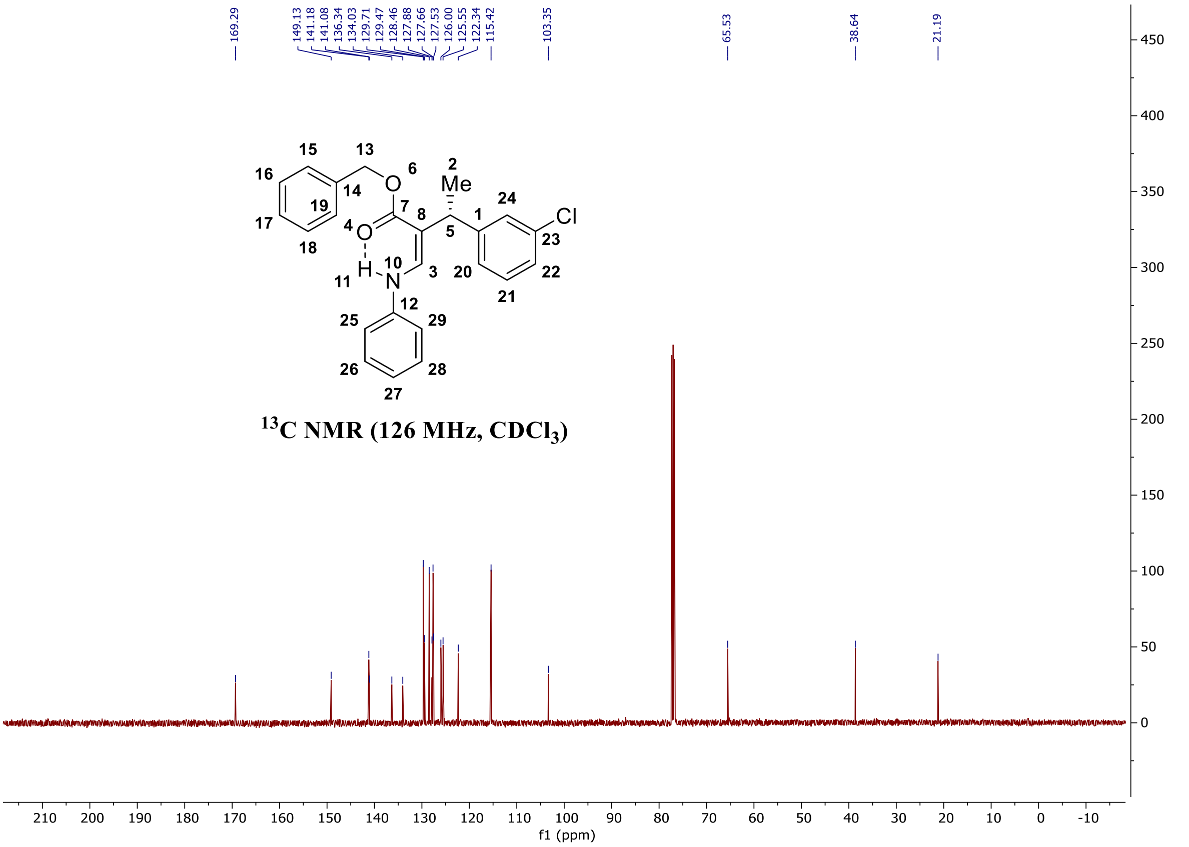

Benzyl (*S*,*Z*)-3-(2-chlorophenyl)-2-((phenylamino)methylene)butanoate (3al):

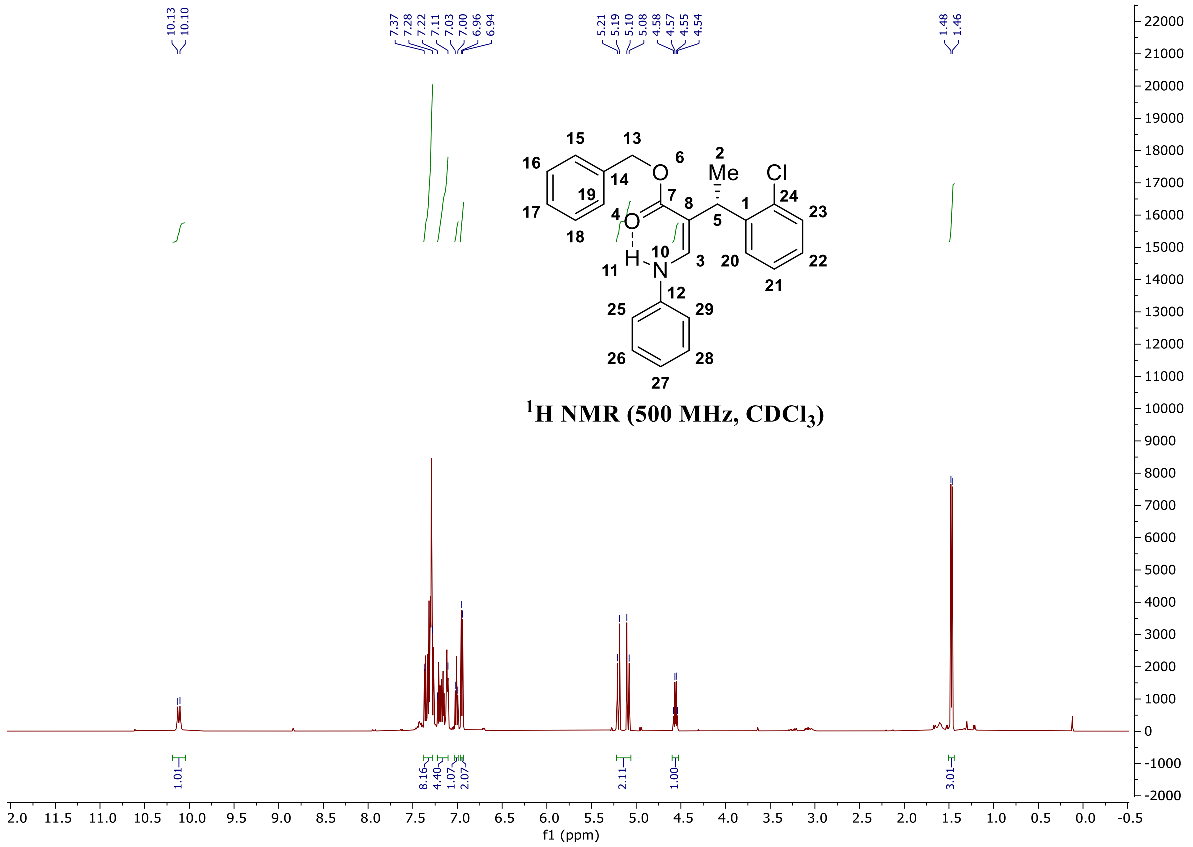

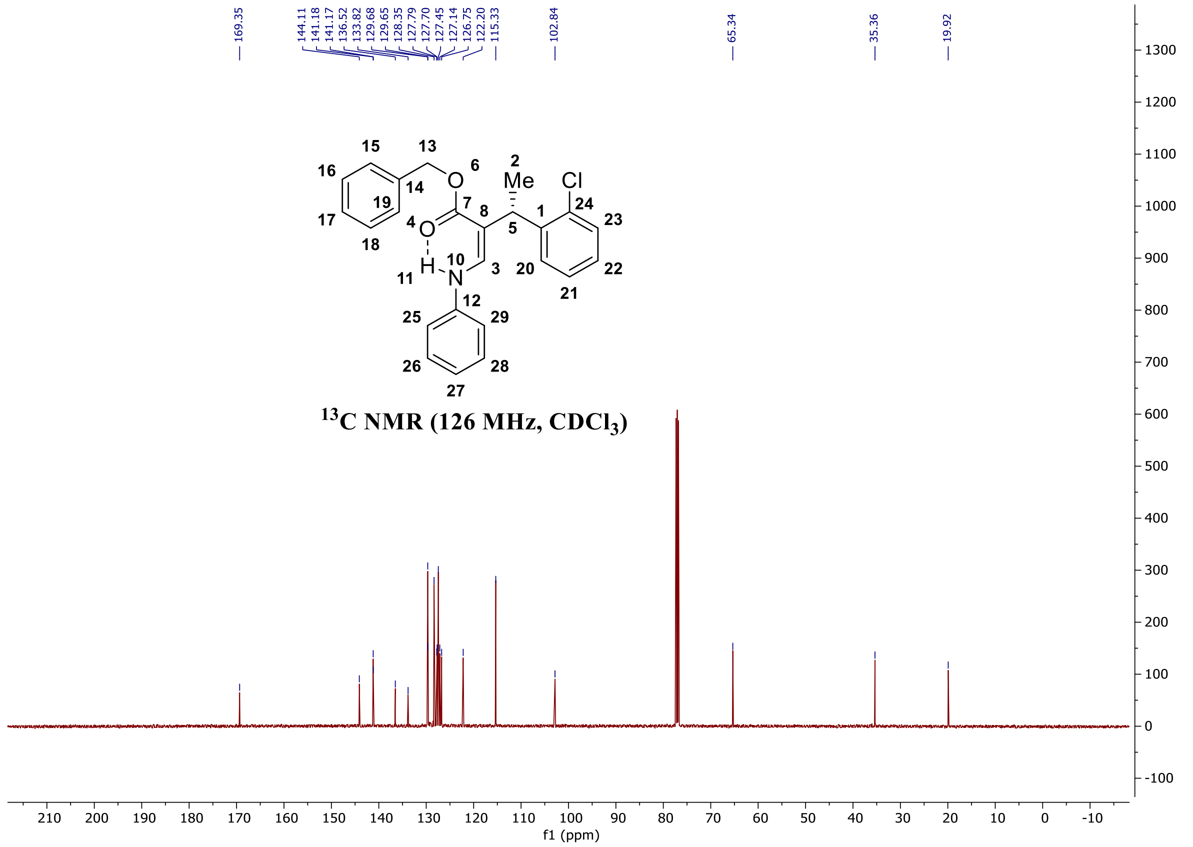

Benzyl (*R*,*Z*)-2-((phenylamino)methylene)-3-(*o*-tolyl)butanoate (3am):

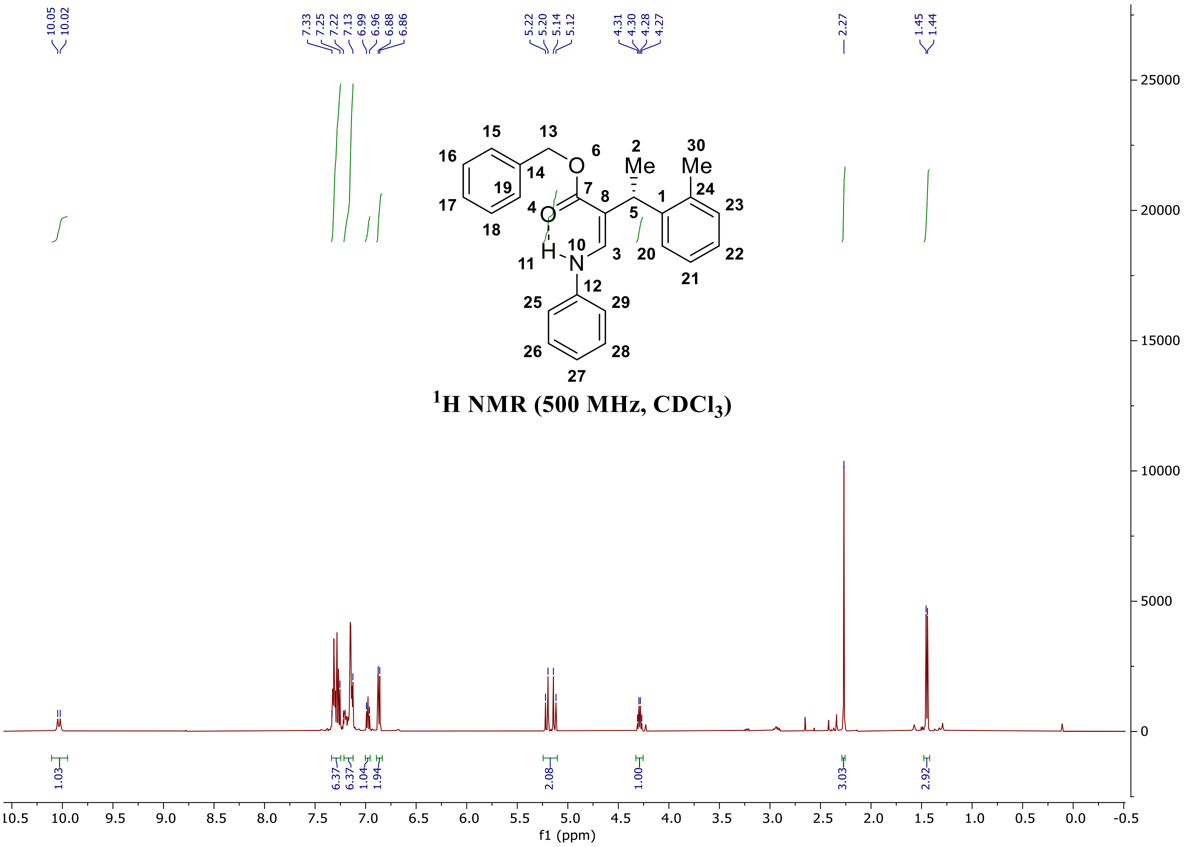

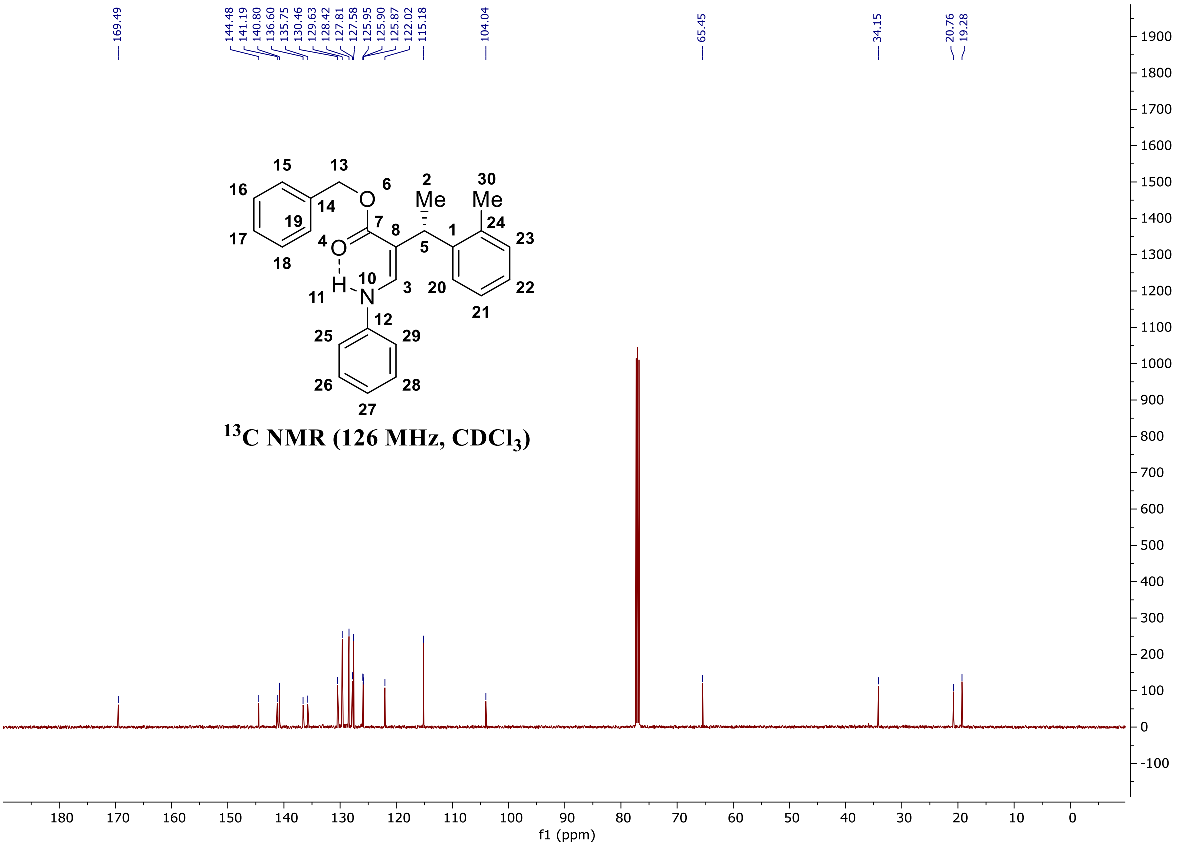

Benzyl (*S*,*Z*)-3-(perfluorophenyl)-2-((phenylamino)methylene)butanoate (3an):

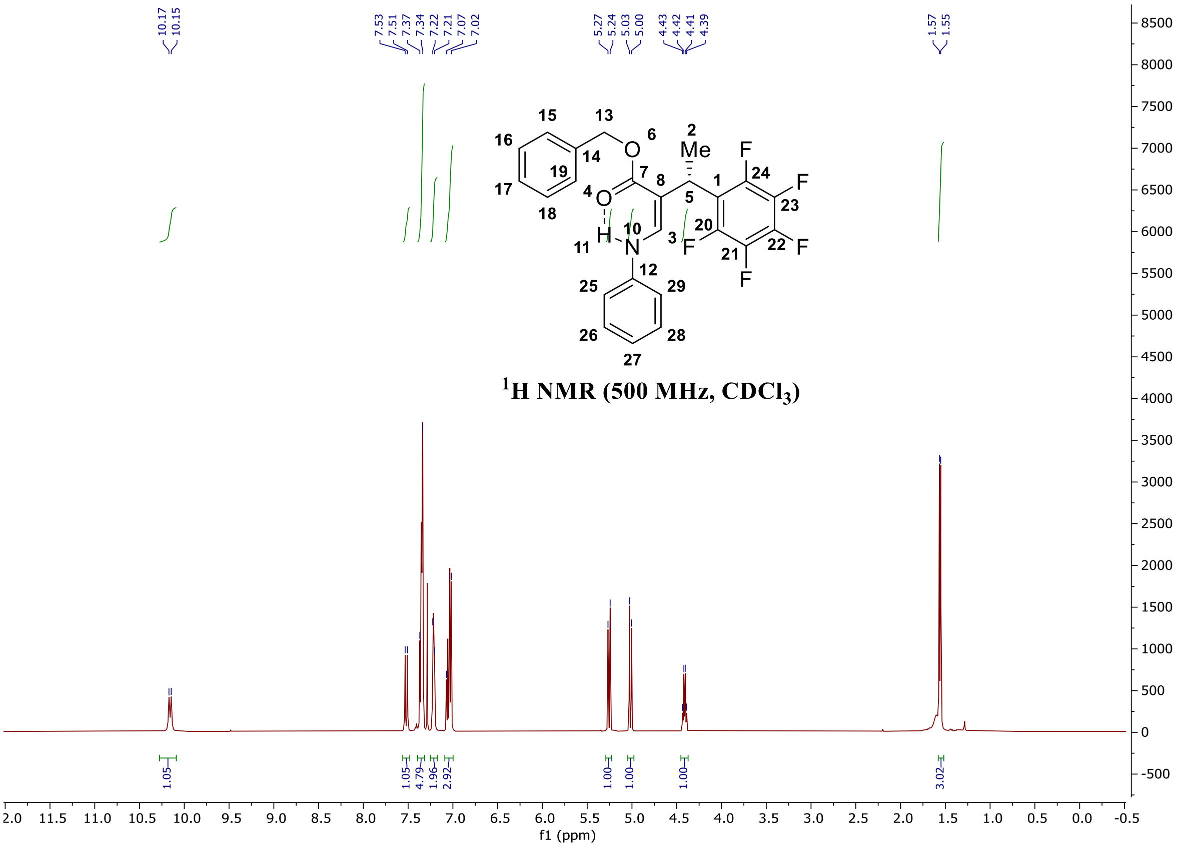

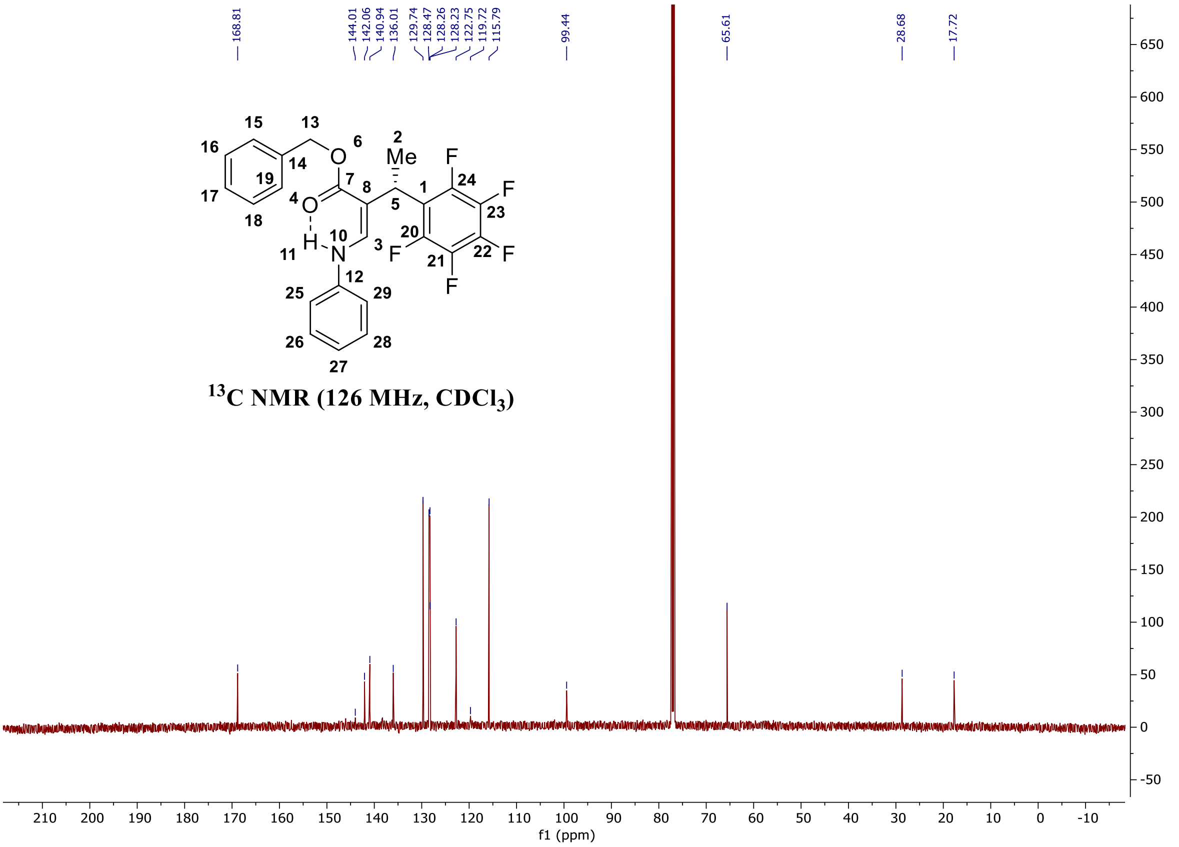

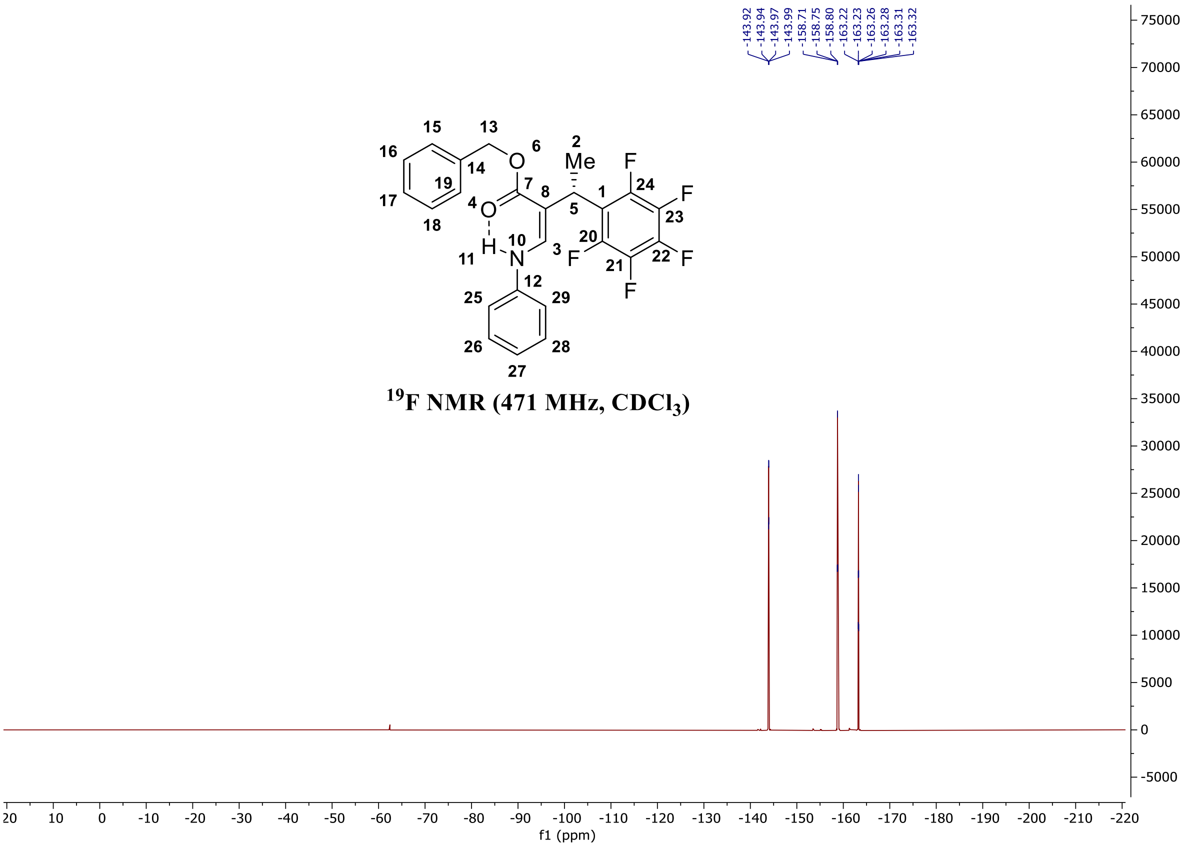

Benzyl (*R*,*Z*)-2-((phenylamino)methylene)-3-(1-tosyl-1*H*-indol-3-yl)butanoate (3ao):

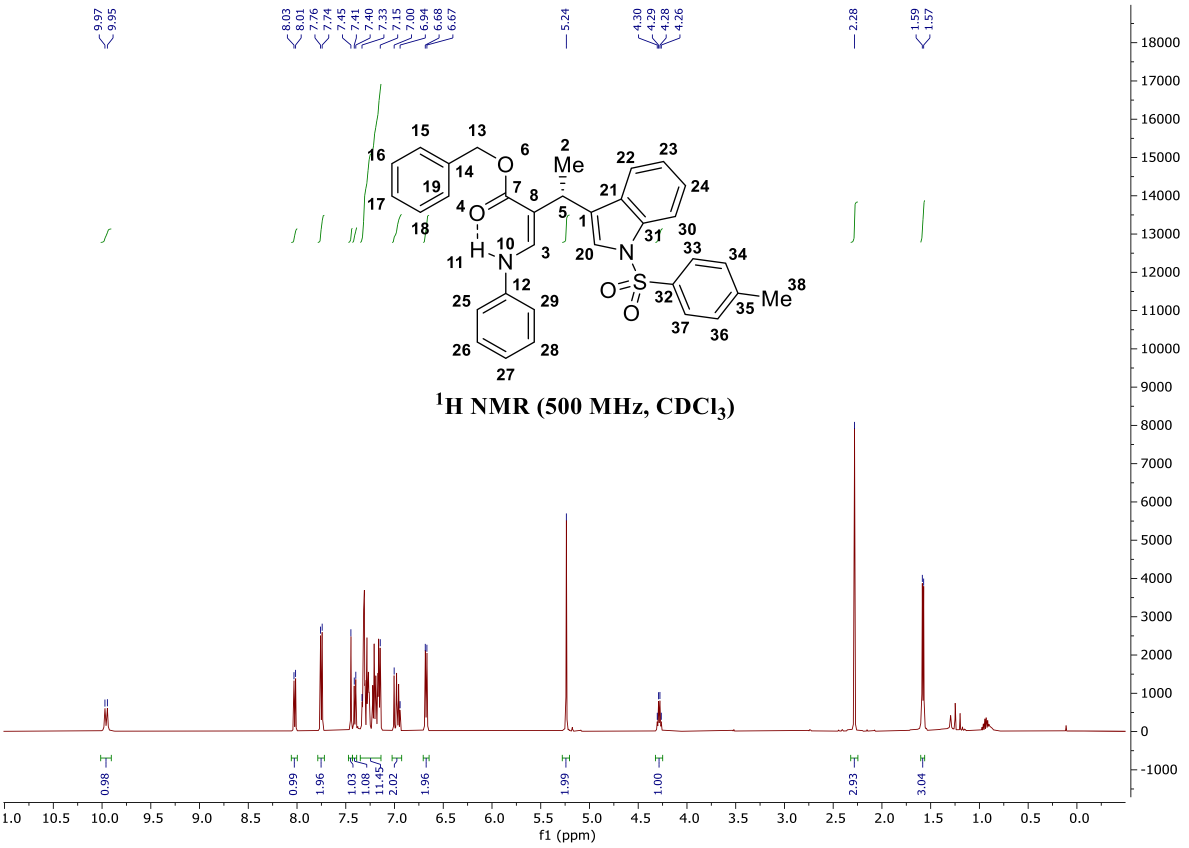

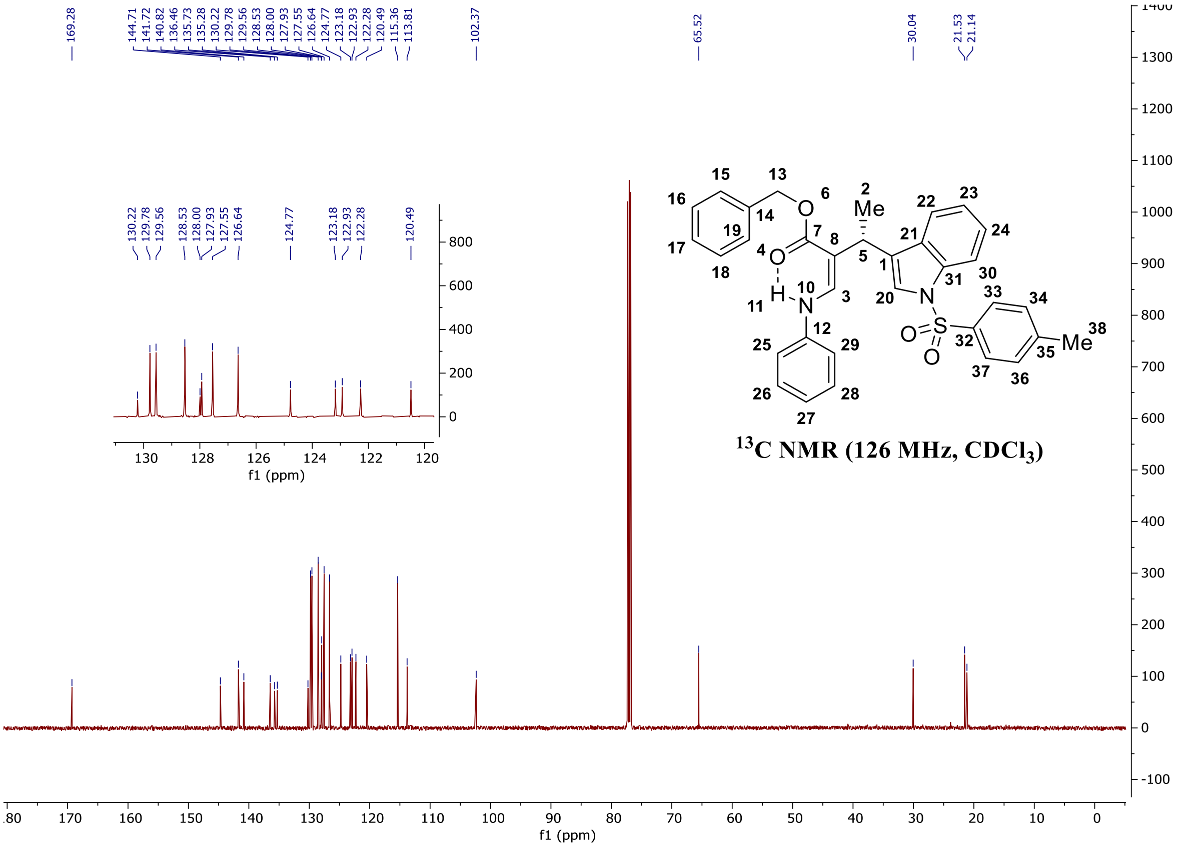

Benzyl (*R*,*Z*)-3-(benzofuran-5-yl)-2-((phenylamino)methylene)butanoate (3ap):

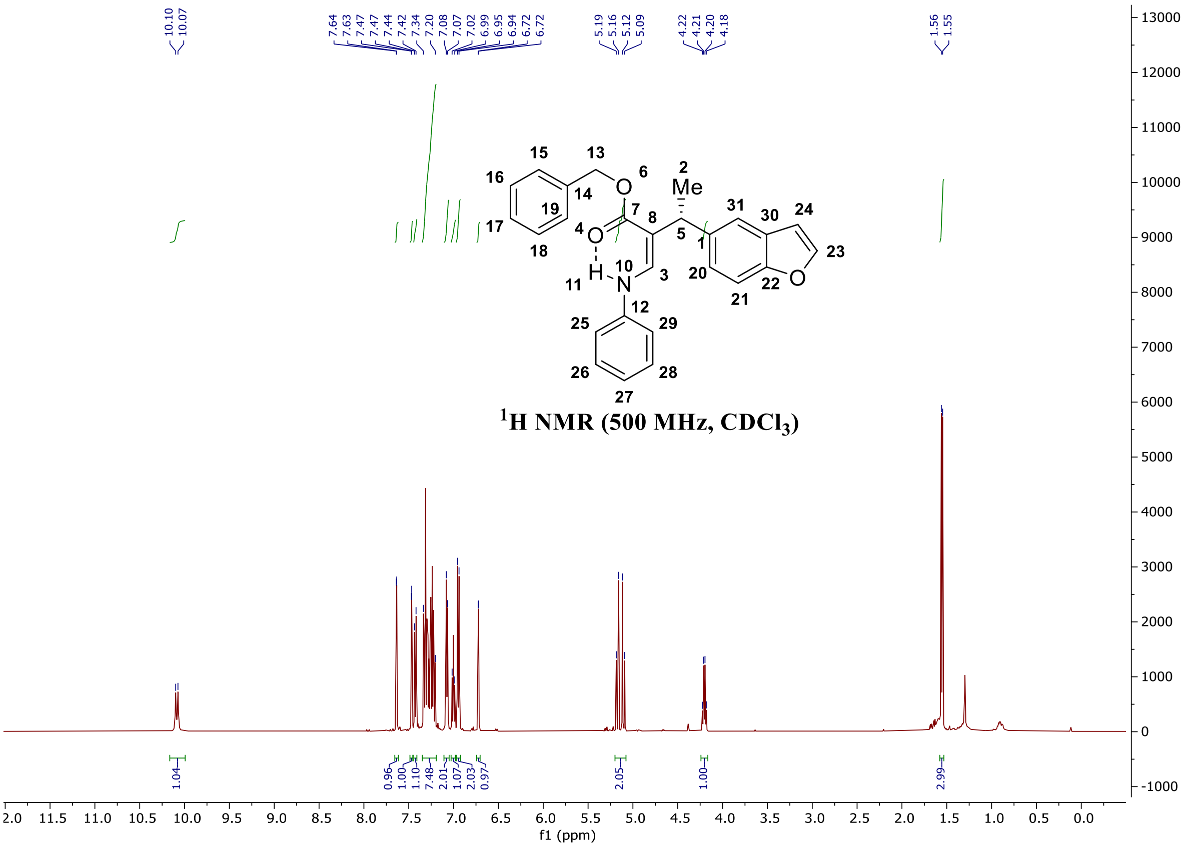

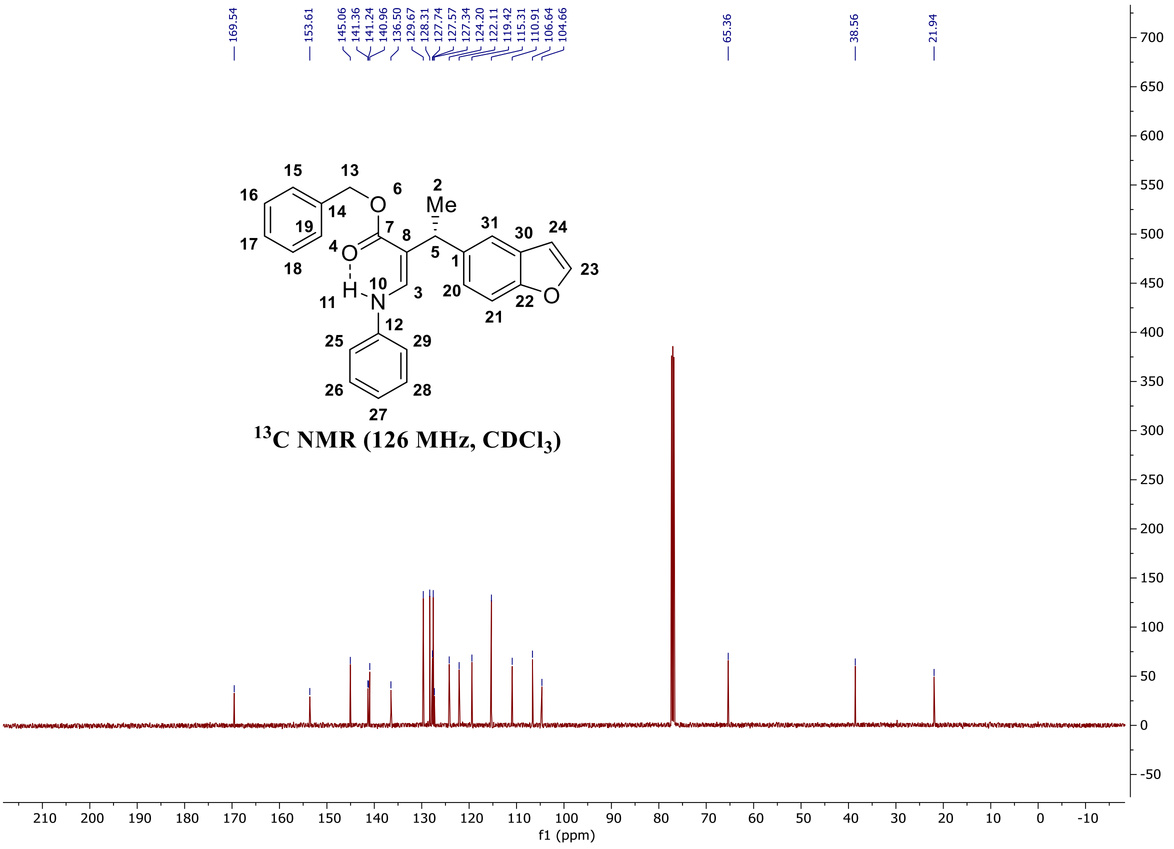

Compound (3aq):

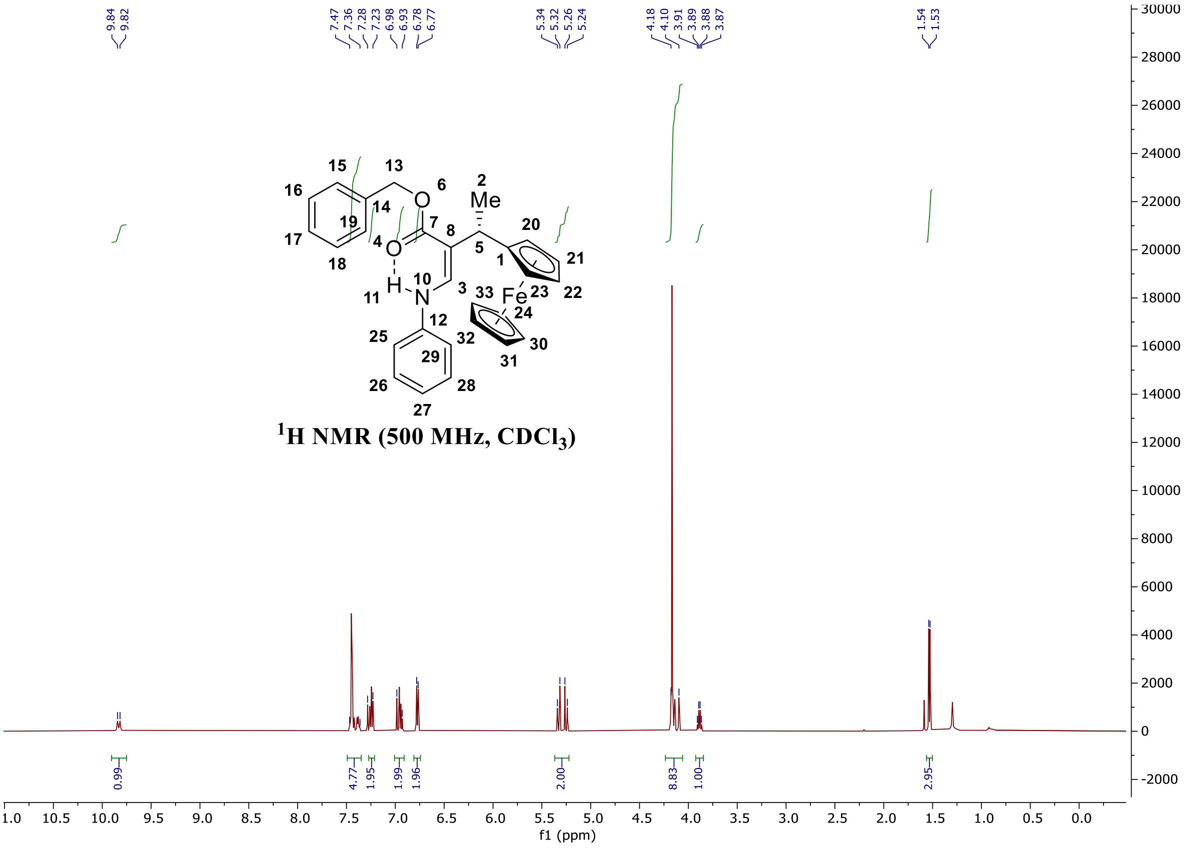

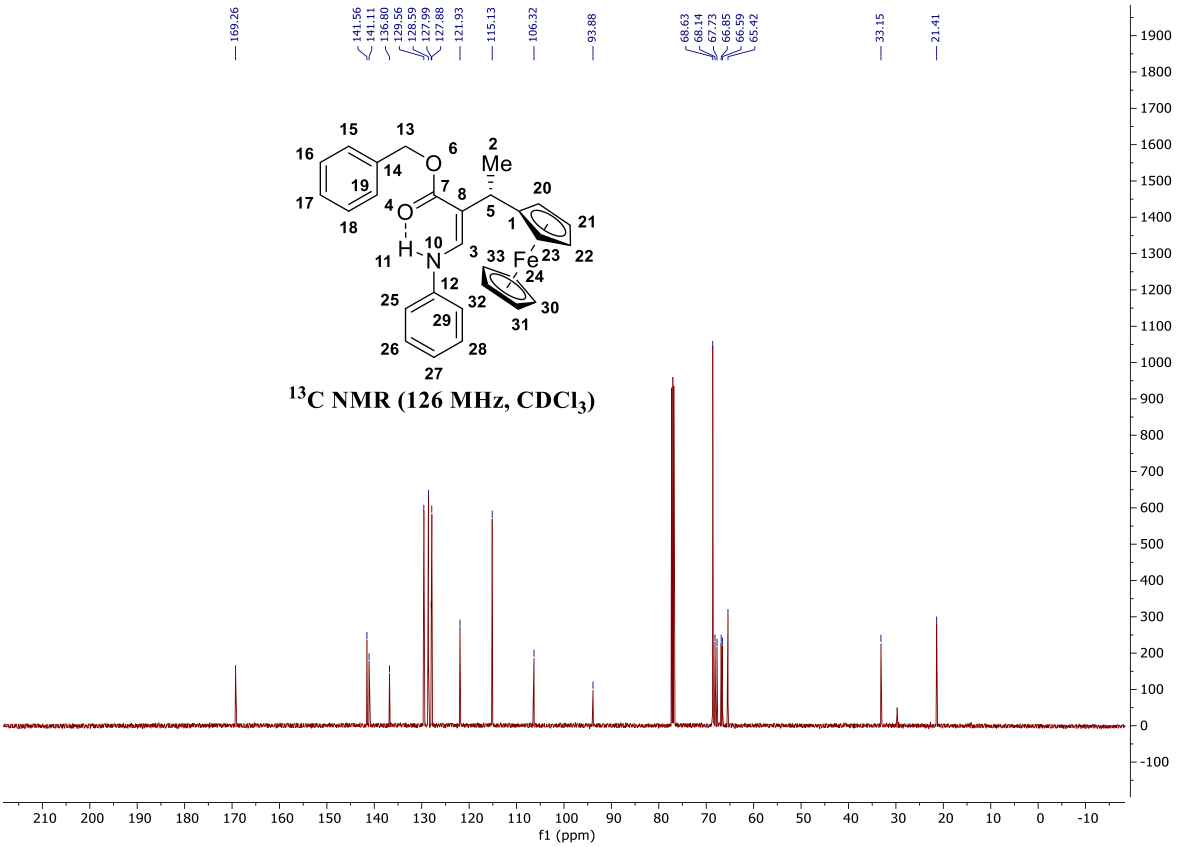

Benzyl (*R*,*Z*)-3-(naphthalen-2-yl)-2-((phenylamino)methylene)butanoate (3ar):

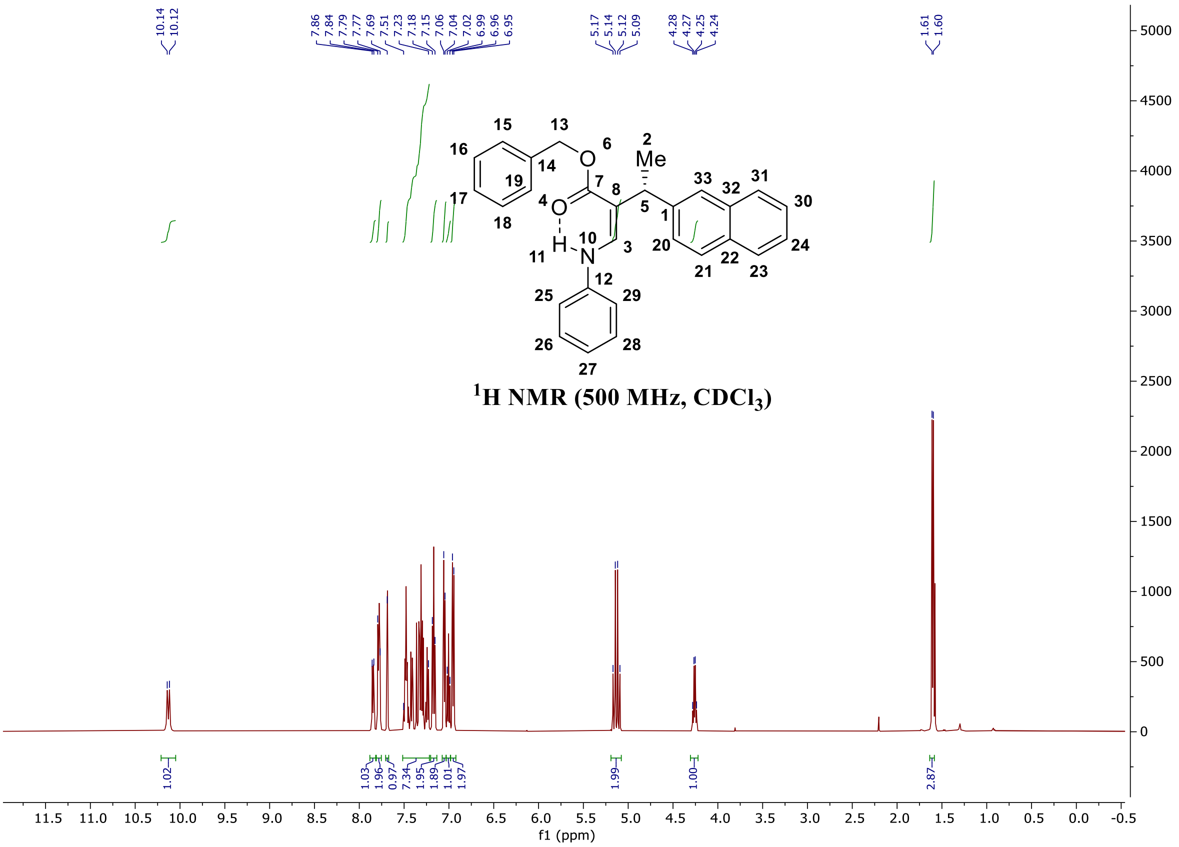

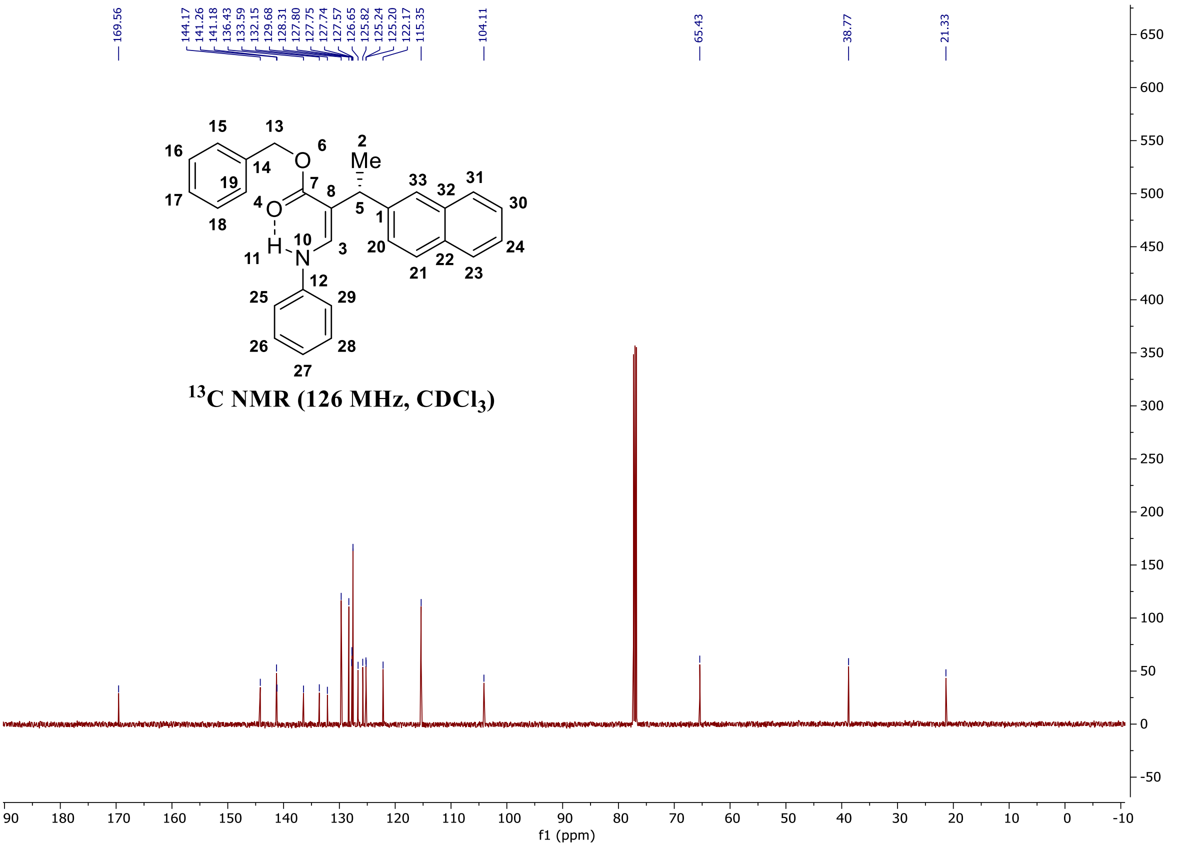

4-Bromobenzyl (S,Z)-2-(((4-methoxyphenyl)amino)methylene)-3-(perfluorophenyl)butanoate (3ln):

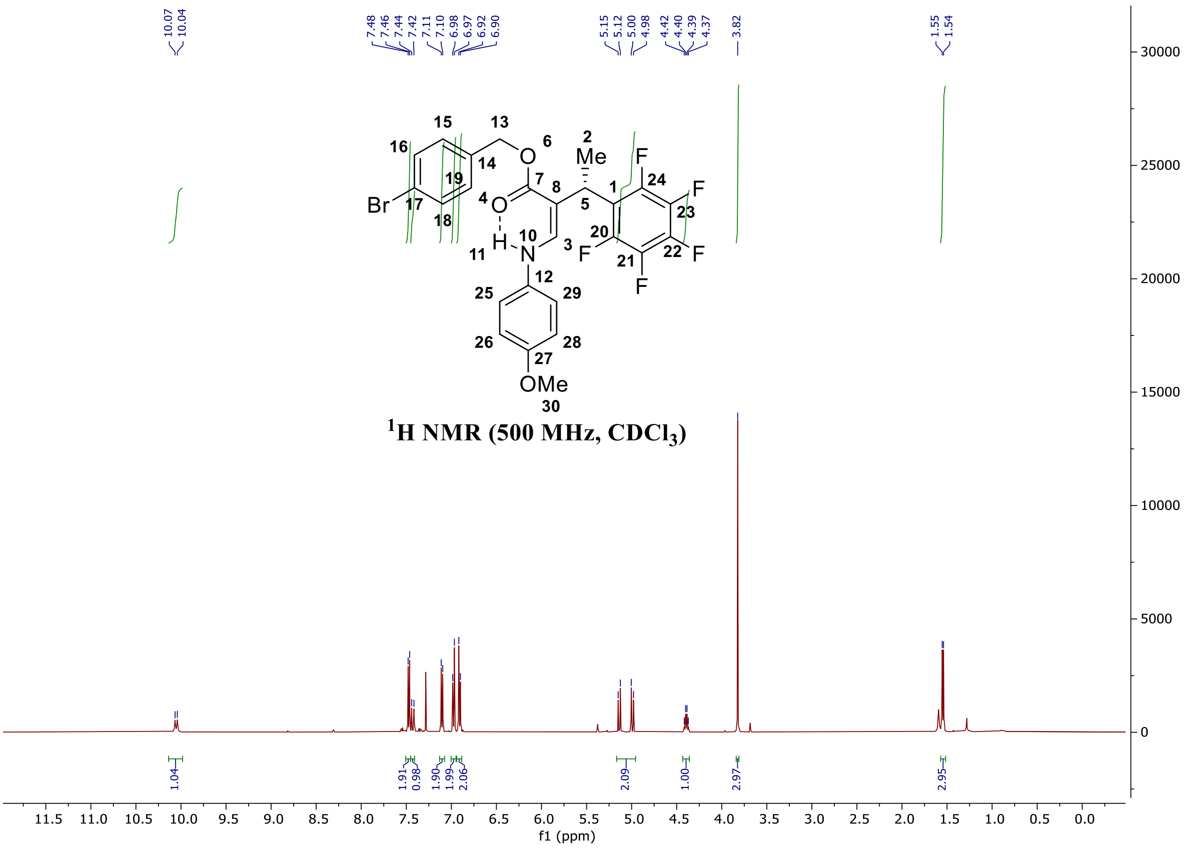

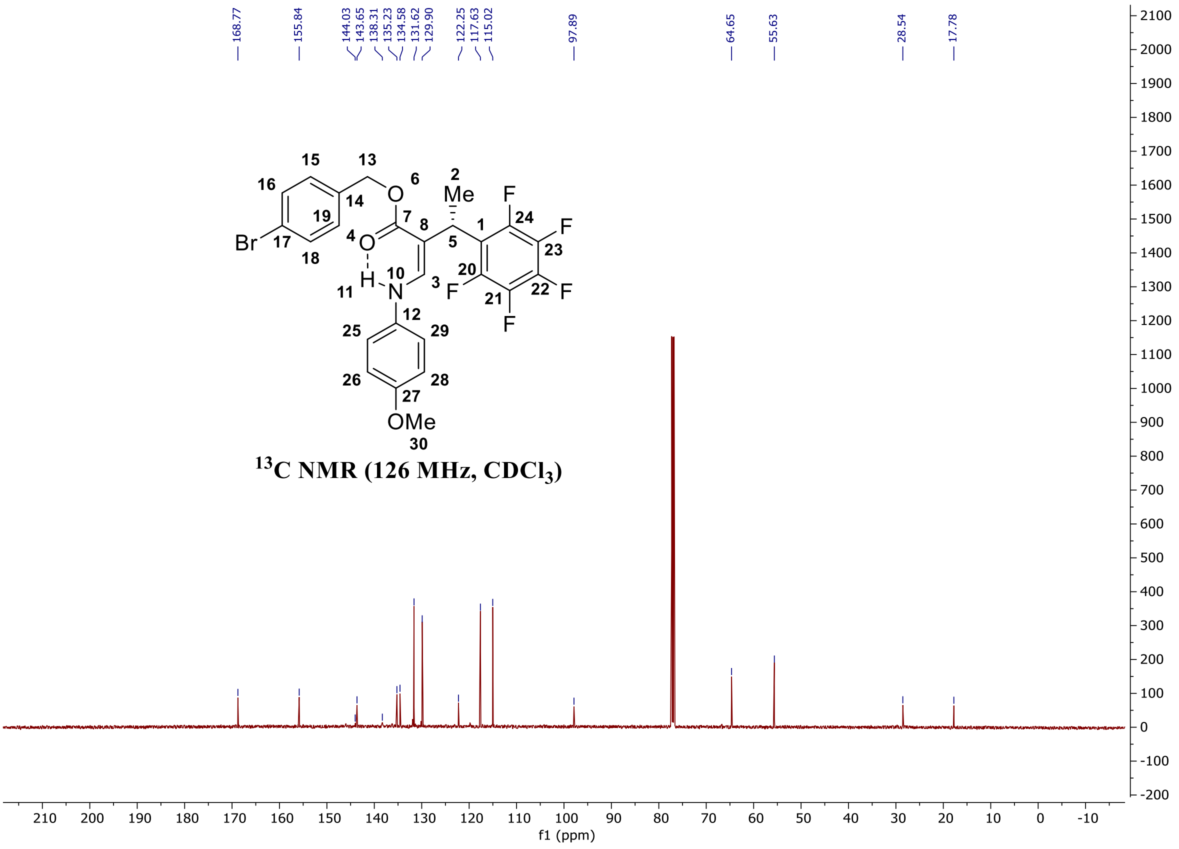

^{13}C NMR (126 MHz, CDCl$_3$)

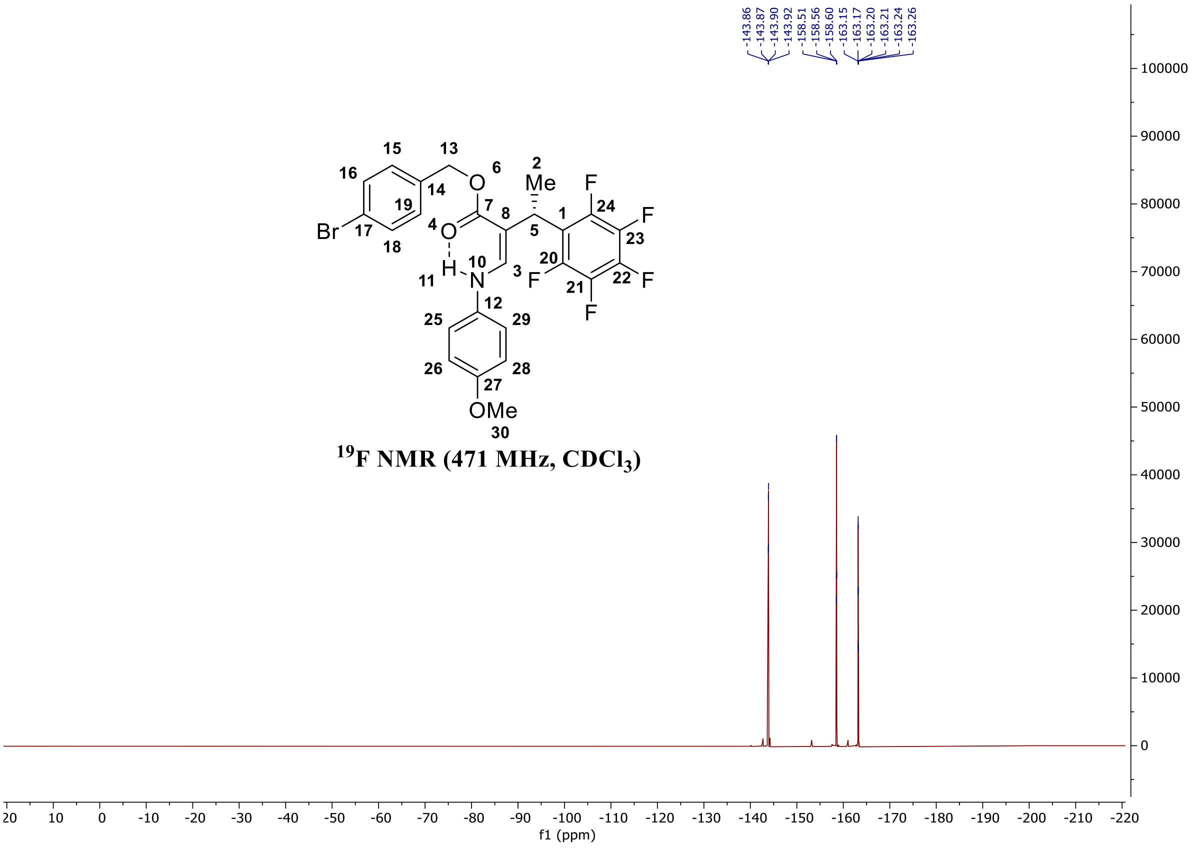

^{19}F NMR (471 MHz, CDCl$_3$)

Benzyl (*R*,*Z*)-3-methyl-2-((phenylamino)methylene)heptanoate (3as):

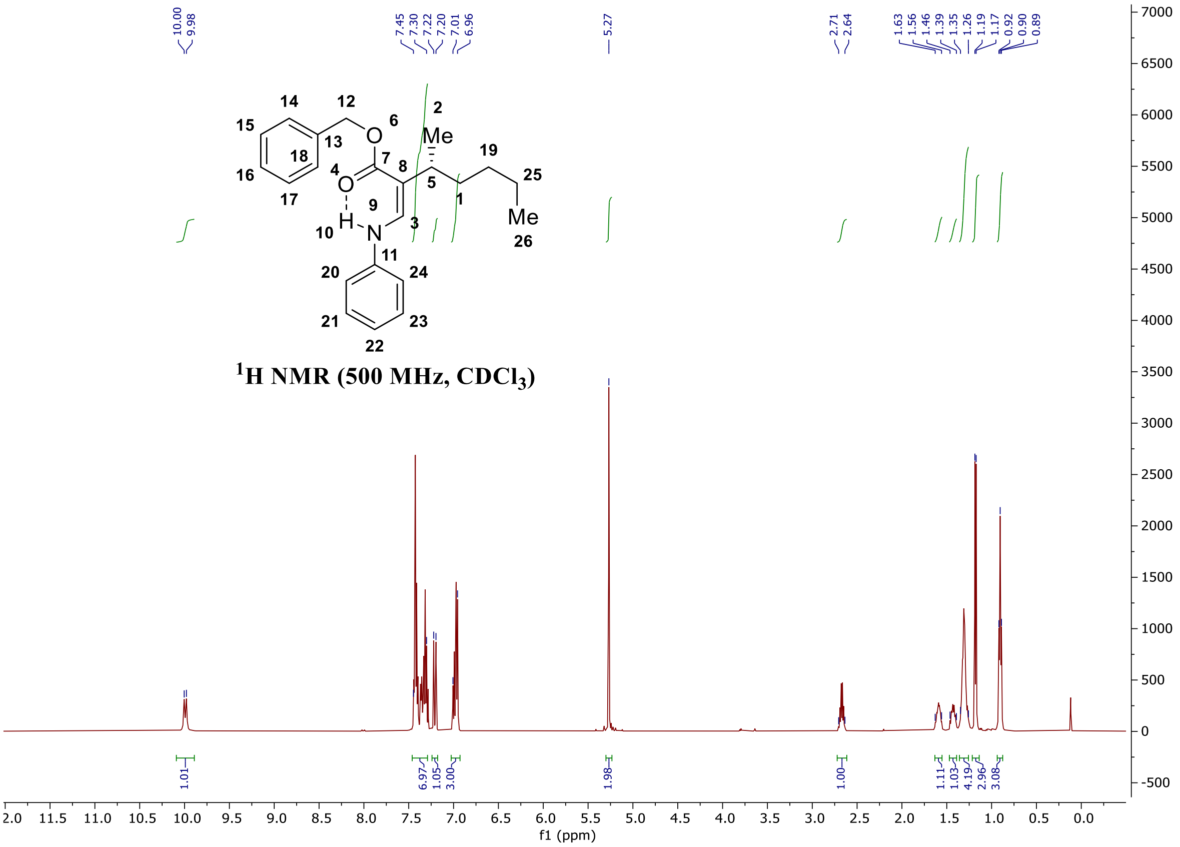

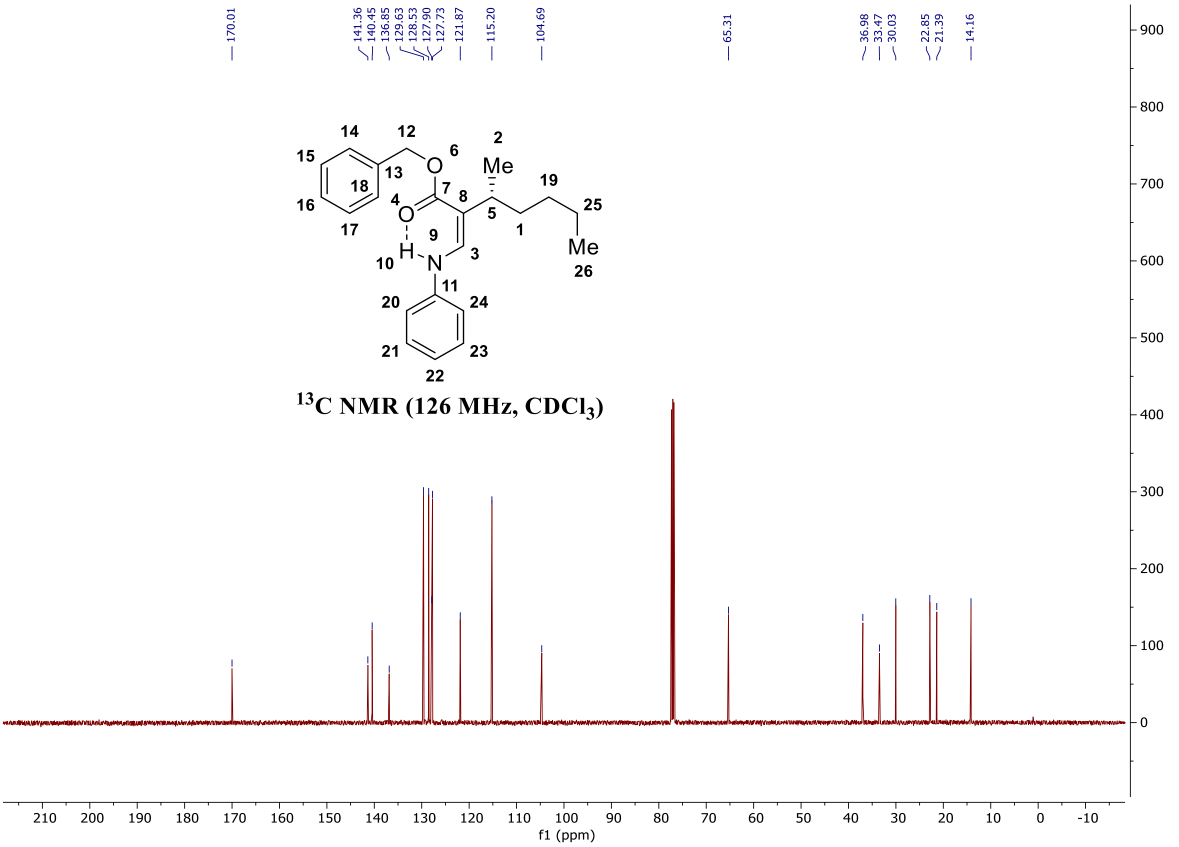

Benzyl (*R*,*Z*)-3-methyl-5-phenyl-2-((phenylamino)methylene)pentanoate (3at):

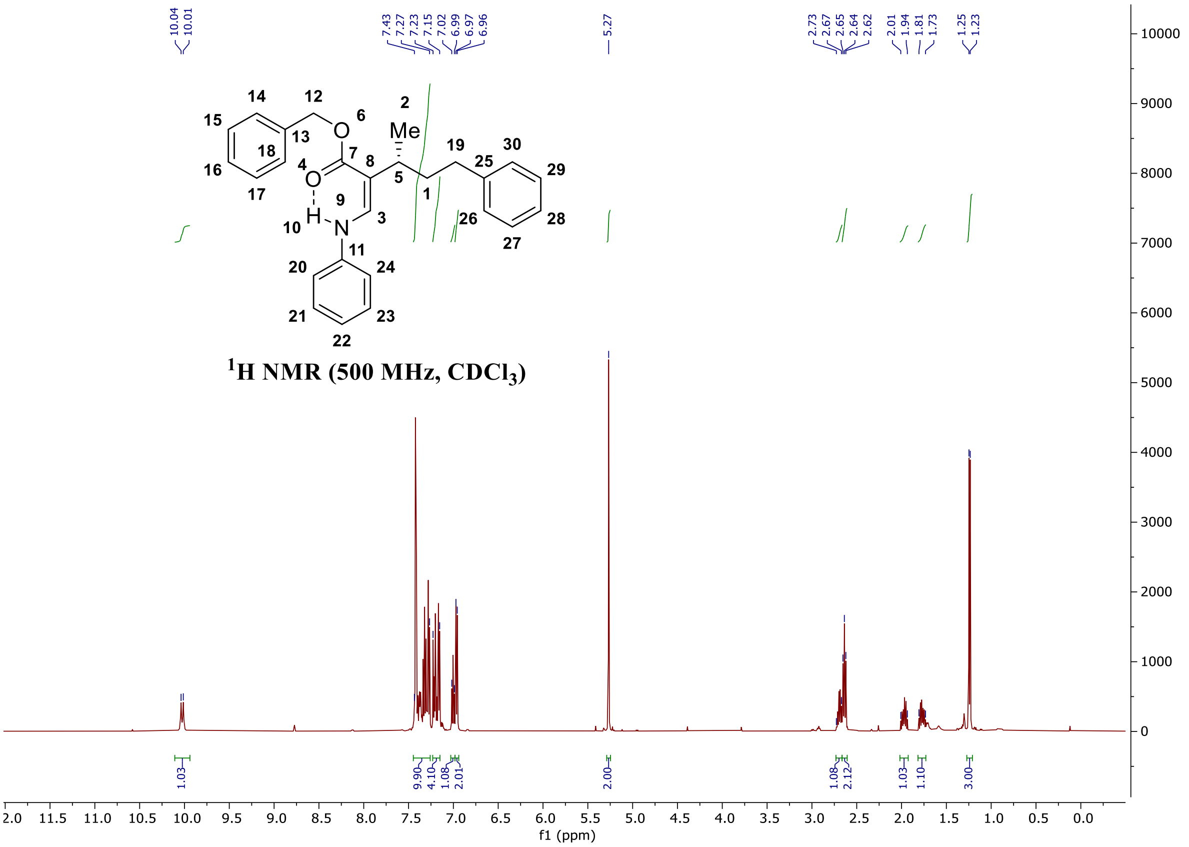

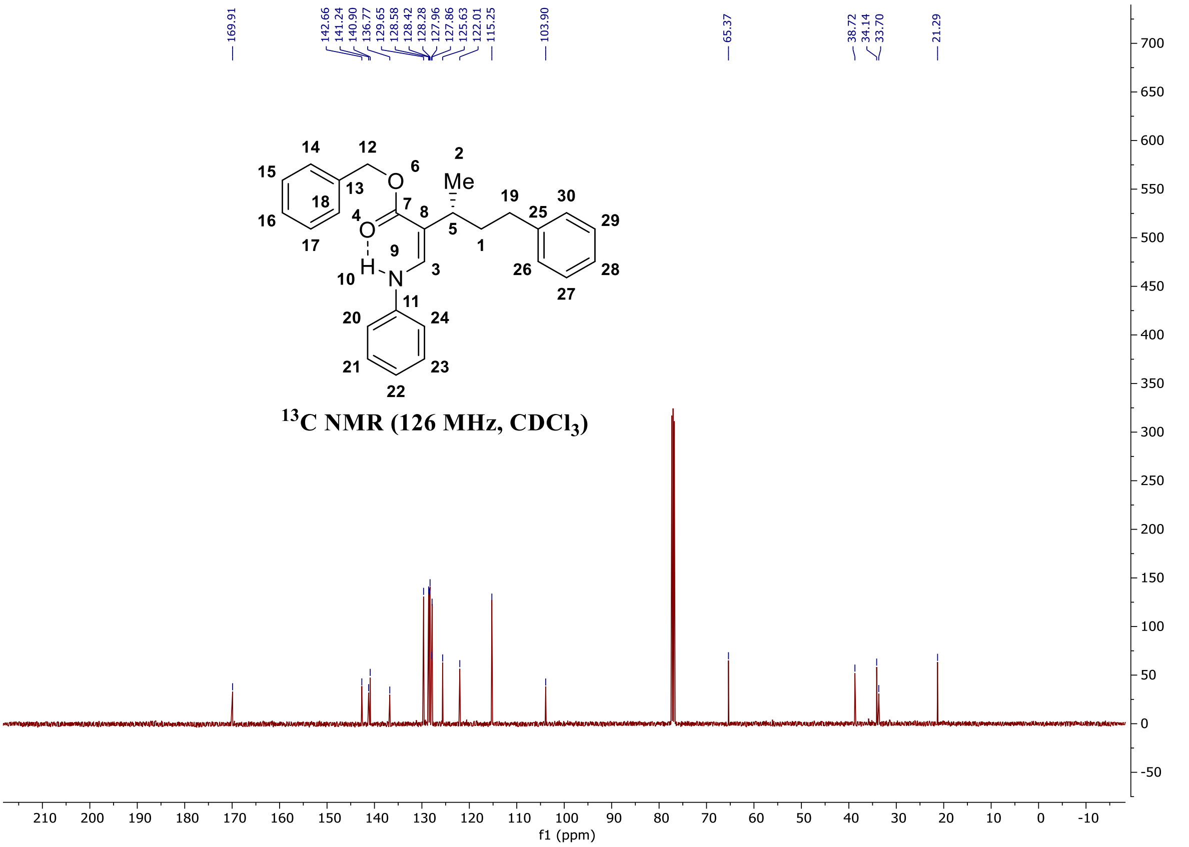

1-Benzyl 7-methyl (*R*,*Z*)-2-(((4-methoxyphenyl)amino)methylene)-3-methylheptanedioate (3hu):

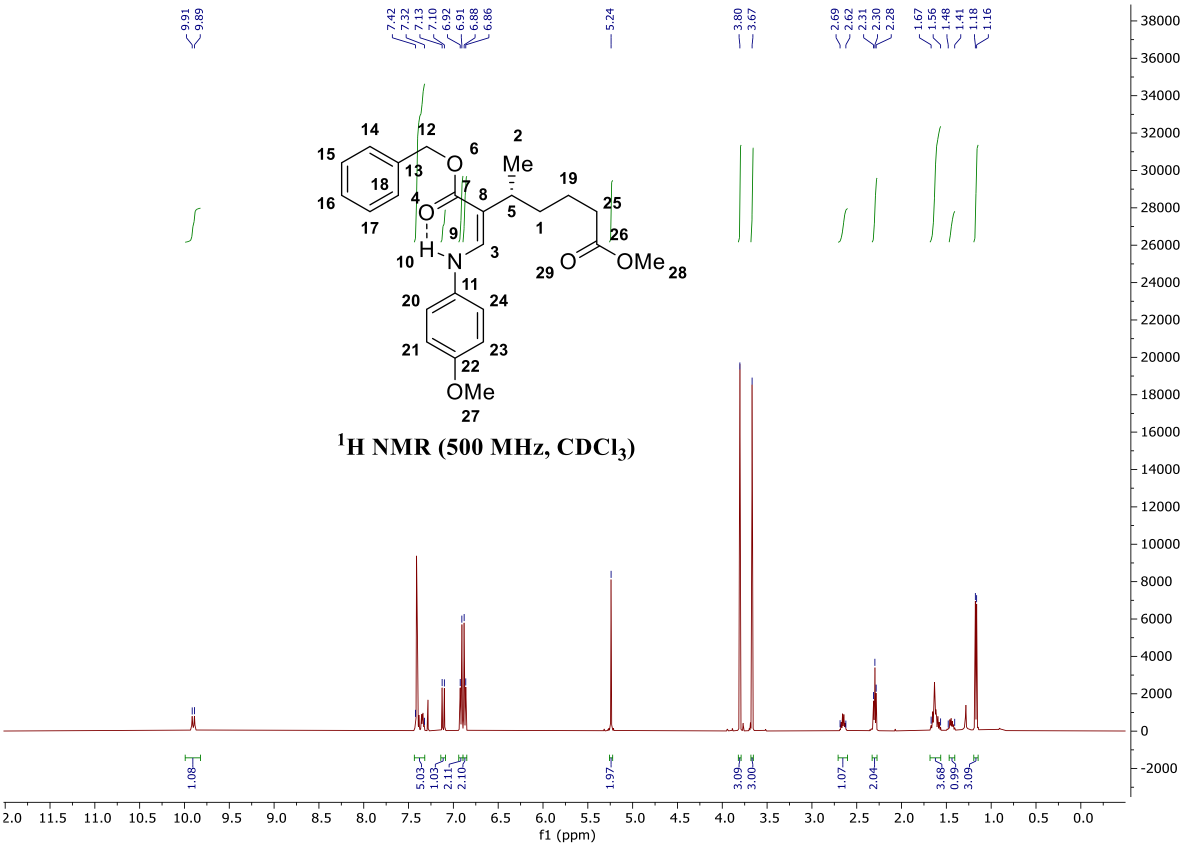

^{1}H NMR (500 MHz, CDCl$_3$)

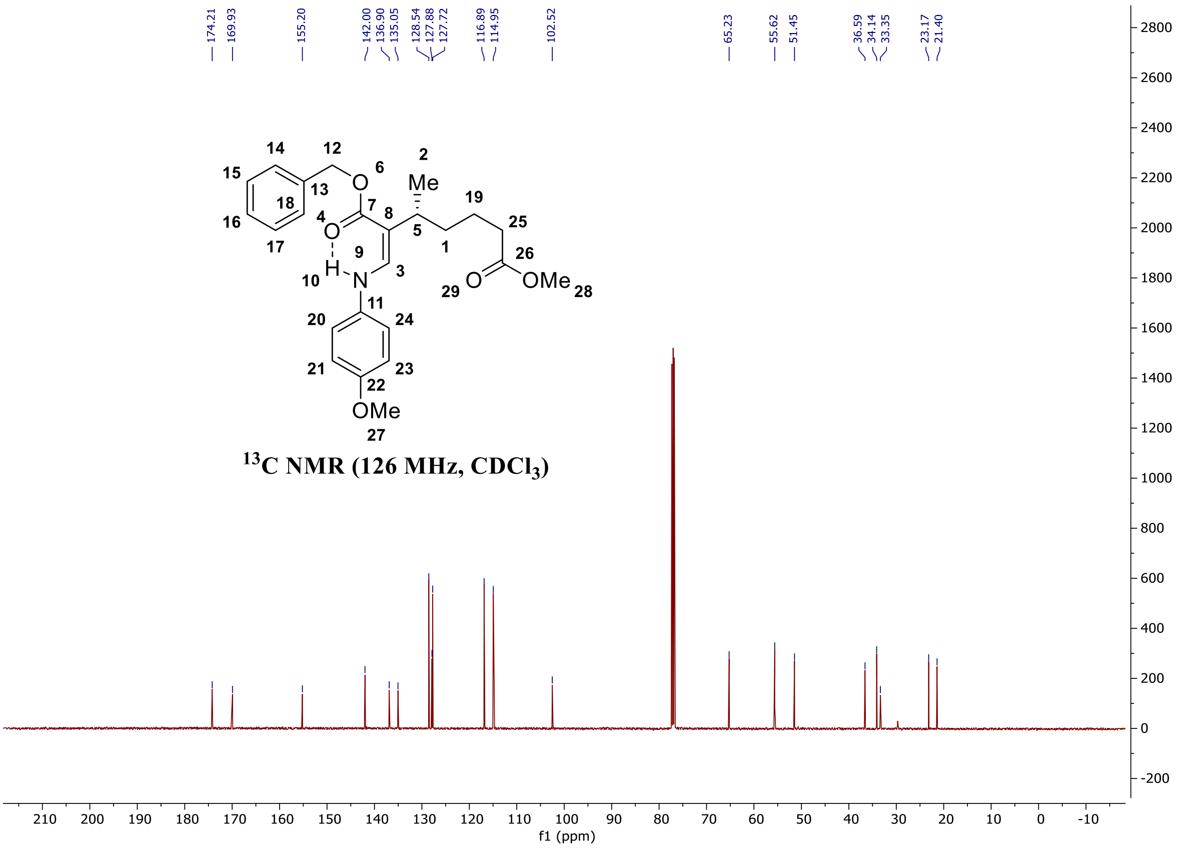

^{13}C NMR (126 MHz, CDCl$_3$)

Benzyl (*R*,*Z*)-5-acetoxy-3-methyl-2-((phenylamino)methylene)pentanoate (3av):

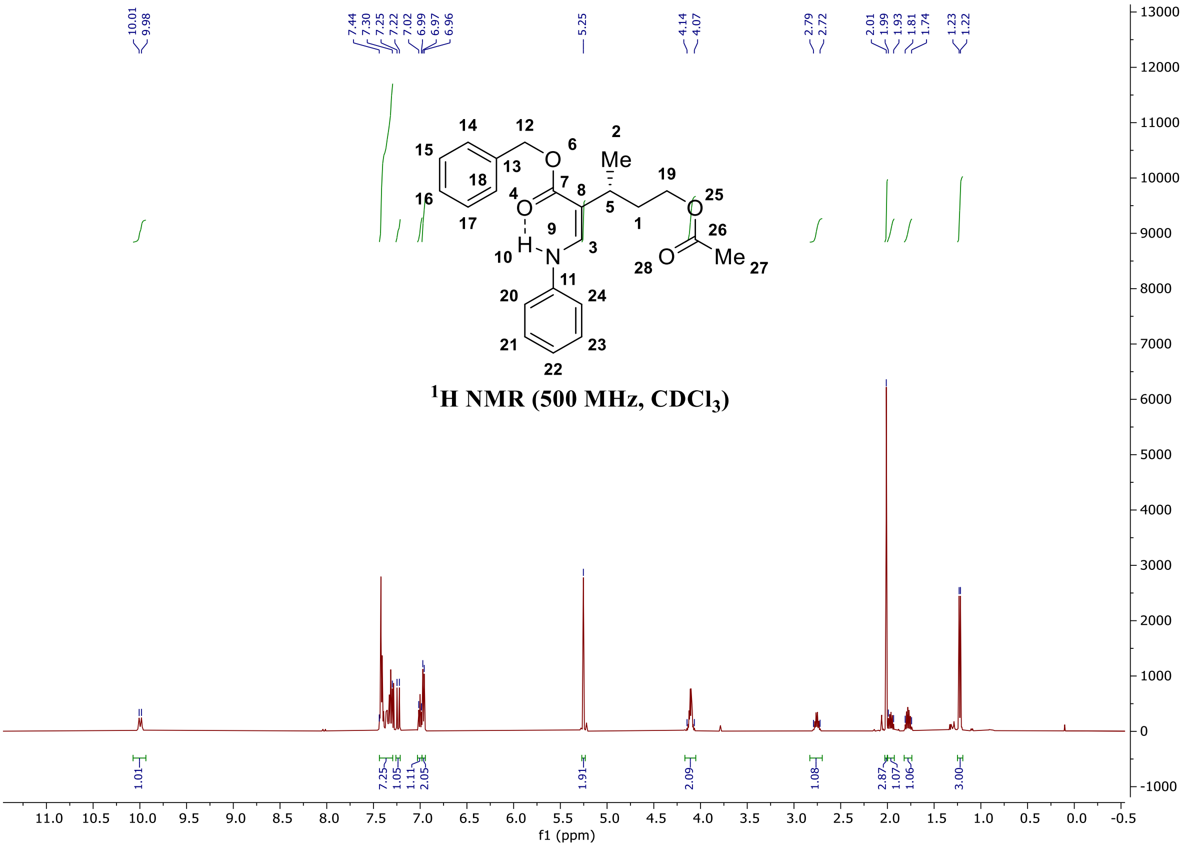

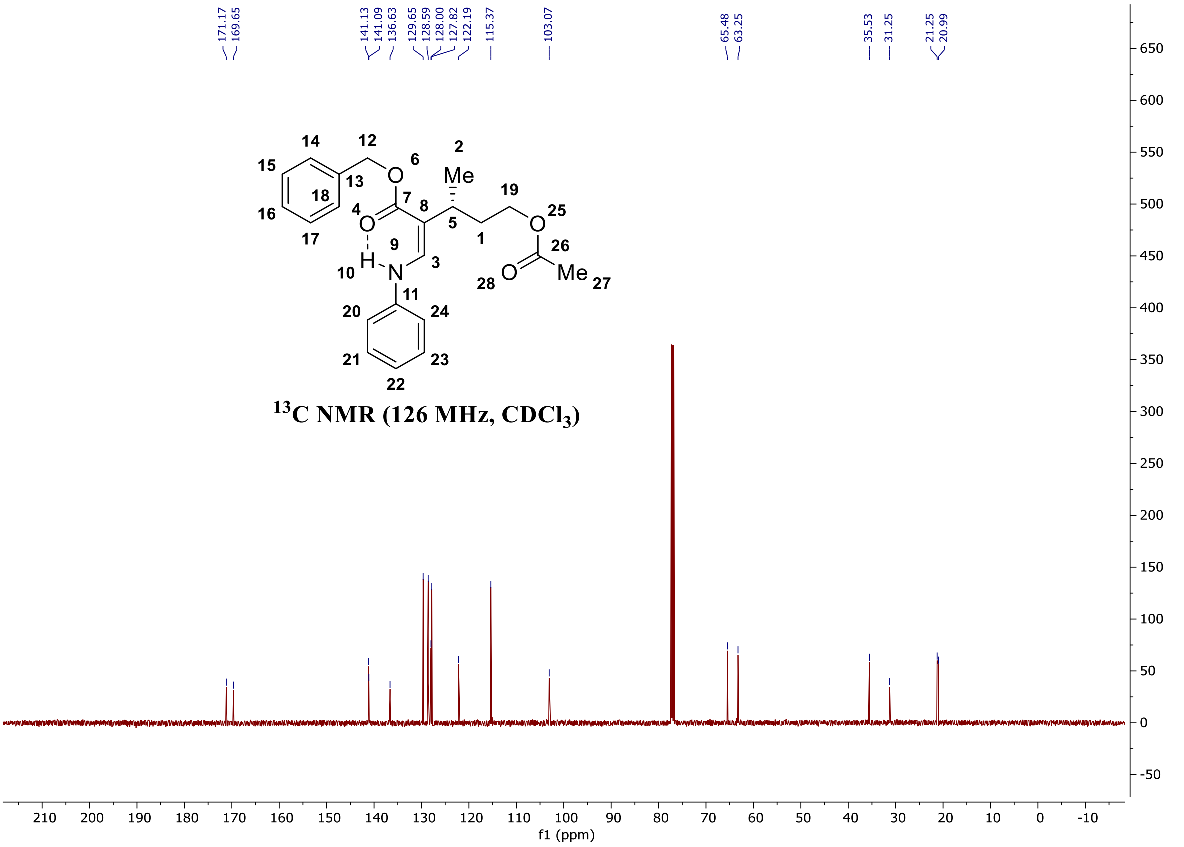

Benzyl (*R*,*Z*)-7-((*tert*-butyldimethylsilyl)oxy)-3-methyl-2-((phenylamino)methylene)heptanoate (3aw):

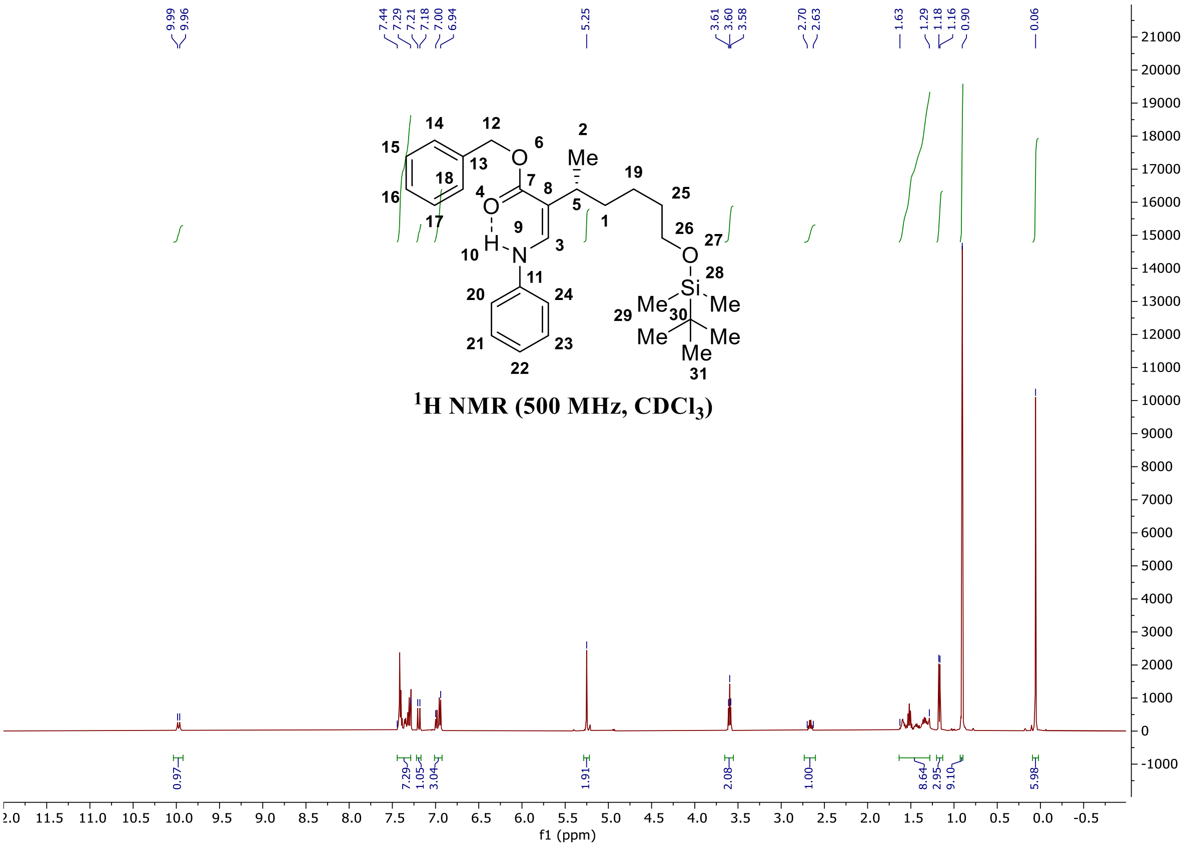

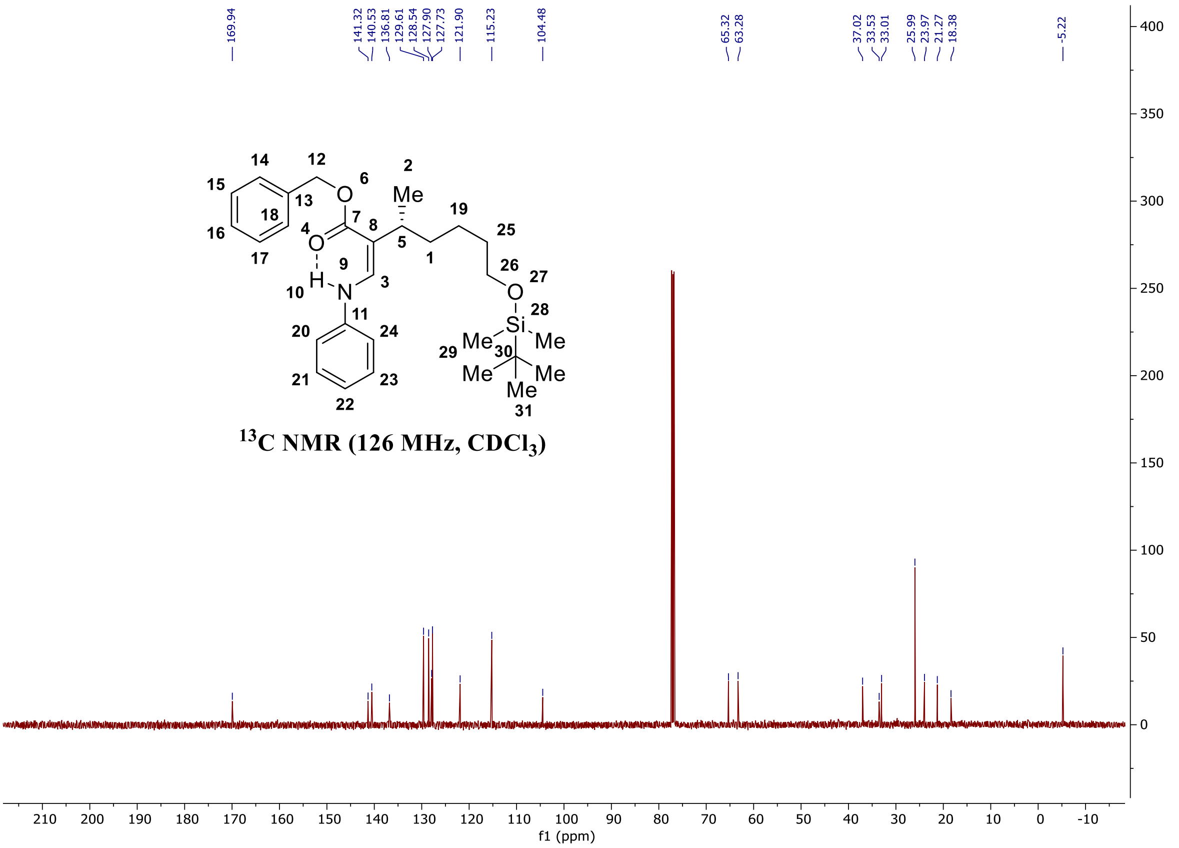

Benzyl (*R*,*Z*)-3-(4-((((*S*)-2-(6-methoxynaphthalen-2-yl)propanoyl)oxy)methyl)phenyl)-2-((phenylamino)methylene)butanoate (3ax):

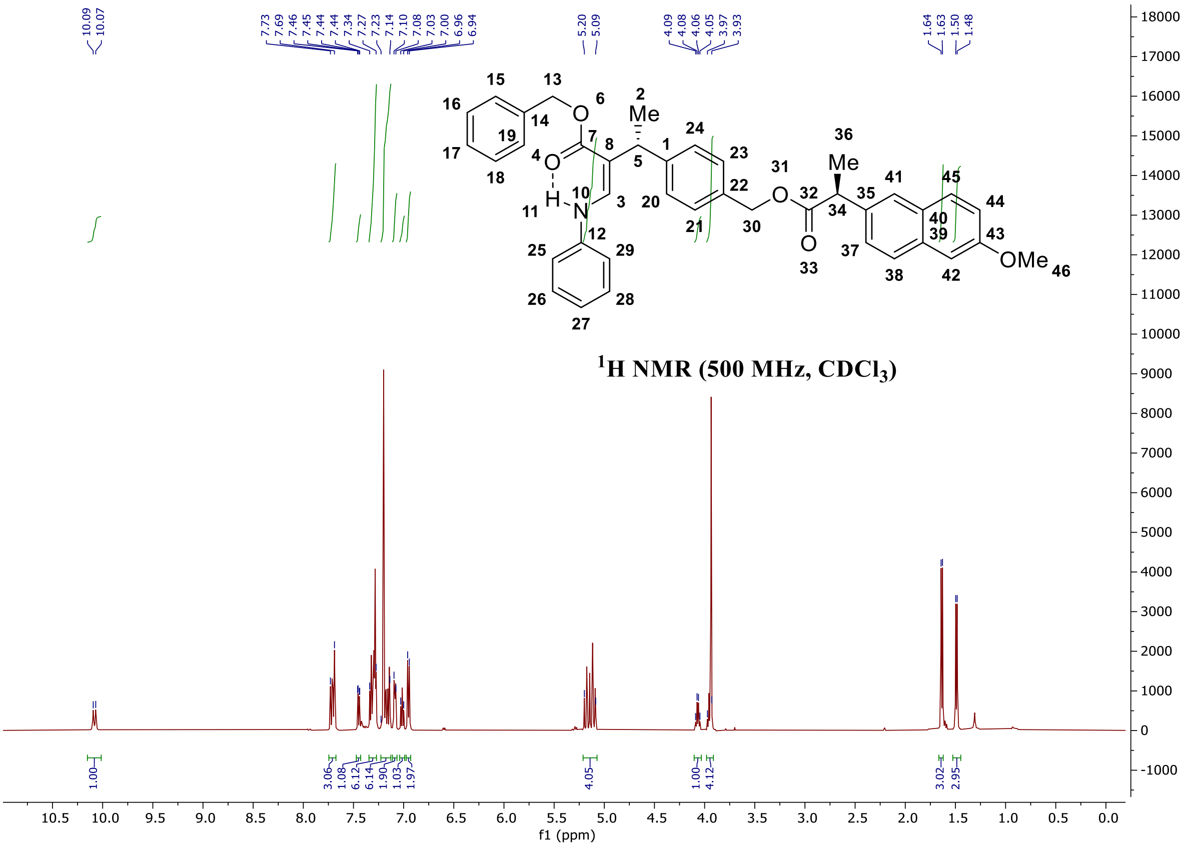

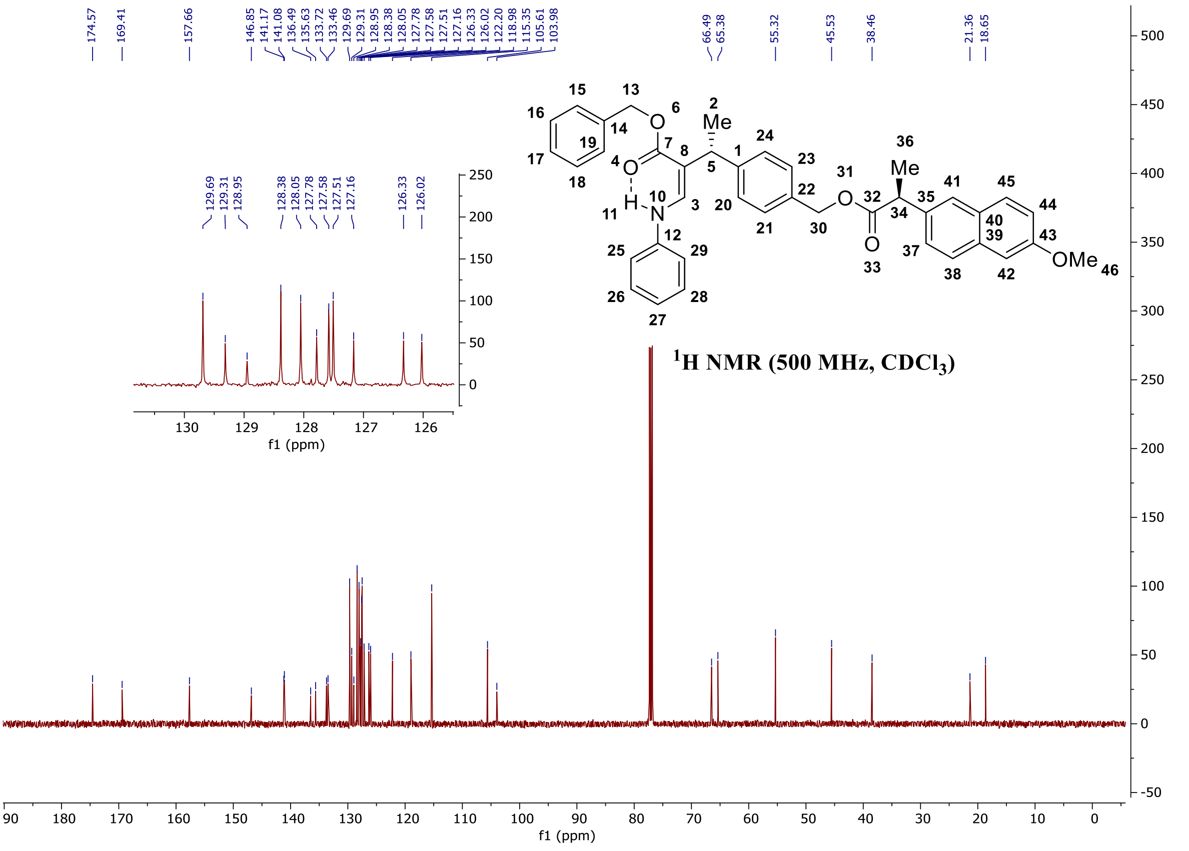

1-Benzyl 6-((3*S*,8*S*,9*S*,10*R*,13*R*,14*S*,17*R*)-10,13-dimethyl-17-((*R*)-6-methylheptan-2-yl)-2,3,4,7,8,9,10,11,12,13,14,15,16,17-tetradecahydro-1*H*-cyclopenta[*a*]phenanthren-3-yl) (*R*,*Z*)-3-methyl-2-((phenylamino)methylene)hexanedioate (3ay):

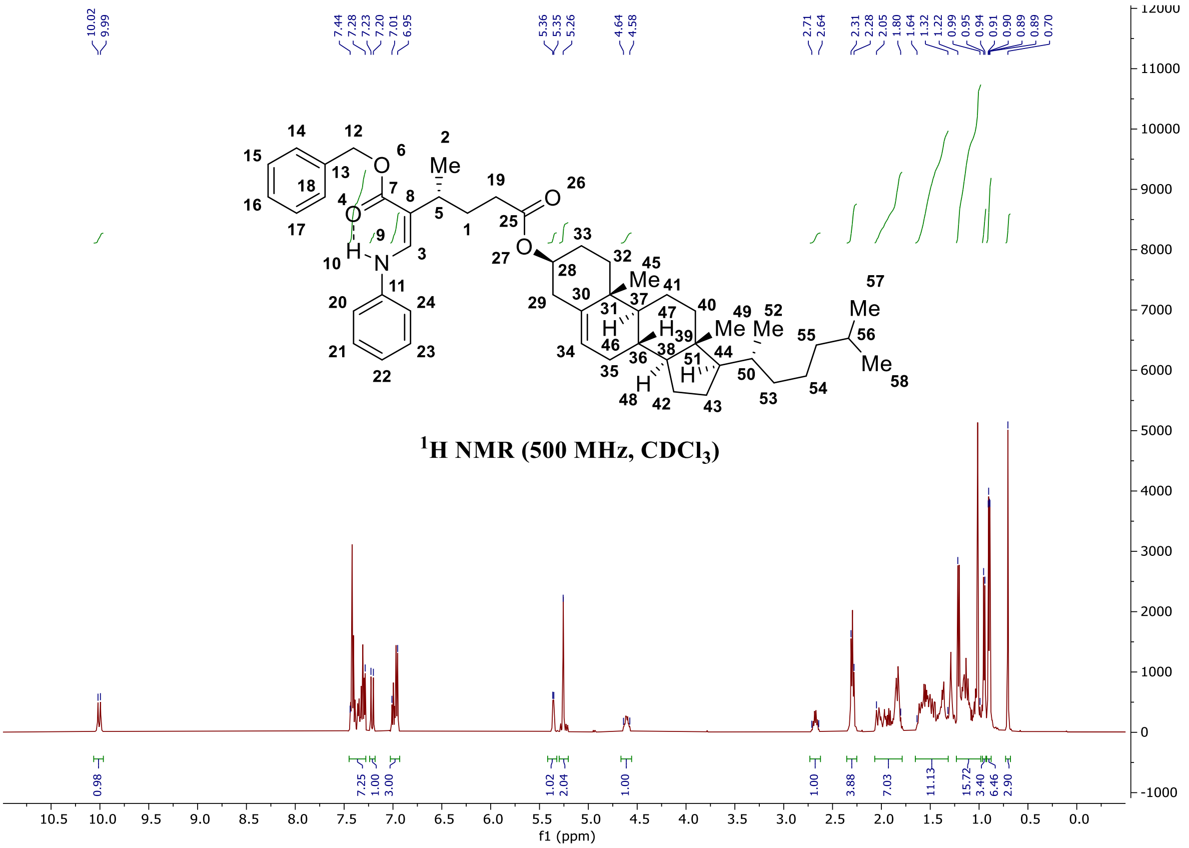

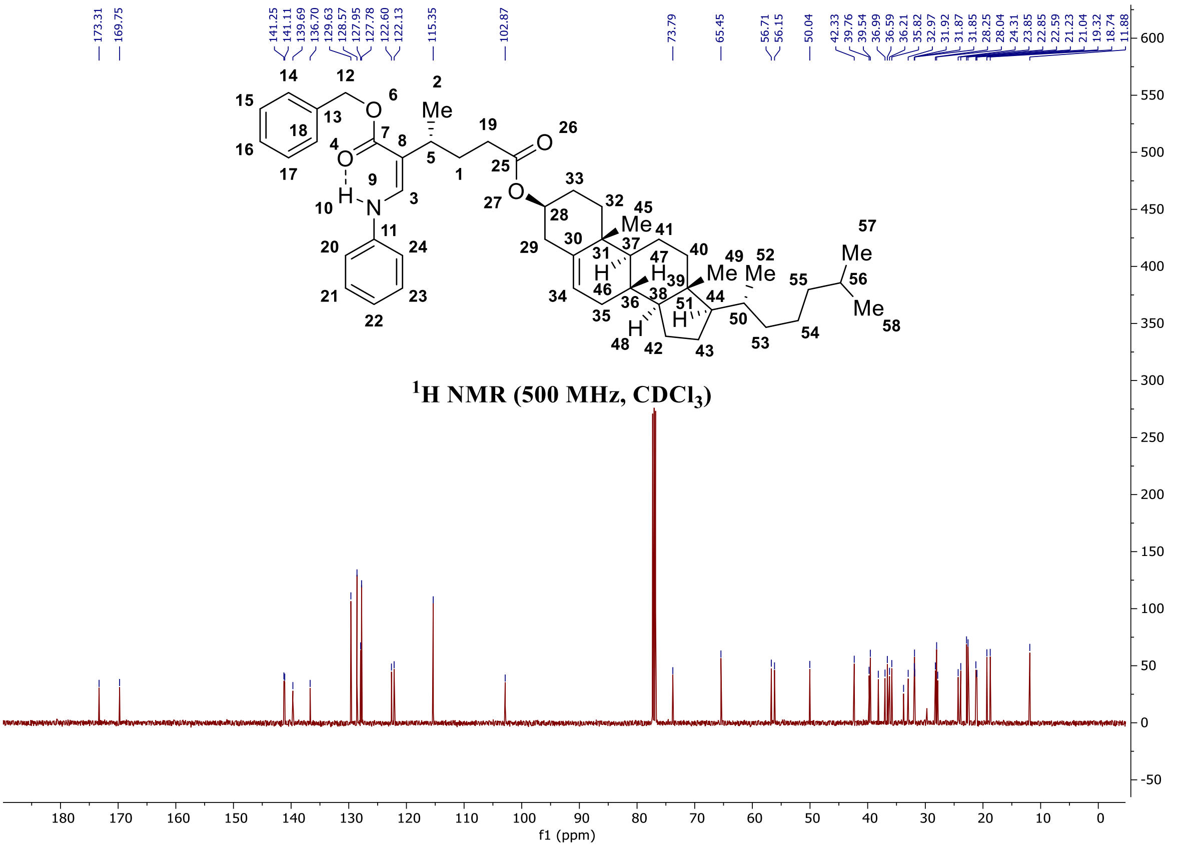

Benzyl (*R*,*Z*)-3-((8*R*,9*S*,13*S*,14*S*)-13-methyl-17-*oxo*-7,8,9,11,12,13,14,15,16,17-decahydro-6*H*-cyclopenta[*a*]phenanthren-3-yl)-2-((phenylamino)methylene)butanoate (3az):

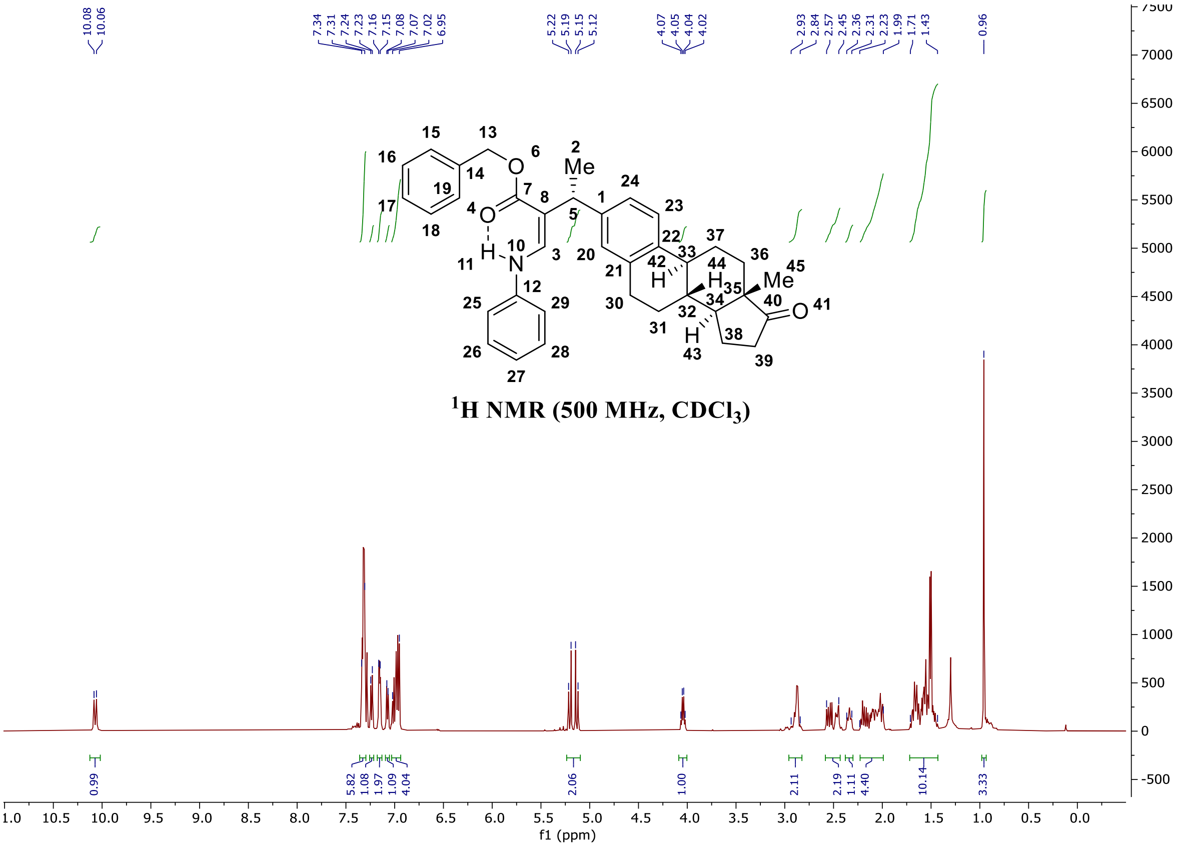

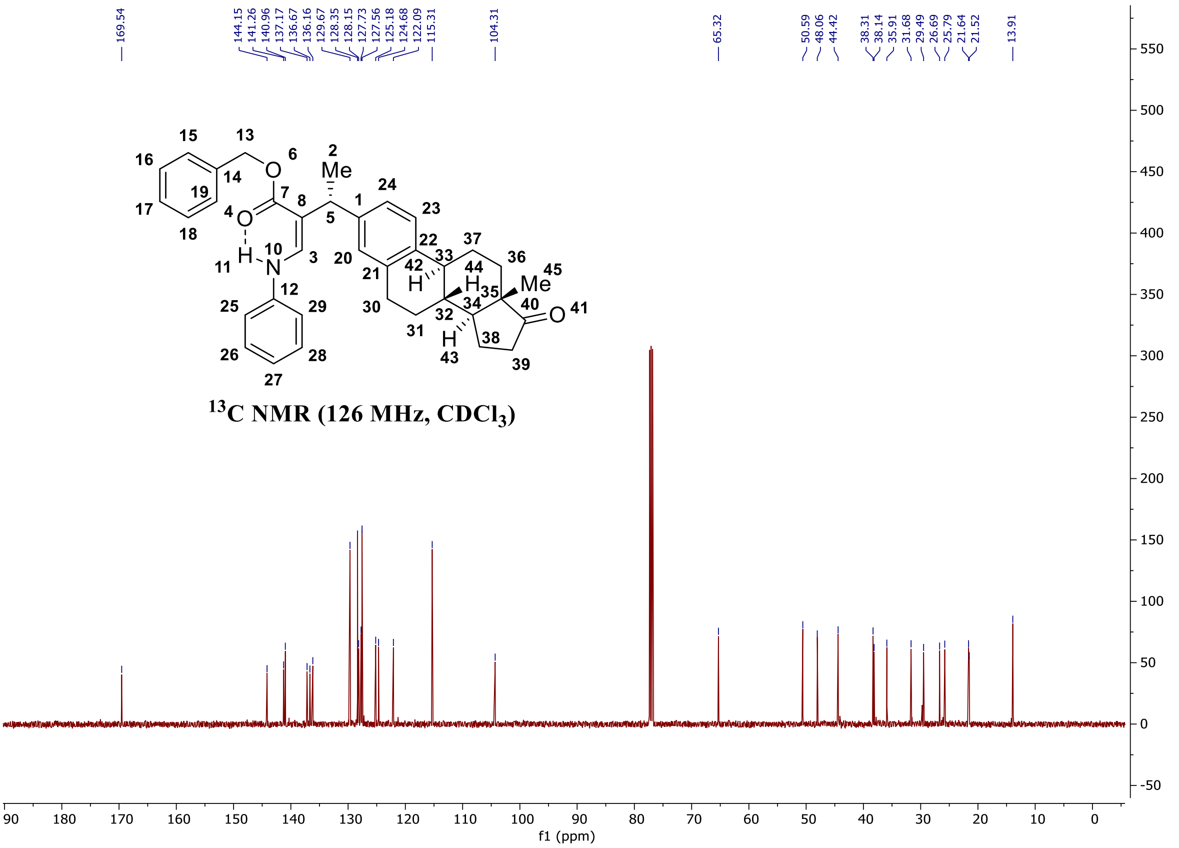

Benzyl (*R*,*Z*)-3-(4-((2-(1-(4-chlorobenzoyl)-5-methoxy-2-methyl-1*H*-indol-3-yl)acetoxy)methyl)phenyl)-2-((phenylamino)methylene)butanoate (3aaa):

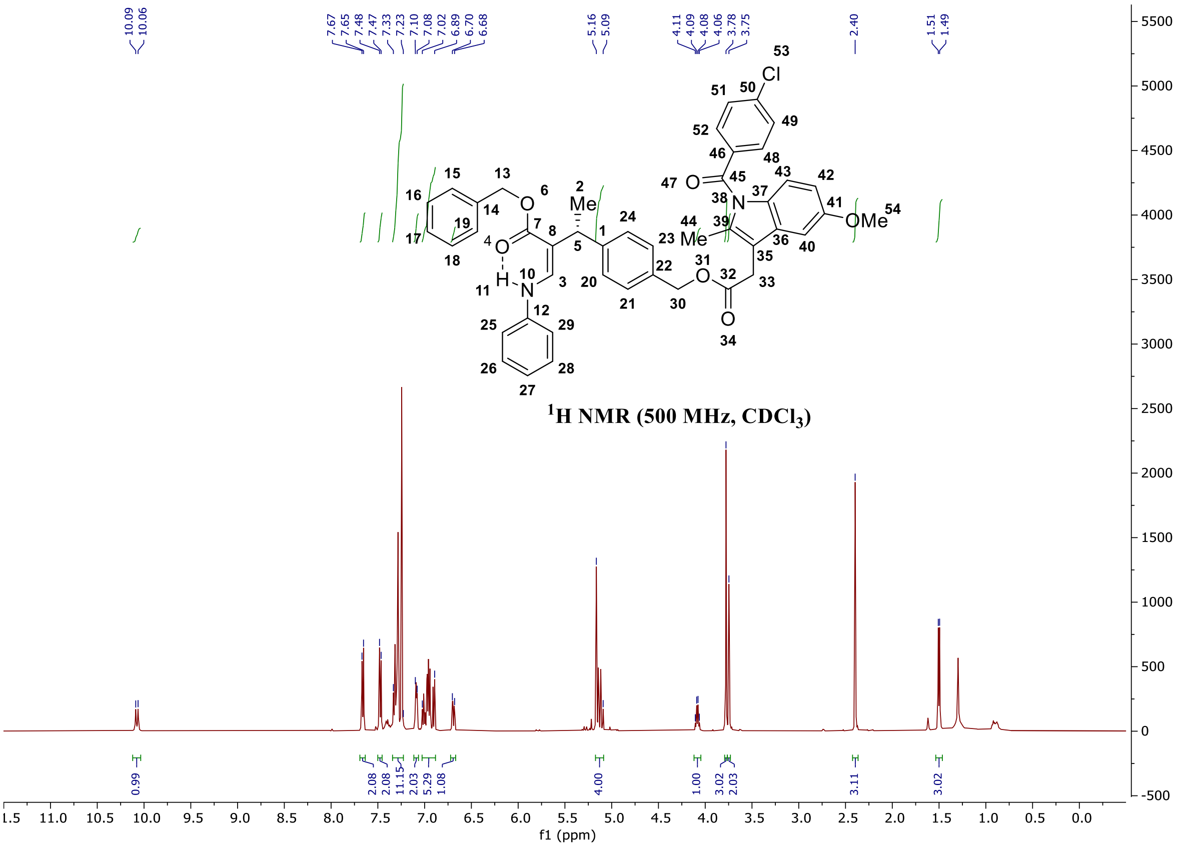

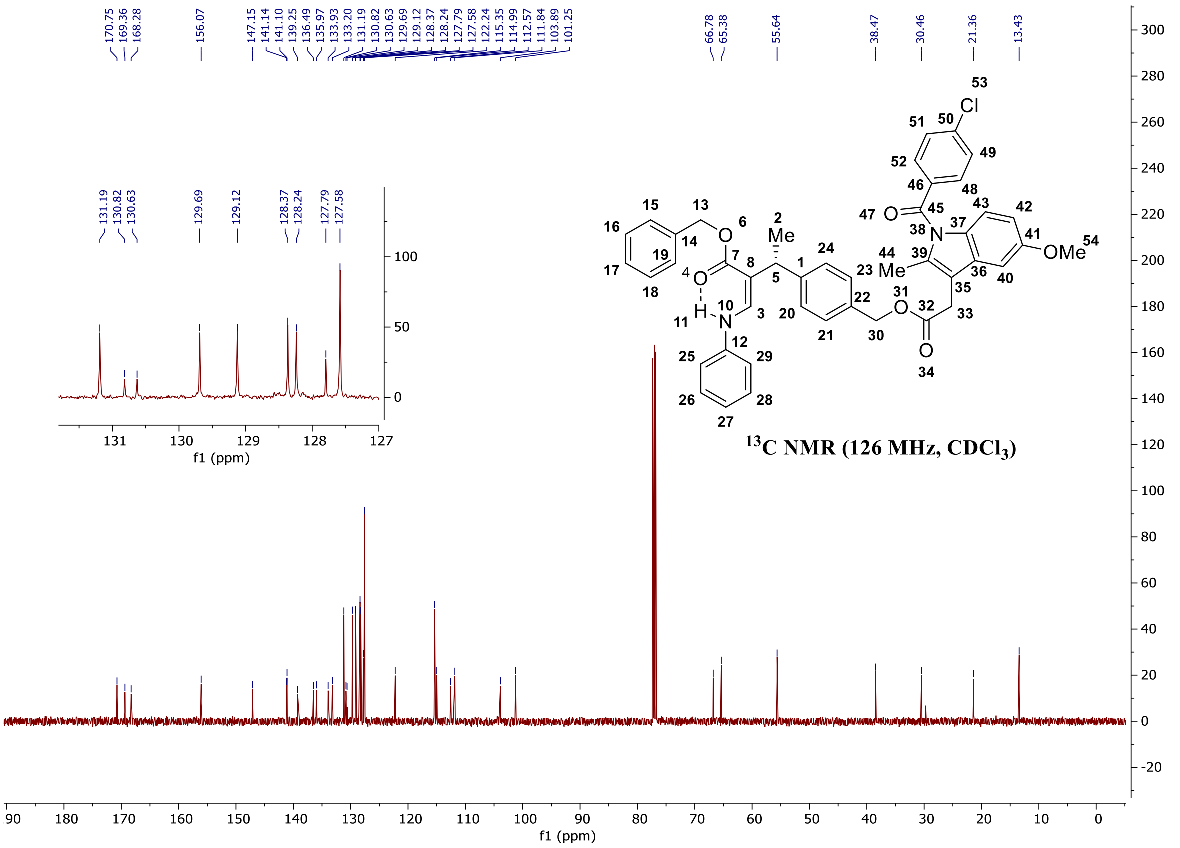

Benzyl (2*S*,3*S*)-2-(((4-methoxyphenyl)amino)methyl)-3-phenylbutanoate (4)

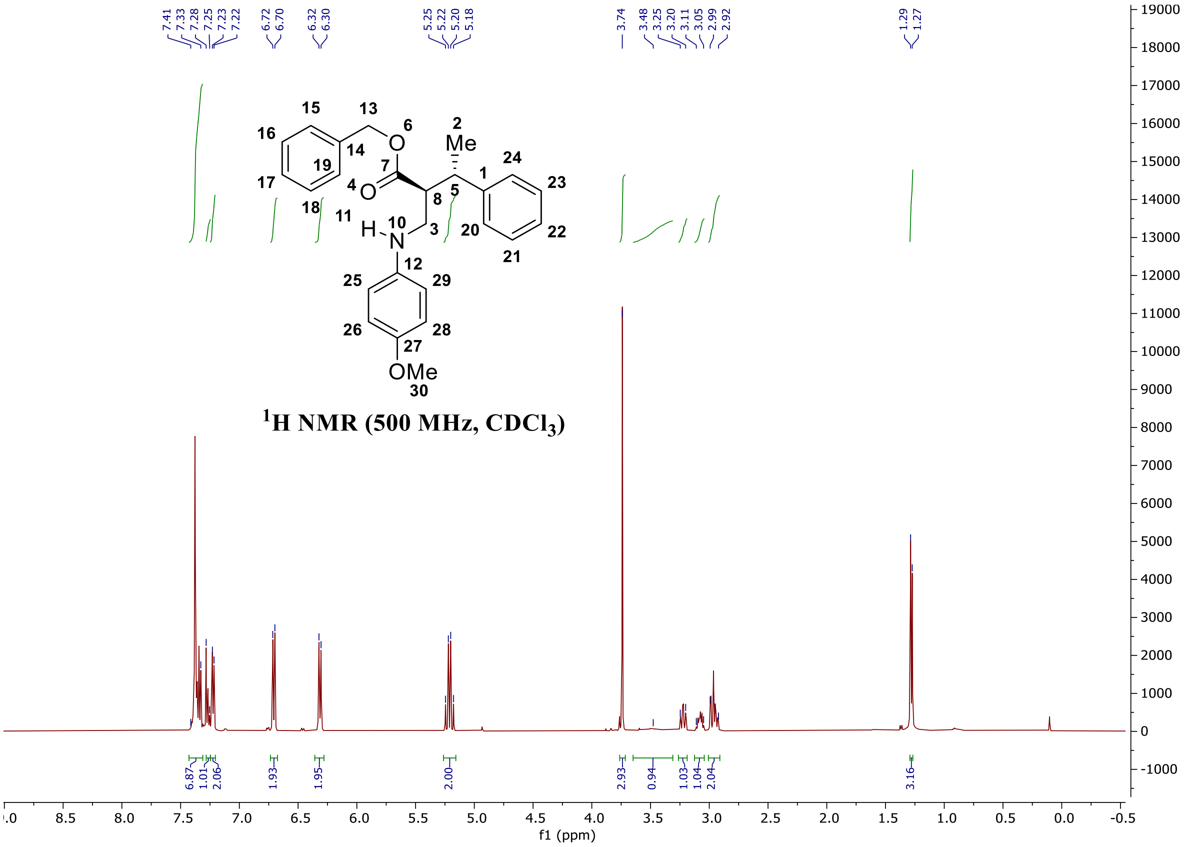

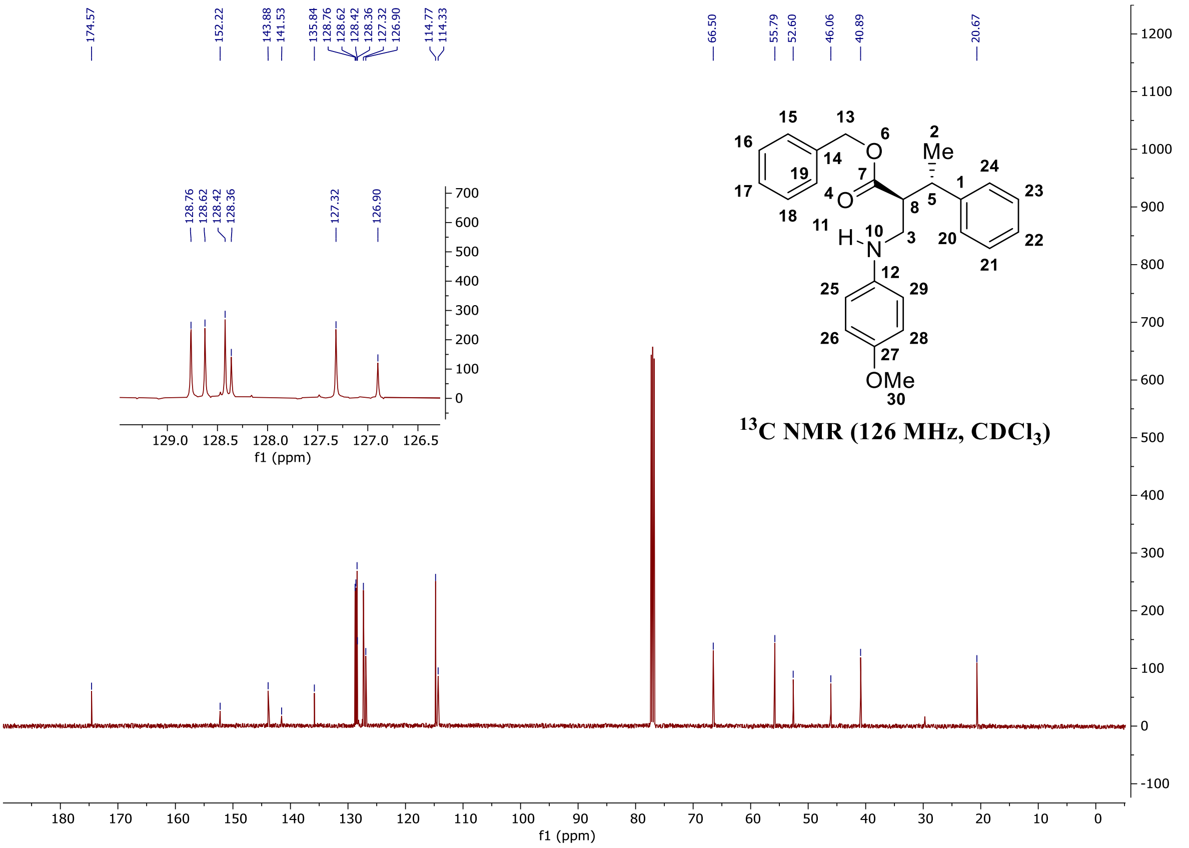

Benzyl (2*S*,3*S*)-2-(((*N*-(4-methoxyphenyl)-4-methylphenyl)sulfonamido)methyl)-3-phenylbutanoate (5):

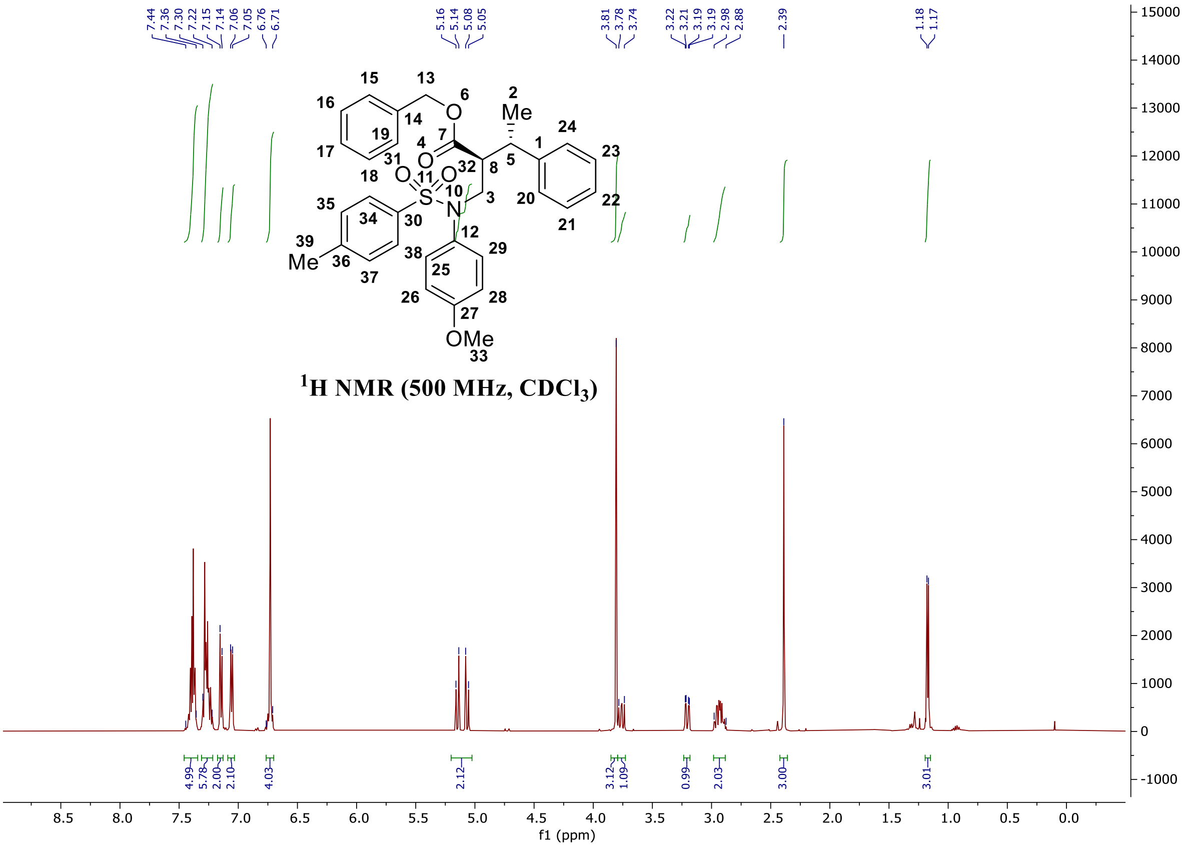

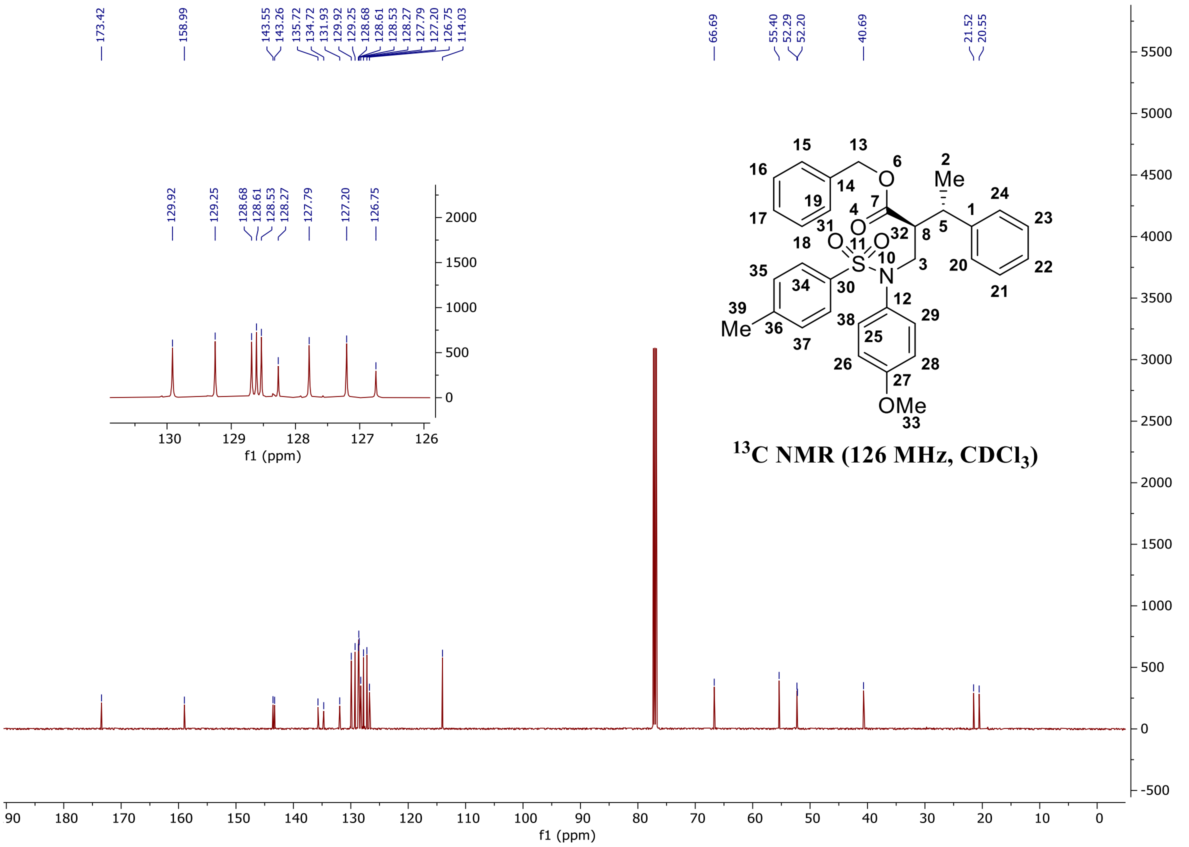

Benzyl (*2S,3S*)-3-(2-chlorophenyl)-2-((phenylamino)methyl)butanoate (6):

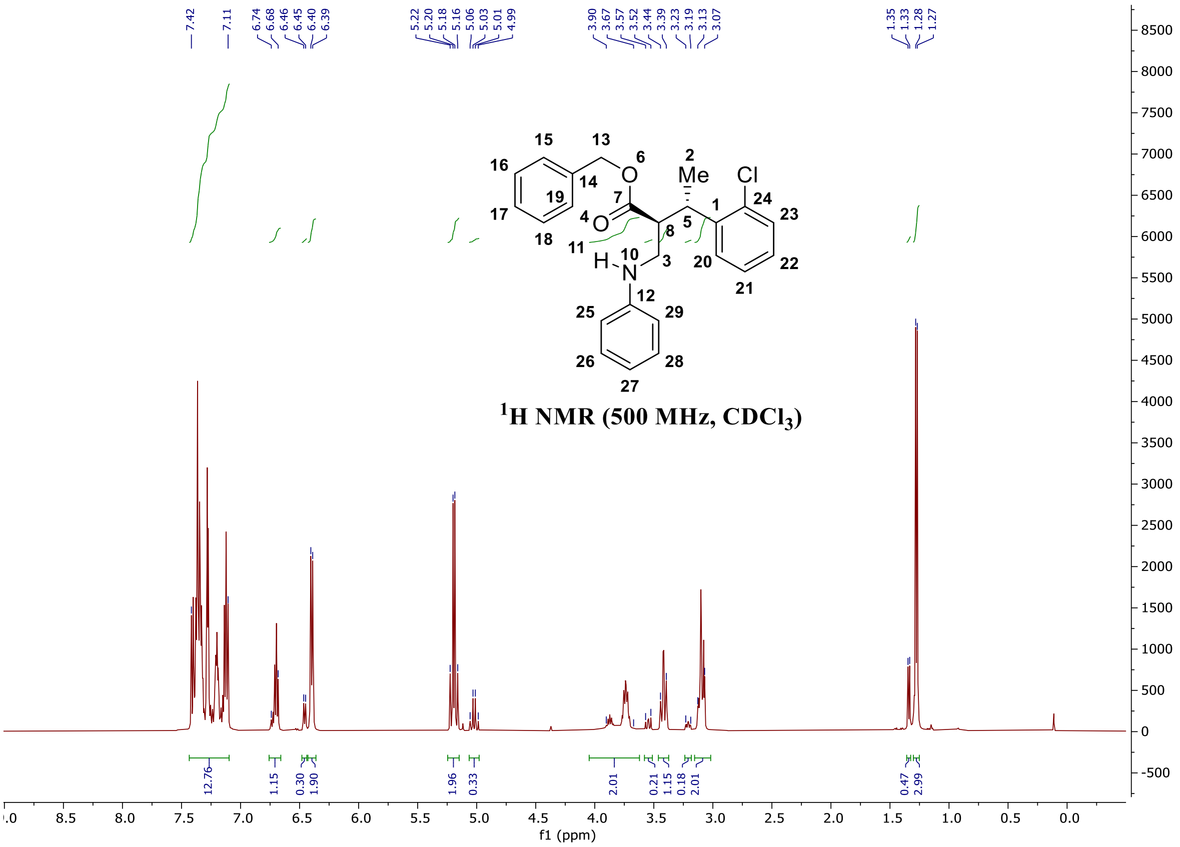

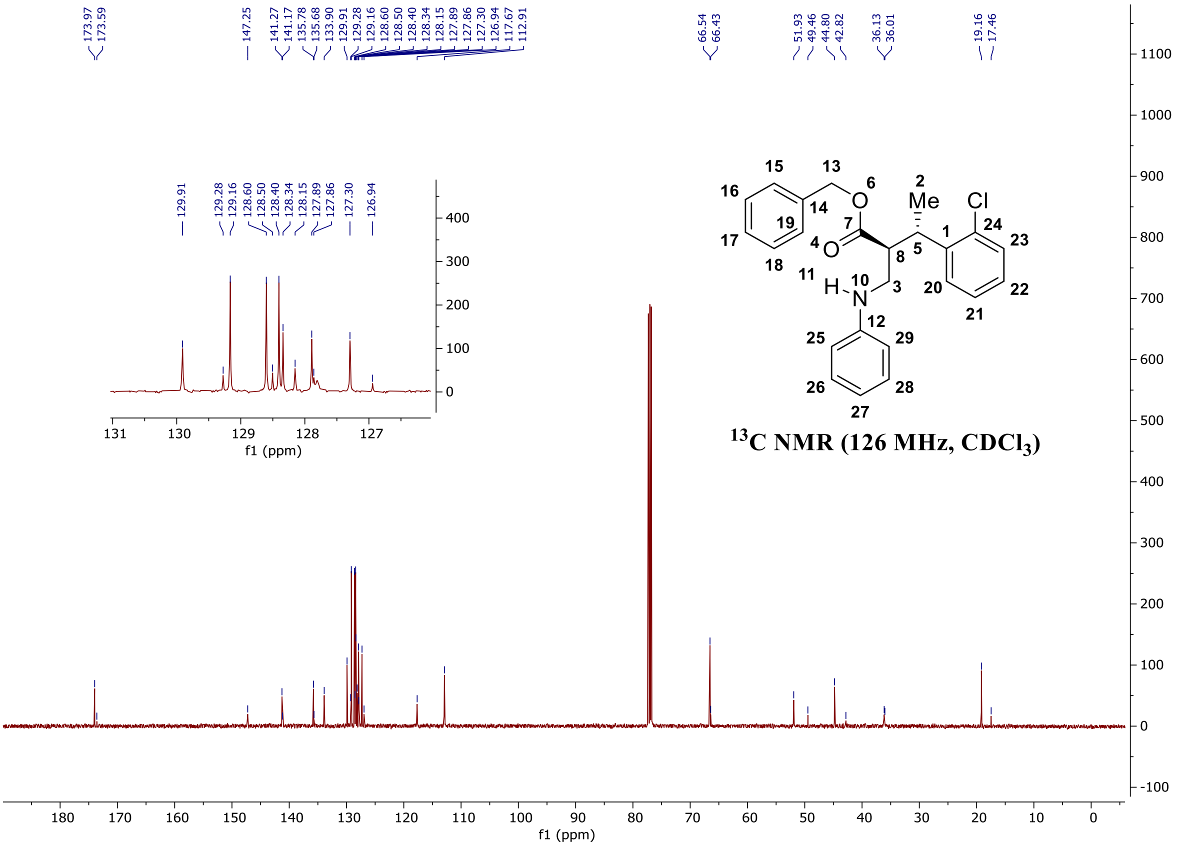

Benzyl (*3S,4S*)-4-methyl-1-phenyl-1,2,3,4-tetrahydroquinoline-3-carboxylate (7):

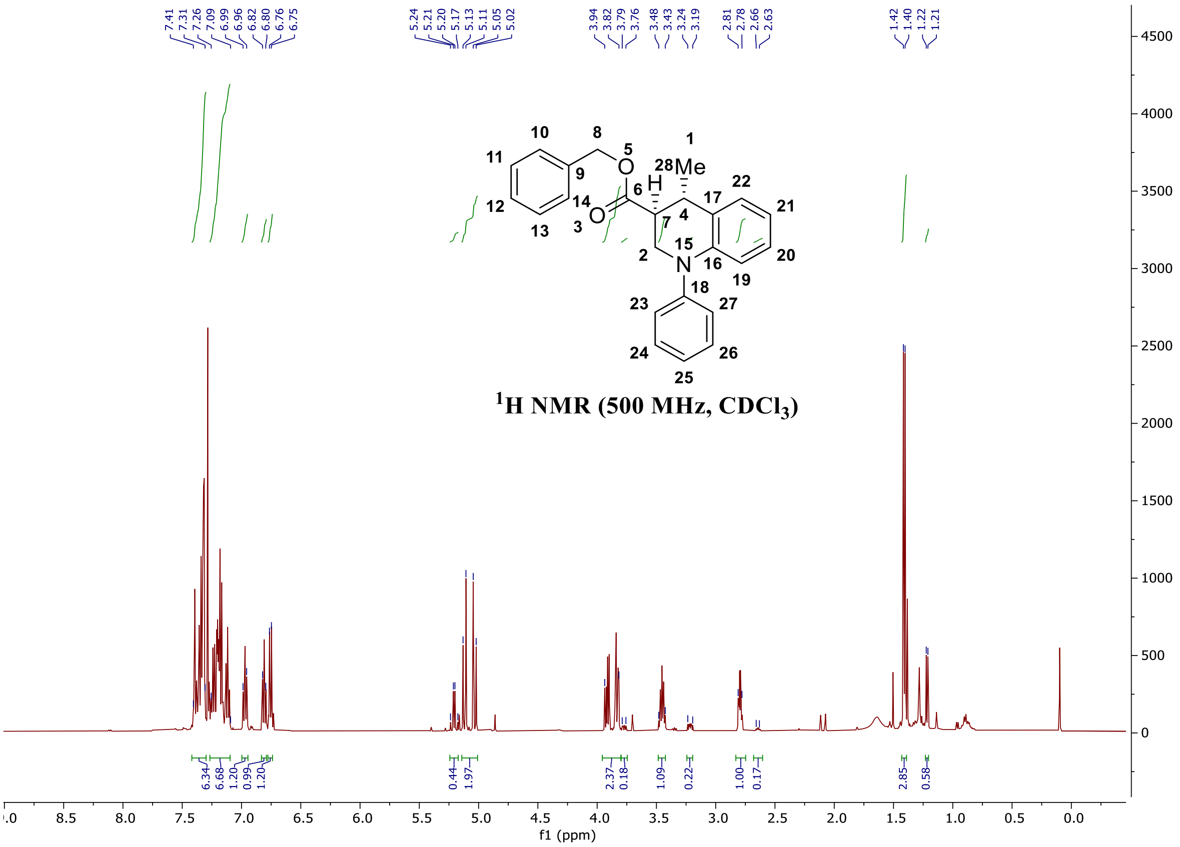

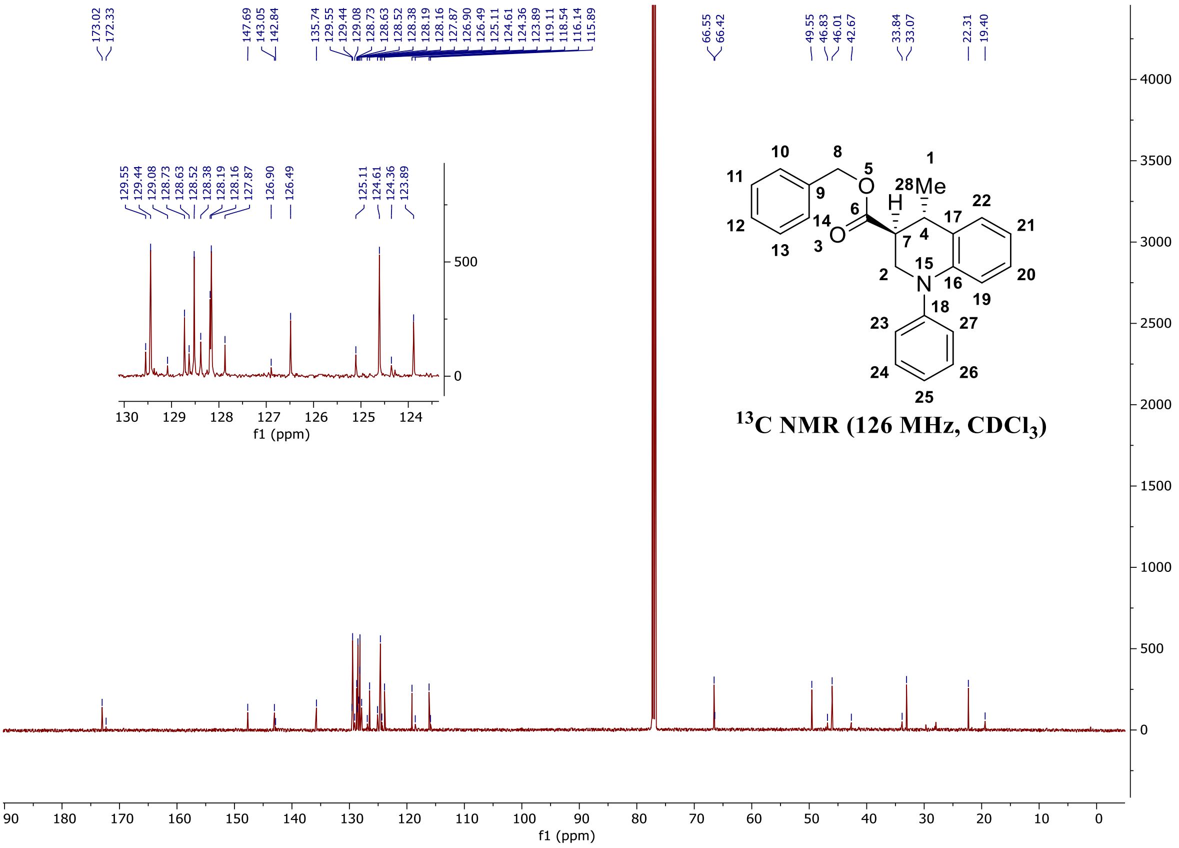

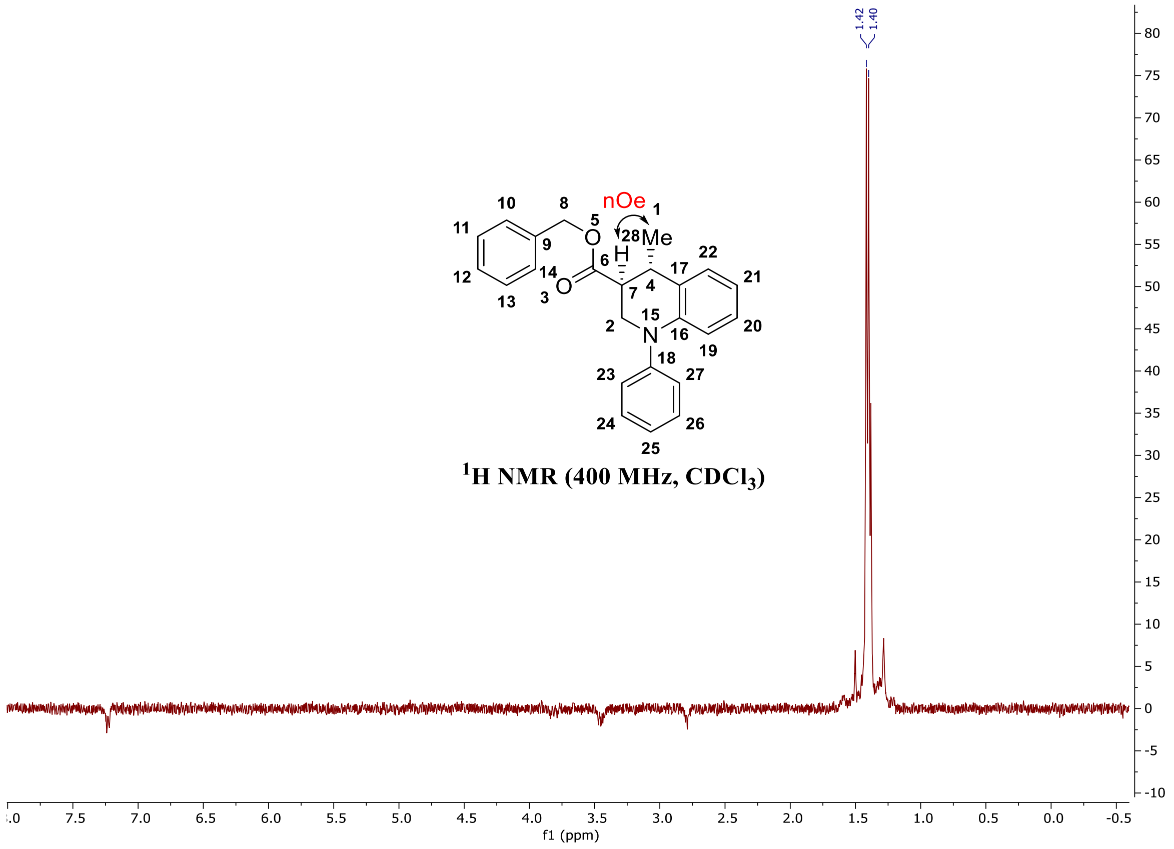

(*S*)-4-Methoxy-*N*-(3-phenylbutyl)aniline (8a):

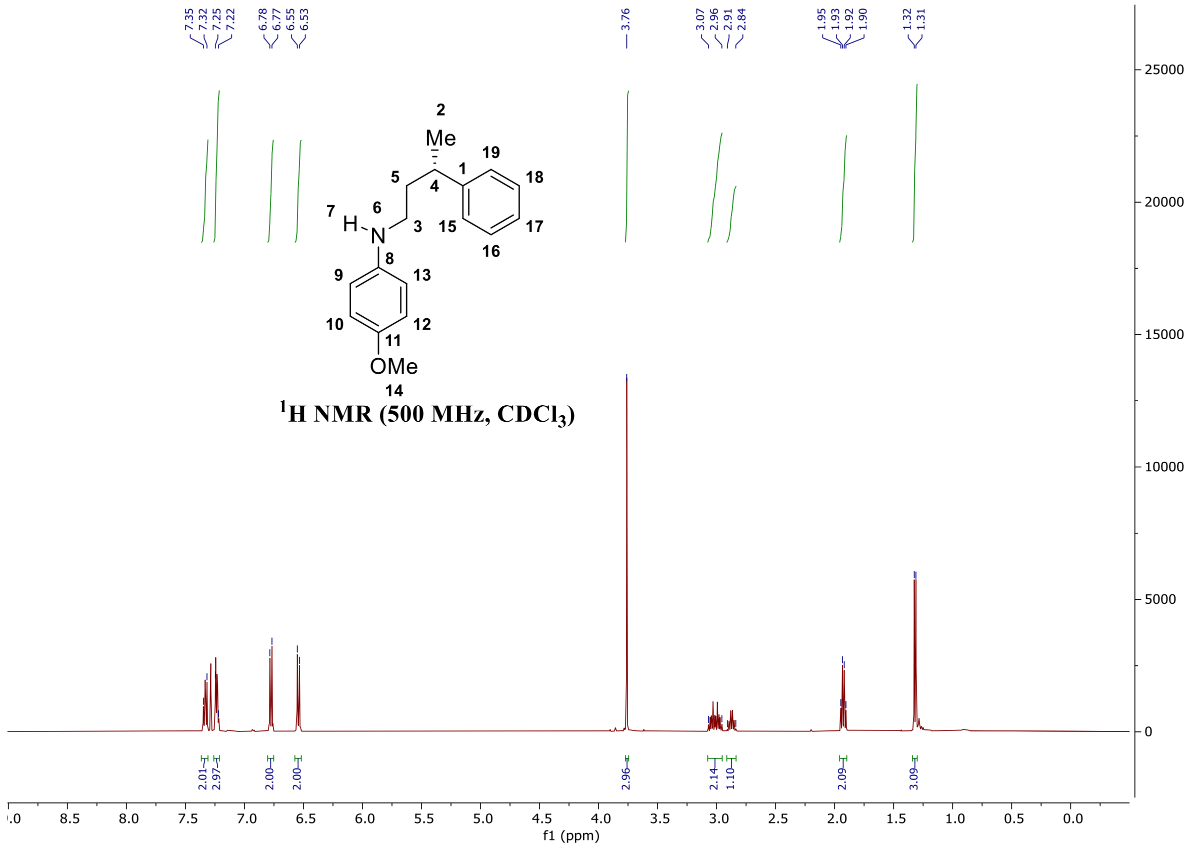

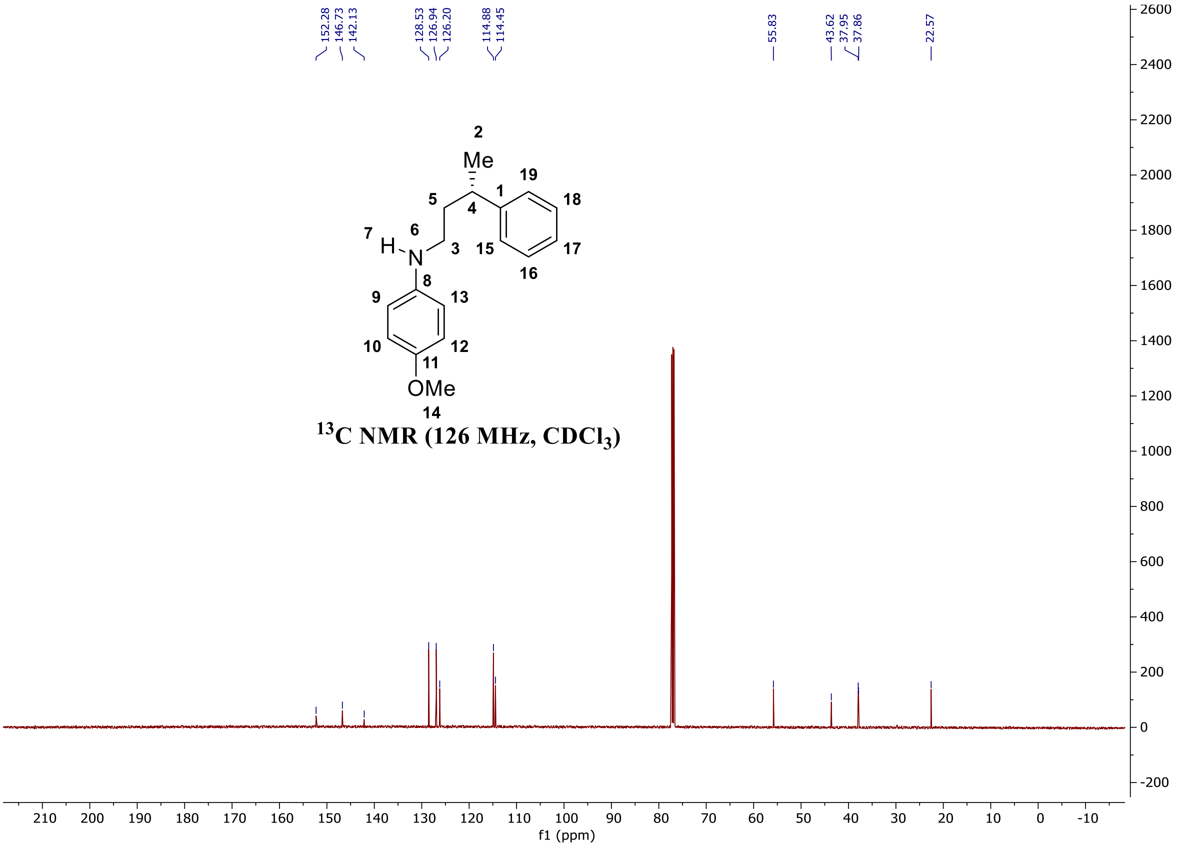

(*S*)-*N*-(3-([1,1'-Biphenyl]-4-yl)butyl)aniline (8b):

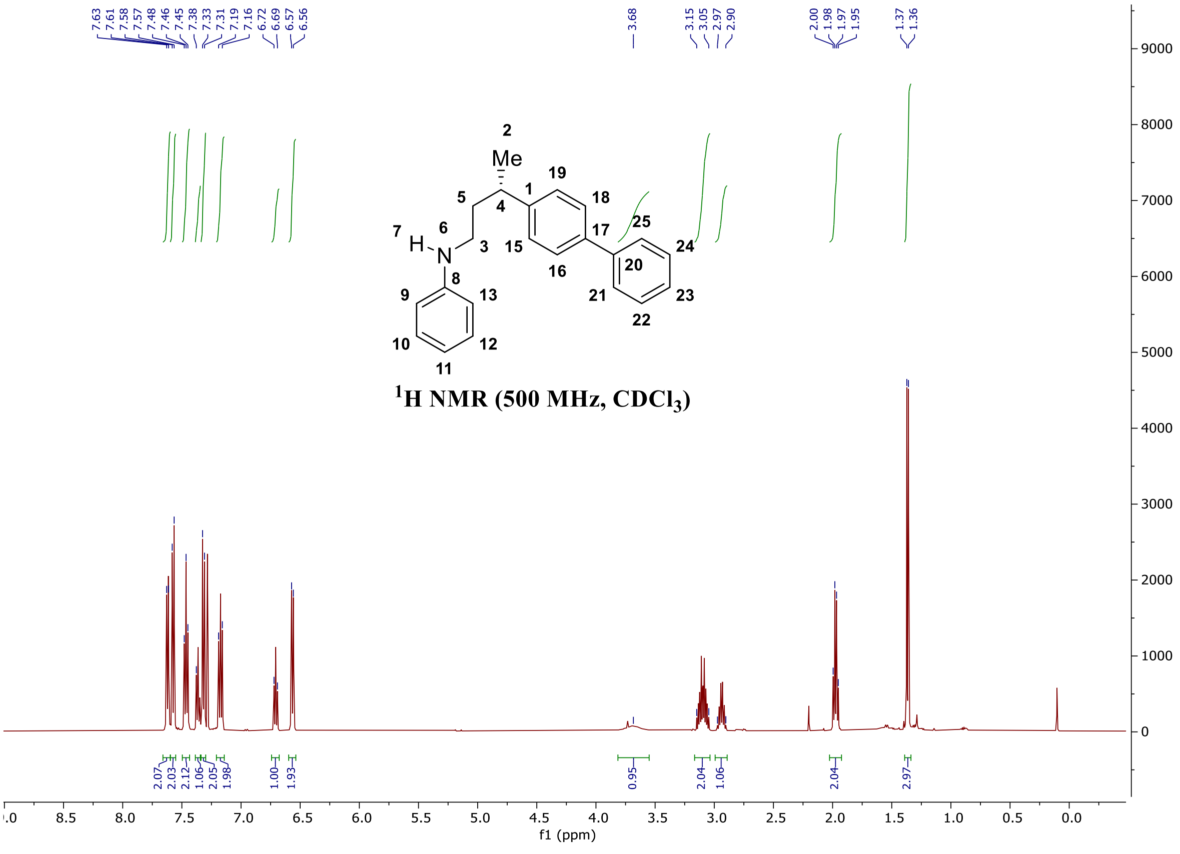

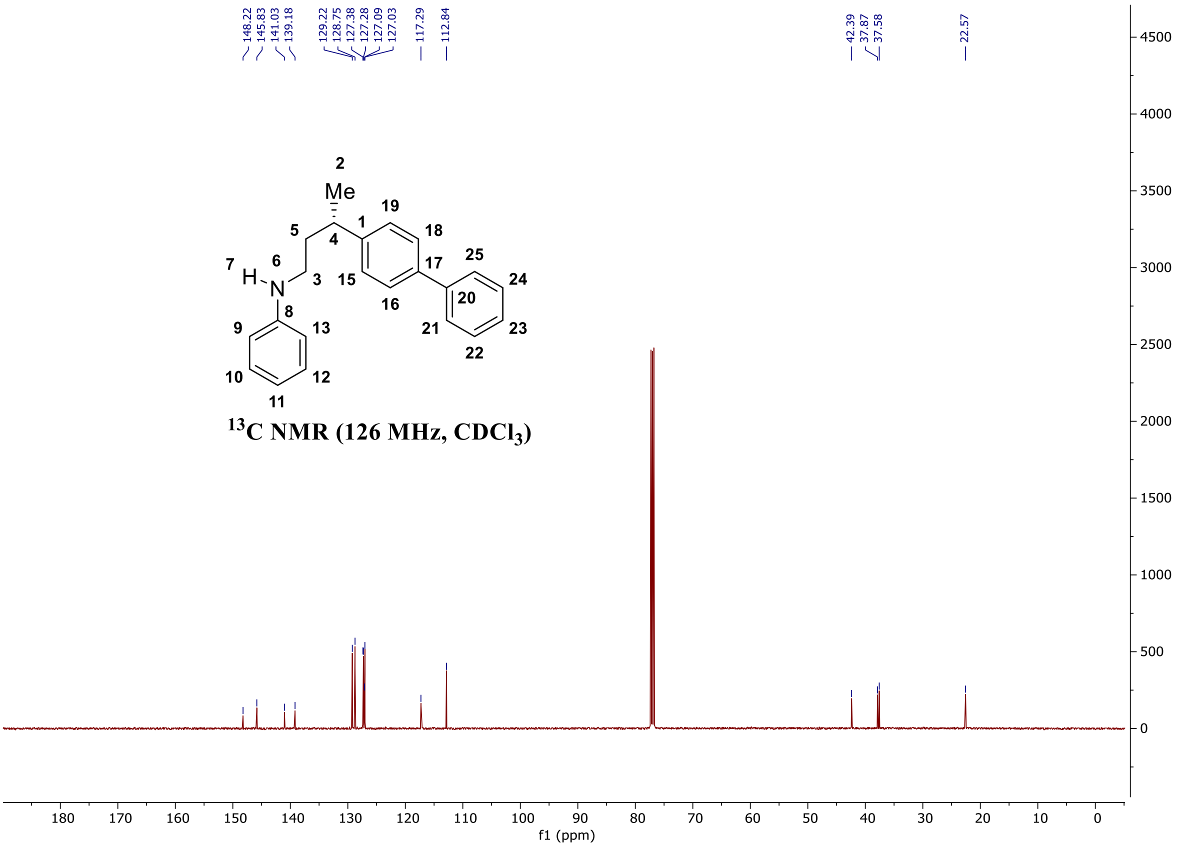

(*S*)-*N*-(3-(1-Tosyl-1*H*-indol-3-yl)butyl)aniline (8c):

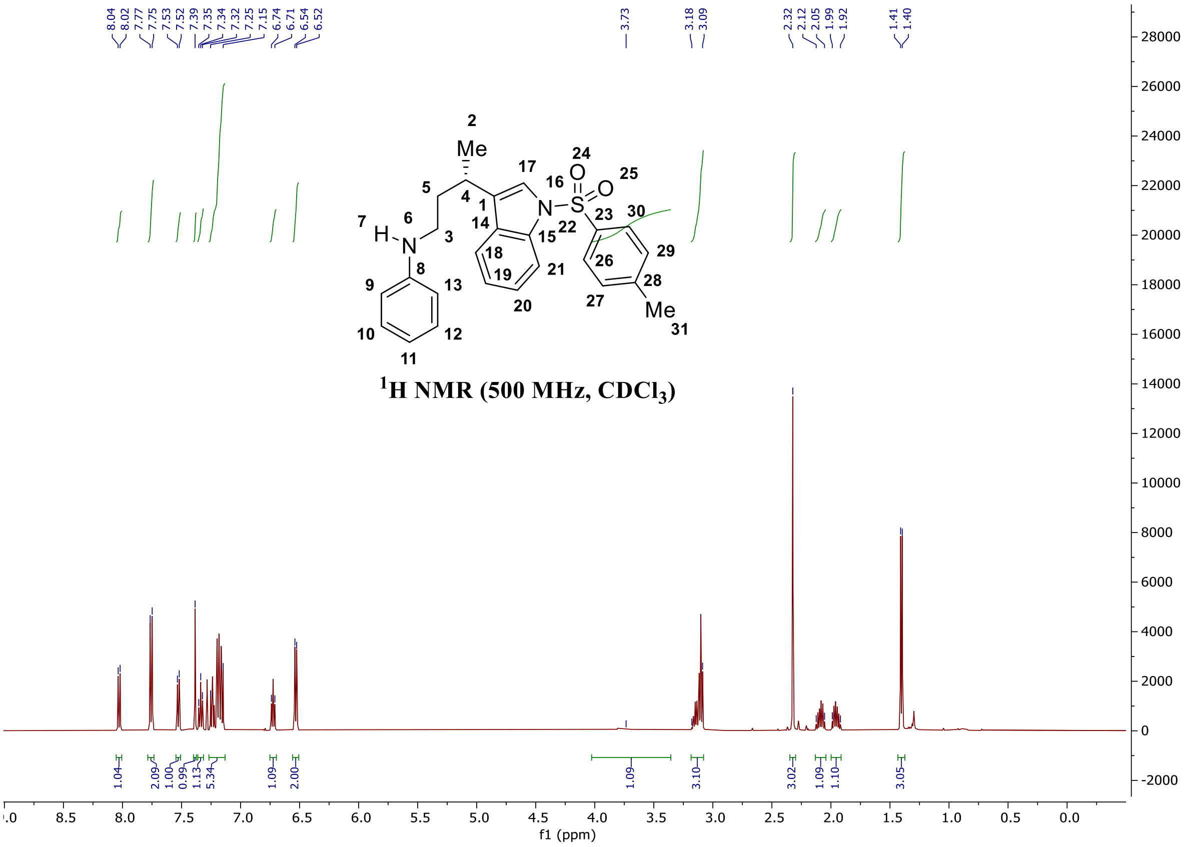

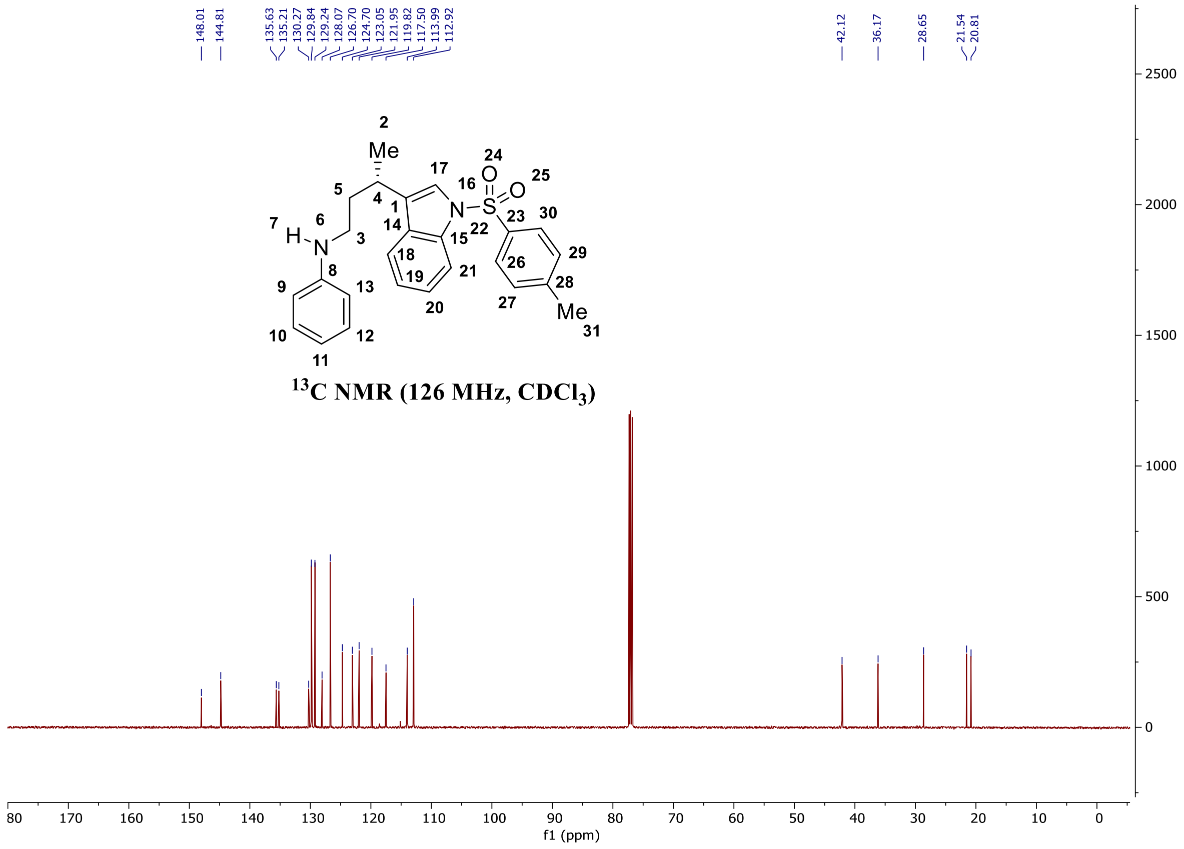
148.01
144.81
135.63
135.21
130.27
129.84
129.24
128.07
126.70
124.70
123.05
121.95
119.82
117.50
113.99
112.92
42.12
36.17
28.65
21.54
20.81
Me
NH
N
O
S
O
Me
13C NMR (126 MHz, CDCl3)
f1 (ppm)

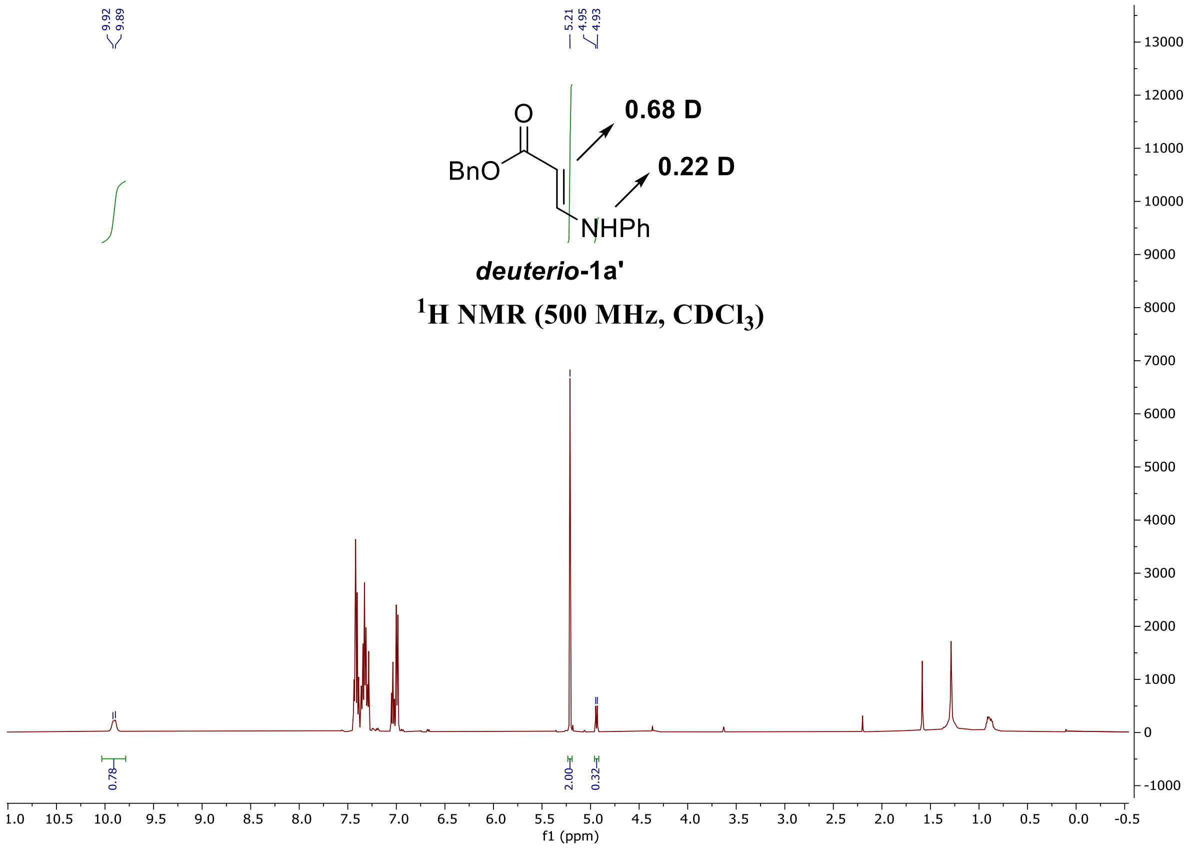
9.92
9.89
5.21
4.95
4.93
O
BnO
0.68 D
0.22 D
NHPh
deuterio-1a'
1H NMR (500 MHz, CDCl3)
0.78
2.00
0.32
f1 (ppm)

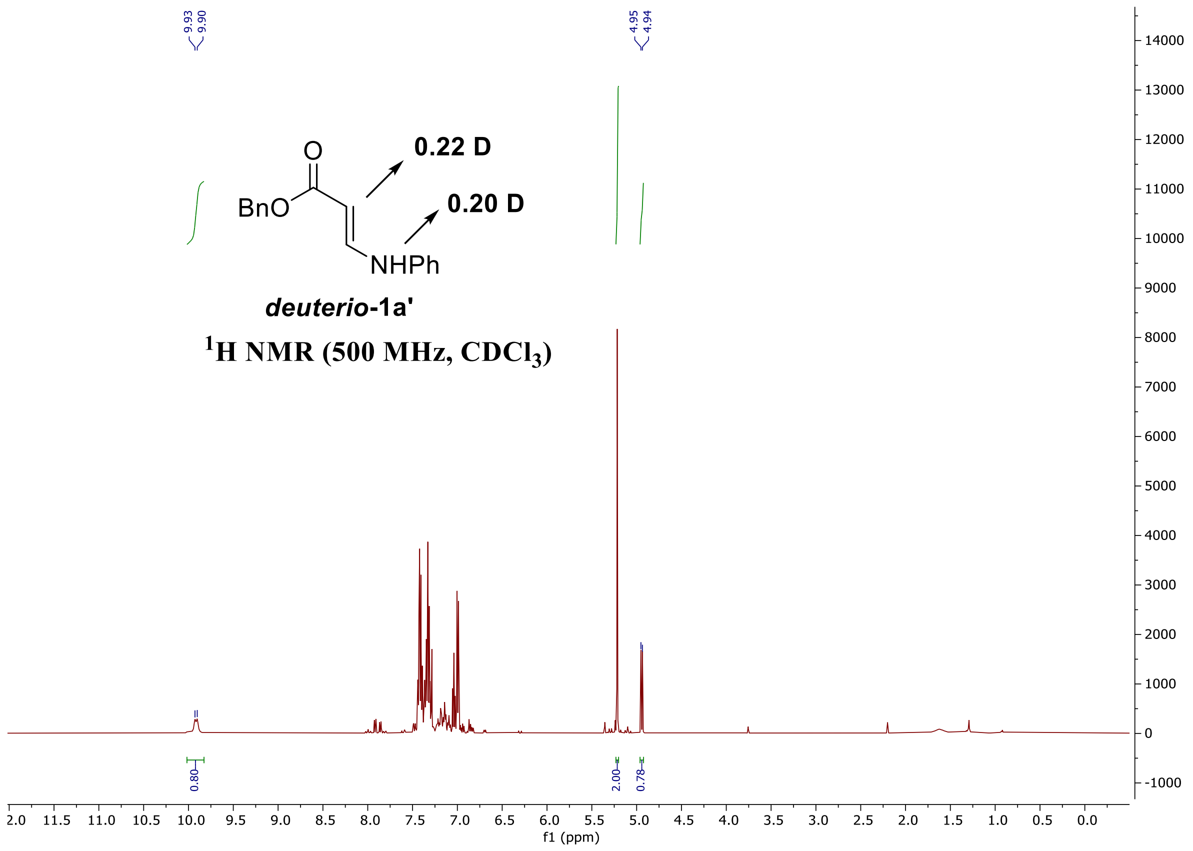
0.22 D
0.20 D
BnO
NHPh
deuterio-1a'
1H NMR (500 MHz, CDCl3)
f1 (ppm)

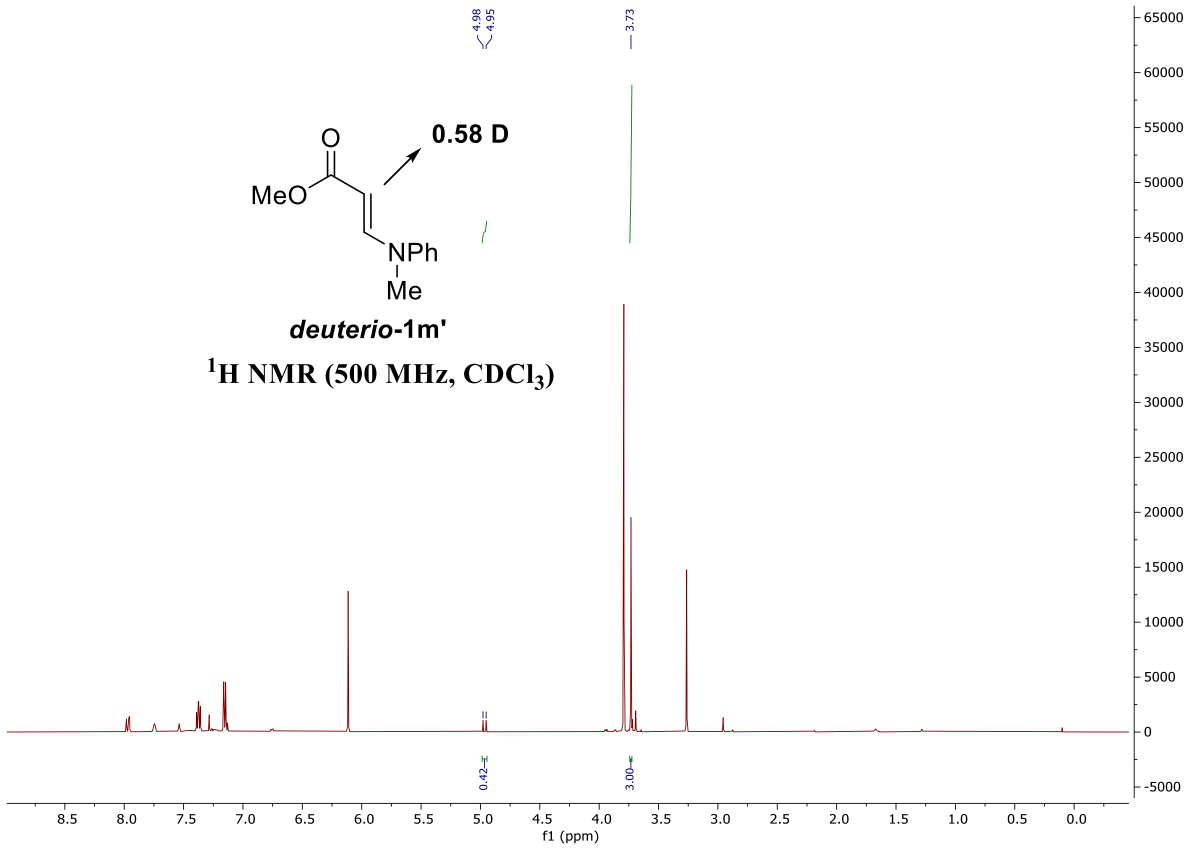
0.58 D
MeO
NPh
Me
deuterio-1m'
1H NMR (500 MHz, CDCl3)
f1 (ppm)

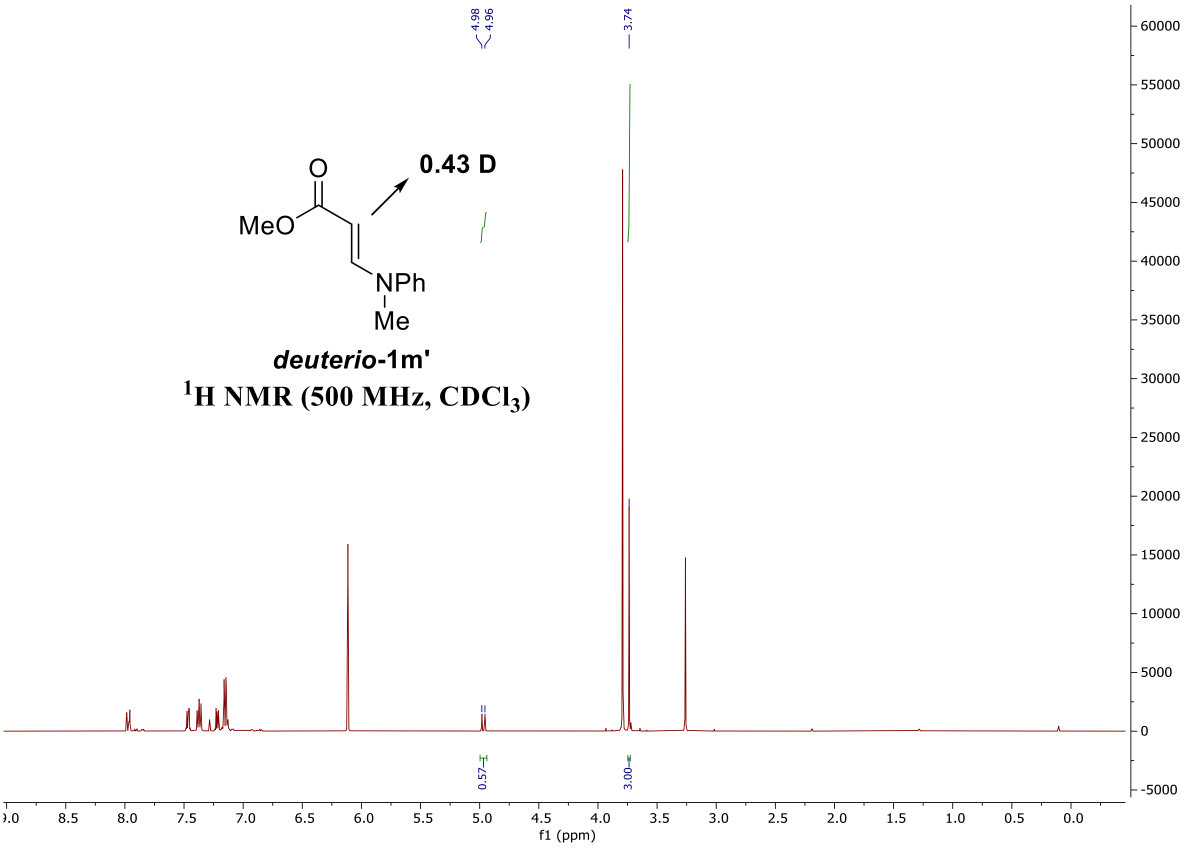
0.43 D
MeO
O
NPh
Me
deuterio-1m'
1H NMR (500 MHz, CDCl3)
f1 (ppm)

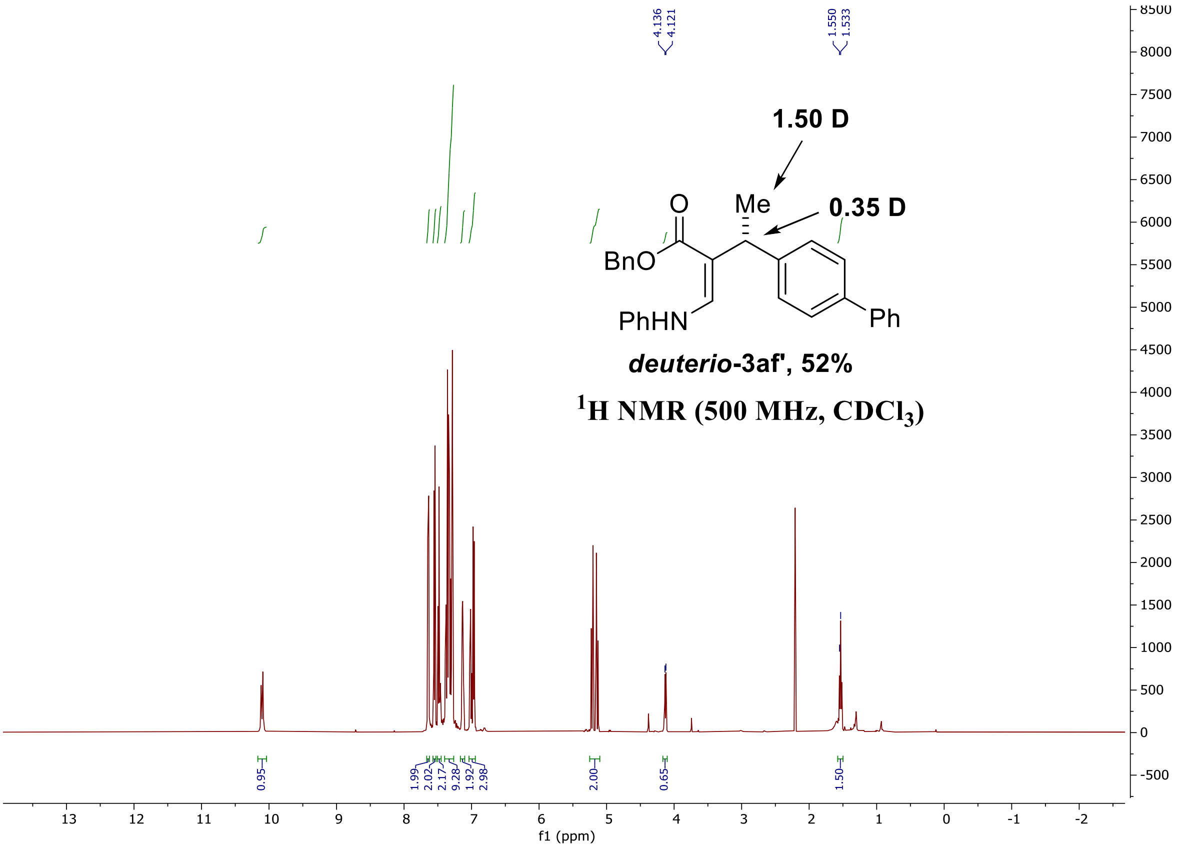
1.50 D
0.35 D
O
Me
BnO
PhHN
Ph
deuterio-3af', 52%
1H NMR (500 MHz, CDCl3)
f1 (ppm)

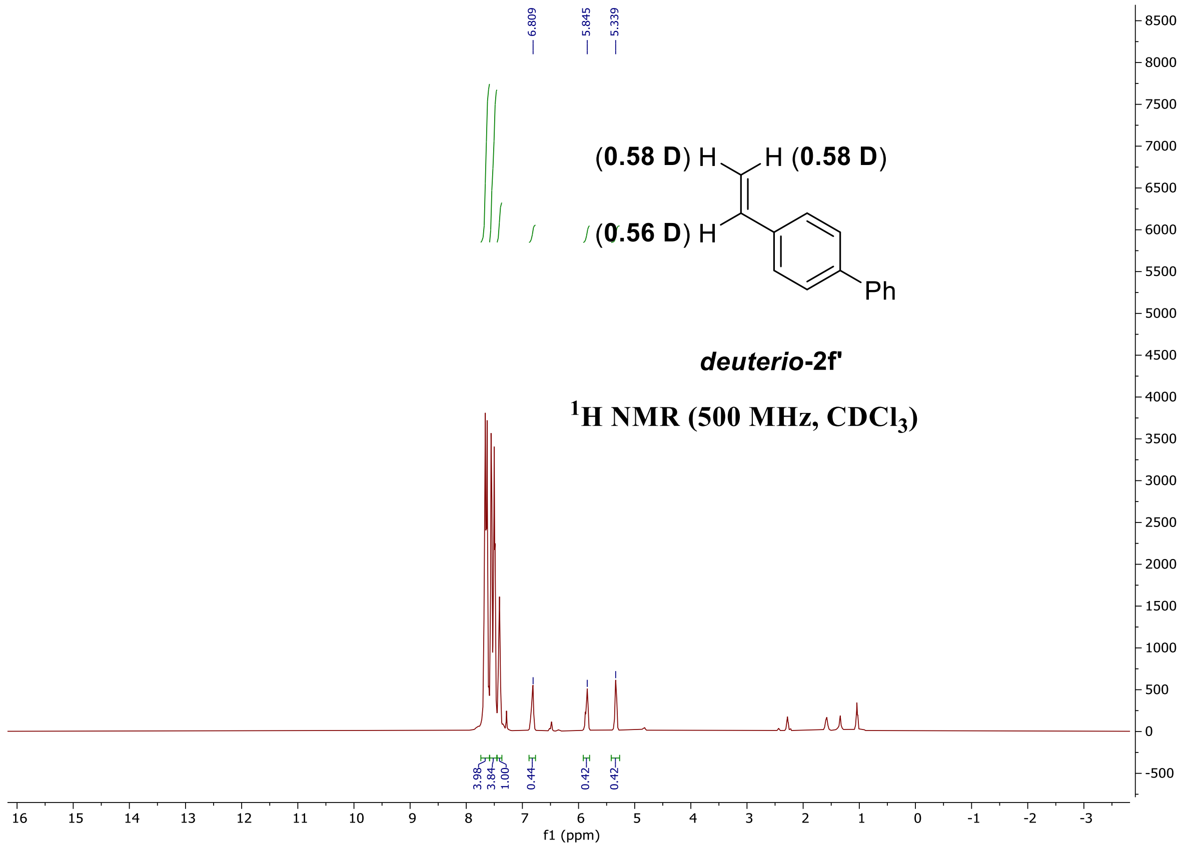

6.809
5.845
5.339
(0.58 D) H
H (0.58 D)
(0.56 D) H
Ph
deuterio-2f'
1H NMR (500 MHz, CDCl3)
3.98
3.84
1.00
0.44
0.42
0.42
f1 (ppm)
